# Enigmazole C: A Cytotoxic Macrocyclic Lactone and Its Ring-Opened Derivatives from a New Species of *Homophymia* Sponge

**DOI:** 10.1021/acs.jnatprod.1c01179

**Published:** 2022-03-02

**Authors:** Guillermo Tarazona, Rogelio Fernández, Marta Pérez, Ramón E. Millán, Carlos Jiménez, Jaime Rodríguez, Carmen Cuevas

**Affiliations:** † R&D, PharmaMar, Avenida De los Reyes, 1, Pol. Ind. La Mina-Norte, 28770-Colmenar Viejo, Madrid, Spain; ‡ Departmento de Química, Facultad de Ciencias and Centro de Investigacions Científicas Avanzadas (CICA), Universidade de A Coruña, 15071 A Coruña, Spain

## Abstract

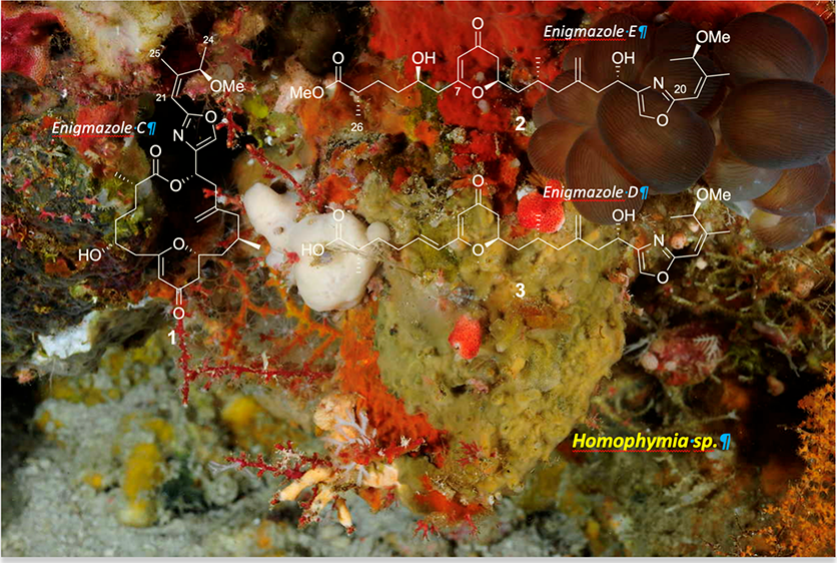

A new macrolide, enigmazole C (**1**), and two additional analogues, enigmazoles E (**2**) and D (**3**), were obtained from a new species of the *Homophymia* genus as part of an ongoing discovery program at PharmaMar to study cytotoxic substances from marine sources. The structures were fully characterized by cumulative analyses of NMR, IR, and MS spectra, along with density functional theory computational calculations. All three of the new compounds feature an unusual 2,3-dihydro-4*H*-pyran-4-one moiety, but only enigmazoles C (**1**) and D (**3**) showed cytotoxic activity in the micromolar range against A-549 (lung), HT-29 (colon), MDA-MB-231 (breast), and PSN-1 (pancreas) tumor cells.

Marine sponges represent a prolific source of structurally unique macrolides possessing promising biological activities, including cytotoxic, anticancer, and neuroprotective properties, thus suggesting their potential value for the development of leads in drug discovery.^1^ Particularly, sponges from the Neopeltidae family have not been extensively chemically investigated, with relatively few structures being described in the literature from each of the three genera *Homophymia*, *Callipelta*, and *Daedalopelta*. By way of illustration, the high molecular weight peptides homophymines A–E/A1–E1,^2,3^ homophymamide A,^4^ pipecolidepsins A–C,^5−7^ and callipeltins A and B,^8−10^ have all been reported to have significant cytotoxic and anti-HIV activities. Others metabolites isolated from Neopeltidae sponges that were assigned in 2013 to the suborder Astrophorina^11,12^ are the bioactive tetramic acid glycoside aurantoside C^13^ and the macrocycles callipeltosides A–C.^14,15^ Furthermore, only two structures belonging to the *Daedalopelta* genus have been described, the cytotoxic cyclodepsipeptide daedophamide^16^ and the 14-membered macrolide neopeltolide,^17^ which has been extensively studied and synthesized because it is a potent cytochrome bc1 complex inhibitor, as well as a cytotoxic compound with activity against A-549 human lung adenocarcinoma, NCI/ADR-Res ovarian sarcoma, and P388 murine leukemia cell lines.^18−21^

During continuing efforts at PharmaMar to discover new cytotoxic compounds from marine natural sources, we have evaluated a new sponge species of the *Homophymia* genus (Vacelet & Vasseur, 1971) collected off the coast of Gorontalo, Indonesia. In this paper, we describe the isolation of a new macrocyclic compound as well as two open-chain analogues, all isolated from a specimen of this sponge collected in Indonesia. Despite the fact that the new macrocycle shows structural similarities to neopeltolide, a clear resemblance to the macrolide enigmazole A isolated by Oku et al. in 2010 from the marine sponge *Cynachyrella enigmatica*,^22^ has led us to designate the three new compounds as enigmazoles C (**1**), E (**2**), and D (**3**). The configurations of these enigmazoles have been solved using a combination of microscale chemical conversions and the use of an elegant *J*-based configurational analysis based on capillary NMR measurements.^23^

**Chart 1:**
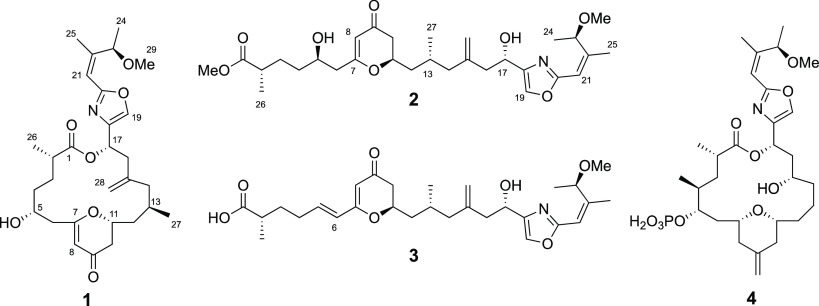
Structures of Enigmazoles C (**1**), E (**2**), and D (**3**) and Known Enigmazole A (**4**)

A methanolic extract of the *Homophymia* sponge specimen showed cytotoxic activity against A-549 (lung), HT-29 (colon), MDA-MB-231 (breast), and PSN-1 (pancreas) tumor cells and was hence selected for a more detailed bioassay-guided chemical investigation.

Careful fractionation of the MeOH extract led to isolation of the new macrolide enigmazole C (**1**, 2.9 mg), and two open-chain derivatives, enigmazole D (**3**, 0.4 mg) and the methanolic adduct enigmazole E (**2**, 1.4 mg), which were purified by semipreparative HPLC.

Enigmazole C (**1**) was isolated as a colorless amorphous solid and showed a [M + H]^+^ ion at *m*/*z* 516.2964 (calcd for C_29_H_42_NO_7_*m*/*z* 516.2956) in its (+)-HR-ESITOF-MS spectrum. The presence of the sodium adduct at *m*/*z* 538.2785 (calcd for C_29_H_41_NO_7_Na *m*/*z* 538.2775) confirmed the molecular formula and 10 degrees of unsaturation required for this compound.

NMR experiments of **1** were carried out in CD_3_CN because a chemical transformation was observed using CD_3_OD. ^1^H, ^13^C, and edited-HSQC NMR analysis of **1** revealed the presence of 29 carbons assigned to seven sp^2^ nonprotonated carbons (δ_C_ 193.6, 175.6, 175.5, 161.2, 152.7, 143.9, and 141.7), three sp^2^ methine carbons (δ_H_/δ_C_ 7.63/135.9, 6.20/113.6, and 5.30/106.8), a disubstituted exomethylene moiety (δ_H_/δ_C_ 4.91; 4.86/113.9), six sp^3^ methine carbons (δ_H_/δ_C_ 5.92/66.7, 5.20/75.5, 4.48/77.8, 3.74/69.5, 2.49/39.9, and 2.20/25.8), seven sp^3^ methylene carbons (δ_H_/δ_C_ 2.72; 2.62/42.2, 2.49; 2.27/44.6, 2.49; 1.59/40.2, 2.41; 2.23/42.2, 1.81; 1.46/42.9, 1.69; 1.63/30.8 and 1.43; 1.31/34.5), and five methyl groups (δ_H_/δ_C_ 3.15/56.6 (OMe), 1.86/17.7, 1.23/19.5, 1.10/17.6, and 0.95/21.2).

Two-dimensional COSY, 2D-TOCSY, and selective 1D-TOCSY experiments of **1** allowed us to identify five different spin systems, fragments **A** (C23–C24), **B** (C21–C25), **C** (C16–C19), **D** (C2–C6, C26), and **E** (C10–14, C27) (Figure 1). Special attention was paid to fragments **D** and **E** due to the presence of four stereogenic centers. Thus, the spin system **D** was identified by selective 1D-TOCSY from selective irradiation of proton H-5 at δ_H_ 3.74, which gave responses to the methylene protons at δ_H_ 1.69/1.63 (H_2_-3), δ_H_ 1.43/1.31 (H_2_-4), and δ_H_ 2.49/2.27 (H_2_-6), as well as the methine at δ_H_ 2.49 (H-2), the methyl group at δ_H_ 1.10 (H_3_-26), and a broad signal at δ_H_ 3.00 assigned to a hydroxy proton (Figure 2b).

**Figure 1:**
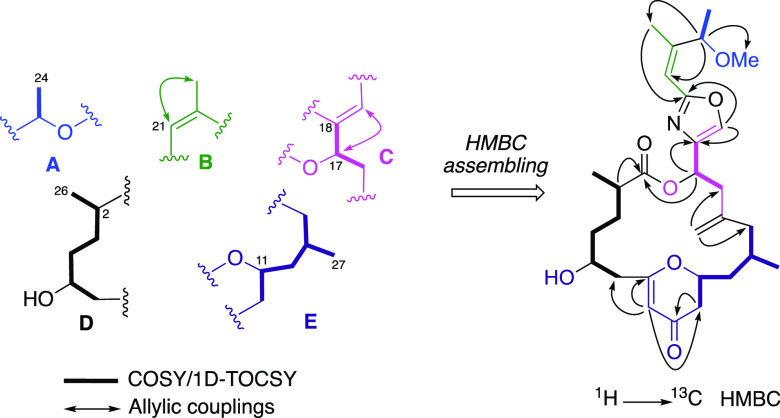
Spin systems deduced by COSY and 1D-TOCSY experiments and HMBC assembling in **1**.

**Figure 2:**
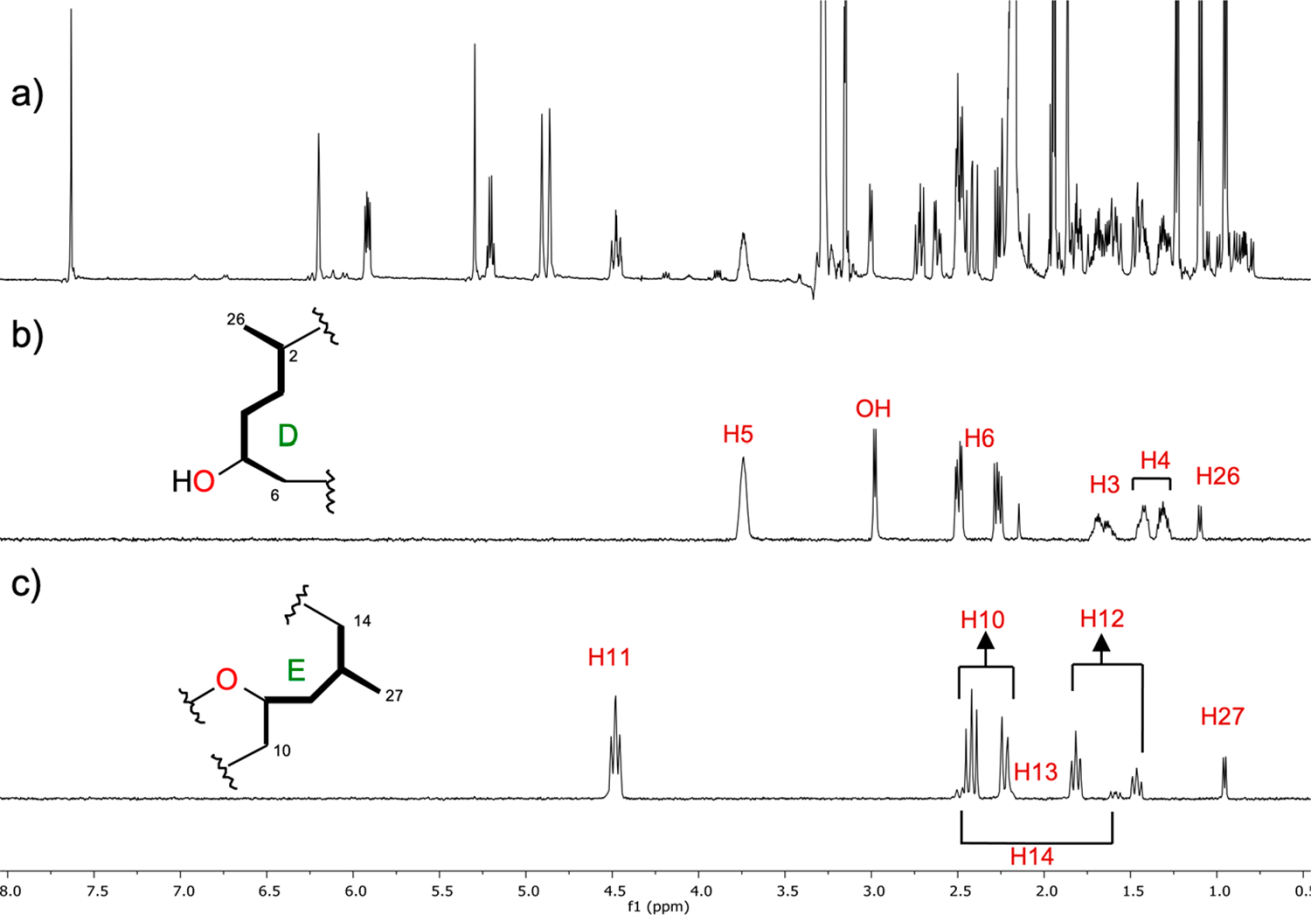
(a) NMR spectrum of enigmazole C (**1**) in CD_3_CN at 500 MHz. Selective 1D-TOCSY irradiation experiments at (b) δ_H_ 3.74 (H-5) and (c) δ_H_ 4.48 (H-11) to identify fragments **D** and **E**.

In the same way, fragment **E** was deduced by selective 1D-TOCSY irradiation of H-11 at δ_H_ 4.48, which showed coupling responses to three methylenes at δ_H_ 2.41/2.23 (H_2_-10), δ_H_ 1.81/1.46 (H_2_-12), and δ_H_ 2.49/1.59 (H_2_-14), the methine H-13 (δ_H_ 2.20), and the methyl group δ_H_ 0.95 (H_3_-27) (Figure 2c).

The interconnection between the determined fragments **A**–**E** was achieved by using an HMBC experiment as follows:

(a) The methine at δ_H_ 5.20 (H-23) showed long-range correlation with three methyl groups at δ_C_ 19.5 (C-24), δ_C_ 17.7 (C-25), and δ_C_ 56.6 (C-29), along with the sp^2^ methine carbon at δ_C_ 113.6 (C-21), implying the position of the methoxy group on C-23 and the connection between fragments **A** and **B**.
(b) The HMBC cross-peak observed from H-21 (δ_H_ 6.20) to C-22 (δ_C_ 152.7) placed a nonprotonated carbon in the double bond. On the other hand, the singlet at δ_H_ 7.63 (δ_C_ 135.9) presented a typical chemical shift and ^1^*J*_CH_ of 210 Hz of a 2,4-disubstituted oxazole ring, which was completed by inspection of the HMBC correlations observed between the oxazolic proton H-19 to carbons C-18 (δ_C_ 141.7) and C-20 (δ_C_ 161.2). The connection between fragments **B** and **C** was undoubtedly determined by the HMBC cross-peak from H-25 (δ_H_ 1.86) to C-20 (δ_C_ 161.2).
(c) Fragments **D** and **E** were connected through a six-membered ring by the cross-peaks observed from δ_H_ 5.30 (H-8) to δ_C_ 44.6 (C-6), δ_C_ 42.2 (C-10), and to a nonprotonated oxygenated sp^2^ carbon at δ_C_ 175.5 (C-7). A dihydropyranone ring was deduced by the observation of the HMBC correlations from both diastereotopic protons at H-10_a_ (δ_H_ 2.41) and H10_b_ (δ_H_ 2.23) to an α,β-unsaturated carbonyl signal at δ_C_ 193.6.
(d) Fragments **C** and **E** were linked by a disubstituted exomethylene group as the HMBC correlations observed of H-28 (δ_H_ 4.86) with C-14 (δ_C_ 40.2) and C-16 (δ_C_ 42.2).
(e) A cross-peak from H-17 (δ_H_ 5.92) to C-1 (δ_C_ 175.6) allowed us to connect fragments **C** and **D**, therefore assembling the cyclic macrolactone planar structure for **1** as is drawn in Figure 1.

At this point, the resemblance of **1** to the known compound enigmazole A (**4**) was clear, as some structural features are common in both compounds: a disubstituted oxazole ring, a pyran ring, the presence of an exomethylene double bond, and a similar macrolactone size. Once the planar structure of **1** was established, the relative and absolute configuration were determined by ROESY experiments, derivatization reactions, and computational calculations.

First, the *Z* configuration of the C-21/C-22 double bond was deduced from the ROESY correlation between H_3_-25 (δ_H_ 1.86) and H-21 (δ_H_ 6.20).

To deduce the relative configuration of the entire spin system **D**, we were nicely able to relate the two stereogenic centers at C-2 and C-5 located three carbon–carbon bonds away through a *J*-based configurational analysis (JBCA).^24^ To make this possible, we had to change the NMR solvent to acetone-*d*_6_, because it gave us a ^1^H NMR spectrum where the two diastereotopic pairs at C-3 (H-3a and H-3b) and C-4 (H-4a and H-4b) are well separated and fully resolved.

Not many approaches of this kind have been applied to a natural compound with two sp^3^ methylenes surrounded by two stereogenic centers, even though this approximation is a very good tool when the diastereotopic protons can be unequivocally assigned with their corresponding chemical shifts and their sets of proton–proton and carbon–proton coupling constants. Therefore, ^13^C–^1^H-HSQC-TOCSY-HECADE and *J*-HMBC experiments were needed to measure key small coupling constants for ^3^*J*_C26–H3a_ and ^3^*J*_C26–H3b_ to place H_3_-26 in a *gauche* disposition to both H-3 diastereotopic protons at the C-2/C-3 single bond (Figure 3a). These two experiments along with selective ^1^H NMR irradiation spectra were satisfactory to deduce relationships from diastereotopic protons H-3a/H-3b and H-4a/H-4b to C-2 and C-5 as is drawn Figure 3b) for the C-3/C-4 bond. The hydroxylated carbon at C-5 helped us to deduce the relative dispositions for H-4a and H-4b to the OH group with regard to the C-4/C-5 bond (Figure 3c), as well as H-6a and H-6b to the mentioned OH relative to the C-5/C-6 bond (Figure 3d).

**Figure 3:**
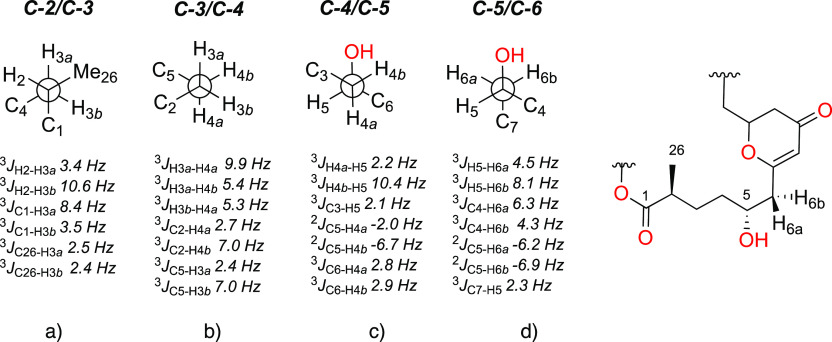
C-2 to C-6 fragment deduced by *J*-based configurational analysis.

The absolute configuration at position C-5 was elucidated by the application of the modified Mosher’s method (MMM).^25,26^ The derivatization of the secondary alcohol with *R*- and *S*-MTPA-Cl was completed directly in pyridine-*d*_5_ in an NMR tube to give compounds **1-***S* and **1-***R*, respectively. Comparison of the Δδ values (δ_*S*_ – δ_*R*_) obtained from the MTPA esters (Figure 4) indicated that the absolute configuration of C-5 was *R*, which implies a C-2*S* configuration.

**Figure 4:**
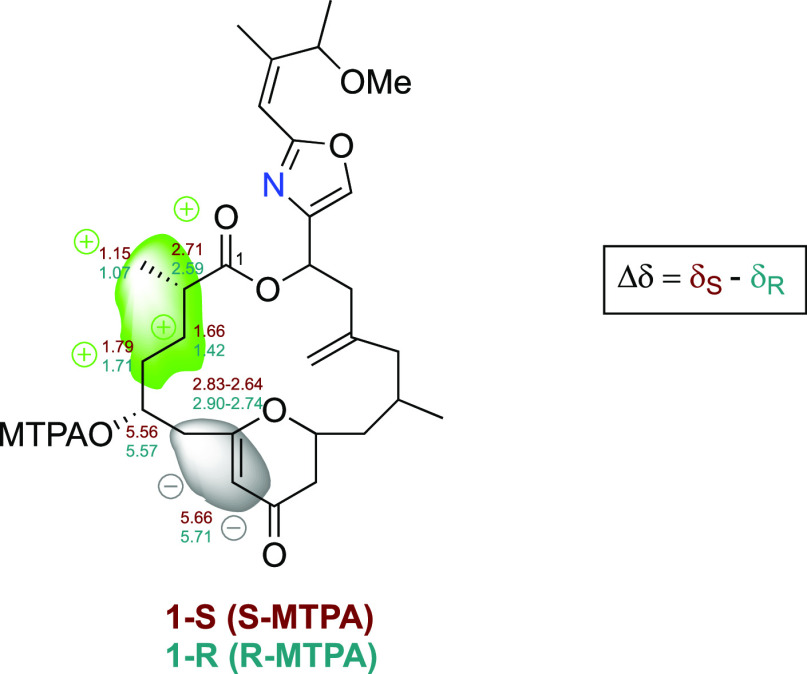
Mosher’s derivatives of enigmazole C (**1**).

During the process of purification of enigmazole C, the use of MeOH in the HPLC separation procedure induced a macrolactone ring-opening that produces the methyl ester adduct **2**, which we have named as enigmazole E.

The (+)-HR-ESIMS-TOF data of **2** at *m*/*z* 548.3249 [M + H]^+^ (calcd for C_30_H_46_NO_8_, *m*/*z* 548.3218) and the ^1^H NMR of **2** revealed the opening of the macrolactone ring.

Thus, the ^1^H NMR spectrum of **2** displays a singlet signal at δ_H_ 3.61 (s, 3H) that was assigned to a methoxy group, which shows an HMBC correlation to an carbonyl carbon at δ_C_ 176.6 assigned to the new carbonyl ester at C-1. Moreover, the proton chemical shift at δ_H_ 5.92, assigned to H-17 in **1**, was clearly shifted to δ_H_ 4.72 in **2** as expected due to the lack of the lactone moiety. The presence of a hydroxy group at C-17 in **2** due to the macrolactone ring opening of **1** could be used to determine the absolute configuration at this position by the application of the MMM using the α-methoxy-α-trifluoromethylphenylacetic (MTPA) esters.

In this case, the comparison between the proton chemical shifts of both MTPA esters at C-17 (δ_**2-**_*S* – δ_**2-**_*R*) established the absolute configuration of C-17 as *S* (see Figure 5). In addition, the esterification of **2** with MTPA-Cl generated also the MTPA esters at C-5; the comparison of proton chemical shift values confirmed again the configuration of this stereogenic center as *R*.

**Figure 5:**
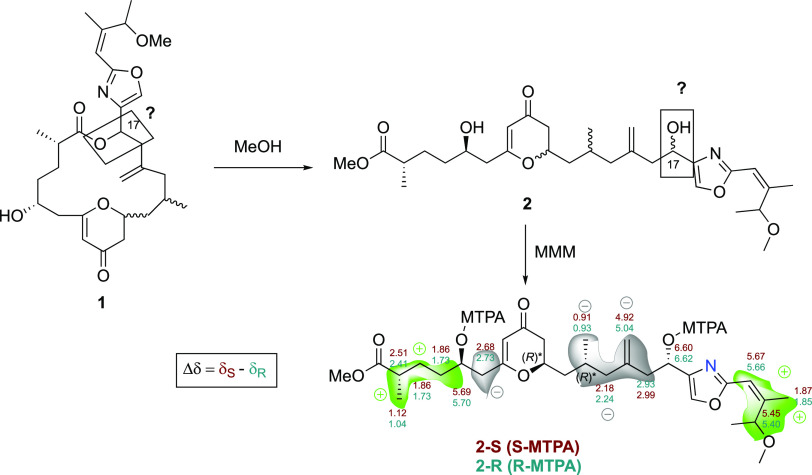
Modified Mosher methodology (MMM) applied to enigmazole E (**2**) to deduce the absolute configuration at C-17 and confirming the absolute configuration at C-5.

To establish the configuration between C-11 and C-13 at **1**, and therefore of **2**, homonuclear coupling constants of H-11/H-12b, H-11/H-12a, H-12b/H-13, and H-12a/H-13 were extracted from selective 1D-TOCSY experiments (Figure 2c). The multiplicity observed for H-11 as a dddd with two large and two small couplings (^3^*J*_H11–H10a_ 14.1 Hz, ^3^*J*_H11–H10b_ 2.7 Hz, ^3^*J*_H11–H12b_ 11.0 Hz, and ^3^*J*_H11–H12a_ 2.6 Hz), the heteronuclear coupling constants (^3^*J*_C10–H12a_ 1.7 Hz, ^3^*J*_C10–H12b_ 1.7, ^2^*J*_C11–H12a_ −5.6 Hz ^2^*J*_C11–H12b_ 0 Hz), and the ROESY correlation observed between H-12b and H-10 allowed us to deduce the presence of the rotamer represented for C-11/C-12 bonds in Figure 6a.

**Figure 6:**
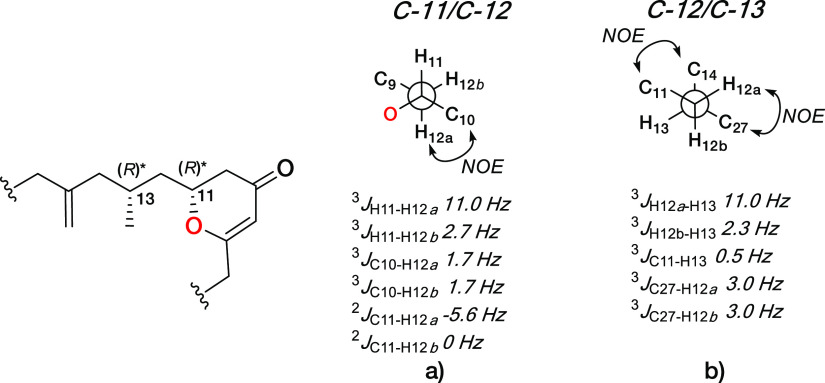
Rotamers along C-11/C-12 (a) and C-12/C-13 (b) bonds in enigmazole C (**1**).

In the same way, a large proton–proton coupling constant of 11.0 Hz between H-13 and H-12a fixed these protons in an *antiperiplanar* disposition, making necessary the use of ROESY correlations between the pairs H-12a/H-27 and H-11/H-14 and the long-range ^13^C–^1^H coupling constants ^3^*J*_C11–H13_ 0.5 Hz, ^3^*J*_C27–H12a_ 3.0 Hz, and ^2^*J*_C27–H12a_ 3.0 Hz to determine the presence of the rotamer drawn in Figure 6b. Once H-12a and H-12b are interrelated to conformers **6a** and **6b**, we were able to determine the relative configuration of the two stereogenic centers C-11 and C-13 as R* and R*.

While the absolute configurations of C-2, C-5, and C-17 are already known as *S*, *R*, and *S*, respectively, the relative configurations of C-11 and C-13 as R* and R* along with the possible configuration at C-23 of either *R* or *S* give us four possible enigmazole C diastereomers (**1a**–**d**) that can be discriminated by an NMR–density functional theory (DFT) approximation. Initially we submitted all diastereoisomers to a conformational search with the Macromodel program using the protocol of Daranas, Sarotti, et al.^27^ Thus, 58 conformers for **1a-**(2*S,*5*R,*11*R,*13*R,*17*S,*23*R*), 64 for **1b-**(2*S,*5*R,*11*S,*13*S,*17*S,*23*S*), 60 for **1c-**(2*S,*5*R,*11*S,*13*S,*17*S,*23*R*), and 62 for **1d-**(2*S,*5*R*,11*R,*13*R,*17*S,*23*S*) were found within a 5.0 kcal/mol window, which were further classified by energy and frequencies using the B3LYP/6-31G(d) functional. Once the duplicates and conformers with imaginary frequencies were removed, a combination of MPW1PW91/6-31G(d,p) and the polarizable continuum model was used for proton and carbon chemical shift calculations using MeCN as solvent. The sets of ^1^H and ^13^C chemical shifts were compared by the statistical DP4+ parameter developed by Sarotti and co-workers.^27,28^ A 100% probability DP4+ value for all chemical shifts was in agreement with diastereoisomer **1a-**(2*S*,5*R,*11*R,*13*R,*17*S,*23*R*) (Figure 7).

**Figure 7:**
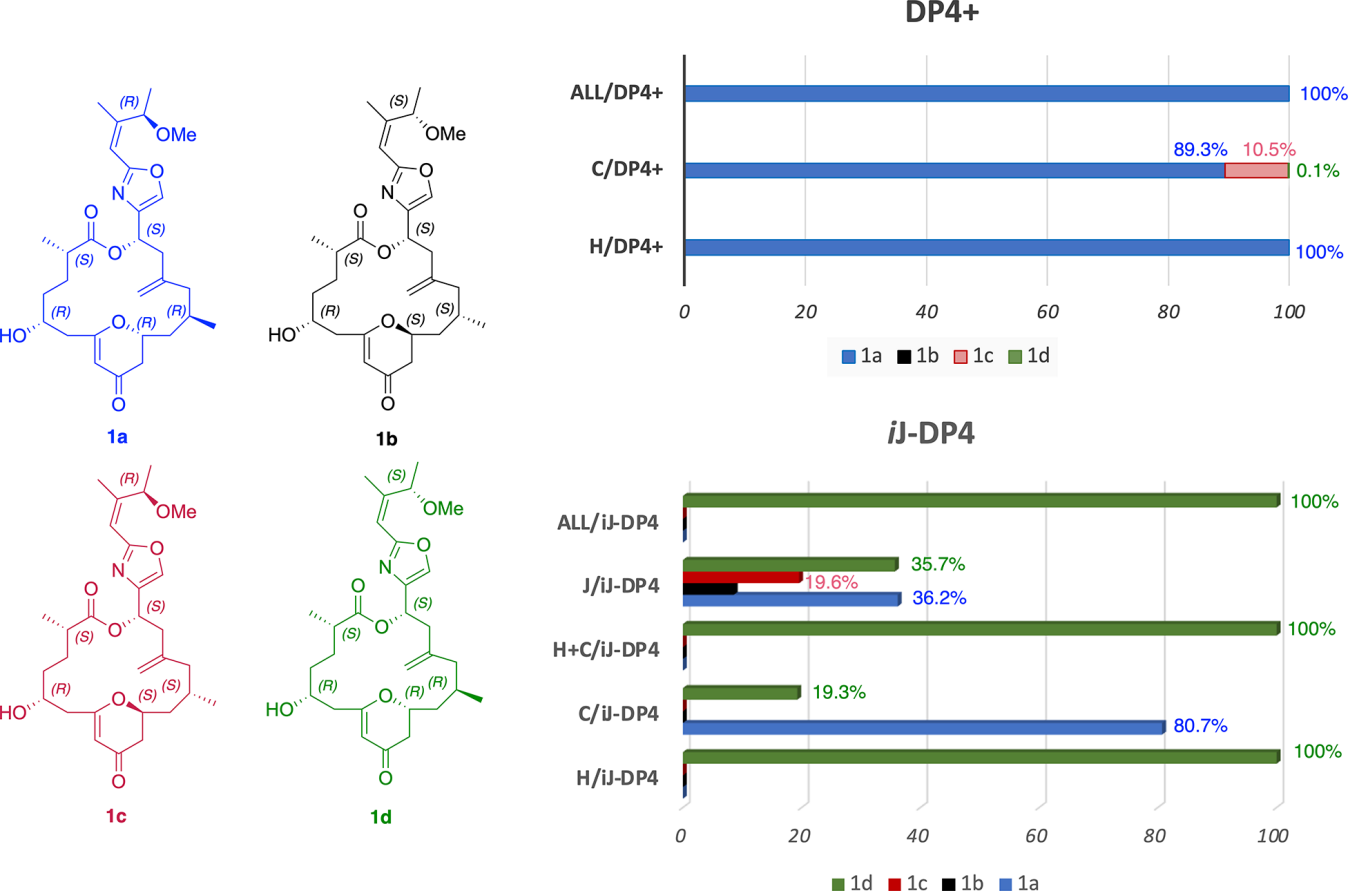
DP4+ and i*J*-DP4 statistical correlations of diastereoisomers **1a-**(2*S,*5*R*,11*R,*13*R,*17*S,*23*R*), **1b-**(2*S*,5*R,*11*S,*13*S,*17*S,*23*S*), **1c-**(2*S*,5*R,*11*S*,13*S,*17*S,*23*R*), and **1d-**(2*S,*5*R,*11*R,*13*R,*17*S,*23*S*) of enigmazole C.

However, when the i*J*-DP4 was computed using the suggested combination of the MMFF-5 kcal/mol conformational search and B3LYP/6-31G(d) for chemical shifts and coupling constants (just the contact Fermi contribution), no clear discrimination between isomers **1a-**(2*S,*5*R,*11*R,*13*R,*17*S,*23*R*) and **1d-**(2*S,*5*R,*11*R,*13*R,*17*S,*23*S*) was achieved when six ^3^*J*_HH_ constraints were introduced.^27^ Clearly both DP4+ and i*J*-DP4 were able to discriminate 11*R*,13*R* diastereoisomers **1a** and **1d** from both 11*S*,13*S*-**1b** and 11*S*,13*S***-1c**. At this point just C-23 remains to be assigned, and since the fragment C-18/C-25 showed similar NMR values (see chemical shifts in CD_3_OD in the Supporting Information) to that for enigmazole A (**4**),^22^ we propose the final structure for enigmazole C (**1**) as 2*S*,5*R*,11*R*,13*R*,17*S*,23*R* (diastereoisomer **1a**). In comparison with enigmazole A, **1** presents the same configurations of the stereogenic centers at the C-2, C-5, C-11, C-13, C-17, and C-23 positions.

Along with **1**, an analogue of **3**, enigmazole D, was also found during the process of isolation and purification. Its HR-ESITOF-MS data showed the [M + H]^+^ adduct at *m*/*z* 516.2932, which was in perfect agreement for a molecular formula of C_29_H_42_NO_7_. Although the formula of **3** matches that found for enigmazole C, its HPLC retention time and ^1^H NMR spectrum were different. Three new ^1^H NMR signals corresponding to two vinylic protons (δ_H_ 6.58 and 6.00) and one oxygenated methine at δ_H_ 4.74, in addition to the absence of the signals for H-17 (δ_H_ 5.92), H-5 (δ_H_ 3.74), and the OH group at δ_H_ 3.00 in **1**, lead us to suspect the opening of the macrolactone ring accompanied by an elimination of H_2_O. COSY correlations of the sp^2^ methine group at δ_H_ 6.58 with δ_H_ 6.00 and 2.21 and two extra signals observed from sp^2^ methines, with the loss of the hydroxy group, clearly suggested the formation of a new double bond in **3**. The movement of the signal at δ_H_ 5.92 (H-17) found in **1** to a lower value of δ_H_ 4.74 in **3** along with the expected chemical shift of the new methine proton is in agreement with the opening of the lactone ring. With all these data on hand, we were able to assign the structure of enigmazole E to **3**, a metabolite resulted from the opening of enigmazole C and a further dehydration at position C-5. It is important to notice that this new double bond has an *E* configuration, based on the 15.6 Hz coupling constant found between H-5 and H-6.

Compounds **2** and **3** can be considered derivatives of enigmazole C, and therefore we assumed the absolute configurations at positions C-2, C-5 (just for compound **2**), C-11, C-13, C-17, and C-23 were the same as for **1**; thus (2*S*,5*R,*11*R,*13*R,*17*S,*23*R*) for enigmazole E (**2**) and (2*S,*11*R,*13*R,*17*S,*23*R*) for enigmazole D (**3**) were determined.

All of the new compounds were tested in a panel of four human cancer cell lines: A-549 (lung), HT-29 (colon), MDA-MB-231 (breast), and PSN-1 (pancreas). Enigmazole C (**1**) showed activity on the order of micromolar range with a GI_50_ of 9.9 μM against the A-549 cell line (Table 2). Although enigmazole D (**3**) is the open form with an additional double bond of compound **1**, cytotoxicity was observed in all cell lines (Table 3), while the methyl ester **2** of the open form of **1** renders the compound totally devoid of cytotoxicity.

**Table 1:** NMR Data of Enigmazole C (**1**) in CD_3_CN and Acetone-*d*_6_

	CD_3_CN δ_H_, m (*J* in Hz)		acetone-*d*_6_	
pos.	δ_C_, type	δ_H_, m (*J* in Hz)	δ_C_	δ_H_, m (*J* in Hz)
1	175.6, C		174.9	
2	39.9, CH	2.49, m	39.7	2.53 ddq (10.6, 6.9, 3.4)
3	30.8, CH_2_	a: 1.69, m	30.8	a: 1.79, dddd (13.8, 9.9, 5.4, 3.4)
		b: 1.63, m		b: 1.68, dddd (13.8, 10.6, 5.3, 5.3)
4	34.5, CH_2_	a: 1.43, m	34.5	a: 1.51, dddd (14.4, 9.9, 5.3, 2.2)
		b: 1.31, m		b: 1.38, dddd (14.4, 10.4, 5.4, 5.3)
5	69.5, CH	3.74, ddddd (10.4, 7.6, 5.5, 5.5, 2.5)	69.6	3.83, ddddd (10.4, 8.1, 5.8, 4.5, 2.2) 2.2)
6	44.6, CH_2_	a: 2.49, dd (13.2, 5.5)	44.9	a: 2.59, dd (13.1, 4.5)
		b: 2.27, dd (13.2, 7.6)		b: 2.31, dd (13.1, 8.1)
7	175.5, C		174.8	
8	106.8, CH	5.30, d (1.0)	106.8	5.28, bs (1.0)
9	193.6, C		192.4	
10	42.2, CH_2_	a: 2.41, dd (16.7, 14.0)	42.3	a: 2.41, dd (16.7, 14.0)
		b: 2.23, dd (16.7, 2.6)		b: 2.26, dd (16.7, 2.7)
11	77.8, CH	4.48, ddt (14.0, 11.0, 2.6, 2.6)	77.4	4.53 ddt (14.0, 11.0, 2.7, 2.7)
12	42.9, CH_2_	a: 1.81, ddd (14.2, 10.9, 2.6)	43.1	a: 1.85, ddd (14.1, 11.0, 2.7)
		b: 1.46, dd (14.2, 11.0)		b: 1.54, ddd (14.2, 11.1, 2.3)
13	25.8, CH	2.20, m	25.6	2.29, m
14	40.2, CH_2_	a: 2.49, dd (15.8, 1.5)	40.0	a: 2.27, dd (16.0, 2.7)
		b: 1.59, dd (15.8, 10.7)		b: 1.61, dd (16.0, 11.0)
15	143.9, C		143.8	
16	42.2, CH_2_	a: 2.72, dd (13.7, 9.6)	42.3	a: 2.75, dd (13.8, 9.7)
		b: 2.62, dd (13.7, 5.2)		b: 2.67, dd (13.8, 5.1)
17	66.7, CH	5.92, ddd (9.4, 5.2, 0.8)	66.3	6.00, dd (9.7, 4.9)
18	141.7, C		142.0	
19	135.9, CH	7.63, d (0.7)	135.6	7.77, s
20	161.2, C		161.0	
21	113.6, CH	6.20, dd (1.5, 0.8)	113.5	6.21, bs
22	152.7, C		152.4	
23	75.5, CH	5.20, dq (6.5, 0.8)	75.3	5.29, bq (6.5)
24	19.5, CH_3_	1.23, d (6.5)	19.4	1.24, d (6.5)
25	17.7, CH_3_	1.86, d (1.5)	17.5	1.88, d (1.6)
26	17.6, CH_3_	1.10, d (6.9)	17.6	1.11, d (6.9)
27	21.2, CH_3_	0.95, d (6.7)	21.2	0.98, d (6.6)
28	113.9, CH_2_	a: 4.91, s	113.5	a: 4.94, s
		b: 4.86, s		b: 4.88, s
29	56.6, CH_3_	3.15, s	56.5	3.17, s
OH	-	3.00, d (6.0)	-	3.90 d (5.8)

**Table 2:** Cytotoxic Activities for Enigmazole C (**1**)

cell line	GI_50_	TGI	LC_50_
A-549	9.9 μM	>19 μM	>19 μM
HT-29	18 μM	>19 μM	>19 μM
MDA-MB-231	>19 μM	>19 μM	>19 μM
PSN-1	87 μM	>19 μM	>19 μM

**Table 3:** Cytotoxic Activities for Enigmazole D (**3**)

cell line	GI_50_	TGI	LC_50_
A-549	1.4 μM	>19 μM	>19 μM
HT-29	1.0 μM	3.7 μM	>19 μM
MDA-MB-231	4.1 μM	>19 μM	>19 μM
PSN-1	1.1 μM	>19 μM	>19 μM

In summary, the investigation of the *Homophymia* sp. sponge led to the isolation of one new macrolide lactone, enigmazole C (**1**), along with two open form congeners, enigmazoles E (enigmazole C seco acid methyl ester) (**2**) and enigmazole D (**3**). Spectroscopic data, chemical derivatization, and NMR-DFT methods were necessary to deduce the configurations of the six stereogenic centers present in their carbon skeletons. Notably, compounds **1** and **3** exhibited cytotoxic activities against A-549 (lung), HT-29 (colon), MDA-MB-231 (breast), and PSN-1 (pancreas) cell lines.

## Experimental Section

### General Experimental Procedures

Optical rotations were measured on a JASCO DIP-1000 polarimeter, with a Na (589 nm) lamp and filter. UV spectra were measured on a JASCO V-650 spectrophotometer. IR spectra were measured on a FTIR Bruker Vector 22 spectrometer. ^1^H, ^13^C, and 2D NMR spectra were recorded on a Varian “Unity 500” (500 MHz for ^1^H and 125 MHz for ^13^C), Varian “Unity 400” (400 MHz for ^1^H and 100 MHz for ^13^C), and Bruker Avance 500 (500 MHz for ^1^H and 125 MHz for ^13^C) with a dual cryoprobe or a BBI probe. CD_3_OD, CD_3_CN, C_5_D_5_N, and acetone-*d*_6_ were used as deuterated solvents. Chemical shifts are reported in δ scale relative to methanol-*d*_4_ (δ 3.31 ppm for ^1^H NMR, δ 49.0 ppm for ^13^C NMR), acetonitrile-*d*_3_ (δ 1.94 ppm for ^1^H NMR, δ 1.32 ppm for ^13^C NMR), acetone-*d*_6_ (δ 2.05 ppm for ^1^H NMR, δ 29.84 ppm for ^13^C NMR), and pyridine-*d*_5_ (δ 8.74 ppm for ^1^H NMR, δ 150.35 ppm for ^13^C NMR). HSQC-TOCSY-HECADE experiments were acquired with 32 scans and 256 increments with a mixing time of 60 ms and 4K points. J-HMBC was run with 16 scans, 200 increments, and 2 K points in F2.

LRESIMS and HRESIMS experiments were performed on the Applied Biosystems QSTAR Elite system or on an Agilent 6230 TOF LC/MS. HPLC separation was performed on an Agilent 1100 or 1200 using reversed-phase chromatographic columns.

### Animal Material

The sponge belongs to the genus *Homophymia* Vacelet and Vasseur, 1971. It was collected by hand using a rebreather diving system in Gorontalo, Indonesia (01° 19.836′ S/122° 45.022′ E) at depths ranging between 40 and 80 m. The animal material was identified by Dr. María Jesús Uriz (Center for Avanced Studies of Blanes). A sample of the specimen was deposited in the Center for Advanced Studies of Blanes in Girona, Spain, with the reference code GORO-093. In addition, a voucher specimen (ORMA136284) was deposited at PharmaMar.

Description: Thickly globular sponge (3 cm high, 4 cm wide, 2 cm tick), smooth without conulous projections. Hard consistency, white color, and small single oscule in apical position. Megascleres: fine monoactines spicules broken in their preparations. Desmas pseudotetraclones with very tuberculated zygomes. Ectosomal megascleras are smooth pesudophyllotriaenes with different irregular pseudocladis tuberculated in the same specimen. Microscleres consist of a single type of microspinose amphiasters occurring in different size classes: the smaller 10–15 μm long, the larger 12–25 μm long.

### Extraction and Isolation

A specimen of *Homophymia* (33 g) was triturated and exhaustively extracted with MeOH/CH_2_Cl_2_ (50:50, 3 × 500 mL). The combined extracts were concentrated to yield a mass of 2.5 g, which was subjected to VLC on Lichroprep RP-18 (Merck KGaA) with a stepped gradient from H_2_O to MeOH and then CH_2_Cl_2_. The fraction eluting with MeOH (96 mg) was subjected to semipreparative HPLC (Ascentis C18, 5 μm, 410 × 150 mm, isocratic H_2_O/MeCN (60:40) for 3 min, gradient H_2_O/MeCN from 50% to 65% MeCN in 25 min, UV detection, flow 1 mL/min, *t*_r_: 19.5 min to give compound **1** (2.9 mg), 12.0 min to give compound **2** (1.4 mg), and 13.0 min to give compound **3** (0.4 mg)).

#### Enigmazole C (**1**):

amorphous, yellow oil; [α]_D_ +56 (*c* 0.03, MeOH); UV (MeOH) λ_max_ 261 nm; IR (ATR) ν_max_ 3378, 2966, 2311, 1675, 1140 cm^–1^; ^1^H NMR (500 MHz) and ^13^C NMR (125 MHz), Table 1; (+)-HRESI-TOFMS *m*/*z* 516.2964 [M + H]^+^ (calcd for C_29_H_42_NO_7_, 516.2956); *m/z* 538.2785, [M + Na]^+^ (calcd for C_29_H_41_NO_7_Na).

#### Enigmazole E (enigmazole C seco acid methyl ester, **2**):

yellow oil; UV (MeOH) λ_max_ 265 nm; ^1^H and ^13^C NMR, Table S1; (+)-HRESI-TOFMS *m*/*z* 548.3249 [M + H]^+^ (calcd for C_30_H_46_NO_8_, 548.3218).

#### Enigmazole D (**3**):

yellow oil; UV (MeOH) λ_max_ 254, 277, 286 nm; ^1^H and ^13^C NMR, Table S2; (+)-HRESI-TOFMS *m*/*z* 516.2932 [M + H]^+^ (calcd for C_29_H_42_NO_7_, 516.2956).

### Computational Calculations

Conformational searches were performed by using the corresponding module implemented in the Maestro Quantum mechanical software. The MMFF force field with acetonitrile as solvent was used, and torsional enhanced sampling with 10 000 steps was fixed using an energy window of 5 kcal/mol.

#### DP4+

Molecular geometry optimizations were performed for **1a**–**d** at the DFT theoretical level using the Gaussian 16W package first at the B3LYP/6-31G(d) level for energy and frequency calculations. After removing redundant conformers and those with imaginary frequencies, theoretical Boltzmann energy population-weighted ^1^H and ^13^C NMR were calculated by using the combination MPW1PW91/6-31G(d,p). Results were input in Sarotti’s and Darana’s Excel spreadsheet^27^ to calculate the best fit.

#### i*J*-DP4

MMFF conformers at 5 kcal/mol were filtered by six proton–proton vicinal coupling restriction: ^3^*J*_H11H12a_, ^3^*J*_H11H12b_, ^3^*J*_H5H4b_, ^3^*J*_H5H4a_, ^3^*J*_H2H3b_, and ^3^*J*_H2H3a_. Those conformers were chosen to calculate the population-weighted Boltzmann MMFF energy. ^1^H and ^13^C NMR and ^1^H–^1^H coupling constants (just the Fermi’s contact contribution) were then calculated by the combination B3LYP/6-31G(d). Results were input in Sarotti’s and Darana’s Excel spreadsheet^27^ to calculate the best fit.

### Preparation of the (*S*)-MTPA Ester of Enigmazole C (**1-*S***)

*R*-(−)-MTPA chloride (10 μL) was added to a solution of **1** (1.0 mg) in 0.5 mL of pyridine-*d*_5_ in an NMR tube. The resulting mixture was allowed to stand at room temperature (rt) for 8 h to yield the (*R*)-MTPA ester of **1**, which was monitored by recording ^1^H NMR spectra at 500 MHz. ^1^H NMR (500 MHz, C_5_D_5_N) δ 5.66 (s, H-8), 5.56 (m, H-5), 2.83 (dd, *J* = 13.7, 5.1 Hz, H-6a), 2.71 (m, H2), 2.64 (dd, *J* = 13.7, 7.2 Hz, H-6b), 1.79 (m, H-4), 1.66 (m, H-3), 1.15 (d, *J* = 6.8 Hz, H-26).

### Preparation of the (*R*)-MTPA Ester of Enigmazole C (**1-*****R***)

Treatment of **1** (1.0 mg) in the same manner as before with *S*-(+)-MTPA chloride (10 μL) gave the (*R*)-MTPA ester of **1**. ^1^H NMR (500 MHz, C_5_D_5_N) δ 5.71 (s, H-8), 5.57 (m, H-5), 2.90 (dd, *J* = 13.8, 5.0 Hz, H-6a), 2.59 (m, H-2), 2.74 (dd, *J* = 13.8, 7.0 Hz, H-6b), 1.71 (m, H-4), 1.42 (m, H-3), 1.07 (d, *J* = 6.8 Hz, H-26).

### Preparation of the (*S*)-MTPA Ester of Enigmazole E (**2-*****S***)

*R*-(−)-MTPA chloride (10 μL) was added to a solution of **2** (0.4 mg) in 0.5 mL of pyridine-*d*_5_ in an NMR tube. The resulting mixture was allowed to stand at rt for 8 h to yield the (*S*)-MTPA ester of **2**, which was monitored by recording ^1^H NMR spectra at 500 MHz. ^1^H NMR (500 MHz, C_5_D_5_N) δ 6.60 (dd, *J* = 7.2, 7.2 Hz, H-17), 5.69 (m, H-5), 5.67 (s, H-21), 5.45 (d, *J* = 6.4 Hz, H-23), 4.92 (s, H-28), 2.93 (m, H-16), 2.68 (m, H-6), 2.51 (m, H-2), 2.18 (m, H-14), 1.87 (s, H-25), 1.86 (m, H-3), 1.86 (m, H-4), 1.12 (d, 7.0 Hz, H-26), 0.91 (d, 5.9 Hz, H-27).

### Preparation of the (*R*)-MTPA Ester of Enigmazole E (**2-*****R***)

Treatment of **2** (0.4 mg) in the same manner as before with *S*-(+)-MTPA chloride (5 μL) gave the (*R*)-MTPA ester of **2**. ^1^H NMR (500 MHz, C_5_D_5_N) δ 6.62 (dd, *J* = 7.1, 7.1 Hz, H-17), 5.70 (m, H-5), 5.66 (s, H-21), 5.40 (d, *J* = 6.3 Hz, H-23), 5.04 (s, H-28), 2.99 (m, H-16), 2.73 (m, H-6), 2.41 (m, H-2), 2.24 (m, H-14), 1.85 (s, H-25), 1.73 (m, H-3), 1.73 (m, H-4), 1.04 (d, 7.0 Hz, H-26), 0.93 (d, 5.9 Hz, H-27).

### Biological Assays

The cytotoxic activities of compounds **1**, **2**, and **3** were tested against A-549 human lung carcinoma cells, MDA-MB-231 human breast adenocarcinoma cells, HT-29 human colorectal carcinoma cells, and PSN-1 pancreatic adenocarcinoma cells. The concentration giving 50% inhibition of cell growth (GI_50_) was calculated according to the procedure described in the literature. Cell survival was estimated using the National Cancer Institute (NCI) algorithm. Three dose–response parameters were calculated for **1**, **2**, and **3**.

## Supporting Information Available

^1^H, ^13^C, and 2D NMR data of the new compounds and *J* configurational analysis of **1** (PDF)
