# Supplementary material for: Enigmazole C: A Cytotoxic Macrocyclic Lactone and Its Ring-Opened Derivatives from a New Species of Homophymia Sponge
Source: J Nat Prod. 2022 Mar 2;85(4):1059–66. doi: 10.1021/acs.jnatprod.1c01179 (PMC9040057; doi:10.1021/acs.jnatprod.1c01179)
Supplement: Supplementary file 1 — np1c01179_si_001.pdf [file np1c01179_si_001.pdf]

SUPPLEMENTARY MATERIAL

Enigmazole C: A Cytotoxic Macrocyclic Lactone and its Ring-Opened Derivatives from a New Species of *Homophymia* Sponge

Guillermo Tarazona [a], Rogelio Fernández [a], Marta Pérez[a],

Ramón E. Millán [b] Carlos Jiménez[b], Jaime Rodríguez*[b], and Carmen Cuevas[a]*

[a] R&D,PharmaMar, Avda. De los Reyes, 1, Pol. Ind. La Mina-Norte, 28770-Colmenar Viejo, Madrid, Spain

[b] Departmento de Química, Facultad de Ciencias and Centro de Investigacions Científicas Avanzadas (CICA) Universidade de A Coruña, 15071 A Coruña, Spain.

E-mail: carlos.jimenez@udc.es; jaime.rodriguez@udc.es

Figure S1. ^{1}H NMR spectrum (500 MHz, CD_3CN) of enigmazole C (**1**).. S4
Figure S2. ^{13}C NMR spectrum (125 MHz, CD_3CN) of enigmazole C (**1**)....................................... S5
Figure S3. DEPT135 Edited-HSQC spectrum (500 MHz, CD_3CN) of enigmazole C (1). S6
Figure S4. ^{1}H-^{1}H COSY spectrum (500 MHz, CD_3CN) of enigmazole C (**1**). S7
Figure S5. HMBC spectrum (500 MHz, CD_3CN) of enigmazole C (**1**). S8
Figure S6. ^{1}H-^{1}H TOSCY spectrum (500 MHz, CD_3CN) of enigmazole C (**1**). S9
Figure S7. ^{1}H-^{1}H ROESY spectrum (500 MHz, CD_3CN) of enigmazole C (**1**). S10
Figure S8. ^{1}H-NMR spectrum (500 MHz, C_5D_5N) of enigmazole C-R-MPTA (**1**-R)...................... S11
Figure S9. Selective 1D-TOCSY (500 MHz, C_5D_5N) of H5 in enigmazole C –R-MTPA (**1**-*R*) S12
Figure S10. ^{1}H NMR spectrum (500 MHz, C_5D_5N) of enigmazole C-S-MPTA (**1**-*S*).................... S13
Figure S11. Selective 1D-TOCSY (500 MHz, C_5D_5N) of H5 in enigmazole C –S-MTPA (**1-S**) S14
Figure S12. ^{1}H NMR spectrum (500 MHz, acetone-d_6) of enigmazole C (**1**)............................. S15
Figure S13. ^{13}C NMR spectrum (125 MHz, acetone-d_6) of enigmazole C (**1**)............................ S16
Figure S14. HSQC-HECADE spectrum (500 MHz, acetone-d_6) of enigmazole C (**1**).................. S17
Figure S15. J-HMBC spectrum (500 MHz, acetone-d_6) of enigmazole C (**1**) (J-scale =53)......... S18
Figure S16. J-HMBC spectrum (500 MHz, acetone-d_6) of enigmazole C (**1**) (J-scale =55)......... S19
Figure S17. J-HMBC spectrum (500 MHz, acetone-d_6) of enigmazole C (**1**) (J-scale =55)......... S20
Figure S18. J-HMBC spectrum (500 MHz, acetone-d_6) of enigmazole C (**1**) (J-scale =55)......... S21
Figure S19. J-HMBC spectrum (500 MHz, acetone-d_6) of enigmazole C (**1**) (J-scale =55)......... S22
Figure S20. HSQC-HECADE spectrum (500 MHz, acetone-d_6) of enigmazole C (**1**).................. S23
Figure S21. HSQC-HECADE C3alfa and C3beta projections from the HSQC-HECADE (500 MHz, acetone-d_6))of enigmazole C (**1**)... S24
Figure S22. HSQC-HECADE C10alfa and C10beta projections from the HSQC-HECADE (500 MHz, acetone-d_6))of enigmazole C (**1**)... S25
Figure S23. HSQC-HECADE spectrum expansion (500 MHz, acetone-d_6) of enigmazole C (1) . S26
Figure S24. HSQC-HECADE C27alfa and C27beta projections from the HSQC-HECADE (500 MHz, acetone-d6))of enigmazole C (**1**).. S27
Figure S25. ^{1}H NMR spectrum (500 MHz, CD_3OD) of enigmazole C (**1**). S28
Figure S26. ^{13}C NMR spectrum (125 MHz, CD_3OD) of enigmazole C (**1**). S29
Figure S27. ^{1}H-^{13}C Edited-HSQC spectrum (500 MHz, CD_3OD) of enigmazole C (**1**)................. S30
Figure S28. ^{1}H-^{1}H COSY spectrum (500 MHz, CD_3OD) of enigmazole C (**1**)............................. S31
Figure S29. LR-ESIMS (+) of enigmazole C (**1**)... S32
Figure S30. UV (MeOH) spectrum of enigmazole C (**1**)... S33
Figure S31. ^{1}H NMR spectrum (500 MHz, CD_3OD) of enigmazole E (**2**). S34

Figure S32. ^{13}C NMR spectrum (125 MHz, CD_3OD) of enigmazole E (**2**)................................... S35
Figure S33. ^{1}H-^{1}H COSY spectrum (500 MHz, CD_3OD) of enigmazole E (**2**). S36
Figure S34. ^{1}H-^{1}H TOCSY spectrum (500 MHz, CD_3OD) of enigmazole E (**2**). S37
Figure S35. DEPT135-Edited HSQC spectrum (500 MHz, CD_3OD) of enigmazole E **(2**). S38
Figure S36. HMBC spectrum (500 MHz, CD_3OD) of enigmazole E (**2**) S39
Figure S37. UV (MeOH) spectrum of enigmazole E (**2**). S40
Table S1. NMR data in CD_3OD of enigmazole E (**2**) S41
Figure S38. ^{1}H NMR spectrum (500 MHz, CD_3OD) of enigmazole D (**3**). S42
Figure S39. ^{1}H-^{1}H COSY spectrum (500 MHz, CD_3OD) of enigmazole D (**3**).............................. S43
Figure S40. Edited-HSQC spectrum (500 MHz, CD_3CN) of enigmazole D (**3**) S44
Figure S41. UV (MeOH) spectrum of enigmazole D (**3**).. S45
Table S2. NMR data in CD_3CN of enigmazole D (**3**) S46
Table S3. Calculated DFT energies of the **1a-(2S5R11R13R17S23R)** diasteroisomer. S47
Table 4. Calculated DFT energies of the **1b-(2S5R11S13S17S23S)** diasteroisomer................. S76
Table 5. Calculated DFT energies of the **1c-(2S5R11S13S17S23R)** diasteroisomer. S144
Table 6. Calculated DFT energies of the **1d-(2S5R11R13R17S23S)** diasteroisomer. S195

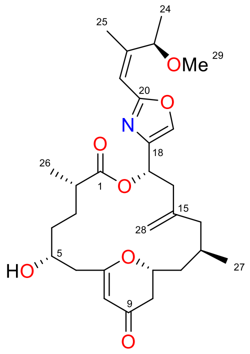

Enigmazole C (**1**)

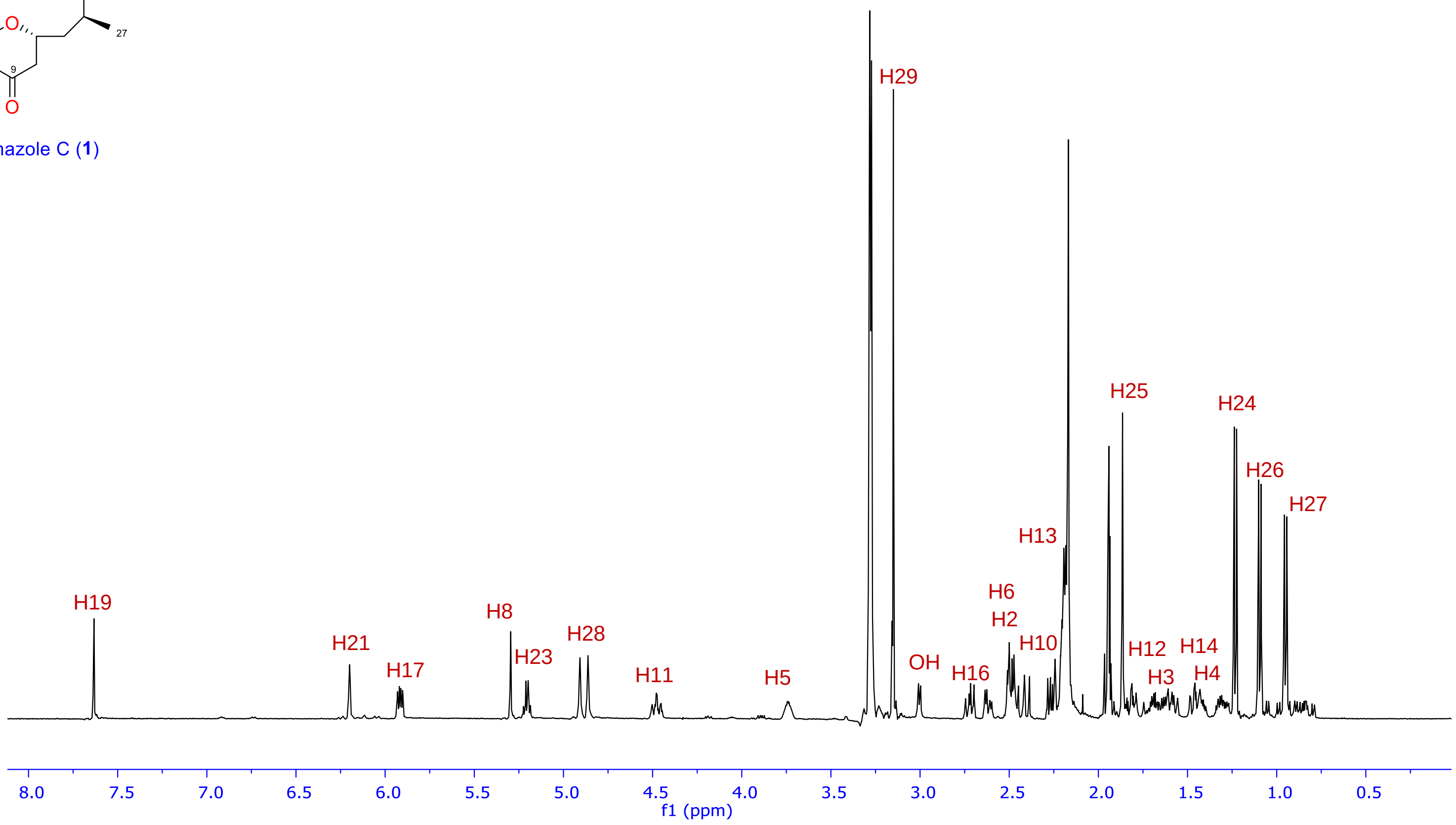

Figure S1. ^{1}H NMR spectrum (500 MHz, CD3CN) of enigmazole C (**1**).

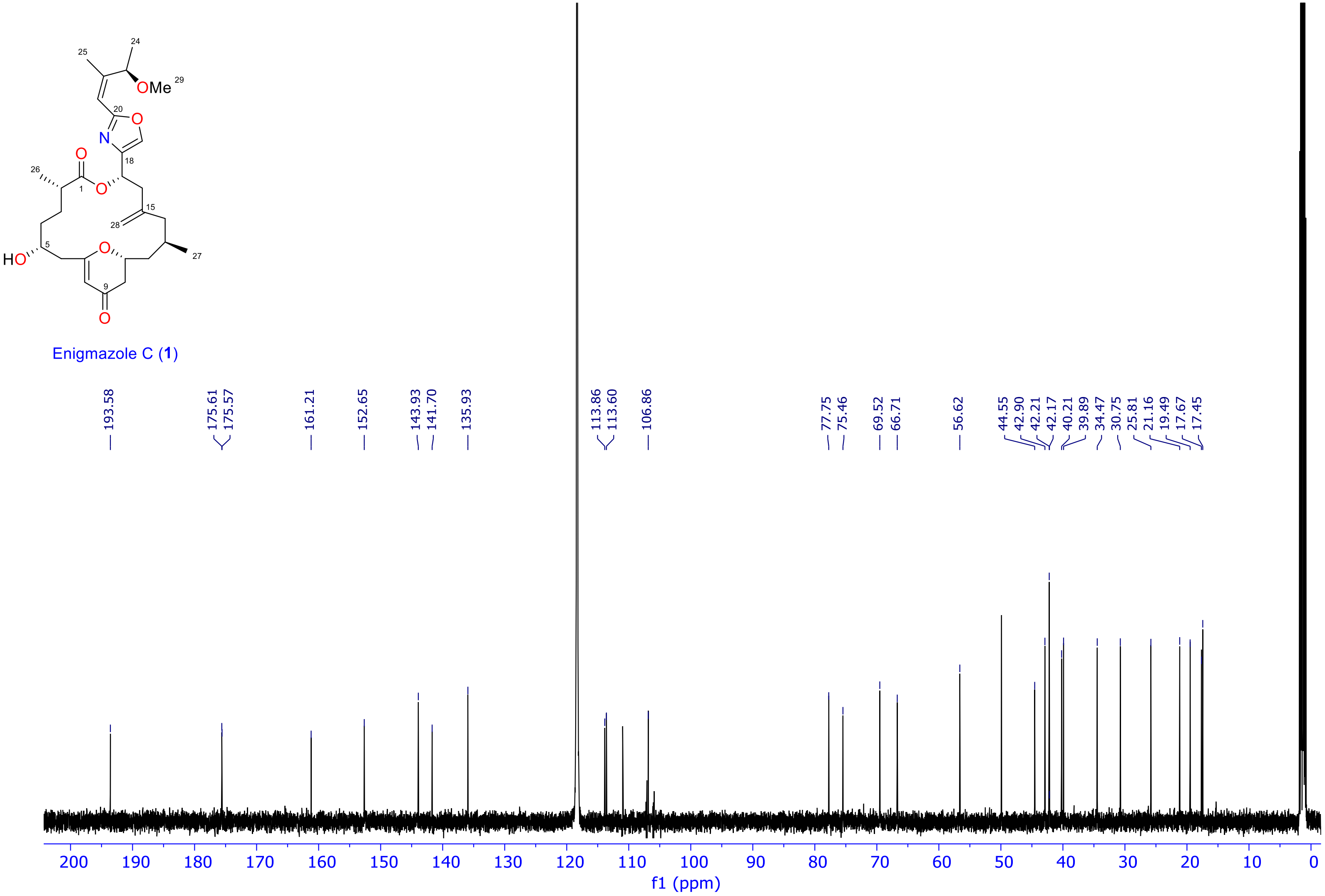

Figure S2. ^{13}C NMR spectrum (125 MHz, CD_3CN) of enigmazole C (**1**).

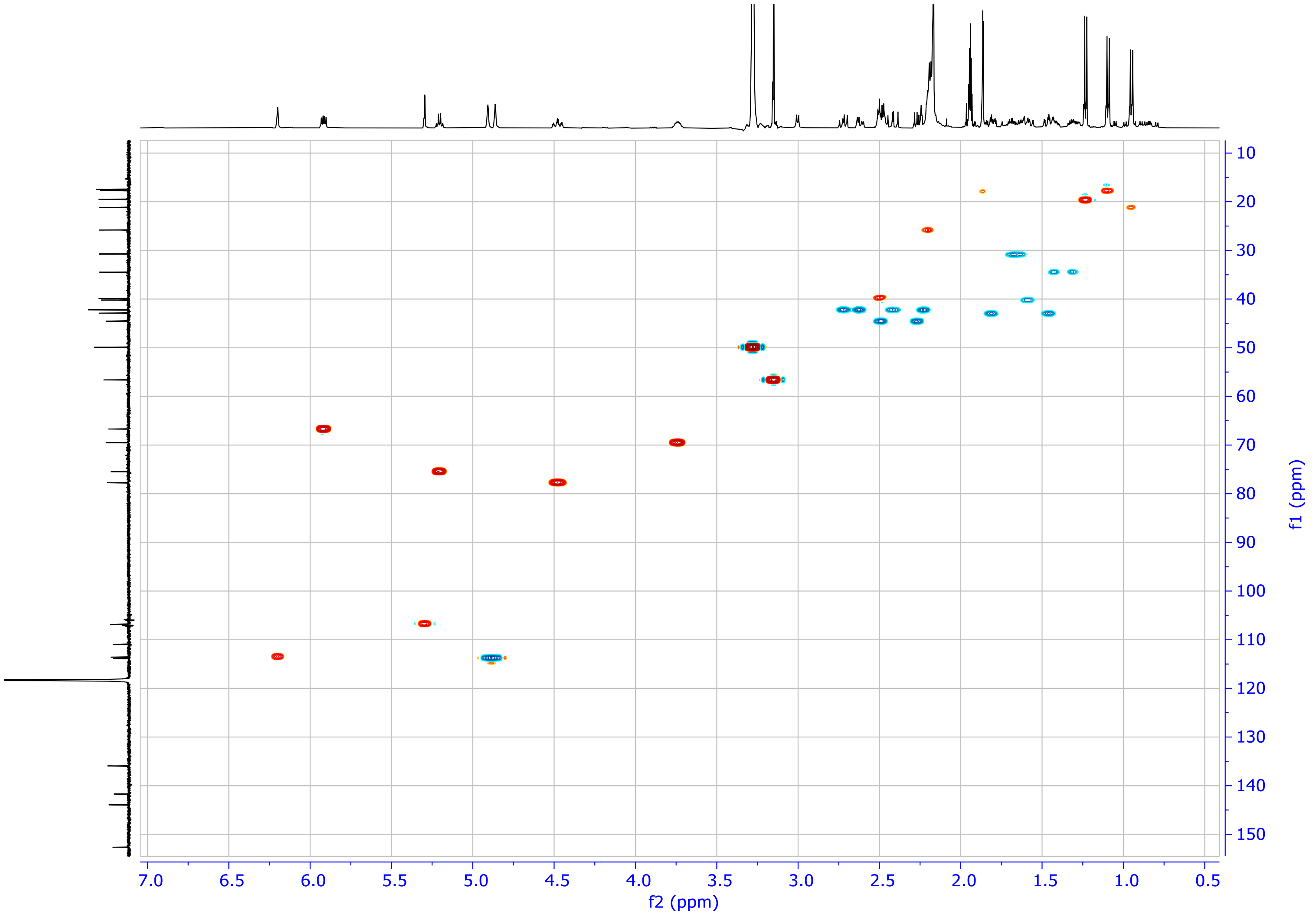

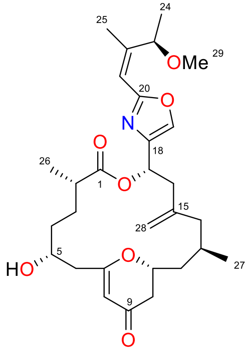

Enigmazole C (**1**)

Figure S3. DEPT135 Edited-HSQC spectrum (500 MHz, CD_3CN) of enigmazole C (**1**).

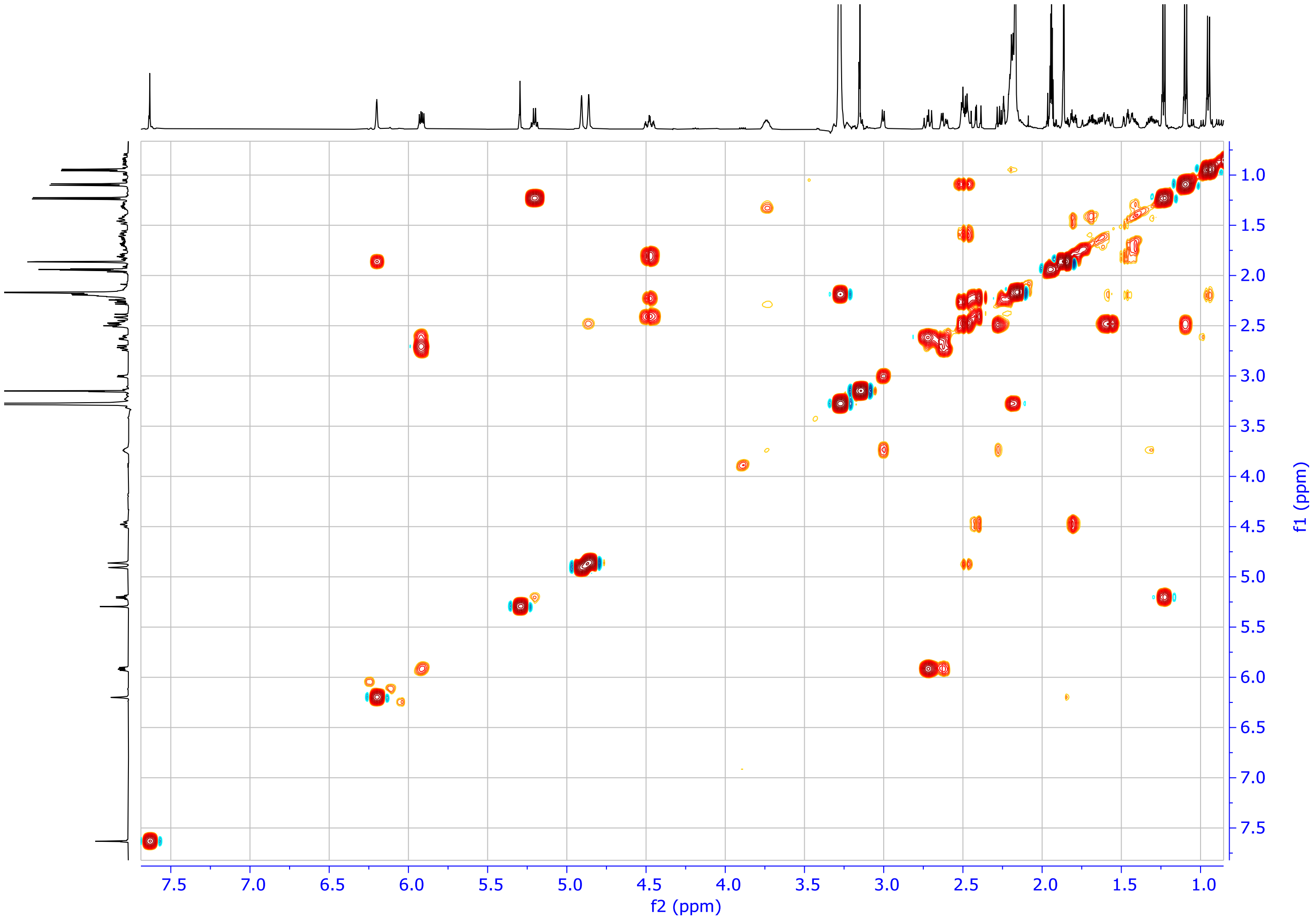

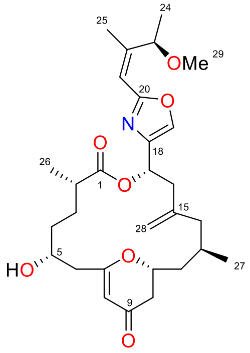

Enigmazole C (1)

Figure S4. ^{1}H-^{1}H COSY spectrum (500 MHz, CD_3CN) of enigmazole C (**1**).

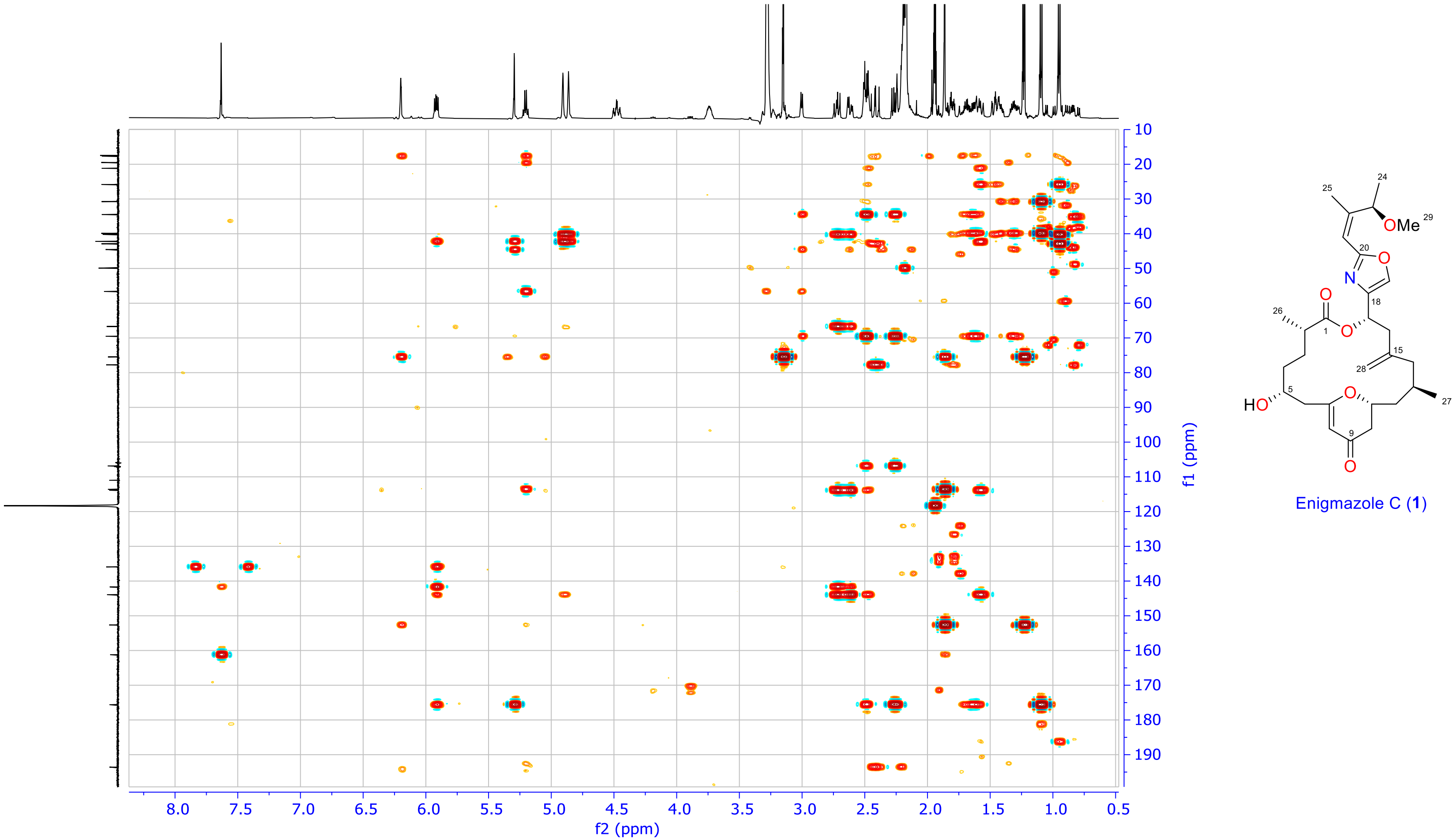

Figure S5. HMBC spectrum (500 MHz, CD_3CN) of enigmazole C (**1**).

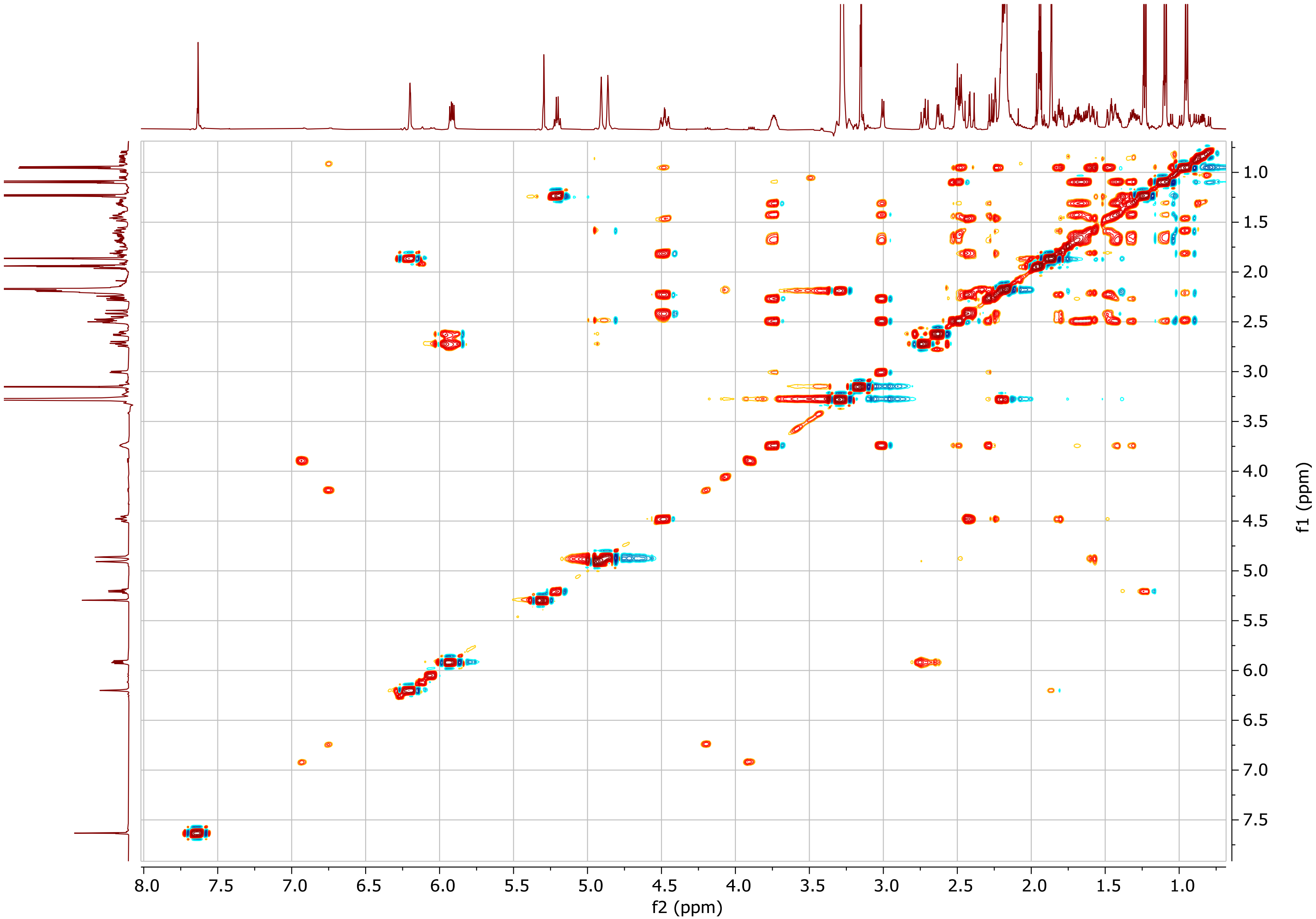

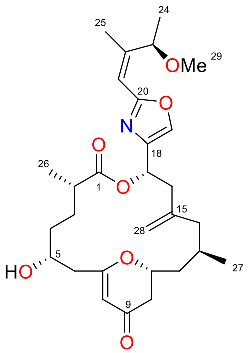

Enigmazole C (**1**)

Figure S6. ^{1}H-^{1}H TOSCY spectrum (500 MHz, CD_3CN) of enigmazole C (**1**).

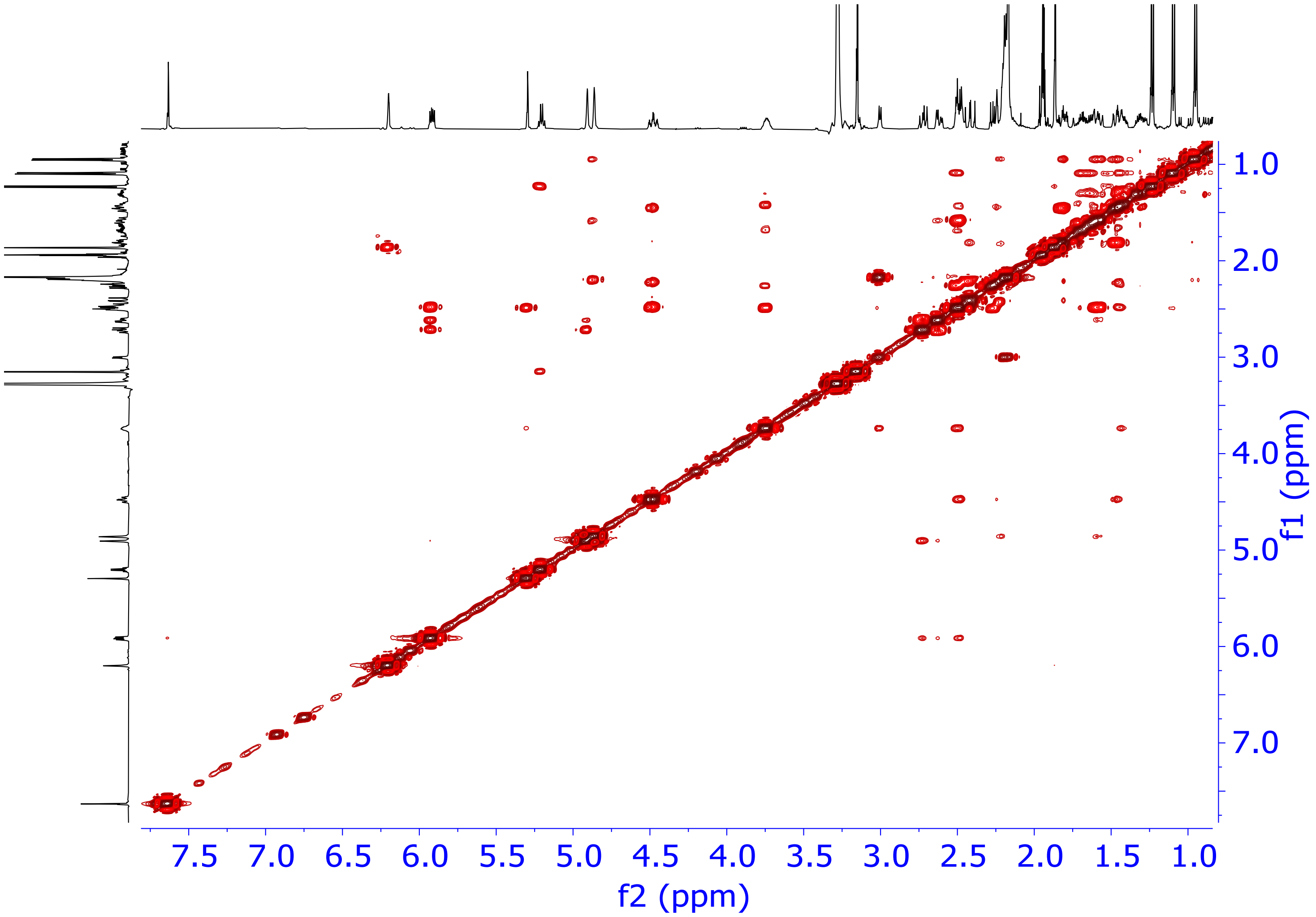

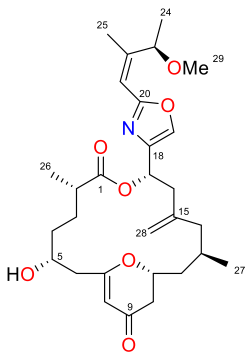

Enigmazole C (**1**)

Figure S7. ^{1}H-^{1}H ROESY spectrum (500 MHz, CD_3CN) of enigmazole C (**1**).

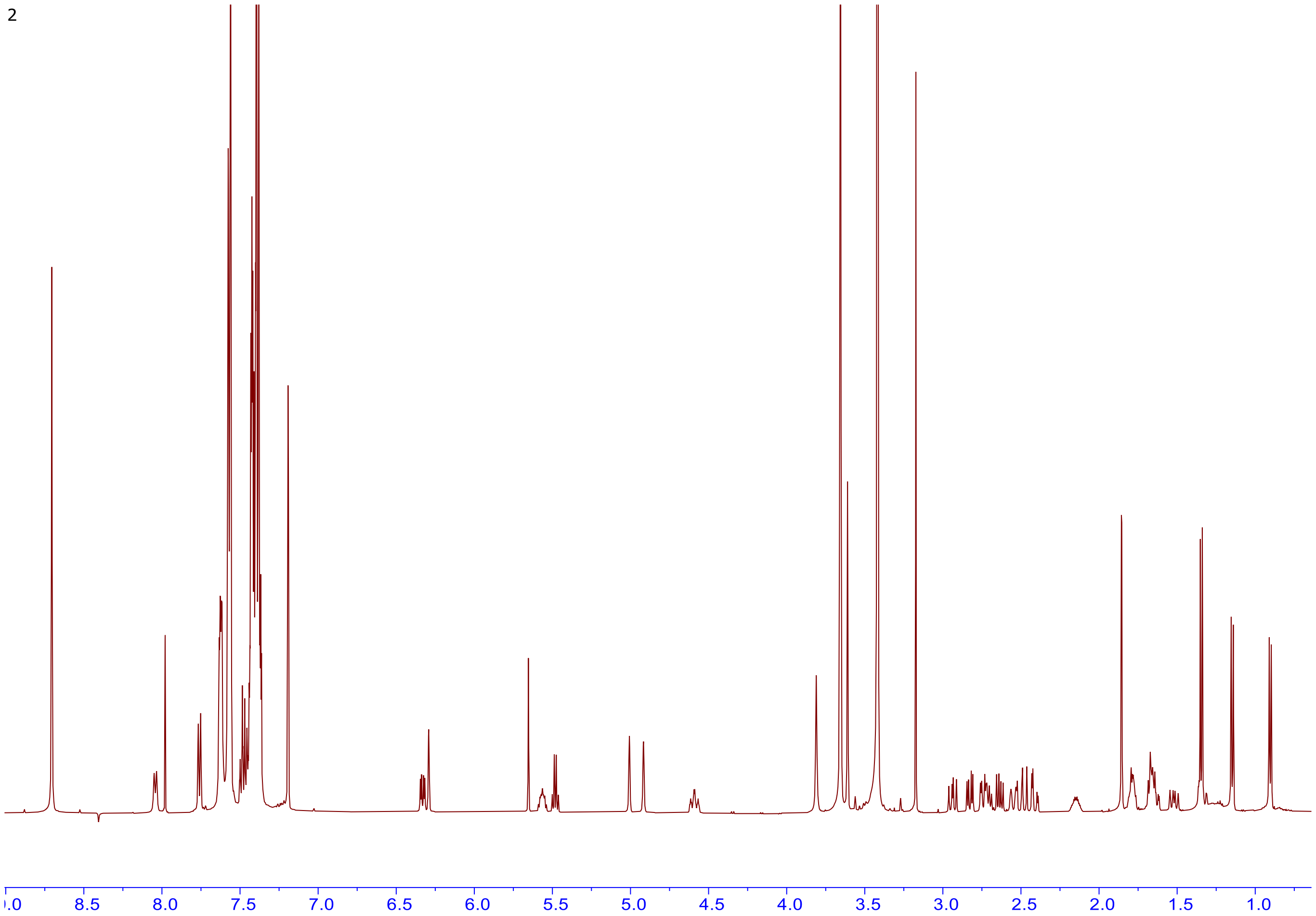

Figure S8. ^{1}H-NMR spectrum (500 MHz, C_5D_5N) of enigmazole C-R-MPTA (**1-R**).

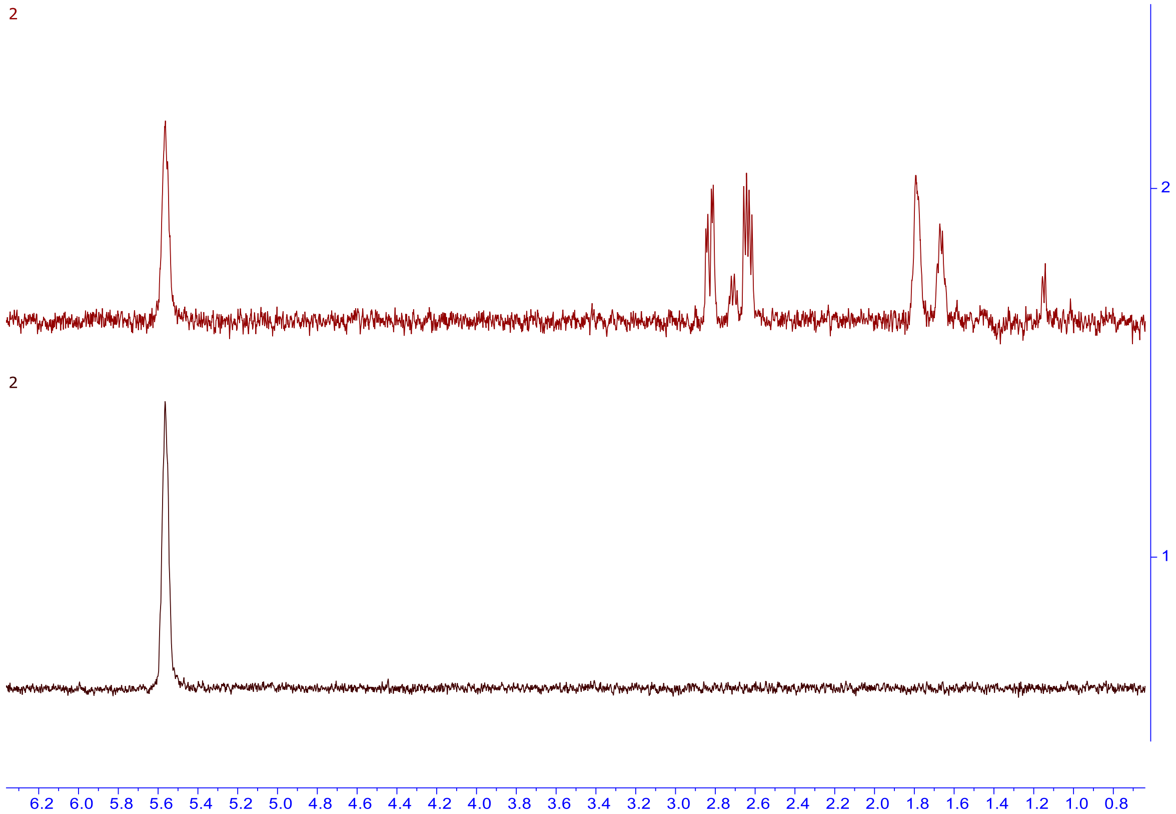

Figure S9. Selective 1D-TOCSY (500 MHz, C_5D_5N) of H5 in enigmazole C –R-MTPA (**1-R**)

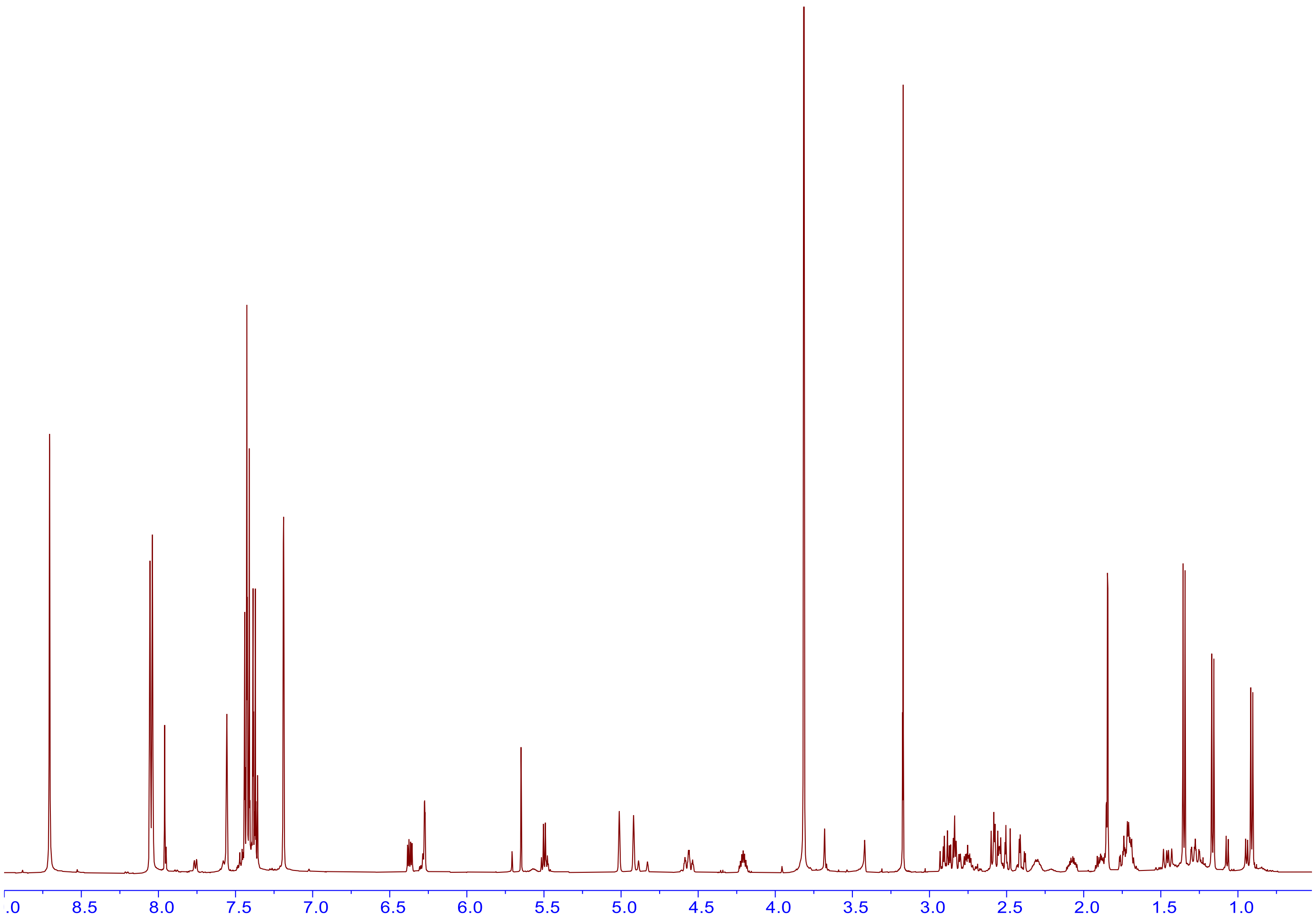

Figure S10. ^{1}H NMR spectrum (500 MHz, C_5D_5N) of enigmazole C-S-MPTA (**1-S**).

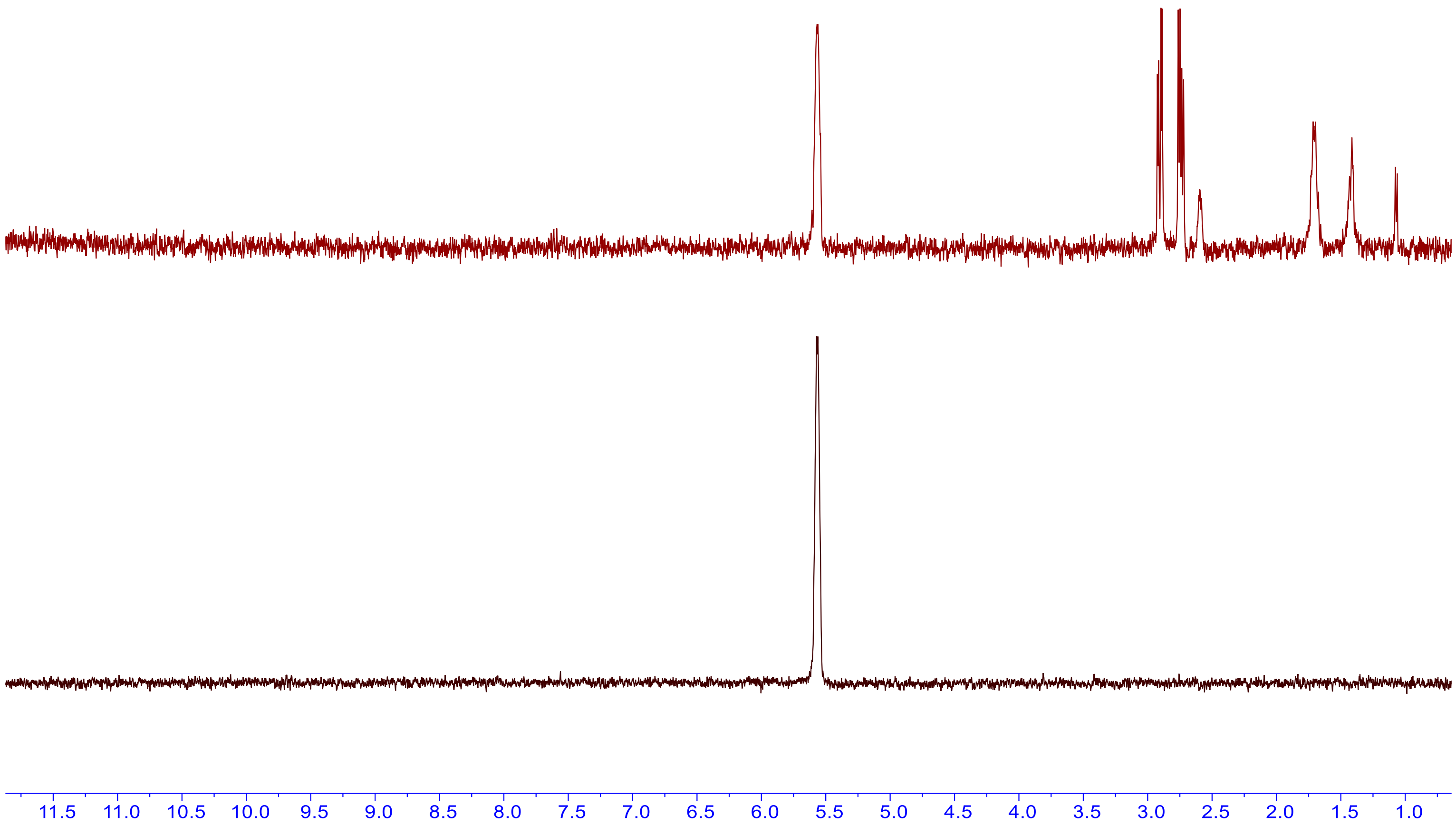

Figure S11. Selective 1D-TOCSY (500 MHz, C_5D_5N) of H5 in enigmazole C –S-MTPA (**1-S**)

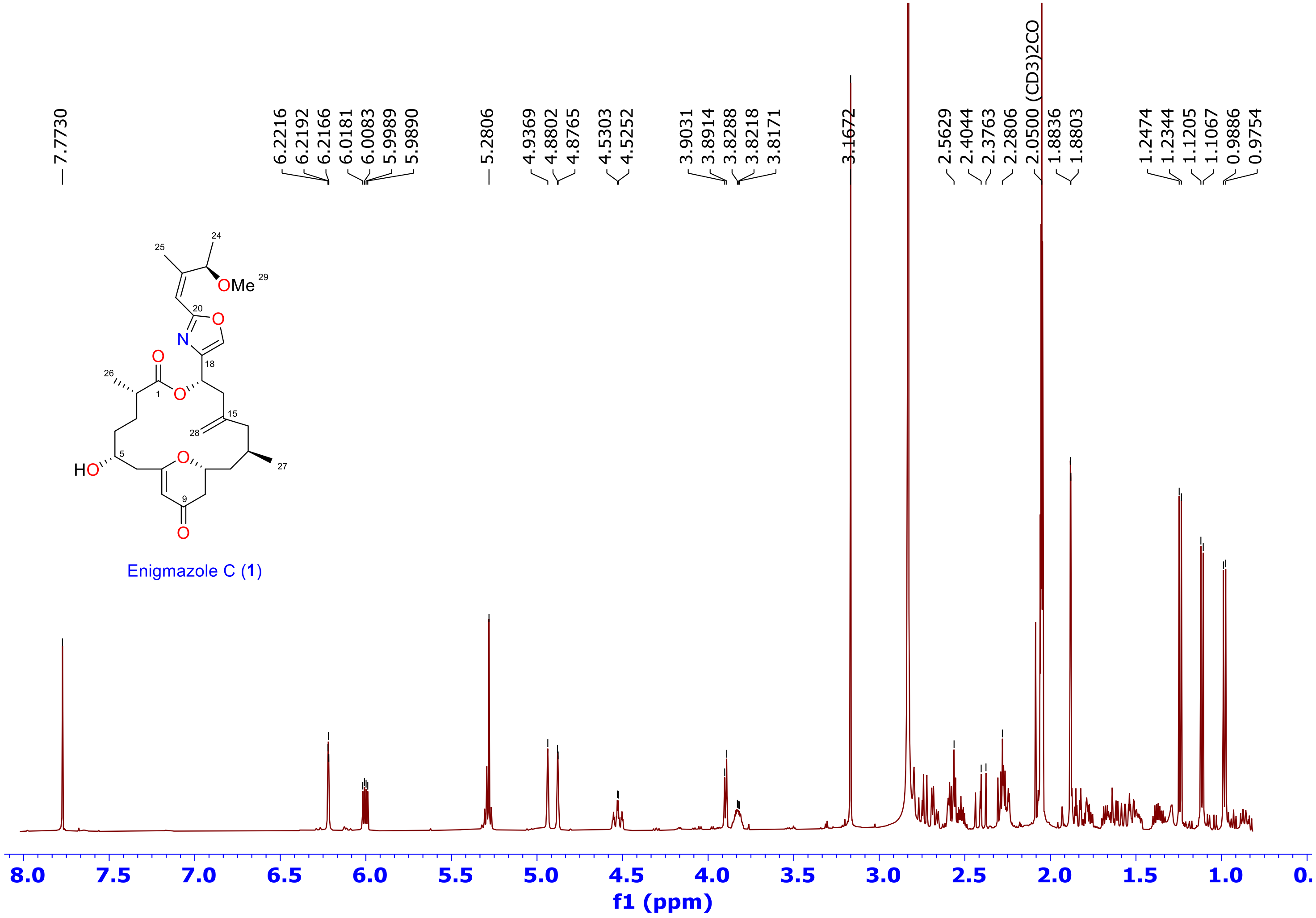

Figure S12. ^{1}H NMR spectrum (500 MHz, acetone-d_6) of enigmazole C (**1**).

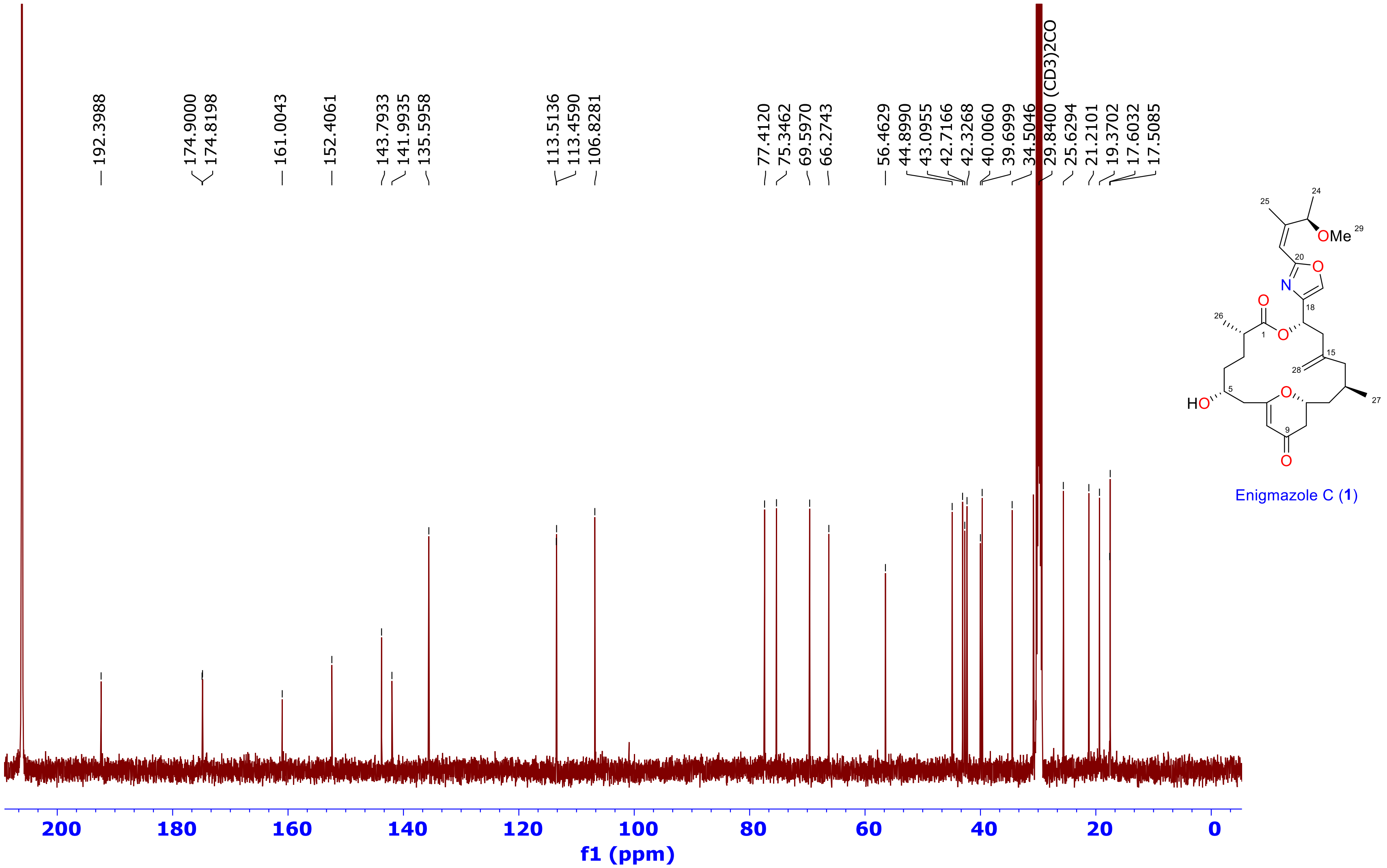

Figure S13. ^{13}C NMR spectrum (125 MHz, acetone-d_6) of enigmazole C (**1**).

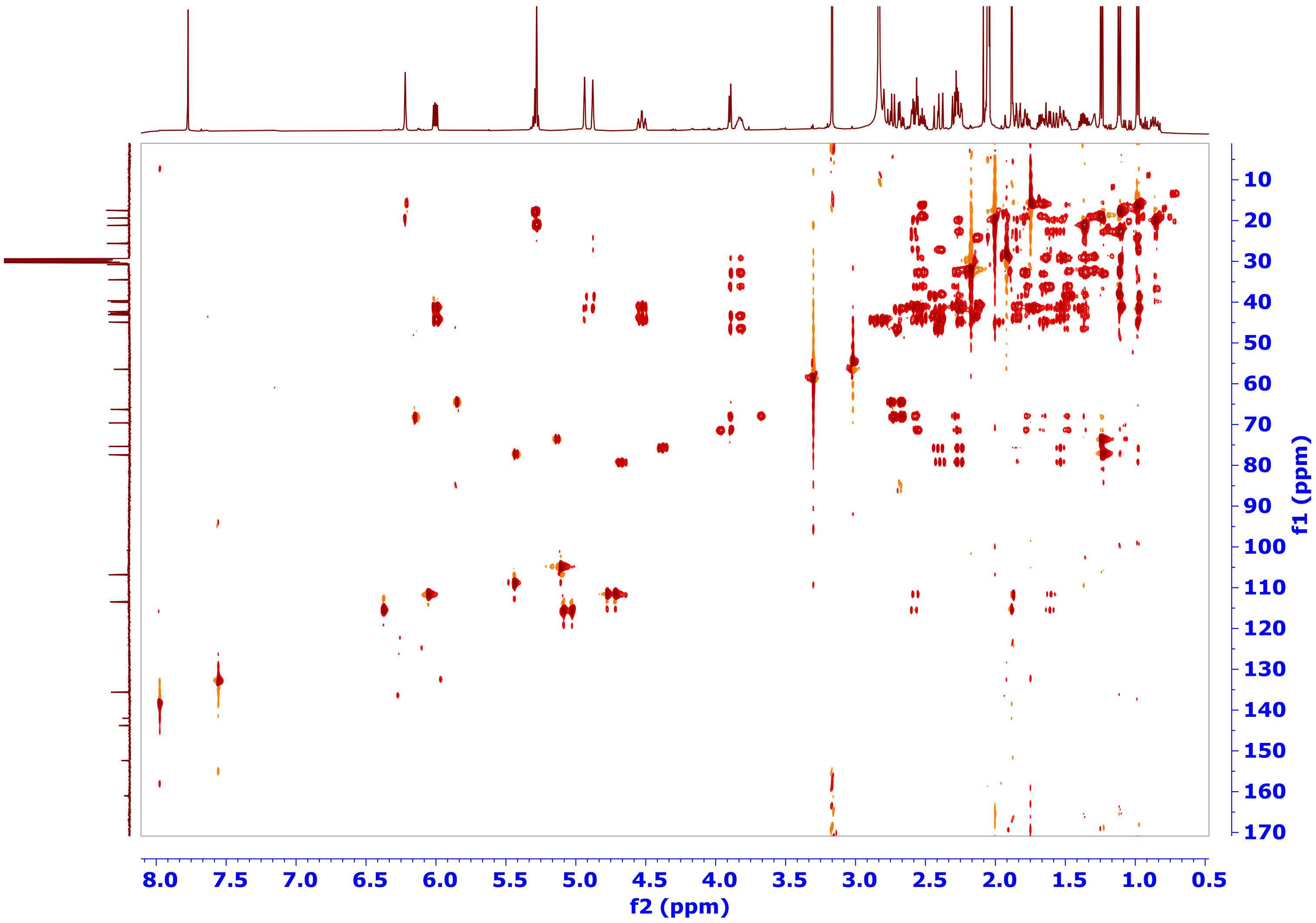

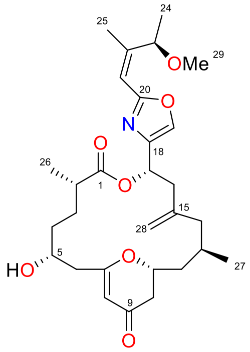

Enigmazole C (1)

Figure S14. HSQC-HECADE spectrum (500 MHz, acetone-d_6) of enigmazole C (**1**).

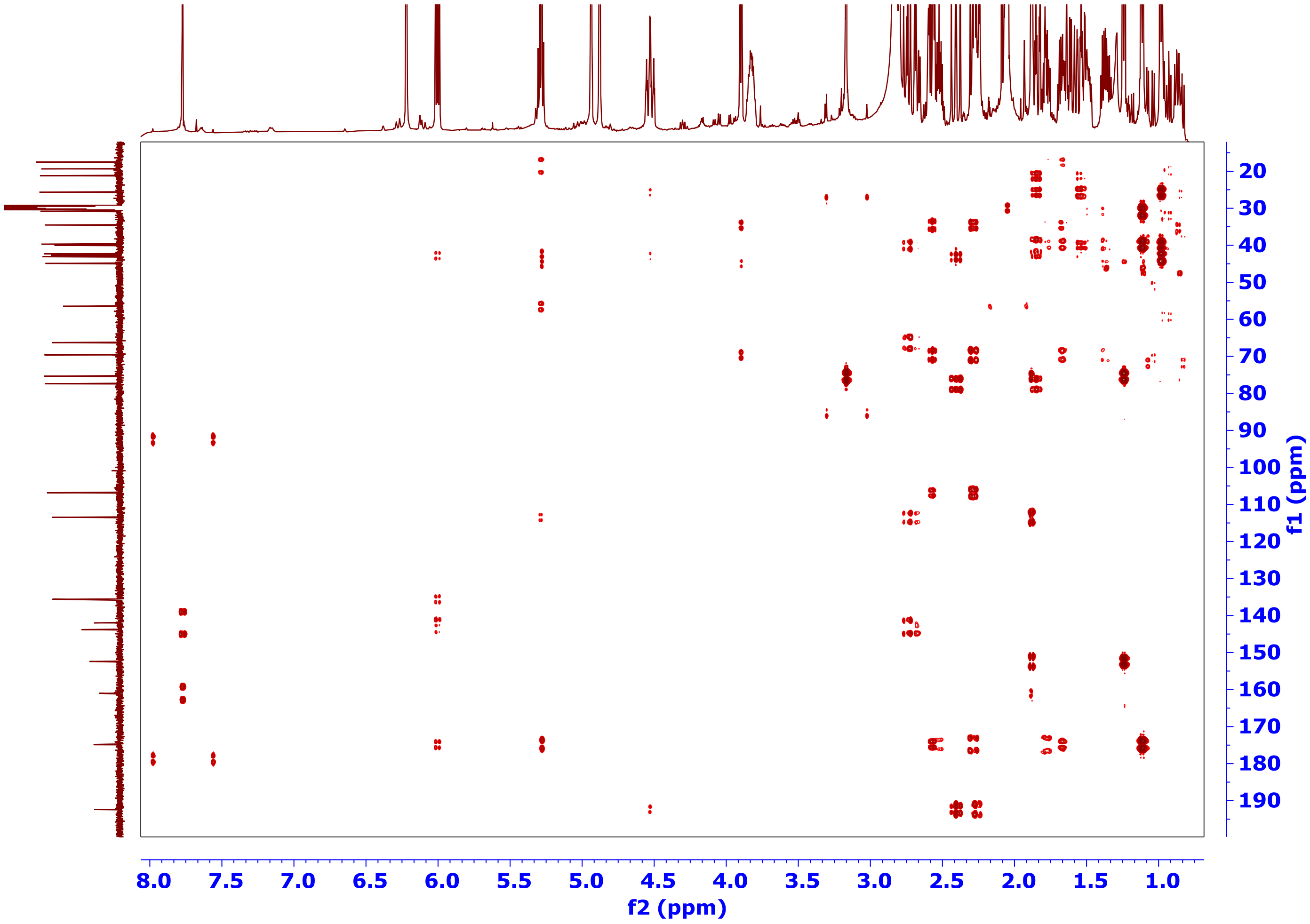

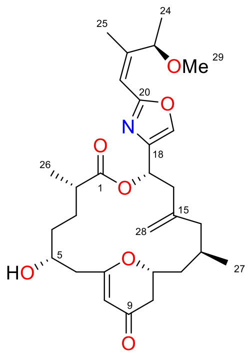

Enigmazole C (1)

Figure S15. J-HMBC spectrum (500 MHz, acetone-d$_6$) of enigmazole C (**1**) (J-scale =53)

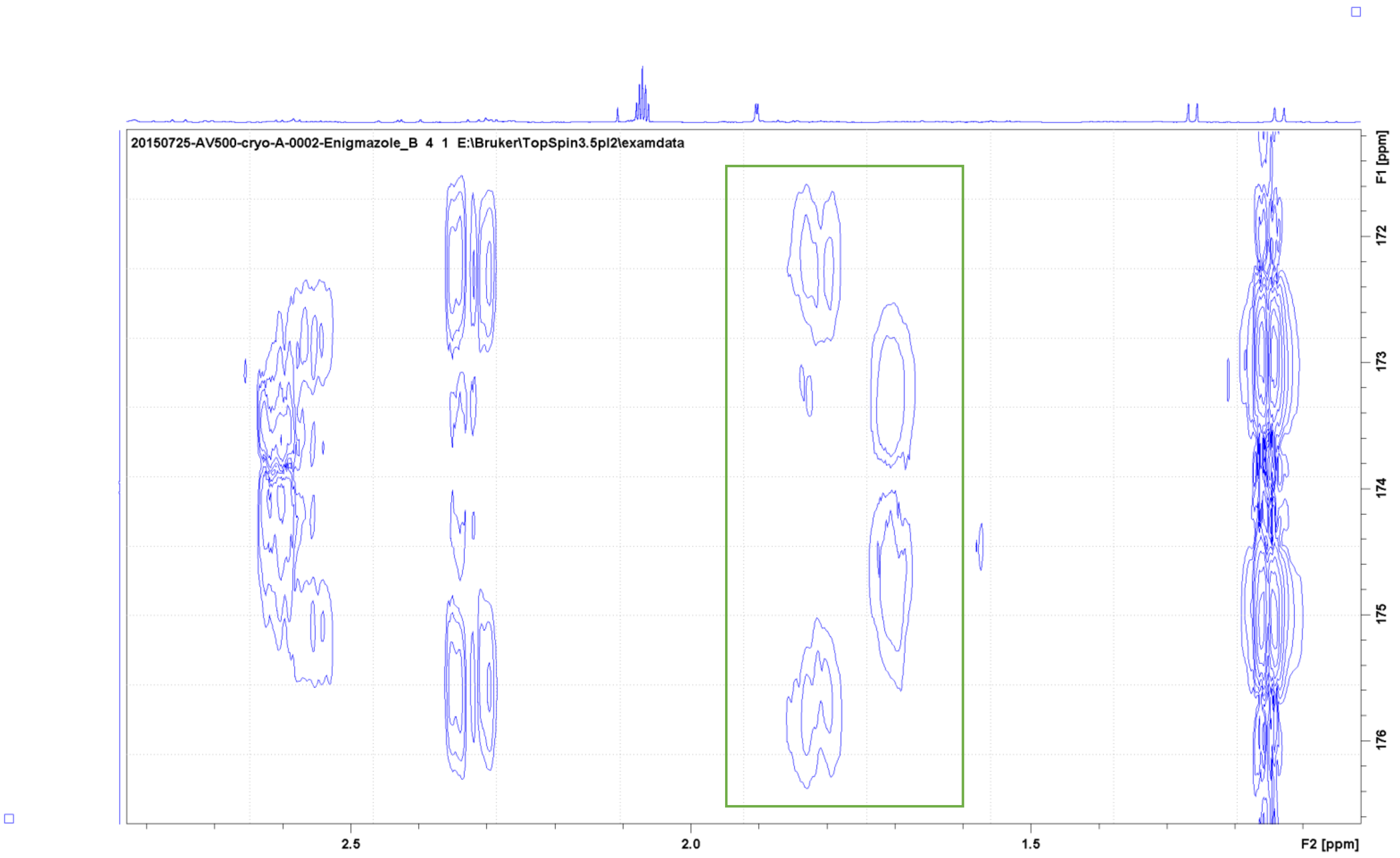

J-scale=53

$^{3}J_{C1H3l}$= 444 /53=8.4 Hz

$^{3}J_{C1H3h}$= 188/53= 3.5 Hz

Figure S16. J-HMBC spectrum expansion (500 MHz, acetone-d_6) of enigmazole C (**1**) (J-scale =55)

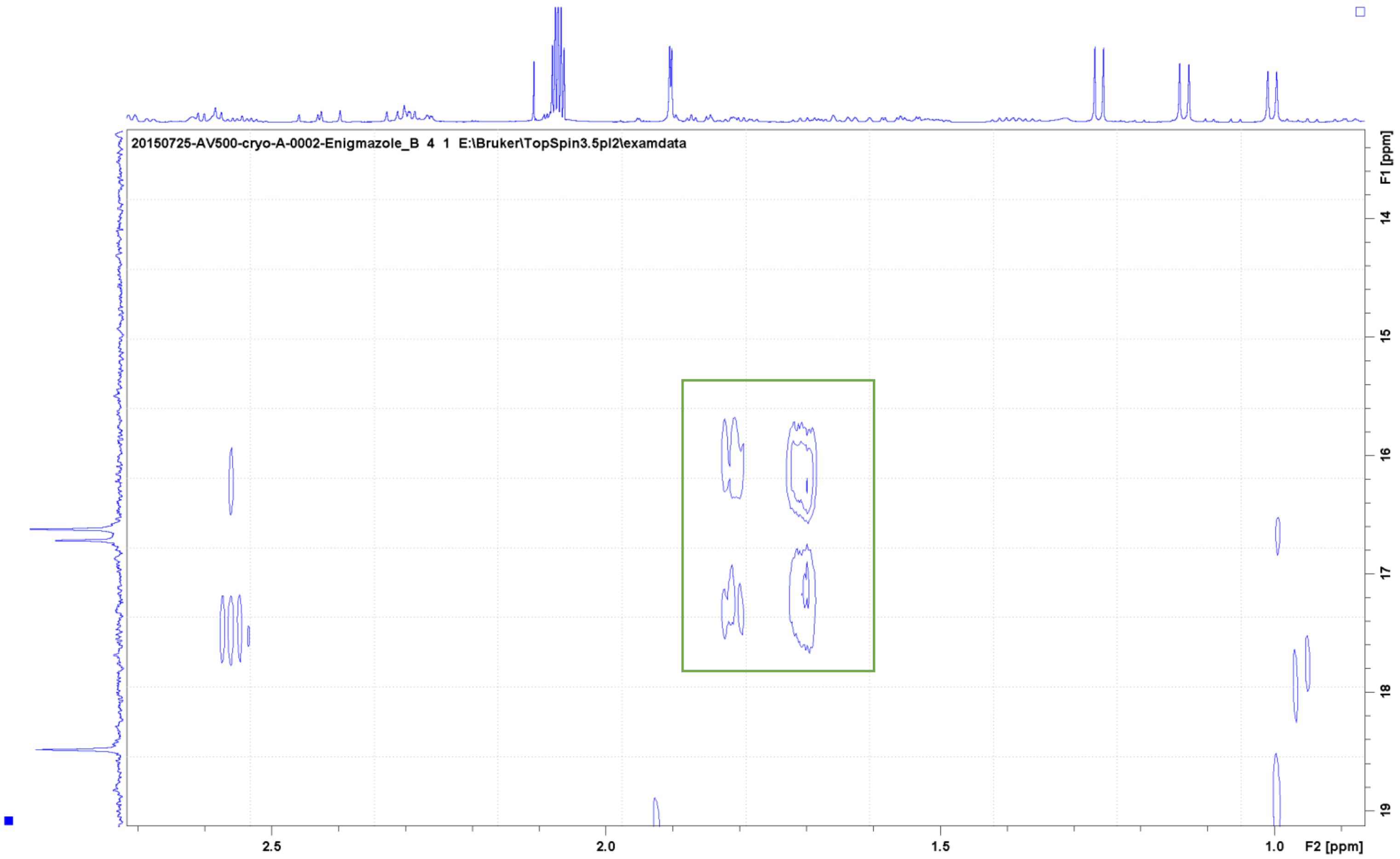

$^{3}J_{C26H3l}$=156/53 = 2.5 Hz

$^{3}J_{C26H3h}$=126/53= 2.4 Hz

Figure S17. J-HMBC spectrum expansion (500 MHz, acetone-d$_6$) of enigmazole C (**1**) (J-scale =55)

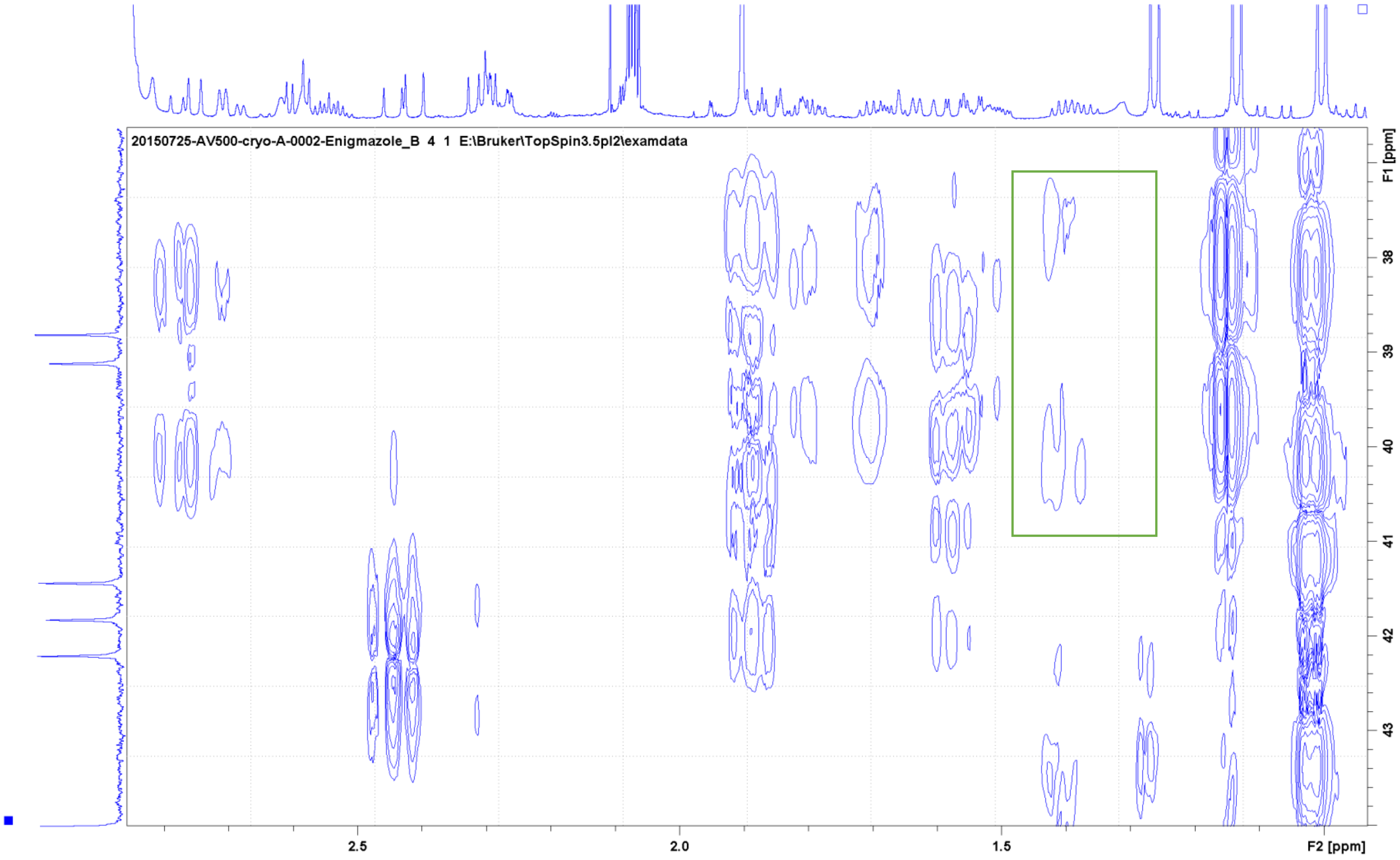

$^{3}J_{C2H4l}$=146/53 = 2.7 Hz $^{3}J_{C2H4h}$=370/53= 7.0 Hz

Figure S18. J-HMBC spectrum expansion (500 MHz, acetone-d$_6$) of enigmazole C (**1**) (J-scale =55)

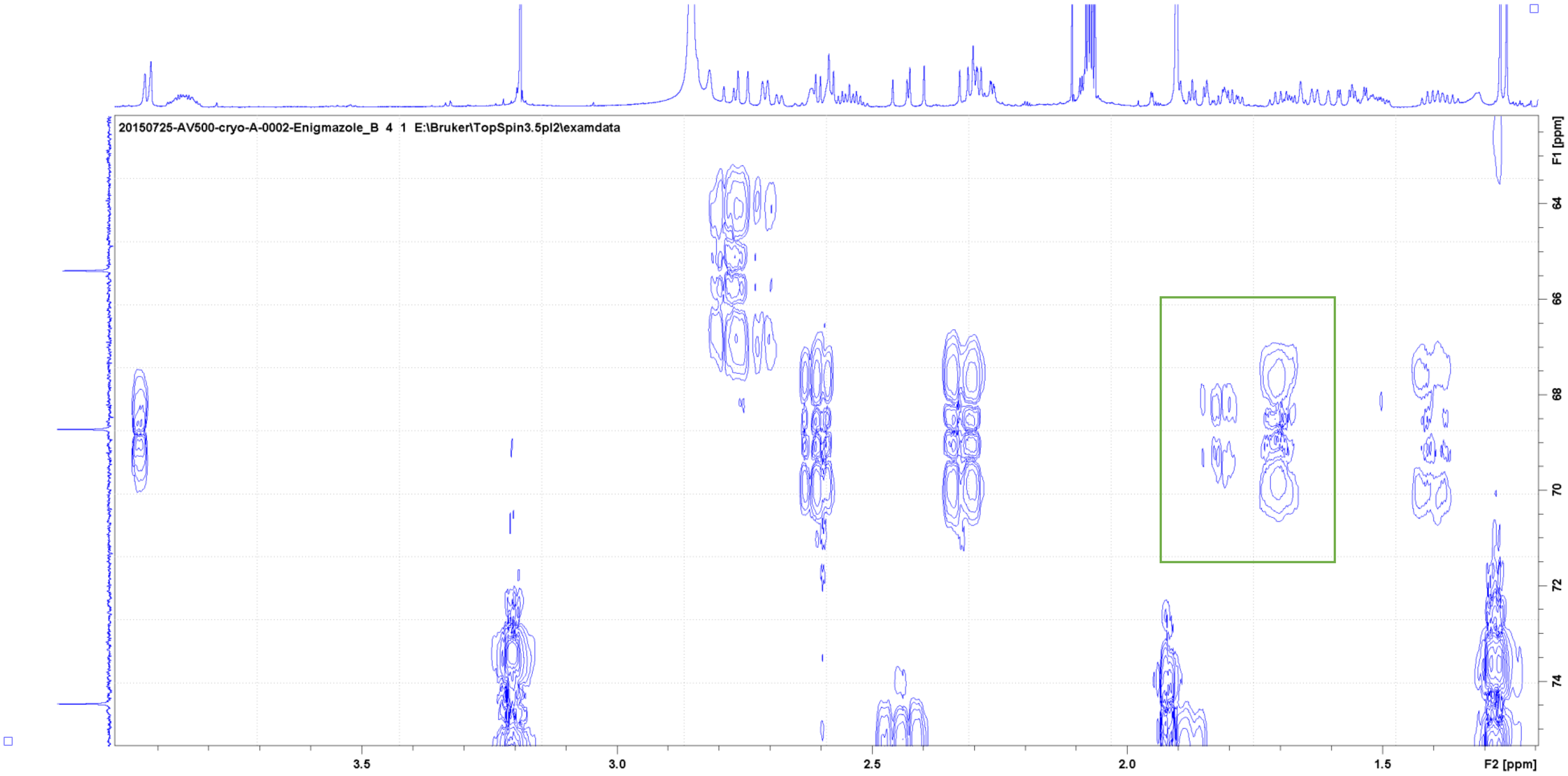

$^{3}J_{C5H3l}$=127/53 = 2.4 Hz

$^{3}J_{C5H3h}$=318/53= 6.0 Hz

Figure S19. J-HMBC spectrum expansion (500 MHz, acetone-d$_6$) of enigmazole C (**1**) (J-scale =55)

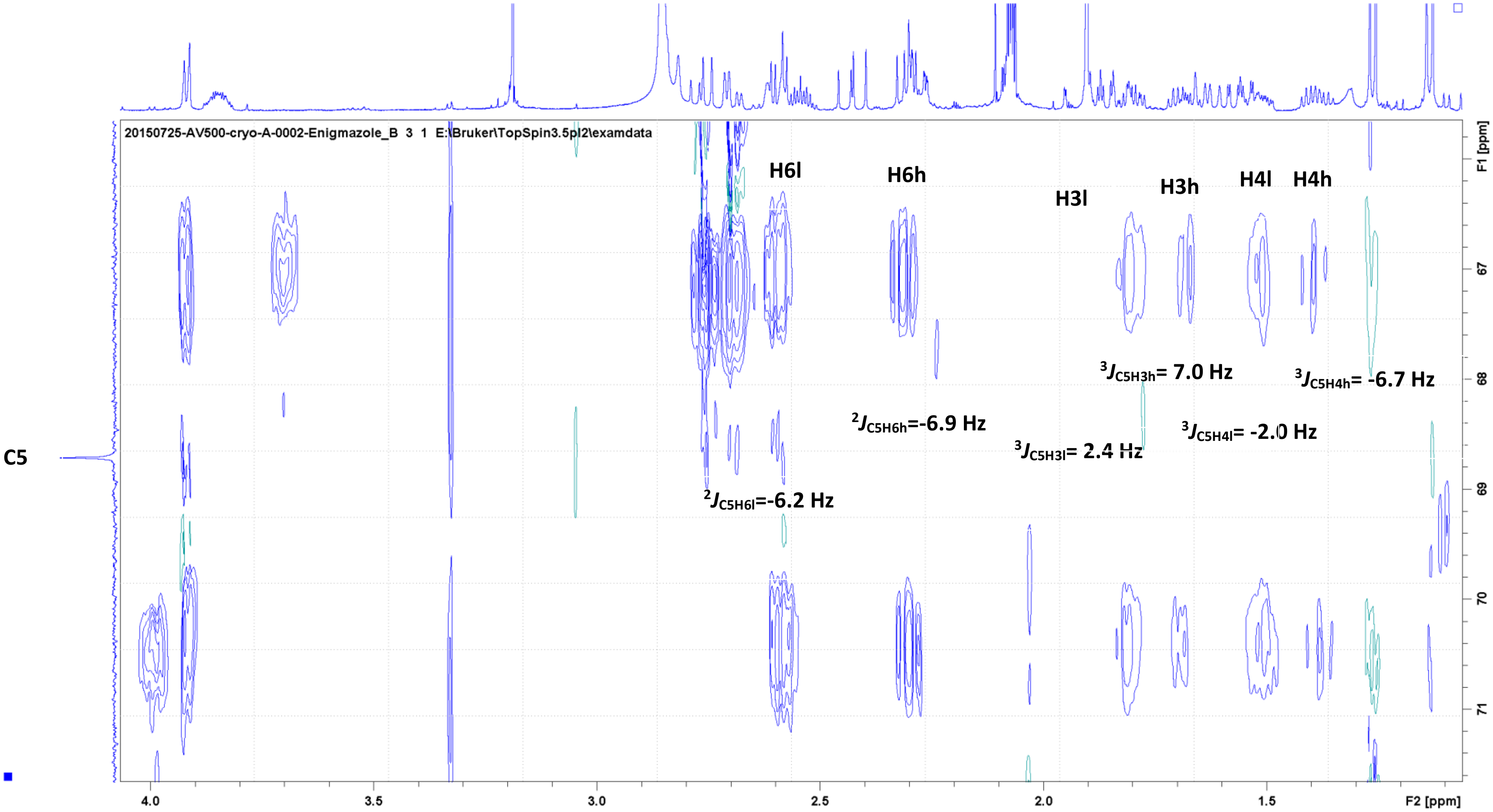

Figure S20. HSQC-HECADE spectrum expansion (500 MHz, acetone-d_6) of enigmazole C (**1**)

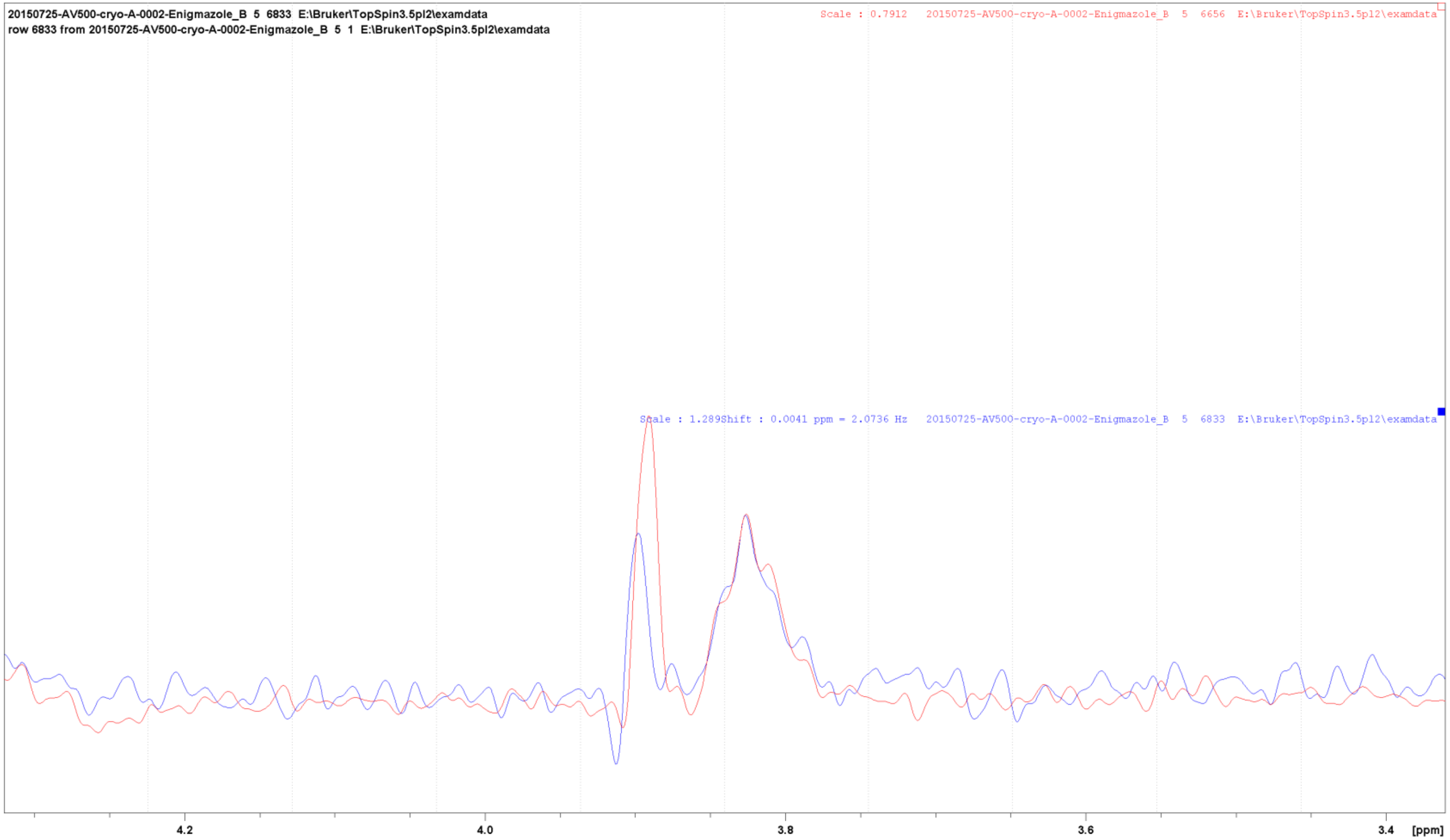

$^{3}J_{C3H5}$**=2.1 Hz**

Figure S21. HSQC-HECADE C3alfa and C3beta projections from the HSQC-HECADE (500 MHz, acetone-d_6))of enigmazole C (**1**)

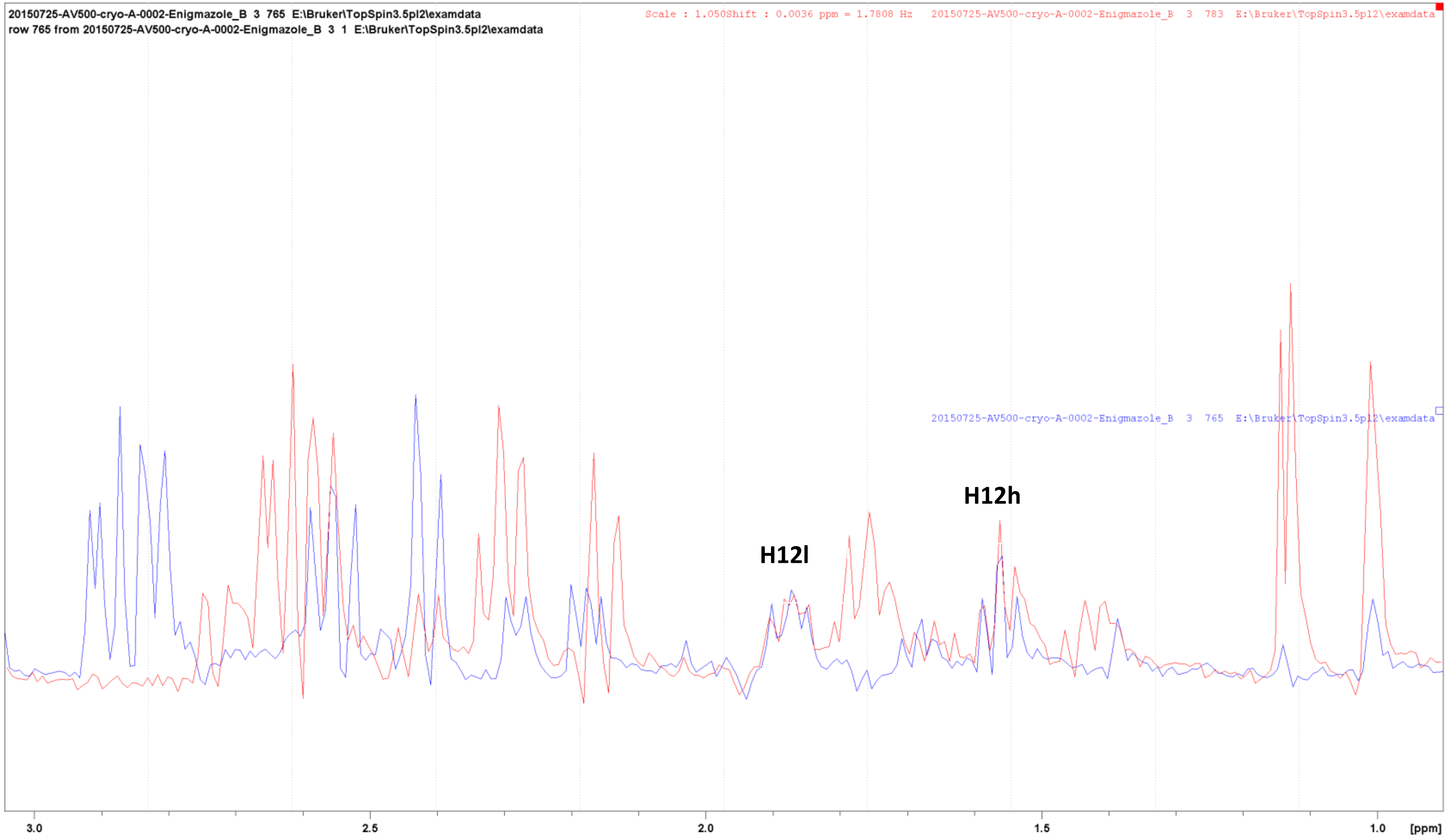

$^{3}J_{C10H12l}$=$^{3}J_{C10H12h}$=1.7 Hz

Figure S22. HSQC-HECADE C10alfa and C10beta projections from the HSQC-HECADE (500 MHz, acetone-d_6))of enigmazole C (**1**)

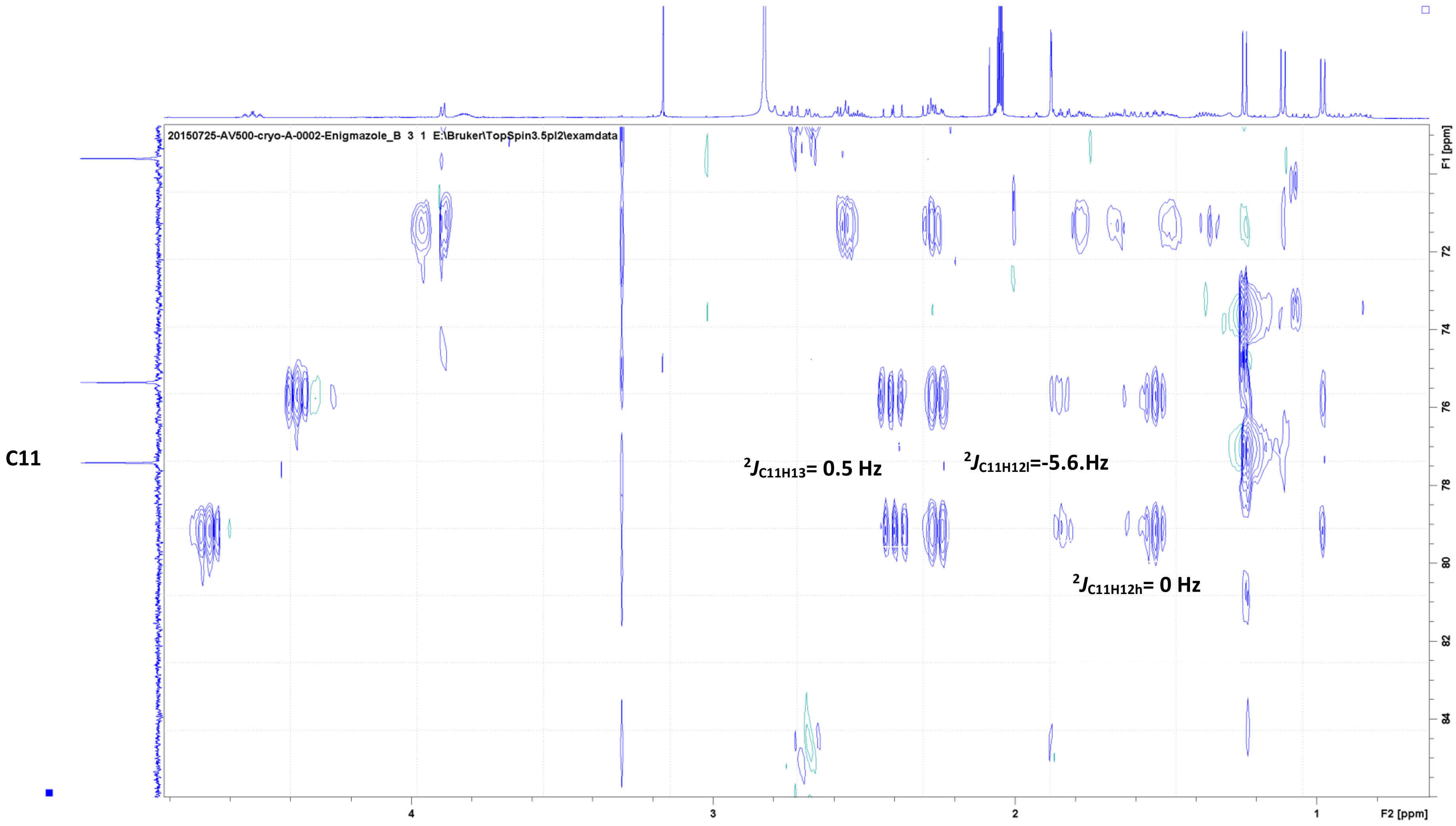

Figure S23. HSQC-HECADE spectrum expansion (500 MHz, acetone-d6) of enigmazole C (**1**)

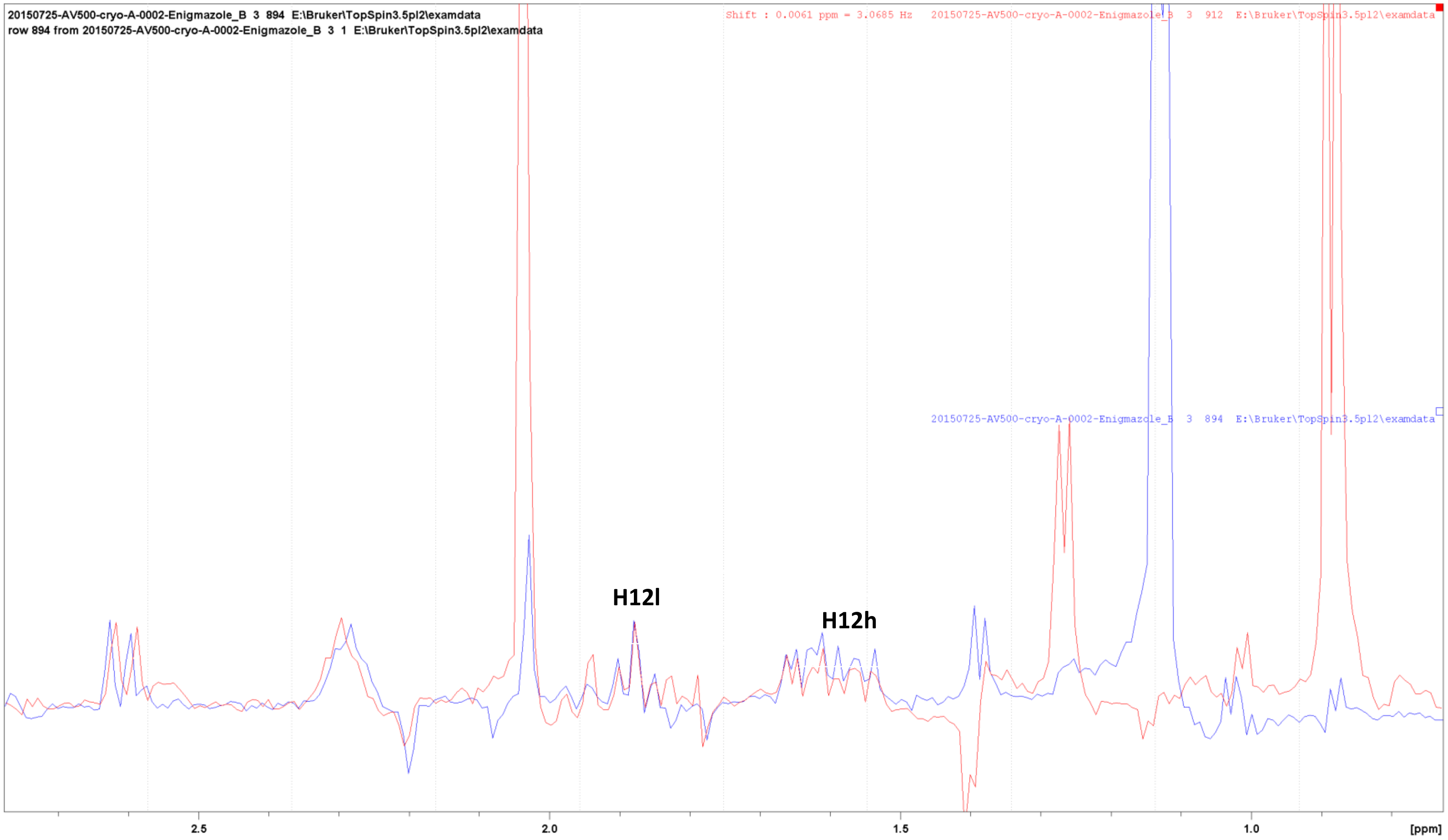

Figure S24. HSQC-HECADE C27alfa and C27beta projections from the HSQC-HECADE (500 MHz, acetone-d6))of enigmazole C (1) $^{3}J_{C27H12l}$=$^{3}J_{C27H12h}$=**3.0 Hz**

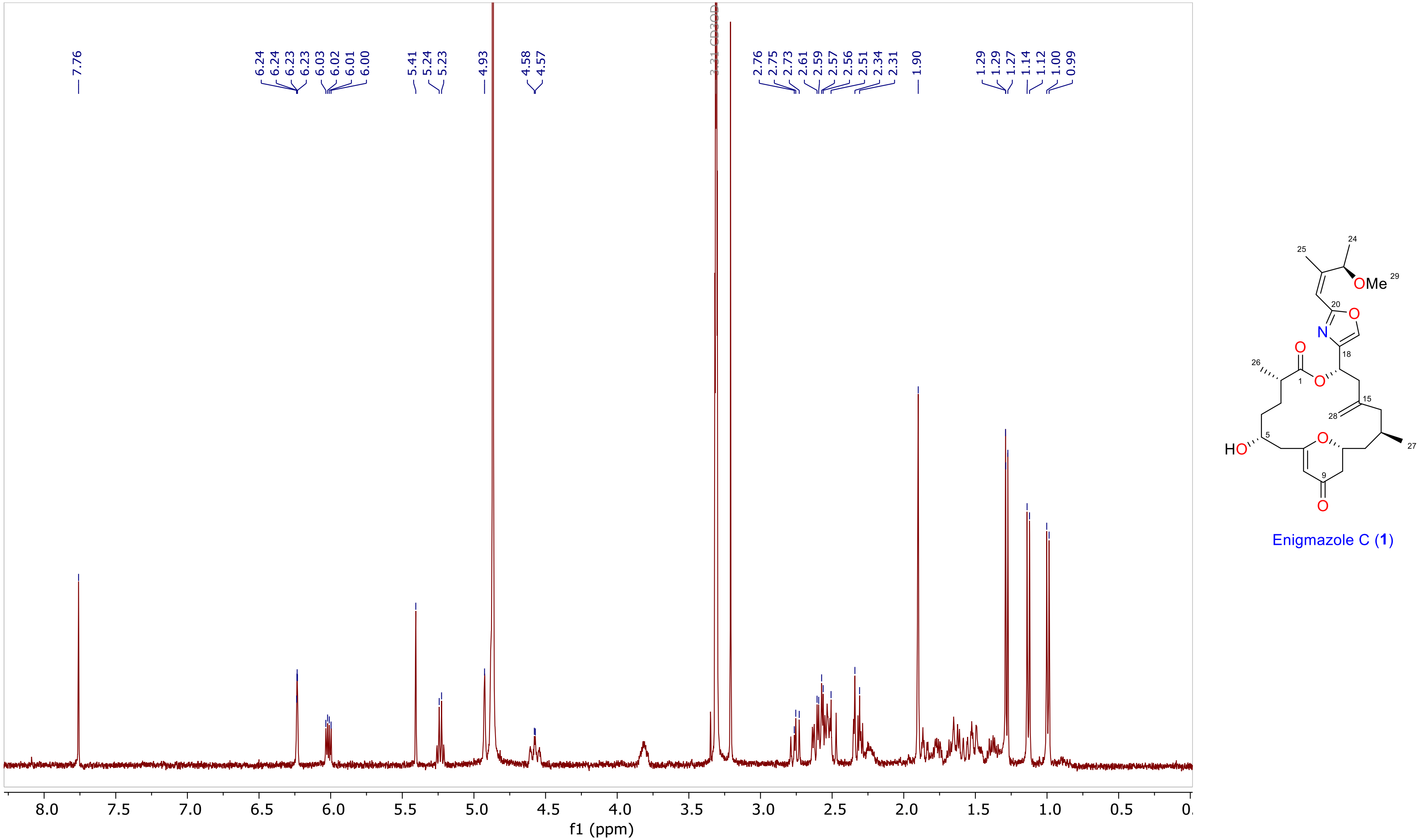

Figure S25. ^{1}H NMR spectrum (500 MHz, CD_3OD) of enigmazole C (**1**).

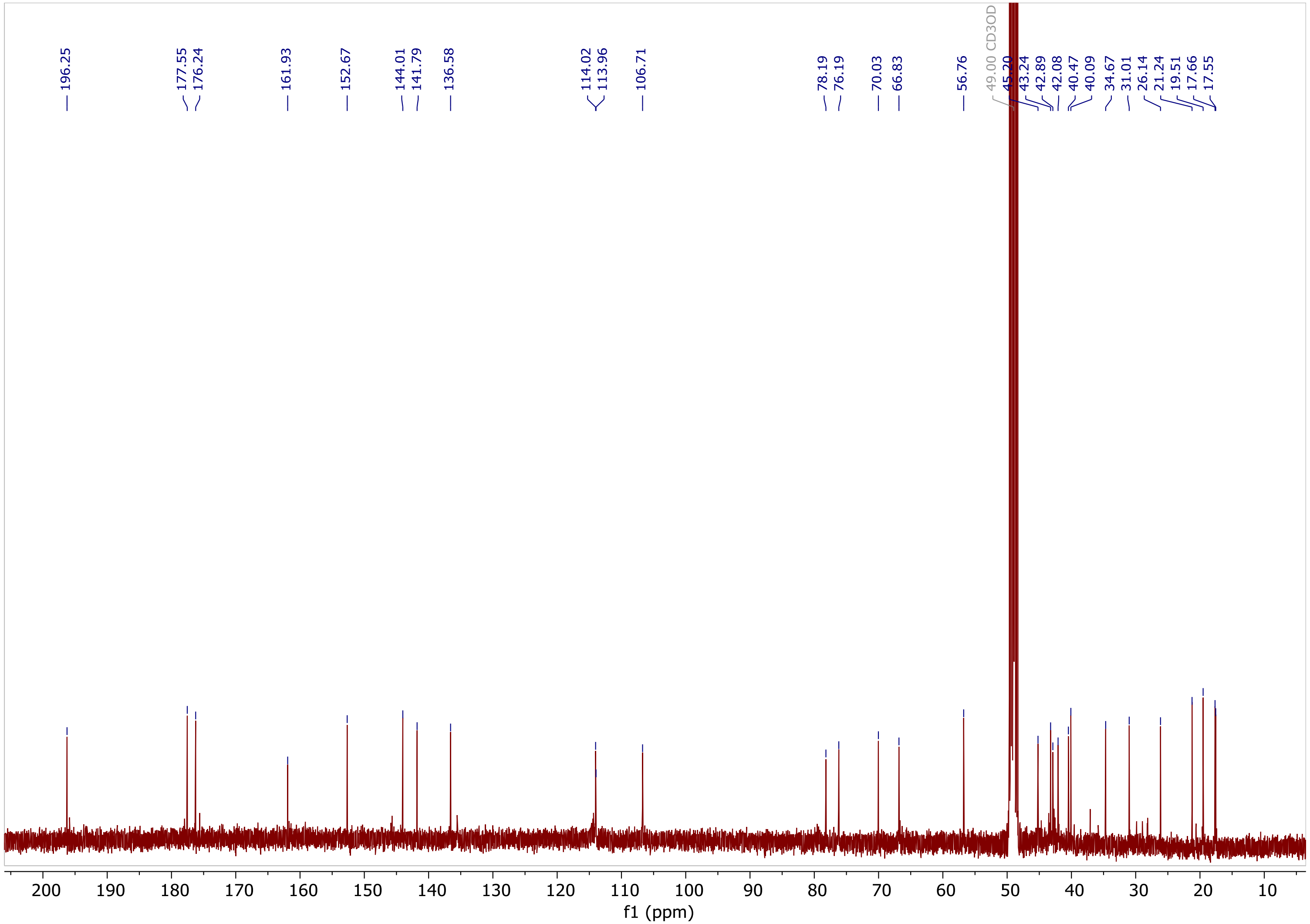

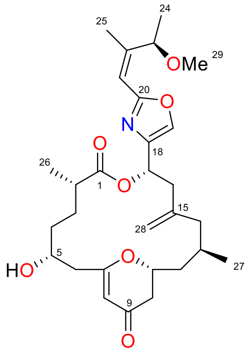

Enigmazole C (**1**)

Figure S26. ^{13}C NMR spectrum (125 MHz, CD_3OD) of enigmazole C (**1**).

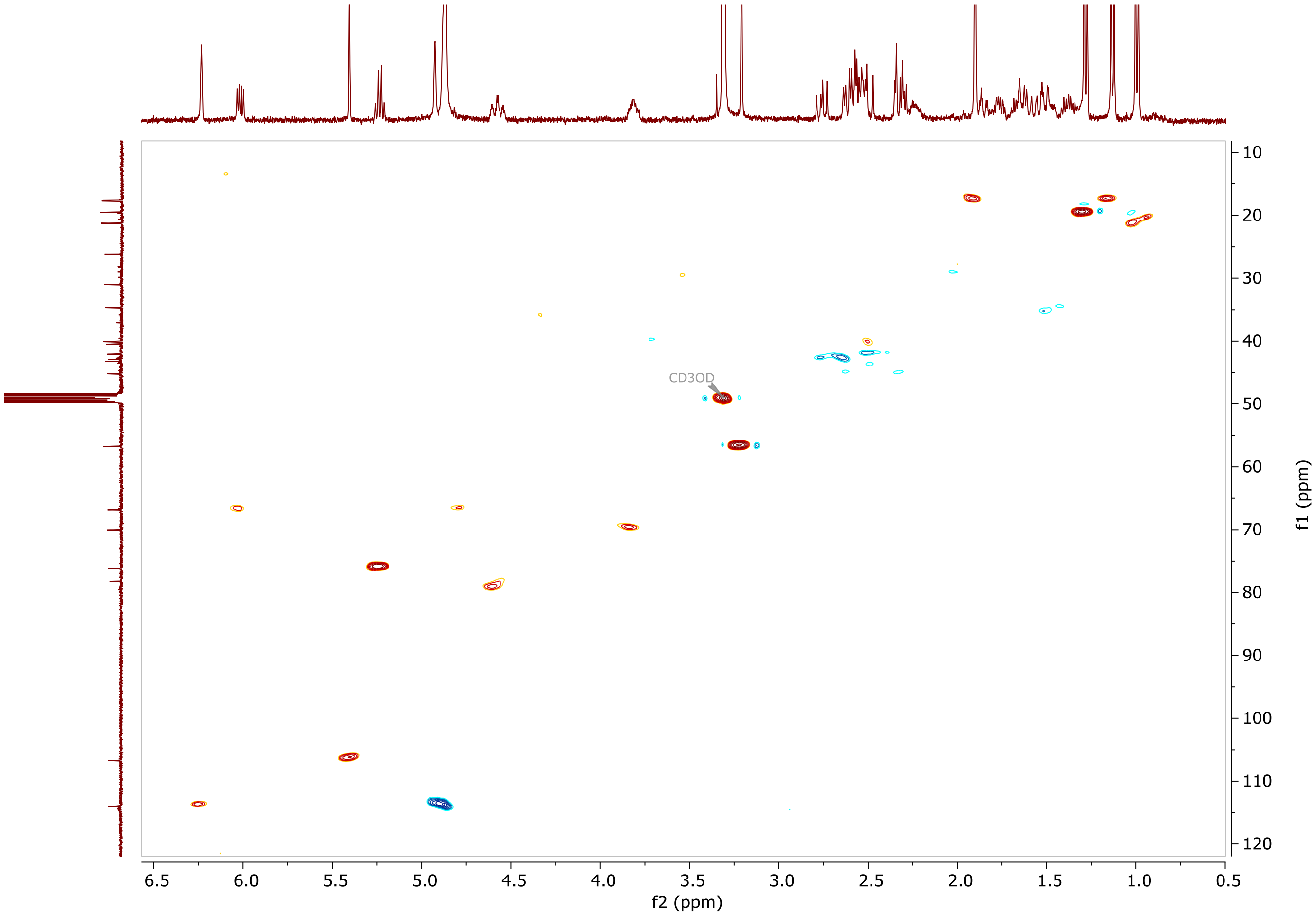

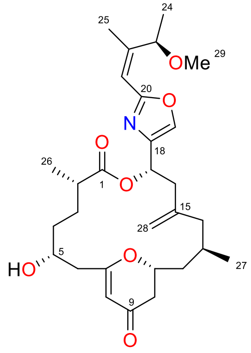

Figure S27. ^{1}H-^{13}C Edited-HSQC spectrum (500 MHz, CD_3OD) of enigmazole C (**1**).

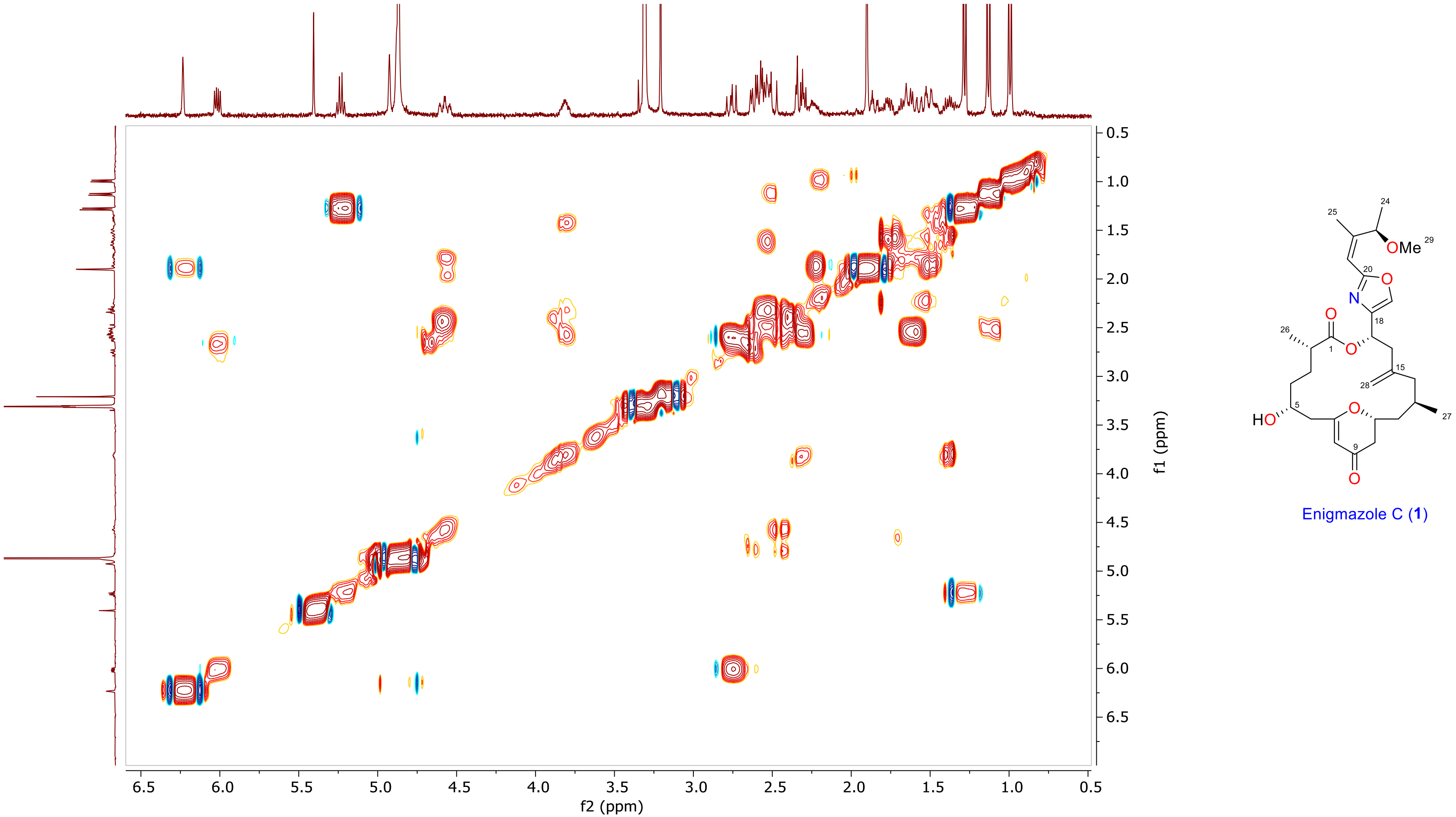

Figure S28. ^{1}H-^{1}H COSY spectrum (500 MHz, CD_3OD) of enigmazole C (**1**).

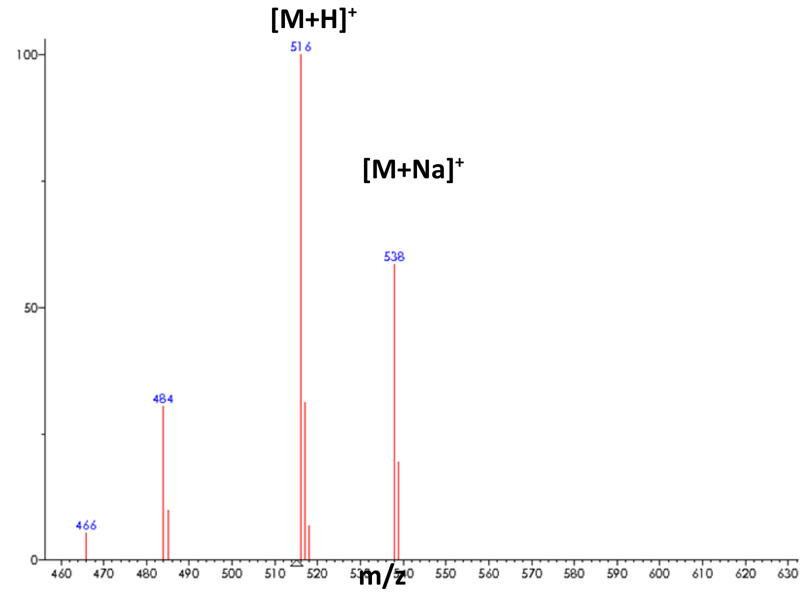

Figure S29. LR-ESIMS (+) of enigmazole C (**1**).

Overlaid Spectra:

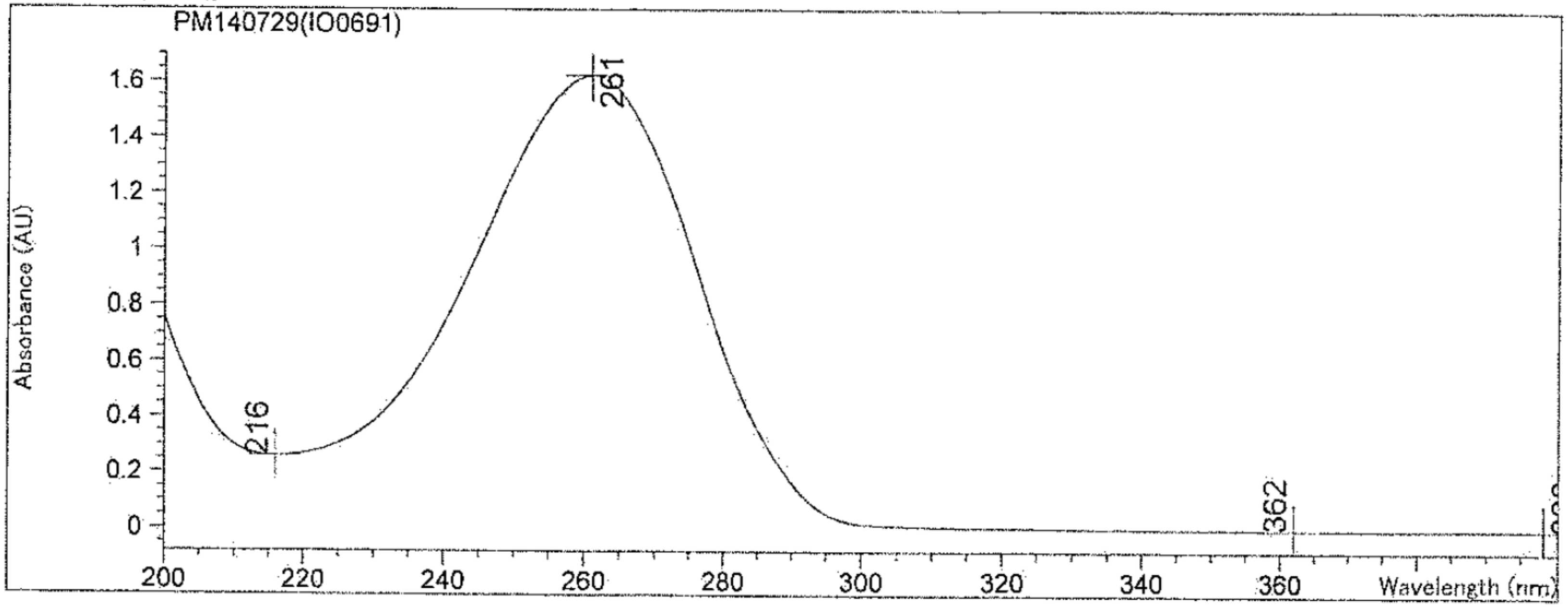

#	Name	Peaks(nm)	Abs(AU)	Valleys(nm)	Abs(AU)
1	PM140729(IO0691)	261.0	1.62410	362.0	-6.6996E-4
1		398.0	-1.4067E-4	216.0	0.25793
1		***	***	***	***

Figure S30. UV (MeOH) spectrum of enigmazole C (**1**).

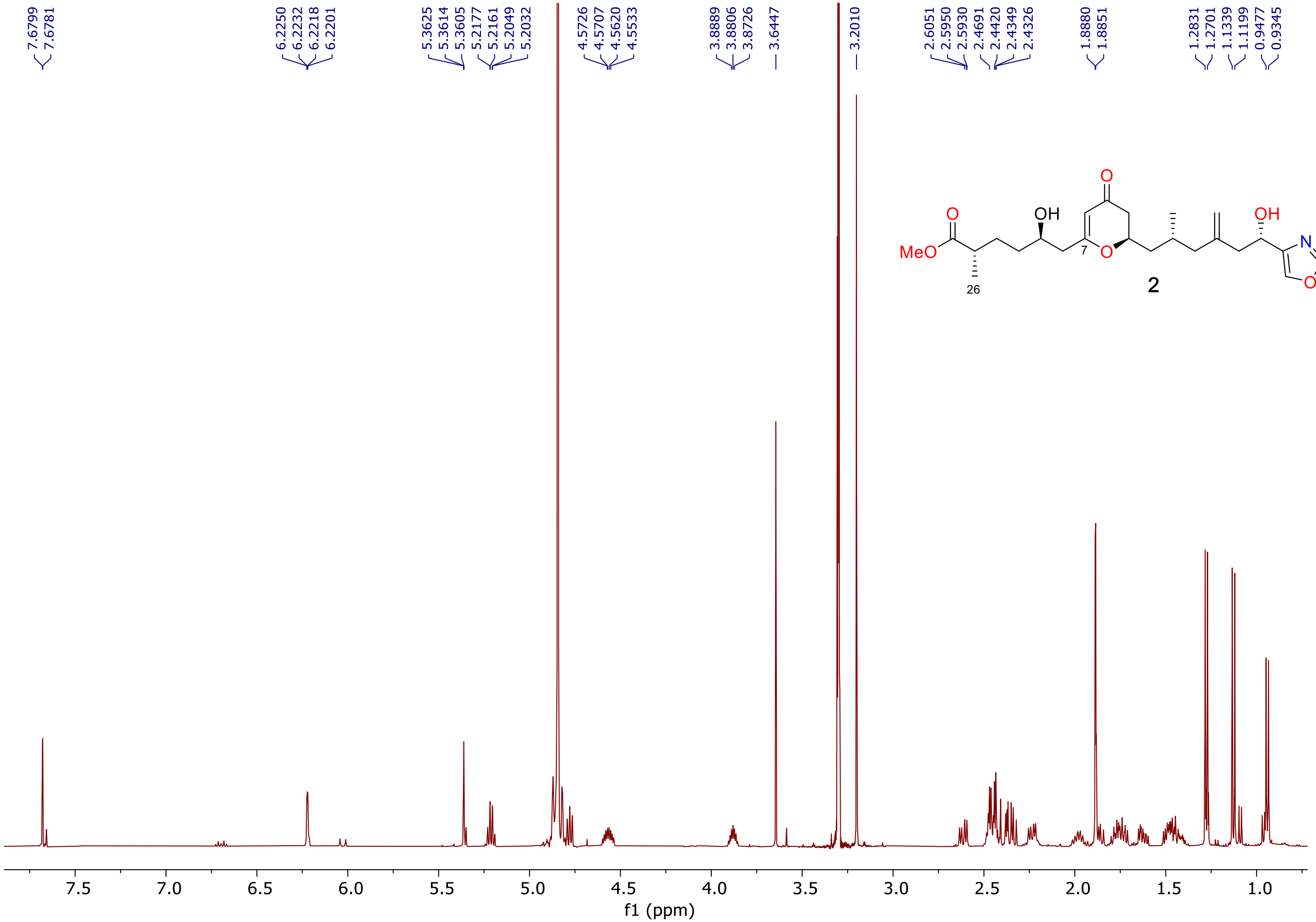

Figure S31. ^{1}H NMR spectrum (500 MHz, CD_3OD) of enigmazole E (**2**).

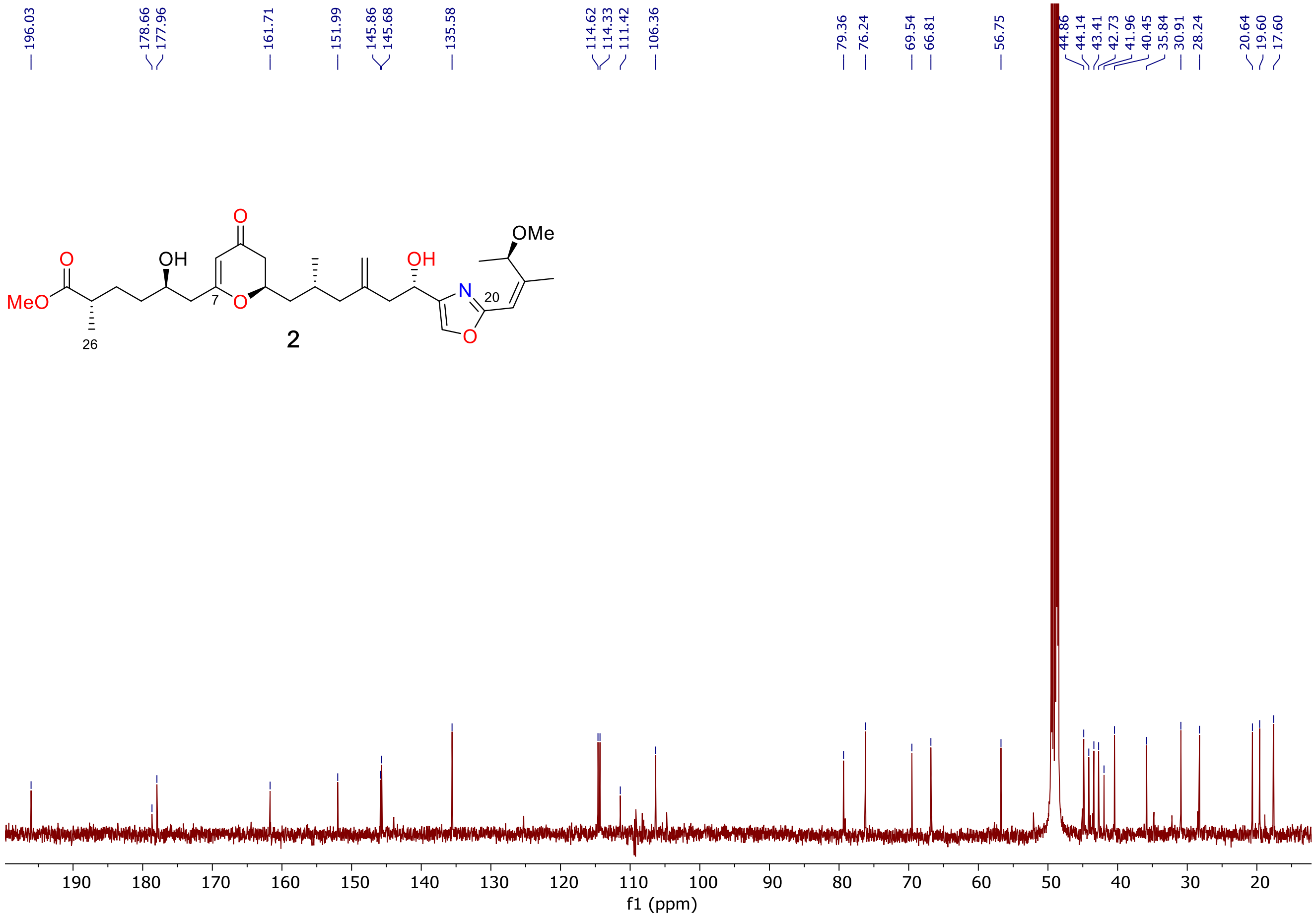

Figure S32. ^{13}C NMR spectrum (125 MHz, CD_3OD) of enigmazole E (**2**).

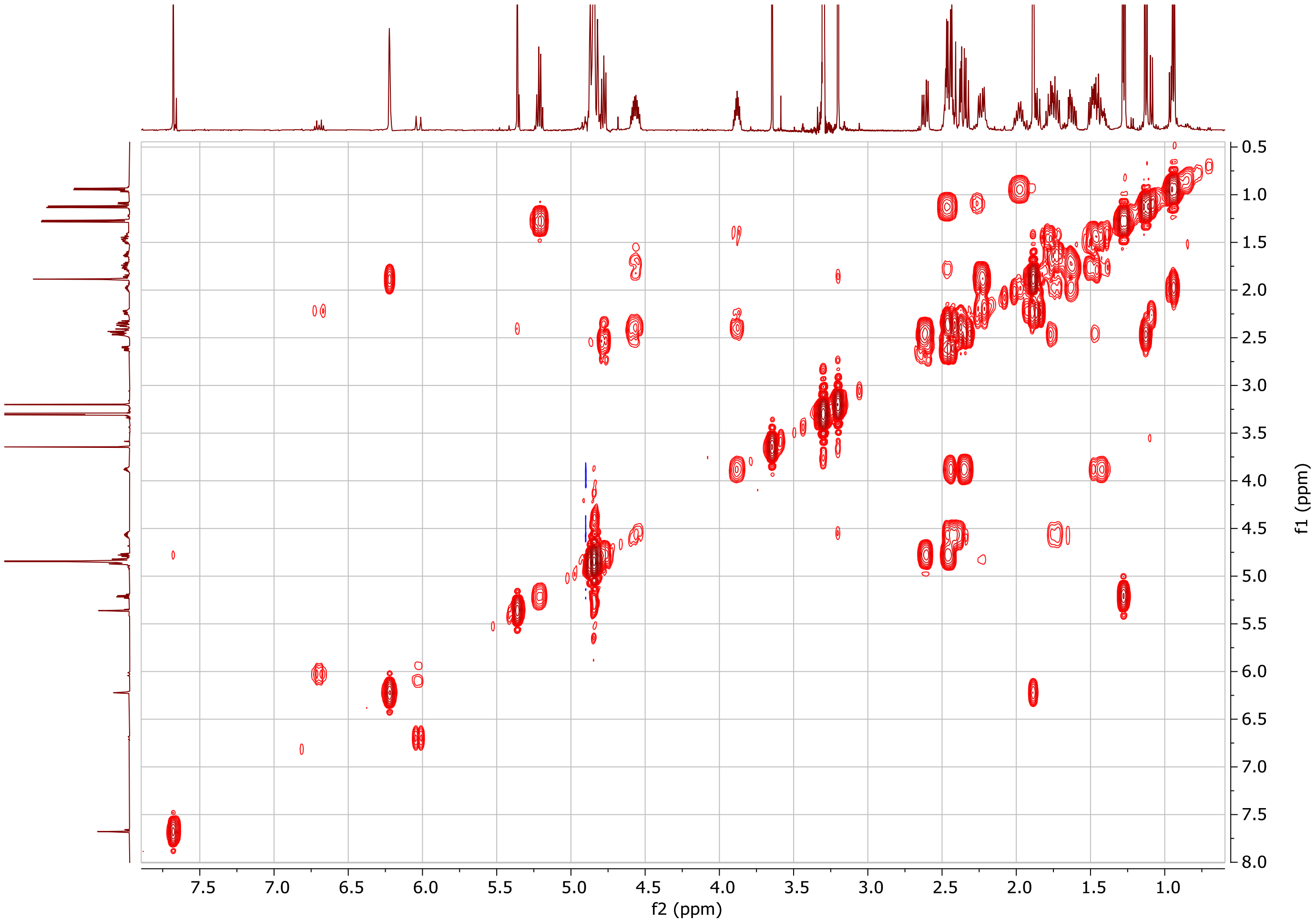

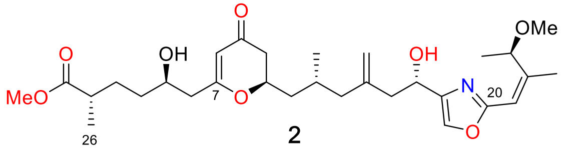

2

Figure S33. ^{1}H-^{1}H COSY spectrum (500 MHz, CD_3OD) of enigmazole E (**2**).

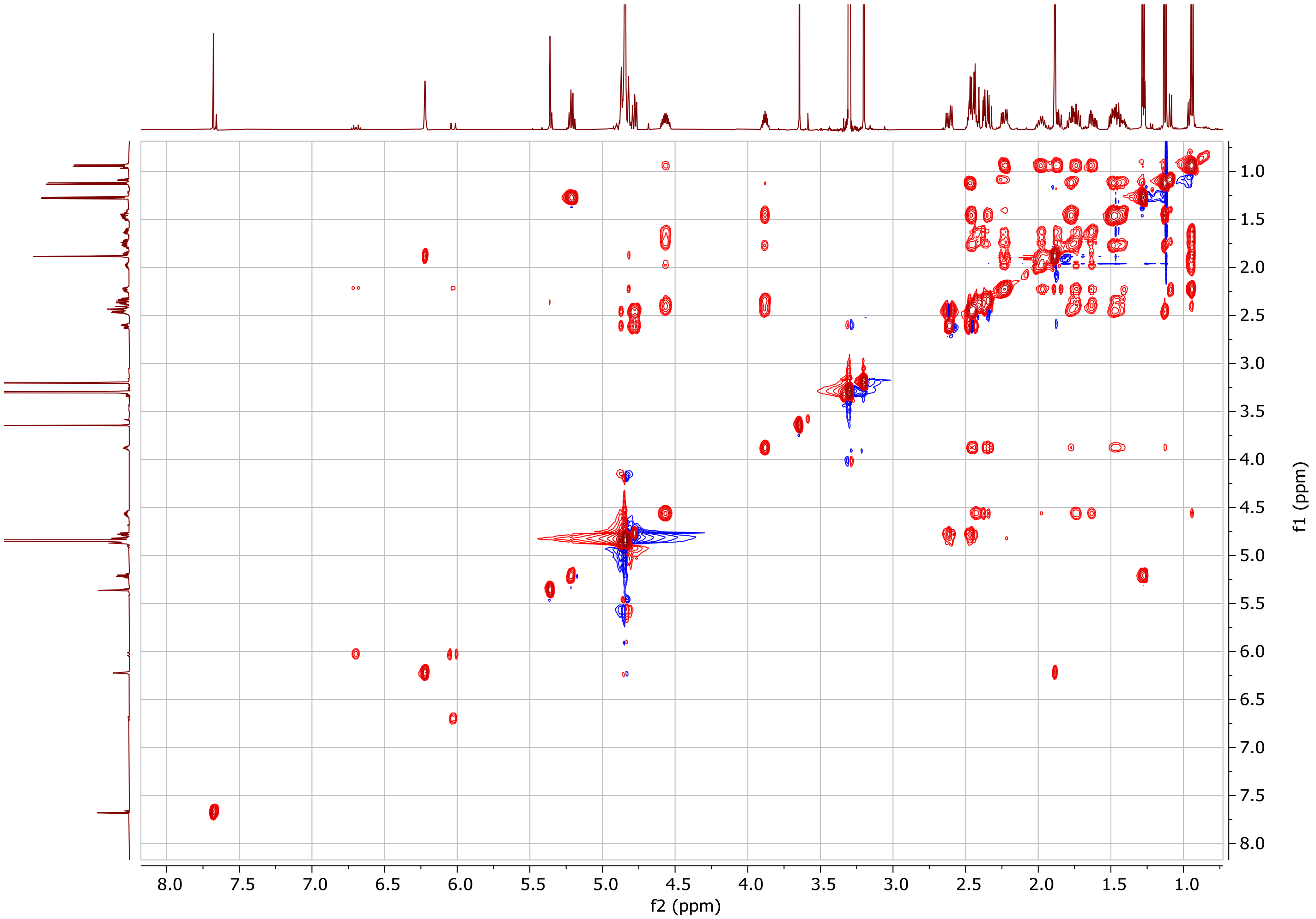

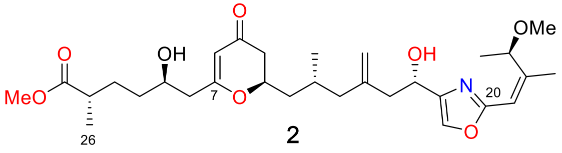

Figure S34. ^{1}H-^{1}H TOCSY spectrum (500 MHz, CD_3OD) of enigmazole E (**2**).

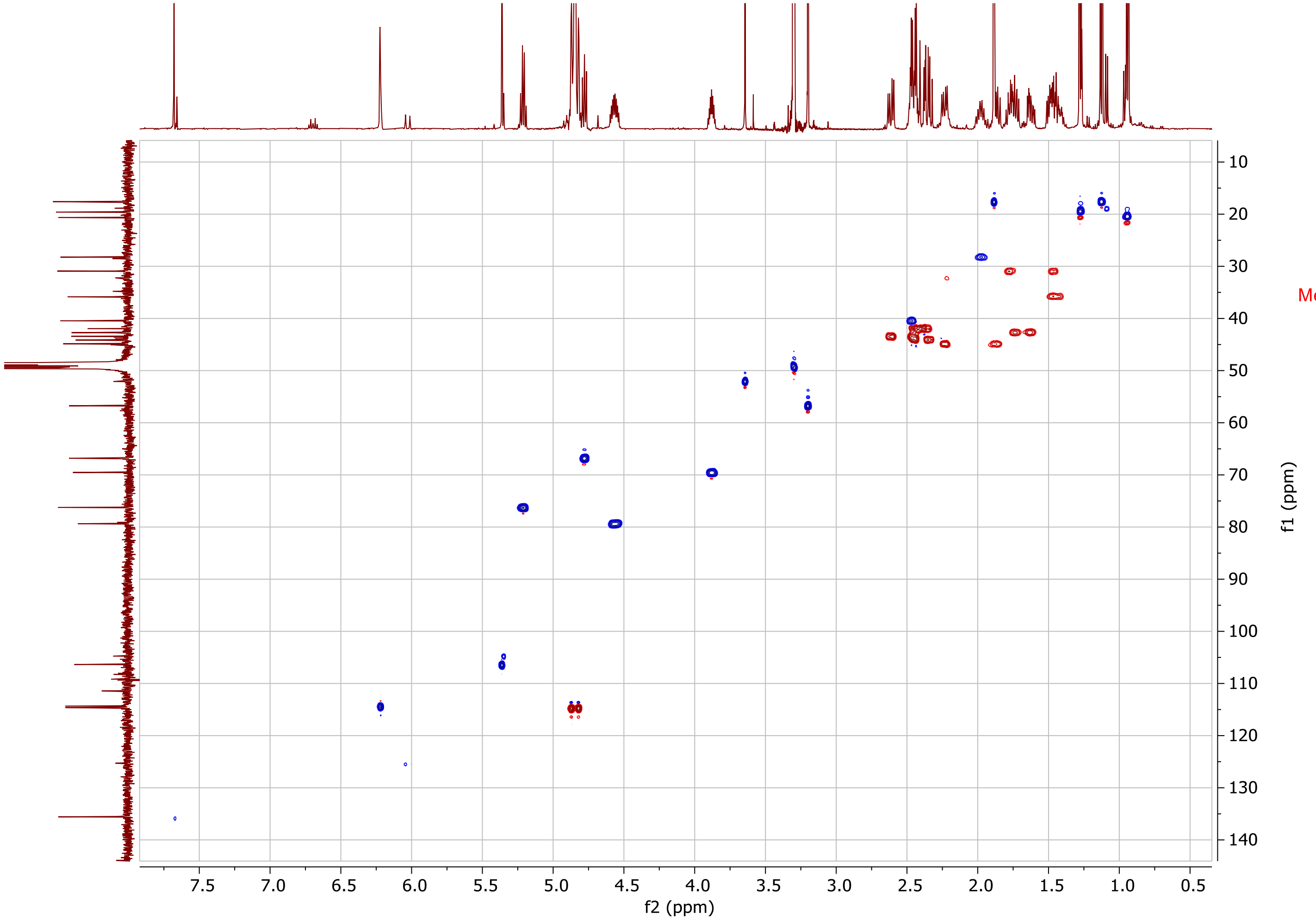

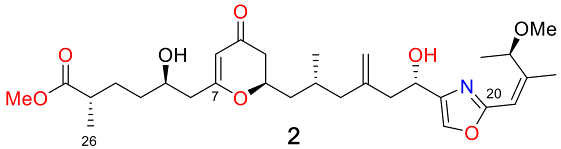

2

Figure S35. DEPT135-Edited HSQC spectrum (500 MHz, CD_3OD) of enigmazole E (**2**).

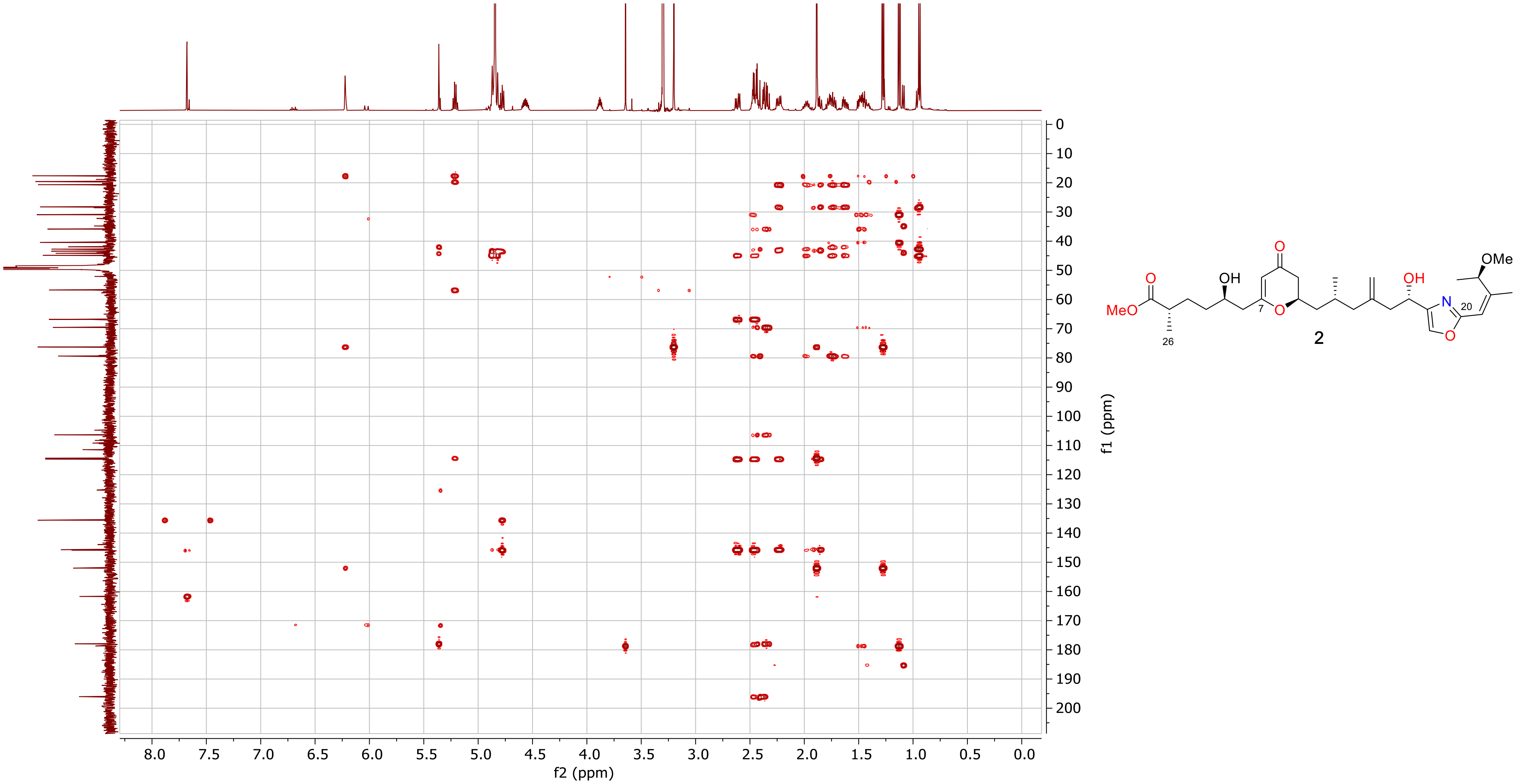

Figure S36. HMBC spectrum (500 MHz, CD_3OD) of enigmazole E (**2**).

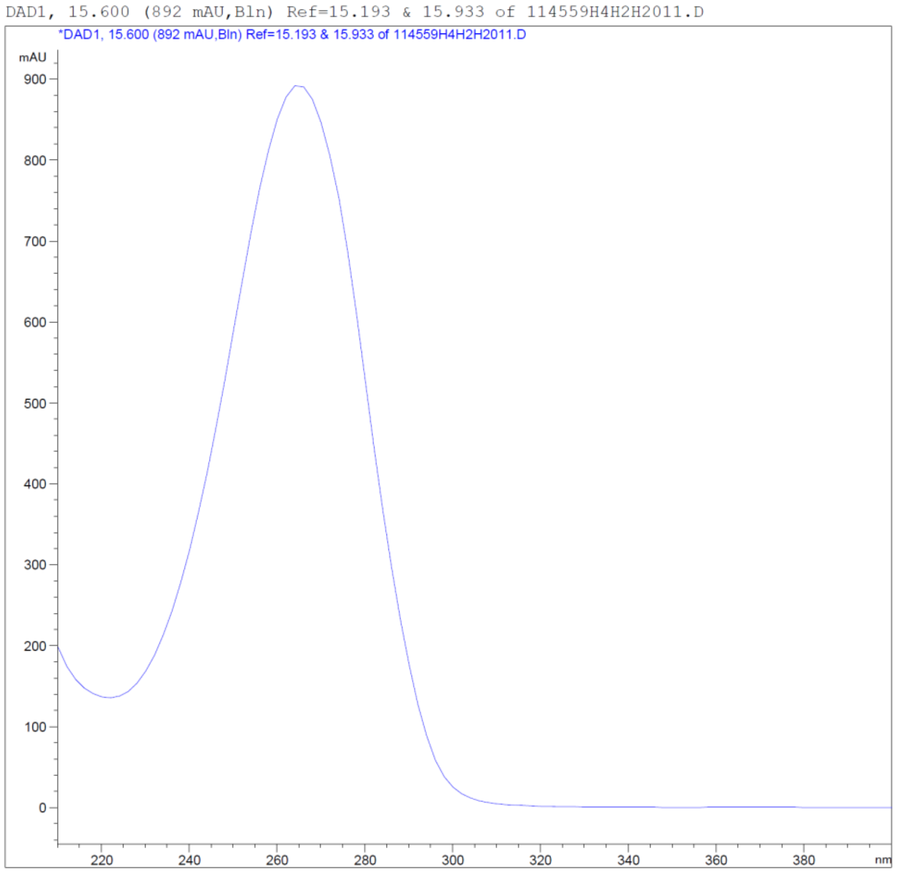

Figure S37. UV (MeOH) spectrum of enigmazole E (**2**).

Table S1. NMR data in CD_3OD of enigmazole E (**2**)

Position	^{13}C NMR ppm	^{1}H NMR ppm	m, J (Hz)
1	178.7		
2	40.5	2.46	m
3	30.9	1.78	m
3		1.46	m
4	35.8	1.47	m
4		1.43	m
5	69.5	3.88	dddd (8.3, 8.3, 4.2, 4.2)
6	44.1	2.45	m
6		2.34	dd (14.3, 8.8)
7	178.0		
8	106.4	5.36	s
9	196.0		
10	42.0	2.44	dd (16.6, 13.4)
10		2.37	dd (16.6, 4.0)
11	79.4	4.56	dddd (13.1, 8.6, 4.3,
12	42.7	1.74	ddd (14.6, 8.3, 6.4)
12		1.63	ddd (14.2, 7.0, 4.8)
13	28.2	1.98	m
14	44.9	2.23	dd (13.8, 5.6)
14		1.86	dd (13.7, 8.3)
15	145.7		
16	43.4	2.61	dd (14.2, 5.9)
16		2.45	dd (14.2, 6.5)
17	66.8	4.78	dd (6.9)
18	145.9		
19	135.6	7.67	d (0.8)
20	161.7		
21	114.3	6.22	dd (1.6, 0.8)
22	152.0		
23	76.2	5.21	q (6.4)
24	19.6	1.28	d (6.5)
25	17.6	1.13	d (7.0)
26	20.6	0.94	d (6.5)
27	114.6	4.87	brs
27		4.82	brs
28	17.6	1.89	d (1.46)
29	56.8	3.20	s
30	-	-	-

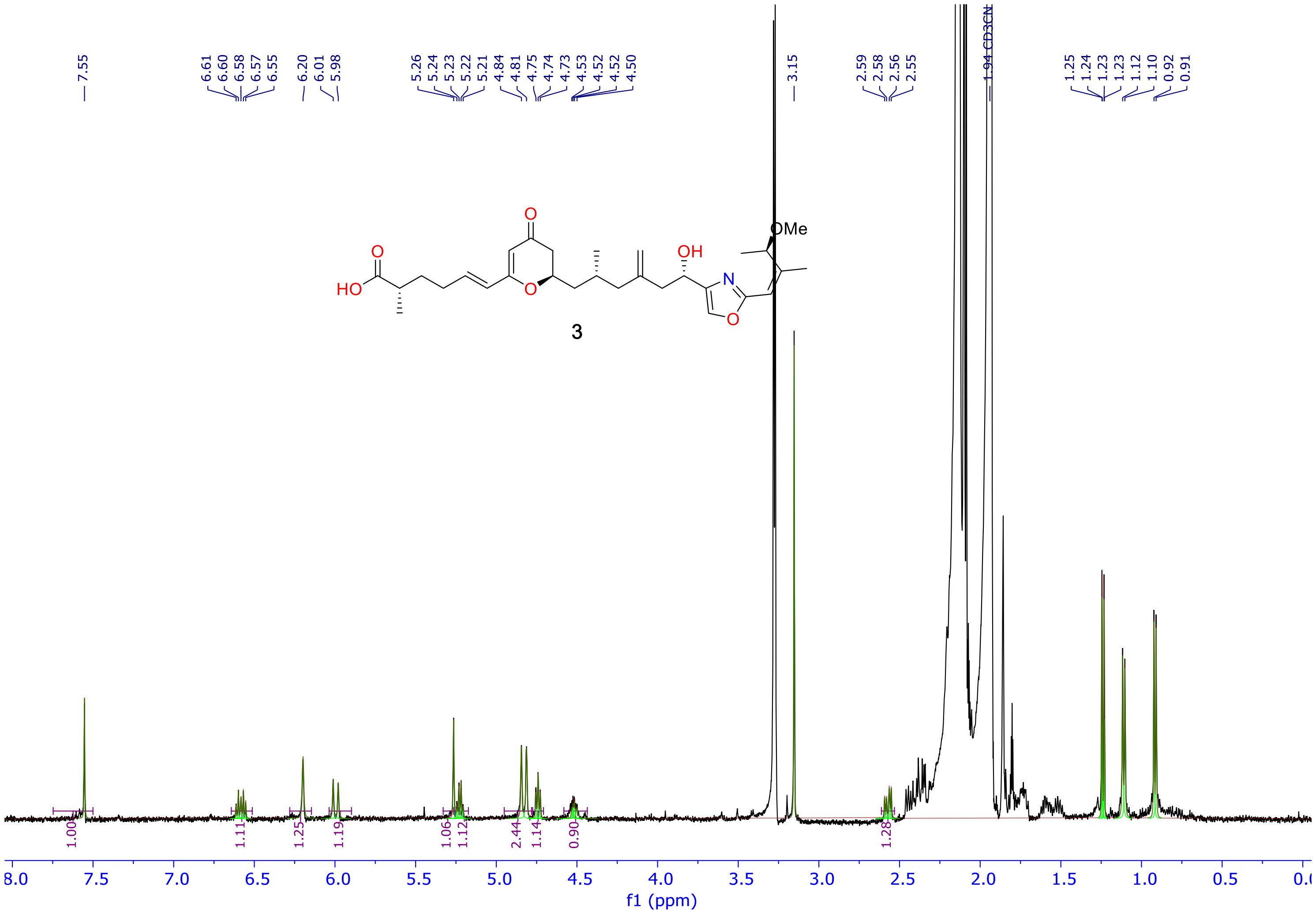

Figure S38. ^{1}H NMR spectrum (500 MHz, CD_3OD) of enigmazole D (**3**).

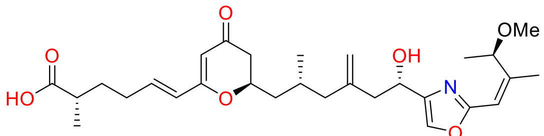

3

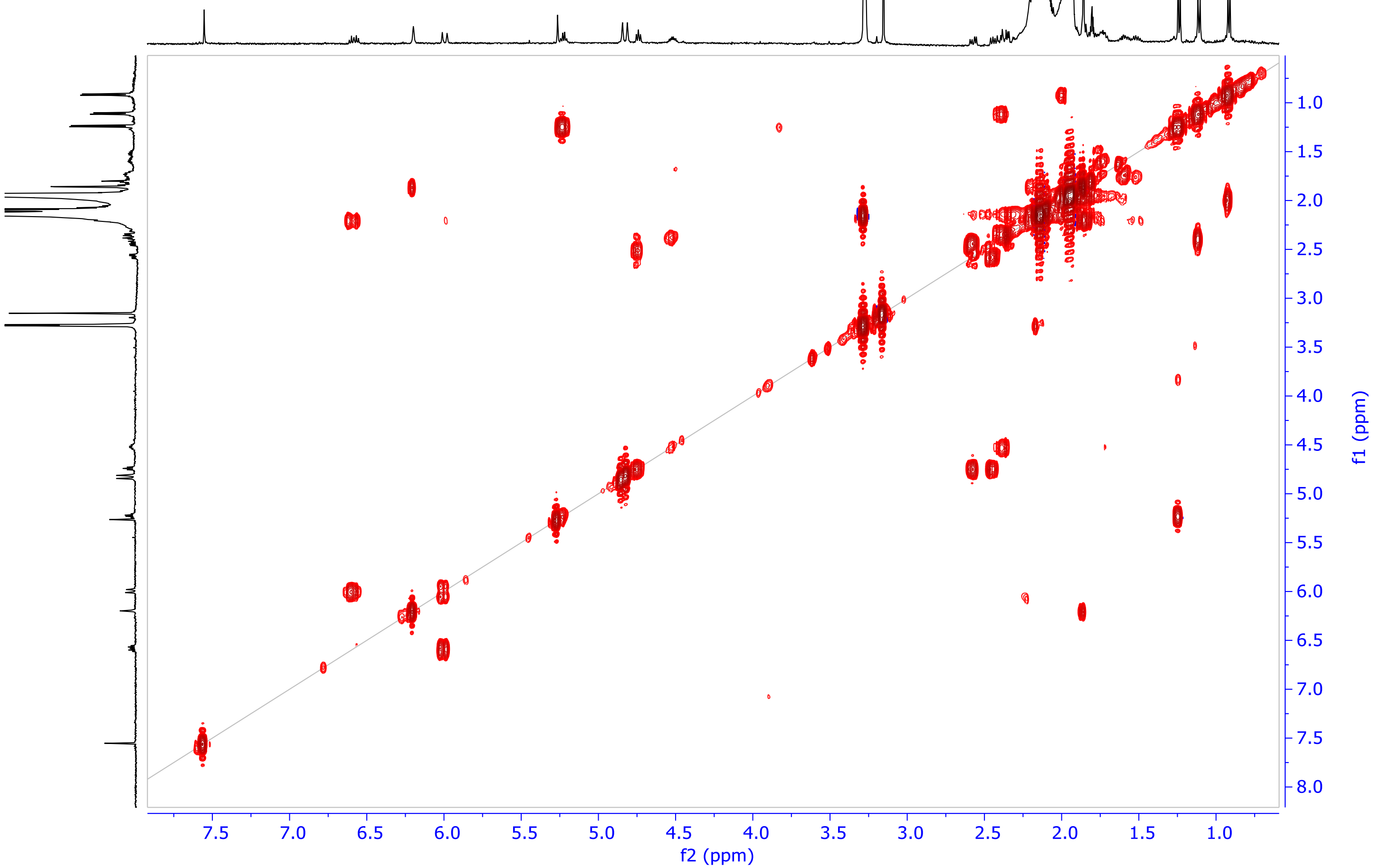

Figure S39. ^{1}H-^{1}H COSY spectrum (500 MHz, CD_3OD) of enigmazole D (**3**)

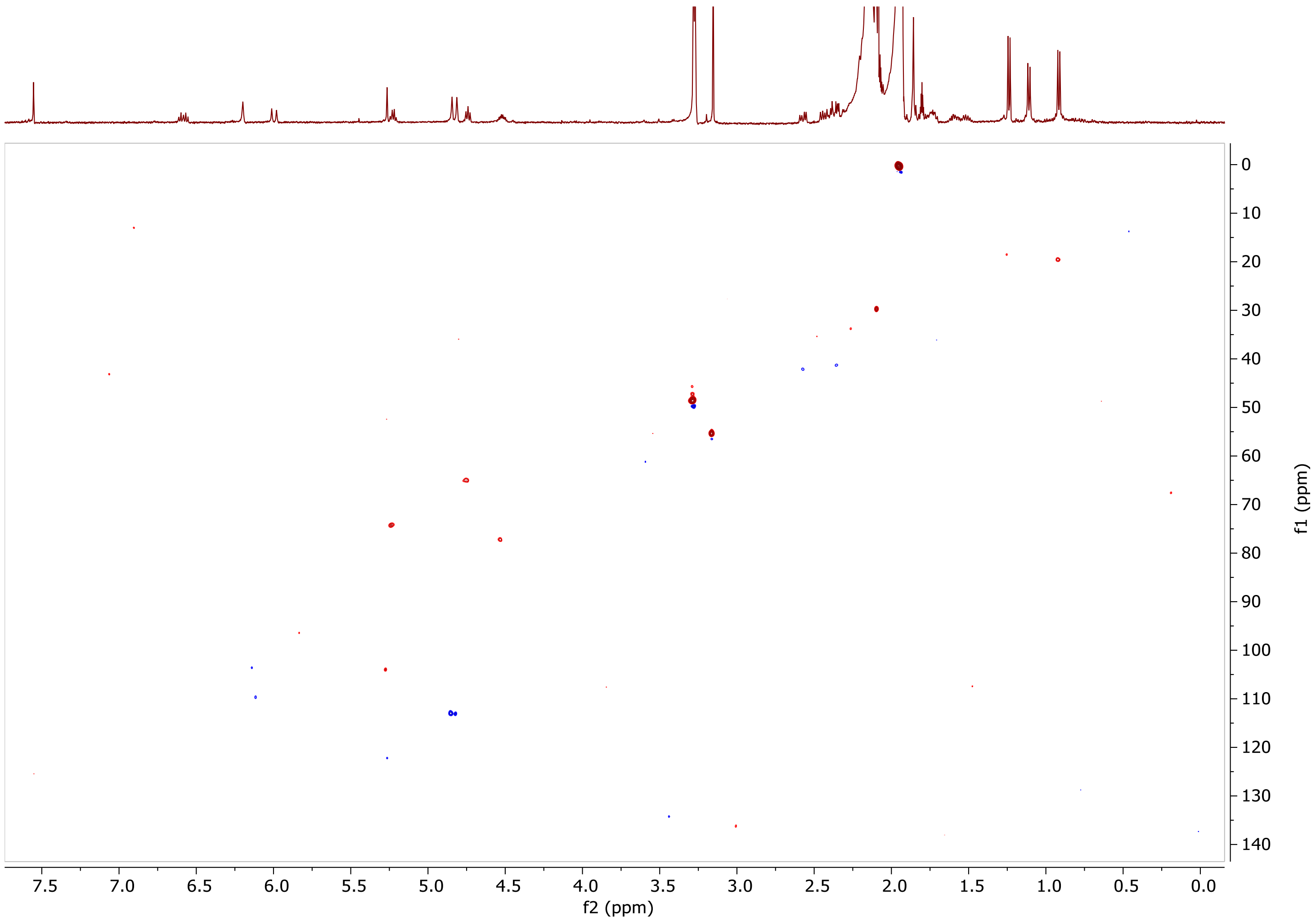

Figure S40. Edited-HSQC spectrum (500 MHz, CD_3CN) of enigmazole D **(3)**

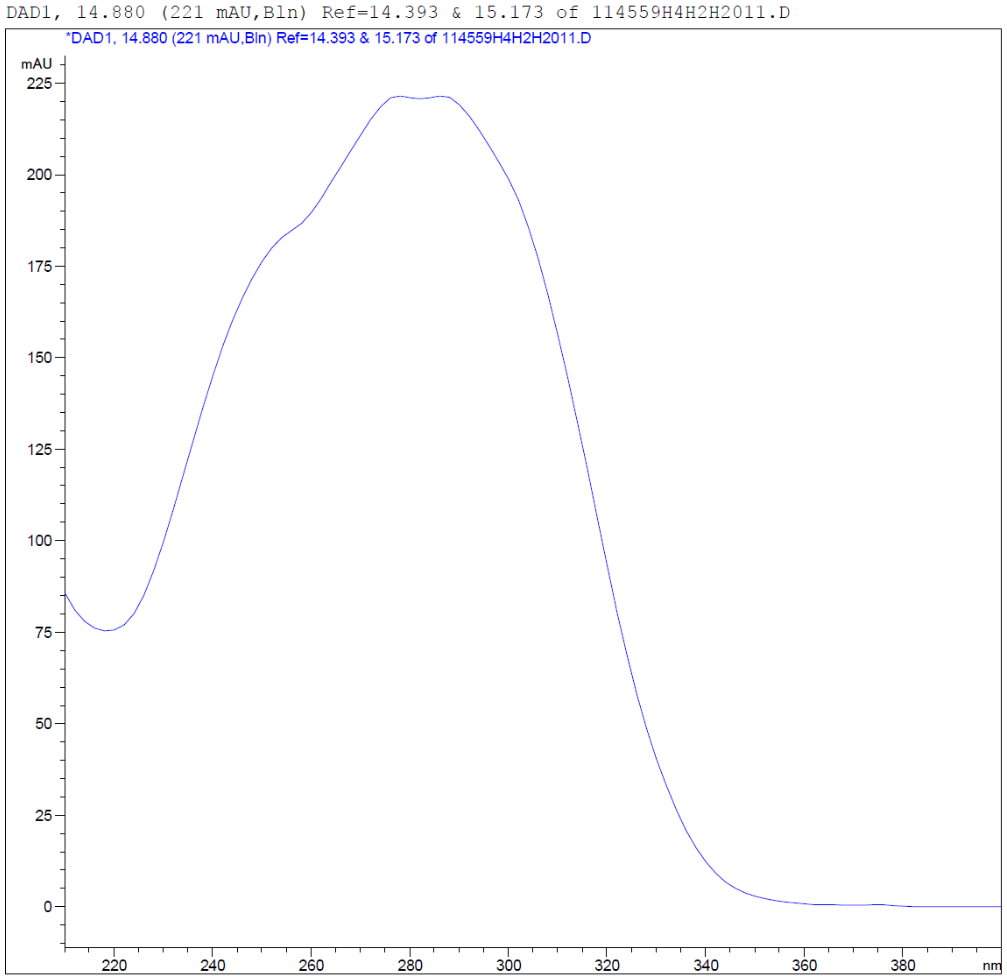

Figure S41. UV (MeOH) spectrum of enigmazole D **(3).**

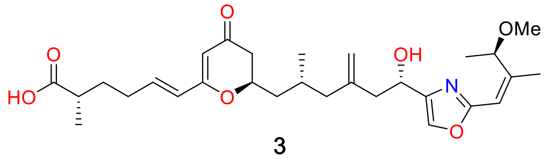

3

Table S2. NMR data in CD_3CN of enigmazole D (**3**)

Position	^{13}C NMR ppm	^{1}H NMR ppm	m, J (Hz)
1	-		
2		2.39	
3		-	
3		-	
4		2.19	
4			
5		6.58	dt, 15.6, 7.1
6	-	6.00	d, 15.6
7	-		
8	105.1	5.26	s
9	196.0		
10	42.3	2.44	
10		2.36	
11	78.4	4.52	m
12	-	1.74	
12		1.61	
13	-	1.98	
14	-	2.23	
14		1.86	
15	-		
16	43.4	2.57	dd, 14.3, 6.7
16		2.45	dd, 14.3, 6.7
17	66.1	4.74	t, 6.7
18	145.9		
19	-	7.55	s
20	161.7		
21	-	6.20	bs
22	152.0		
23	75.4	5.22	q,6.5
24	19.6	1.24	
25	-	1.11	d, 6.7
26	20.6	0.91	d, 6.7
27	114.1	4.85	bs
27		4.82	bs
-	-	1.89	
29	56.4	3.15	s

Table S3. Calculated DFT energies of the **1a-(2S5R11R13R17S23R)** diasteroisomer**.**

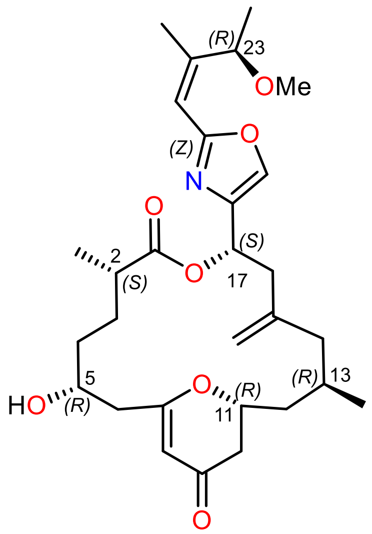

Conformers **1a**	OPLS2008 Force Field Conformotional Search Relative Energy (kcal/mol)	B3LYP/6-31+G(d,p) DFT Energy (hartree)	Δ (DFT Energy) (kcal/mol)	% Population
**1a6**	0.858102294	-1710.461923	0	64.40
**1a29**	3.018642447	-1710.460565	0.852157753	15.26
**1a1**	0	-1710.459781	1.344125116	6.65
**1a21**	2.317638623	-1710.459393	1.587598759	4.40
**1a31**	3.215033461	-1710.459381	1.595128872	4.35
**1a13**	1.829134799	-1710.45839	2.216990678	1.52
**1a11**	1.531763862	-1710.458116	2.388928252	1.14
**1a3**	0.107002868	-1710.457939	2.499997414	0.94
**1a22**	2.380234226	-1710.456575	3.355920223	0.22
**1a16**	2.030736138	-1710.456348	3.498364855	0.17
**1a2**	0.080807839	-1710.456243	3.564253341	0.16
**1a34**	3.28166826	-1710.456065	3.675950012	0.13
**1a4**	0.739268642	-1710.455876	3.794549287	0.11
**1a12**	1.58874283	-1710.45573	3.886165658	0.09

14 conformers counting for the 99.54% of the DFT conformational population

DFT COORDINATES FOR CONFORMATIONAL SEARCH OF **1a**
(2S*, 5R*, 11R*, 13R* , 17S*, 23R*)

Compound 1a: Isomer 2S, 5R, 11R, 13R, 17S, 23R conformer 1

C	-2.81439	1.21179	1.55029
C	-4.03916	1.38349	2.11606
C	-5.13199	2.03955	1.39955
C	-4.76046	2.52652	-0.00188
C	-3.68677	1.64237	-0.63154
O	-2.54456	1.52002	0.26034
C	-1.6505	0.54101	2.21751
C	-1.69819	-0.98207	1.92485
C	-0.42229	-1.70245	2.35852
C	-0.49393	-3.23245	2.19811
C	-0.90184	-3.78355	0.80356
C	0.15406	-3.47406	-0.24678
C	-1.14516	-5.2966	0.87114
O	0.07152	-2.1901	-0.6833
O	0.9892	-4.25826	-0.64907
C	1.09517	-1.76347	-1.63468
C	0.56498	-0.50076	-2.32164
C	2.39787	-1.52258	-0.92756
C	-0.71762	-0.7036	-3.12034
C	-3.18685	2.15557	-1.98003
C	-1.90611	1.50885	-2.55141
C	-1.97637	-0.03884	-2.5996
C	-0.72033	-1.43433	-4.24019
C	-1.6138	2.11051	-3.93416
O	-6.24015	2.2308	1.88059
O	-2.79123	-1.59409	2.60448
C	3.45239	-2.3683	-0.78175
O	4.41864	-1.72471	-0.06632
C	3.89783	-0.48361	0.20163
N	2.69327	-0.32605	-0.28376
C	4.76022	0.40124	0.95939
C	4.58526	1.71597	1.21116
C	3.38569	2.51866	0.72214
C	5.59143	2.46482	2.04648
C	2.30502	2.62682	1.80105
O	3.76402	3.85324	0.37957
C	4.42124	3.94982	-0.86961

H	-4.1991	1.1076	3.15302
H	-5.66016	2.55156	-0.62435
H	-4.38938	3.55849	0.08199
H	-4.0866	0.62287	-0.73759
H	-1.68166	0.69582	3.30046
H	-0.71942	0.96299	1.82314
H	-1.81198	-1.10305	0.83801
H	-0.23263	-1.48153	3.41773
H	0.41365	-1.28501	1.78674
H	-1.23189	-3.61527	2.91035
H	0.47445	-3.66783	2.47836
H	-1.82872	-3.28427	0.50131
H	-1.44555	-5.69588	-0.1029
H	-1.93849	-5.51622	1.59312
H	-0.23698	-5.82426	1.17785
H	1.22222	-2.57464	-2.35485
H	1.3642	-0.14255	-2.98194
H	0.43828	0.2671	-1.5536
H	-3.01368	3.23718	-1.89915
H	-4.0142	2.02946	-2.69325
H	-1.08534	1.78577	-1.8787
H	-2.16474	-0.41276	-1.58465
H	-2.82993	-0.34343	-3.22075
H	-1.62733	-1.59285	-4.81807
H	0.1814	-1.90928	-4.622
H	-0.67152	1.73505	-4.3445
H	-1.5491	3.20384	-3.88262
H	-2.40764	1.8563	-4.64891
H	-3.60503	-1.14689	2.31745
H	3.6453	-3.38786	-1.07193
H	5.64407	-0.09573	1.3522
H	2.95037	2.0238	-0.15461
H	6.38336	1.80293	2.40876
H	6.0491	3.28258	1.47918
H	5.10893	2.93222	2.91448
H	1.47262	3.22859	1.42262
H	1.93556	1.63121	2.06413
H	2.69381	3.111	2.70298
H	4.63401	5.00931	-1.03597
H	5.36896	3.38985	-0.88999
H	3.78595	3.57909	-1.68911

Compound 1a: Isomer 2S, 5R, 11R, 13R, 17S, 23R conformer 2

C	2.9488	-2.13843	-0.37577
C	4.01704	-2.67306	0.27003
C	5.3326	-2.04634	0.23379
C	5.4022	-0.79609	-0.64375
C	4.07484	-0.04111	-0.64365
O	2.97555	-0.92884	-0.98709
C	1.59779	-2.79225	-0.45598
C	0.75144	-2.63079	0.83723
C	0.47851	-1.16091	1.18958
C	-0.22303	-0.9906	2.54639
C	-0.09961	0.41505	3.19514
C	-0.90178	1.48221	2.45947
C	-0.51378	0.38516	4.67151
O	-0.38762	1.73373	1.22545
O	-1.87137	2.06119	2.89818
C	-1.07393	2.71836	0.4164
C	-0.16392	2.99812	-0.79444
C	-2.42761	2.22594	-0.00956
C	1.20277	3.56043	-0.43078
C	4.05273	1.12317	-1.62928
C	2.75435	1.95161	-1.73192
C	2.37953	2.60719	-0.37613
C	1.34491	4.86361	-0.16434
C	2.91825	2.9947	-2.84811
O	6.32174	-2.497	0.79711
O	-0.42754	-3.41587	0.75035
C	-3.60526	2.90032	-0.07592
O	-4.5496	2.05728	-0.59183
C	-3.89149	0.87668	-0.80317
N	-2.62353	0.94071	-0.48993
C	-4.69178	-0.20744	-1.35183
C	-4.45347	-1.53341	-1.2748
C	-3.29185	-2.1187	-0.48552
C	-5.36891	-2.51193	-1.96485
C	-3.74785	-3.13693	0.56034
O	-2.36078	-2.81634	-1.34637
C	-1.81031	-2.01844	-2.3926
H	3.92084	-3.63174	0.76847

H	6.21275	-0.15239	-0.28896
H	5.65418	-1.11	-1.66742
H	3.86988	0.30824	0.37897
H	1.71295	-3.86565	-0.63603
H	1.05219	-2.36045	-1.30342
H	1.32232	-3.08	1.66028
H	-0.10412	-0.68721	0.39104
H	1.44105	-0.63376	1.21789
H	0.22157	-1.69767	3.25979
H	-1.28012	-1.27106	2.46872
H	0.95531	0.71562	3.12234
H	-0.41616	1.37217	5.13313
H	0.11562	-0.32033	5.22412
H	-1.55806	0.07518	4.77834
H	-1.19482	3.62844	1.01226
H	-0.70183	3.71152	-1.42948
H	-0.07321	2.06978	-1.36838
H	4.29121	0.72233	-2.62374
H	4.88348	1.79065	-1.35846
H	1.94806	1.26426	-2.01838
H	2.15116	1.81417	0.34741
H	3.25453	3.15104	0.00588
H	2.30448	5.28958	0.11757
H	0.50681	5.5563	-0.21272
H	1.99923	3.56731	-3.00491
H	3.18492	2.51447	-3.79689
H	3.71201	3.71205	-2.60154
H	-0.93889	-3.17609	-0.05016
H	-3.93268	3.89203	0.19191
H	-5.59087	0.13482	-1.86044
H	-2.75462	-1.30574	0.00791
H	-6.07349	-2.00536	-2.63067
H	-4.78517	-3.23065	-2.55272
H	-5.94831	-3.09838	-1.24069
H	-2.88782	-3.46353	1.15121
H	-4.48317	-2.68297	1.23344
H	-4.19716	-4.01615	0.08898
H	-1.04413	-2.63091	-2.87525
H	-2.57137	-1.7504	-3.13658
H	-1.36373	-1.09435	-2.00479

Compound 1a: Isomer 2S, 5R, 11R, 13R, 17S, 23R conformer 3

C	-2.87036	1.16771	1.53203
C	-4.07766	1.40864	2.09838
C	-5.14858	2.07901	1.36943
C	-4.77235	2.5054	-0.05066
C	-3.72402	1.57659	-0.6597
O	-2.58558	1.4412	0.23402
C	-1.71415	0.49941	2.21724
C	-1.69416	-1.01997	1.91832
C	-0.38722	-1.68783	2.3672
C	-0.40241	-3.22274	2.24226
C	-0.78535	-3.82167	0.86115
C	0.26045	-3.49605	-0.19452
C	-0.97035	-5.34091	0.96363
O	0.13901	-2.22114	-0.64536
O	1.1159	-4.26174	-0.58941
C	1.14208	-1.77825	-1.6116
C	0.56562	-0.54476	-2.31313
C	2.44086	-1.48624	-0.9174
C	-0.71891	-0.80095	-3.09404
C	-3.21619	2.0585	-2.01841
C	-1.92977	1.40906	-2.57201
C	-1.98561	-0.13921	-2.58866
C	-0.71557	-1.57151	-4.18681
C	-1.63691	1.98479	-3.96584
O	-6.24547	2.33263	1.85015
O	-2.84427	-1.6651	2.46477
C	3.52868	-2.29026	-0.78125
O	4.47647	-1.60888	-0.07602
C	3.91053	-0.38852	0.19595
N	2.69588	-0.27856	-0.27716
C	4.74606	0.53084	0.94273
C	4.52567	1.83995	1.18811
C	3.2942	2.59577	0.7031
C	5.51069	2.63068	2.00975
C	2.21933	2.67486	1.79036
O	3.62247	3.93977	0.34664
C	4.26454	4.04884	-0.90952
H	-4.25693	1.13183	3.13076
H	-5.67544	2.53124	-0.66838

H	-4.37713	3.53096	-0.00497
H	-4.15186	0.56722	-0.74656
H	-1.77254	0.65874	3.30106
H	-0.78	0.95021	1.86114
H	-1.79724	-1.14926	0.83768
H	-0.19477	-1.43554	3.42212
H	0.4379	-1.25448	1.79112
H	-1.12916	-3.62156	2.95883
H	0.5786	-3.61648	2.5393
H	-1.73004	-3.36584	0.54623
H	-1.25234	-5.77223	-0.00197
H	-1.75811	-5.57489	1.68732
H	-0.04381	-5.82719	1.28381
H	1.28975	-2.59716	-2.31885
H	1.34602	-0.17155	-2.98774
H	0.42229	0.23077	-1.55596
H	-3.04674	3.14232	-1.96198
H	-4.03954	1.91442	-2.7329
H	-1.11391	1.7068	-1.9022
H	-2.17402	-0.49222	-1.56633
H	-2.83452	-0.46574	-3.20479
H	-1.62341	-1.76565	-4.75228
H	0.19163	-2.04458	-4.55793
H	-0.68996	1.60948	-4.36584
H	-1.58182	3.07949	-3.93639
H	-2.42604	1.70953	-4.67801
H	-2.7839	-1.61029	3.43287
H	3.75856	-3.3018	-1.07279
H	5.65078	0.06855	1.33039
H	2.86939	2.07839	-0.16569
H	6.32963	2.001	2.36943
H	5.93257	3.46076	1.43279
H	5.01849	3.08611	2.87864
H	1.36283	3.24315	1.41407
H	1.88785	1.66893	2.06472
H	2.59815	3.18036	2.68485
H	3.6348	3.64991	-1.71995
H	4.43921	5.11362	-1.08597
H	5.23069	3.52151	-0.93429

Compound 1a: Isomer 2S, 5R, 11R, 13R, 17S, 23R conformer 4

C	-1.12248	1.99234	1.33796
C	-1.37007	3.18039	1.94856
C	-1.94065	4.31122	1.21884
C	-2.18873	4.03327	-0.26463
C	-2.57052	2.57142	-0.48512
O	-1.55097	1.70003	0.08449
C	-0.37562	0.84412	1.95106
C	-1.30011	-0.34443	2.29873
C	-0.49765	-1.59421	2.66577
C	-1.32005	-2.89226	2.7257
C	-2.05052	-3.31722	1.42051
C	-1.08794	-3.39479	0.24091
C	-2.76415	-4.66137	1.61113
O	-0.9048	-2.17197	-0.31979
O	-0.53898	-4.40126	-0.15707
C	0.02887	-2.07008	-1.43834
C	-0.28604	-0.75272	-2.16722
C	1.44953	-2.1302	-0.95415
C	-1.64925	-0.71049	-2.83106
C	-2.75609	2.20035	-1.95854
C	-3.57957	0.93259	-2.29595
C	-2.87575	-0.41105	-1.97581
C	-1.76494	-0.93696	-4.14521
C	-4.98016	0.96629	-1.66189
O	-2.15163	5.40727	1.71943
O	-2.14486	-0.04416	3.41035
C	2.26127	-3.21738	-0.86696
O	3.47263	-2.80784	-0.39615
C	3.34718	-1.45335	-0.21145
N	2.15401	-1.01188	-0.51841
C	4.54769	-0.79191	0.26419
C	4.80917	0.5299	0.34695
C	3.82253	1.62063	-0.05171
C	6.14392	0.99904	0.86728
C	3.21265	2.30988	1.17123
O	4.4724	2.64278	-0.81214
C	4.76276	2.25969	-2.14327
H	-1.03515	3.34494	2.96679
H	-2.9785	4.69881	-0.62523
H	-1.27198	4.27287	-0.82335
H	-3.48919	2.37012	0.08429

H	0.1401	1.17834	2.85694
H	0.37713	0.50518	1.22837
H	-1.90871	-0.55188	1.41024
H	-0.04231	-1.42294	3.64993
H	0.32377	-1.70451	1.9508
H	-2.08389	-2.79712	3.50601
H	-0.6568	-3.71299	3.02913
H	-2.79117	-2.54689	1.17813
H	-3.33887	-4.93857	0.72119
H	-3.45375	-4.60524	2.46012
H	-2.04069	-5.45977	1.80096
H	-0.15959	-2.92474	-2.09302
H	0.49503	-0.62304	-2.92499
H	-0.17716	0.06774	-1.45091
H	-1.7652	2.14197	-2.42639
H	-3.26478	3.05262	-2.42865
H	-3.71818	0.96352	-3.38552
H	-2.60911	-0.43997	-0.91739
H	-3.61327	-1.20991	-2.13155
H	-2.73033	-0.93523	-4.64719
H	-0.89935	-1.13531	-4.77314
H	-5.60947	0.17029	-2.07571
H	-5.4849	1.92197	-1.84988
H	-4.94394	0.81563	-0.57602
H	-2.65052	0.75584	3.19293
H	2.13039	-4.27029	-1.05478
H	5.32047	-1.49458	0.56752
H	3.01625	1.17797	-0.64861
H	6.80396	0.15834	1.09897
H	6.639	1.65038	0.13926
H	6.0216	1.59799	1.77915
H	2.48837	3.06308	0.84624
H	2.70328	1.58015	1.80768
H	3.98464	2.81361	1.76165
H	5.21137	3.12853	-2.63236
H	5.47124	1.41855	-2.19215
H	3.85017	1.97264	-2.68851

Compound 1a: Isomer 2S, 5R, 11R, 13R, 17S, 23R conformer 6

C	-5.15153	-0.2362	0.5308
C	-5.91413	-0.42719	-0.57305

C	-6.08427	0.61384	-1.58352
C	-5.37976	1.92864	-1.24948
C	-4.09164	1.68556	-0.46939
O	-4.35931	0.85925	0.70686
C	-5.0998	-1.16864	1.70886
C	-3.69398	-1.71412	2.06036
C	-3.01154	-2.43301	0.88571
C	-1.7395	-3.17629	1.31829
C	-0.85681	-3.65282	0.14439
C	-0.41047	-2.46565	-0.69849
C	0.35728	-4.45884	0.64128
O	0.26832	-1.56642	0.05216
O	-0.64829	-2.31431	-1.87996
C	0.62049	-0.30233	-0.58905
C	0.55113	0.76106	0.51941
C	1.95851	-0.42314	-1.25244
C	0.41286	2.16895	-0.0292
C	-3.4282	2.96876	0.02565
C	-2.00445	2.82799	0.60657
C	-0.96199	2.58396	-0.51729
C	1.46173	2.99633	-0.09055
C	-1.64658	4.07024	1.43418
O	-6.77996	0.48588	-2.58096
O	-2.86472	-0.68886	2.60382
C	2.22134	-0.74673	-2.54657
O	3.5746	-0.79429	-2.70696
C	4.08982	-0.49193	-1.47071
N	3.16169	-0.27202	-0.57586
C	5.53651	-0.49163	-1.38611
C	6.30807	-0.12318	-0.34141
C	5.75159	0.37149	0.98842
C	7.80792	-0.23335	-0.43682
C	5.62356	-0.76845	2.00218
O	6.6078	1.35571	1.57192
C	6.48269	2.6335	0.9794
H	-6.49261	-1.33786	-0.67904
H	-5.16949	2.46881	-2.17728
H	-6.0675	2.54759	-0.65484
H	-3.40611	1.0976	-1.09642
H	-5.78475	-2.00139	1.52079
H	-5.47032	-0.63085	2.59206

H	-3.83059	-2.43229	2.87924
H	-2.77357	-1.68624	0.11557
H	-3.71806	-3.13455	0.42025
H	-2.01739	-4.05416	1.91785
H	-1.14726	-2.52163	1.96531
H	-1.45039	-4.27687	-0.53348
H	0.97859	-4.80004	-0.19405
H	0.02045	-5.34272	1.1941
H	0.98052	-3.85362	1.30685
H	-0.13239	-0.12135	-1.35977
H	1.4436	0.67494	1.14465
H	-0.31187	0.50343	1.14496
H	-4.09289	3.40755	0.78115
H	-3.40371	3.6779	-0.81351
H	-1.99689	1.96385	1.28459
H	-1.34665	1.81168	-1.19933
H	-0.87816	3.50222	-1.11355
H	1.37496	4.00041	-0.49943
H	2.44569	2.69367	0.25768
H	-0.62778	4.00306	1.82725
H	-2.3322	4.19042	2.28071
H	-1.70915	4.97903	0.82087
H	-2.98907	0.09205	2.03842
H	1.61111	-0.98368	-3.40305
H	6.01739	-0.84805	-2.2939
H	4.75559	0.8019	0.82731
H	8.117	-0.66471	-1.39337
H	8.28871	0.74427	-0.32161
H	8.205	-0.86215	0.37028
H	5.25979	-0.36833	2.9537
H	4.91414	-1.51683	1.63798
H	6.59111	-1.24943	2.18053
H	7.15779	3.30304	1.51942
H	6.76077	2.63235	-0.08602
H	5.45404	3.01844	1.06449

Compound 1a: Isomer 2S, 5R, 11R, 13R, 17S, 23R conformer 11

C	-2.81354	1.11231	1.56476
C	-4.04488	1.24737	2.126
C	-5.15354	1.87319	1.40653

C	-4.79224	2.37021	0.00584
C	-3.69088	1.51766	-0.61945
O	-2.54825	1.43119	0.27612
C	-1.63294	0.47469	2.23501
C	-1.63085	-1.04742	1.9332
C	-0.33494	-1.72979	2.36883
C	-0.36014	-3.26096	2.20681
C	-0.74987	-3.82247	0.81137
C	0.29806	-3.48352	-0.23817
C	-0.95074	-5.34183	0.87799
O	0.18262	-2.20188	-0.67355
O	1.15251	-4.24615	-0.6416
C	1.19034	-1.75197	-1.63264
C	0.6217	-0.50889	-2.32443
C	2.49027	-1.47151	-0.93492
C	-0.66131	-0.75119	-3.11124
C	-3.2027	2.04295	-1.96749
C	-1.90474	1.42966	-2.53677
C	-1.93406	-0.11942	-2.58331
C	-0.65319	-1.48659	-4.22798
C	-1.62658	2.03755	-3.91975
O	-6.26771	2.03381	1.88476
O	-2.70725	-1.69736	2.60404
C	3.55749	-2.29719	-0.76879
O	4.51573	-1.61988	-0.07364
C	3.97777	-0.37953	0.16094
N	2.76985	-0.25262	-0.3261
C	4.82815	0.54084	0.88717
C	4.62689	1.85736	1.10677
C	3.3944	2.60055	0.62367
C	5.63215	2.65765	1.89081
C	2.26795	2.53268	1.669
O	3.78828	3.95122	0.36503
C	2.93508	4.64388	-0.52126
H	-4.20096	0.96455	3.16169
H	-5.69038	2.36803	-0.61936
H	-4.4525	3.41293	0.08998
H	-4.05985	0.48659	-0.72492
H	-1.67521	0.62187	3.31862
H	-0.71323	0.92819	1.84896
H	-1.73545	-1.16564	0.84512

H	-0.15311	-1.50398	3.42837
H	0.48904	-1.28782	1.79816
H	-1.08668	-3.66684	2.91799
H	0.62084	-3.6667	2.48772
H	-1.69008	-3.34955	0.5078
H	-1.23882	-5.74881	-0.09656
H	-1.73834	-5.58397	1.59905
H	-0.02839	-5.84396	1.18525
H	1.33481	-2.56396	-2.34849
H	1.40605	-0.13483	-2.99366
H	0.4812	0.2614	-1.5613
H	-3.05857	3.12894	-1.88733
H	-4.0255	1.89447	-2.68165
H	-1.09224	1.72823	-1.8632
H	-2.1067	-0.49725	-1.56695
H	-2.78287	-0.44704	-3.19923
H	-1.56028	-1.67252	-4.79749
H	0.25813	-1.93823	-4.61518
H	-0.67319	1.68839	-4.32753
H	-1.59336	3.13245	-3.86987
H	-2.41119	1.75987	-4.63588
H	-3.53347	-1.27479	2.31534
H	3.76525	-3.32095	-1.03256
H	5.73189	0.07793	1.27508
H	3.03455	2.13821	-0.30447
H	6.45104	2.02809	2.25071
H	6.04251	3.46402	1.27427
H	5.1609	3.14396	2.75481
H	1.39114	3.09619	1.33153
H	1.96428	1.49307	1.82231
H	2.5989	2.95413	2.62412
H	3.38632	5.62541	-0.69073
H	2.84384	4.12202	-1.48725
H	1.9247	4.79115	-0.11103

Compound 1a: Isomer 2S, 5R, 11R, 13R, 17S, 23R conformer 12

C	-2.86076	1.05832	1.54703
C	-4.07548	1.2589	2.1131
C	-5.16813	1.89316	1.38392
C	-4.8075	2.32877	-0.03754

C	-3.72823	1.43478	-0.64465
O	-2.58529	1.34203	0.24887
C	-1.68375	0.42704	2.23181
C	-1.61508	-1.09041	1.92952
C	-0.28755	-1.71744	2.37663
C	-0.25457	-3.25175	2.25031
C	-0.61631	-3.86083	0.8682
C	0.41963	-3.50129	-0.18657
C	-0.7529	-5.38538	0.96878
O	0.25923	-2.23075	-0.6371
O	1.29815	-4.24055	-0.58142
C	1.24433	-1.76067	-1.61048
C	0.62447	-0.55203	-2.31806
C	2.53604	-1.42164	-0.92436
C	-0.65765	-0.85409	-3.08628
C	-3.23836	1.92883	-2.00546
C	-1.9332	1.31846	-2.55948
C	-1.94094	-0.23092	-2.57336
C	-0.63911	-1.6299	-4.17513
C	-1.65938	1.90039	-3.95466
O	-6.27215	2.11202	1.86525
O	-2.744	-1.77277	2.47465
C	3.63864	-2.20031	-0.76274
O	4.57184	-1.48023	-0.07642
C	3.9828	-0.26317	0.1577
N	2.76723	-0.18987	-0.32119
C	4.79767	0.69565	0.87483
C	4.54542	2.00523	1.08323
C	3.28484	2.69587	0.59447
C	5.51944	2.85053	1.85936
C	2.16256	2.59539	1.64173
O	3.62682	4.05769	0.322
C	2.74556	4.70951	-0.56821
H	-4.24581	0.97583	3.14529
H	-5.7113	2.322	-0.65469
H	-4.44791	3.36752	0.00531
H	-4.121	0.41102	-0.72779
H	-1.74789	0.58206	3.31592
H	-0.76416	0.90817	1.87753
H	-1.71401	-1.22039	0.84856
H	-0.10143	-1.45969	3.43131

H	0.52301	-1.25898	1.79938
H	-0.96898	-3.67427	2.9657
H	0.73813	-3.6143	2.54828
H	-1.57485	-3.43566	0.55204
H	-1.02207	-5.82397	0.0028
H	-1.53218	-5.64527	1.69283
H	0.18892	-5.84254	1.28722
H	1.41483	-2.57954	-2.31252
H	1.38789	-0.16116	-3.00199
H	0.46335	0.22582	-1.56703
H	-3.1033	3.01773	-1.95245
H	-4.05805	1.75683	-2.71795
H	-1.12627	1.64202	-1.89076
H	-2.1119	-0.58809	-1.54935
H	-2.78322	-0.58455	-3.18358
H	-1.54511	-1.85622	-4.73155
H	0.27962	-2.07567	-4.55157
H	-0.70048	1.55566	-4.35359
H	-1.6409	2.99647	-3.92817
H	-2.43866	1.59727	-4.66627
H	-2.68378	-1.72064	3.44291
H	3.88862	-3.21509	-1.0248
H	5.71981	0.27201	1.26441
H	2.94208	2.21081	-0.32851
H	6.36236	2.25614	2.22355
H	5.89783	3.66712	1.23586
H	5.0301	3.32532	2.71969
H	1.26461	3.12151	1.29975
H	1.89906	1.54635	1.80494
H	2.47811	3.03853	2.5923
H	3.15972	5.70526	-0.74889
H	2.67074	4.17491	-1.52856
H	1.73187	4.82321	-0.15576

Compound 1a: Isomer 2S, 5R, 11R, 13R, 17S, 23R conformer 13

C	-2.99356	-1.72242	-1.32555
C	-4.26873	-1.74369	-1.79086
C	-5.41559	-1.78886	-0.89134
C	-5.04397	-1.88266	0.58922
C	-3.73014	-1.16218	0.88487

O	-2.67905	-1.61576	-0.01181
C	-1.77211	-1.75769	-2.20137
C	-1.44323	-0.38718	-2.85437
C	-1.27513	0.73248	-1.8241
C	-1.00801	2.10695	-2.45306
C	-1.1328	3.3155	-1.48638
C	0.00779	3.37874	-0.47506
C	-1.22242	4.63958	-2.25386
O	0.02084	2.2883	0.33258
O	0.81115	4.28308	-0.38119
C	1.09935	2.20597	1.31168
C	0.6704	1.17026	2.36013
C	2.38841	1.83565	0.63809
C	-0.64487	1.49085	3.0556
C	-3.24471	-1.37818	2.31587
C	-1.89435	-0.74919	2.72219
C	-1.89968	0.794	2.56569
C	-0.67976	2.36453	4.06775
C	-1.55997	-1.17778	4.15914
O	-6.57981	-1.81114	-1.2687
O	-0.30161	-0.51762	-3.69858
C	3.4142	2.64696	0.26793
O	4.37623	1.87799	-0.31679
C	3.88138	0.59865	-0.28051
N	2.69666	0.53048	0.27067
C	4.73981	-0.41613	-0.85831
C	4.59133	-1.75689	-0.80422
C	3.43517	-2.44961	-0.09281
C	5.57923	-2.6543	-1.50397
C	2.30002	-2.78466	-1.06415
O	3.85234	-3.6819	0.49519
C	4.57053	-3.51575	1.70329
H	-4.45318	-1.79985	-2.85843
H	-5.85739	-1.46589	1.19076
H	-4.95274	-2.94706	0.85092
H	-3.8606	-0.0903	0.67461
H	-1.90482	-2.4857	-3.00756
H	-0.91762	-2.07733	-1.59291
H	-2.2608	-0.13127	-3.53904
H	-0.47084	0.46275	-1.13068
H	-2.18962	0.7892	-1.21987

H	-1.73938	2.2709	-3.25603
H	-0.02476	2.11925	-2.93882
H	-2.05435	3.17522	-0.90255
H	-1.31288	5.48958	-1.57176
H	-2.09357	4.63096	-2.91735
H	-0.32635	4.80005	-2.86181
H	1.20031	3.19433	1.7666
H	1.48016	1.12218	3.09791
H	0.63583	0.19116	1.87364
H	-3.189	-2.46151	2.48889
H	-4.03272	-0.99964	2.98299
H	-1.12765	-1.16175	2.05423
H	-2.04186	1.03875	1.50526
H	-2.76335	1.20111	3.10955
H	-1.60821	2.62189	4.57135
H	0.21824	2.86117	4.43016
H	-0.57947	-0.80736	4.47311
H	-1.55487	-2.27017	4.25121
H	-2.30008	-0.78578	4.86923
H	0.47081	-0.65225	-3.12449
H	3.58788	3.7096	0.31149
H	5.59284	-0.00316	-1.39138
H	3.03827	-1.78629	0.68547
H	6.33773	-2.0742	-2.03722
H	6.08132	-3.32317	-0.79665
H	5.07167	-3.30533	-2.22736
H	1.50323	-3.30919	-0.52742
H	1.89384	-1.86223	-1.49089
H	2.64931	-3.42987	-1.87668
H	4.80488	-4.51723	2.07385
H	5.51049	-2.96106	1.55954
H	3.97036	-2.98507	2.45867

Compound 1a: Isomer 2S, 5R, 11R, 13R, 17S, 23R conformer 16

C	-3.01764	1.78456	1.03523
C	-4.12859	2.35154	1.57267
C	-5.28349	2.69913	0.74194
C	-5.11485	2.37747	-0.7466
C	-4.21571	1.15859	-0.93882
O	-2.95329	1.37224	-0.2522

C	-1.75806	1.43517	1.77301
C	-1.72921	-0.08525	2.06846
C	-0.3554	-0.58209	2.51522
C	-0.32924	-2.08548	2.84433
C	-0.76114	-3.06615	1.71711
C	0.27933	-3.15053	0.60817
C	-1.02098	-4.46532	2.29031
O	0.26993	-2.05263	-0.19466
O	1.04281	-4.08034	0.44609
C	1.27815	-2.03491	-1.25491
C	0.84178	-1.01501	-2.31634
C	2.62296	-1.6991	-0.67837
C	-0.55735	-1.2142	-2.88345
C	-3.90937	0.82215	-2.39661
C	-3.0685	-0.45039	-2.68755
C	-1.59149	-0.2851	-2.27911
C	-0.7816	-2.10132	-3.85795
C	-3.70098	-1.7129	-2.07858
O	-6.289	3.24267	1.17652
O	-2.66525	-0.41802	3.09152
C	3.6473	-2.53463	-0.3623
O	4.66727	-1.78524	0.14605
C	4.20626	-0.49272	0.12207
N	2.99162	-0.39864	-0.35372
C	5.12528	0.50621	0.62969
C	4.99775	1.84943	0.5819
C	3.80534	2.56338	-0.04388
C	6.05043	2.72941	1.205
C	2.75093	2.9221	1.00645
O	4.20465	3.78559	-0.66683
C	4.82823	3.5999	-1.92297
H	-4.14063	2.64593	2.61655
H	-6.10228	2.21462	-1.18872
H	-4.66982	3.25301	-1.24167
H	-4.68781	0.29819	-0.44176
H	-1.69071	1.99017	2.71358
H	-0.89974	1.70157	1.1445
H	-1.99683	-0.59908	1.13382
H	-0.05685	-0.02822	3.41542
H	0.36952	-0.34499	1.72916
H	-1.00442	-2.25943	3.68847

H	0.67765	-2.36249	3.18368
H	-1.68717	-2.68952	1.26772
H	-1.32168	-5.16831	1.50731
H	-1.81807	-4.41923	3.0395
H	-0.11948	-4.86624	2.76357
H	1.3089	-3.04001	-1.6812
H	1.58494	-1.07926	-3.12041
H	0.93689	-0.01333	-1.88465
H	-3.42222	1.69275	-2.85593
H	-4.88178	0.71197	-2.89597
H	-3.09793	-0.55716	-3.78152
H	-1.27099	0.74189	-2.51534
H	-1.50203	-0.35349	-1.19018
H	-1.76496	-2.2666	-4.28751
H	0.02462	-2.70426	-4.27033
H	-3.1585	-2.61152	-2.38565
H	-4.74699	-1.82701	-2.38897
H	-3.67769	-1.68271	-0.98238
H	-3.53518	-0.08243	2.81817
H	3.78806	-3.60233	-0.39935
H	6.00625	0.07824	1.10205
H	3.34028	1.90651	-0.78913
H	6.83501	2.13649	1.68387
H	6.51228	3.38803	0.46142
H	5.60788	3.39075	1.96097
H	1.93064	3.46518	0.52617
H	2.35518	2.01243	1.46736
H	3.17259	3.56286	1.78804
H	5.0586	4.59457	-2.31422
H	5.763	3.02317	-1.84655
H	4.16244	3.08185	-2.63079

Compound 1a: Isomer 2S, 5R, 11R, 13R, 17S, 23R conformer 21

C	-5.34662	0.66707	0.27596
C	-5.75507	0.7912	1.56222
C	-5.80335	-0.35161	2.471
C	-5.41017	-1.67983	1.82467
C	-4.35122	-1.47853	0.74576
O	-4.79673	-0.47686	-0.22212
C	-5.47385	1.73834	-0.7712

C	-4.15073	2.15617	-1.45814
C	-3.07683	2.63857	-0.4694
C	-1.87096	3.26296	-1.18591
C	-0.64413	3.49755	-0.27826
C	-0.165	2.18064	0.31765
C	0.49515	4.19912	-1.04019
O	0.14695	1.29332	-0.65471
O	-0.09361	1.93247	1.50506
C	0.46193	-0.06916	-0.23024
C	-0.02193	-0.98262	-1.36749
C	1.92633	-0.1872	0.0733
C	-0.2196	-2.41934	-0.92069
C	-4.02975	-2.7485	-0.03913
C	-2.79067	-2.69182	-0.95851
C	-1.47788	-2.72611	-0.13172
C	0.68644	-3.3626	-1.19964
C	-2.83476	-3.83946	-1.97698
O	-6.18606	-0.2822	3.63031
O	-3.65988	1.11826	-2.30362
C	2.55491	0.04987	1.25536
O	3.89518	-0.14896	1.07018
C	4.02151	-0.49921	-0.24986
N	2.87444	-0.53694	-0.8764
C	5.31033	-0.81428	-0.83185
C	6.55387	-0.68203	-0.32313
C	6.87065	-0.13574	1.06639
C	7.74282	-1.12749	-1.1377
C	7.05892	-1.26064	2.08811
O	8.08273	0.6196	1.05713
C	7.93766	1.91221	0.49993
H	-6.1457	1.73869	1.91574
H	-5.04296	-2.36061	2.59828
H	-6.31212	-2.13228	1.38714
H	-3.44853	-1.05953	1.21299
H	-5.94811	2.61284	-0.31474
H	-6.14693	1.37256	-1.5585
H	-4.39655	2.9831	-2.13696
H	-2.75762	1.78265	0.1415
H	-3.51687	3.36334	0.23002
H	-2.16679	4.22853	-1.61897
H	-1.57878	2.61549	-2.01872

H	-0.93355	4.11198	0.582
H	1.36006	4.37466	-0.39108
H	0.15298	5.1695	-1.41653
H	0.82328	3.59605	-1.89248
H	-0.10008	-0.24603	0.68991
H	0.69736	-0.93012	-2.18841
H	-0.96555	-0.55759	-1.73007
H	-4.91923	-2.99808	-0.63209
H	-3.90112	-3.56564	0.68446
H	-2.8256	-1.74781	-1.51907
H	-1.5615	-2.00912	0.69819
H	-1.38795	-3.71825	0.33028
H	0.55973	-4.39089	-0.86854
H	1.59137	-3.13315	-1.75543
H	-1.94924	-3.83216	-2.61961
H	-3.72186	-3.7657	-2.61643
H	-2.86919	-4.81165	-1.46739
H	-3.74202	0.29175	-1.7983
H	2.23113	0.36033	2.23541
H	5.19819	-1.21775	-1.83492
H	6.05248	0.51086	1.40505
H	7.4315	-1.56675	-2.08968
H	8.42289	-0.29347	-1.34236
H	8.3345	-1.87342	-0.59171
H	7.31404	-0.83038	3.06156
H	6.13805	-1.8429	2.18675
H	7.8703	-1.93166	1.78805
H	8.91313	2.40059	0.57212
H	7.6322	1.88142	-0.55715
H	7.1967	2.50896	1.05466

Compound 1a: Isomer 2S, 5R, 11R, 13R, 17S, 23R conformer 22

C	2.91005	-1.90614	1.02439
C	4.14748	-2.0041	1.57422
C	5.35854	-1.83577	0.7791
C	5.09984	-1.61516	-0.71192
C	3.78819	-0.86918	-0.94844
O	2.68999	-1.52469	-0.25567
C	1.62748	-2.15521	1.76924
C	1.20617	-0.97354	2.68392

C	1.04596	0.33443	1.91594
C	0.70945	1.53446	2.81291
C	0.89212	2.92646	2.14928
C	-0.16696	3.2103	1.08912
C	0.8991	4.04943	3.19283
O	-0.08668	2.3306	0.05956
O	-0.9842	4.10548	1.13656
C	-1.10044	2.43816	-0.9829
C	-0.57736	1.65014	-2.19226
C	-2.41413	1.91403	-0.48414
C	0.76832	2.1308	-2.71559
C	3.4143	-0.775	-2.42494
C	2.07351	-0.09979	-2.78543
C	2.00997	1.3702	-2.29265
C	0.84027	3.19543	-3.52216
C	1.85702	-0.20462	-4.30284
O	6.49145	-1.92008	1.23485
O	-0.05016	-1.2588	3.29975
C	-3.51029	2.6139	-0.09053
O	-4.4658	1.72388	0.29912
C	-3.89245	0.48699	0.12927
N	-2.67053	0.55688	-0.33109
C	-4.74142	-0.63108	0.4926
C	-4.49862	-1.95235	0.36304
C	-3.21063	-2.52975	-0.21057
C	-5.52984	-2.95264	0.81949
C	-2.27814	-3.04566	0.88821
O	-3.48888	-3.6425	-1.06692
C	-4.00757	-3.26702	-2.32828
H	4.2563	-2.28554	2.6163
H	5.9408	-1.065	-1.14464
H	5.06353	-2.59943	-1.20165
H	3.86769	0.13253	-0.50165
H	1.73688	-3.05323	2.39033
H	0.82447	-2.33767	1.04892
H	1.98081	-0.84407	3.4583
H	0.27865	0.19766	1.14935
H	1.98744	0.53613	1.39054
H	1.37389	1.51768	3.68817
H	-0.31002	1.43809	3.2038
H	1.85914	2.91683	1.62518

H	1.0282	5.02843	2.72263
H	1.7157	3.89561	3.90634
H	-0.04465	4.07371	3.74698
H	-1.2035	3.49763	-1.23069
H	-1.33802	1.74366	-2.97643
H	-0.54367	0.59213	-1.91595
H	3.41041	-1.79427	-2.83433
H	4.2333	-0.24267	-2.93008
H	1.2778	-0.66614	-2.28528
H	2.06356	1.37727	-1.19649
H	2.89531	1.9051	-2.66368
H	1.79044	3.56866	-3.8961
H	-0.04823	3.7396	-3.83661
H	0.88742	0.20545	-4.60035
H	1.90036	-1.24886	-4.63411
H	2.62917	0.35245	-4.84984
H	0.05839	-2.04092	3.86331
H	-3.7434	3.66214	0.00083
H	-5.68702	-0.31542	0.92714
H	-2.68681	-1.7531	-0.77981
H	-6.41428	-2.45832	1.23161
H	-5.84001	-3.60199	-0.00628
H	-5.11855	-3.61703	1.59037
H	-1.39644	-3.49756	0.42141
H	-1.953	-2.23321	1.5449
H	-2.76998	-3.81643	1.49134
H	-4.1404	-4.18712	-2.90407
H	-4.97908	-2.75536	-2.24735
H	-3.3145	-2.60311	-2.86826

Compound 1a: Isomer 2S, 5R, 11R, 13R, 17S, 23R conformer 29

C	-5.53223	0.55021	-0.23423
C	-6.17648	0.76053	0.93877
C	-6.27878	-0.27716	1.9606
C	-5.66301	-1.6172	1.5595
C	-4.46662	-1.44036	0.62839
O	-4.82338	-0.5825	-0.50296
C	-5.50887	1.51361	-1.38876
C	-4.09103	2.01296	-1.76829
C	-3.35485	2.64243	-0.57625
C	-1.98102	3.21729	-0.95398

C	-1.00013	3.33732	0.24508
C	-0.65898	1.9277	0.7064
C	0.2316	4.17873	-0.1148
O	0.39312	1.41522	0.02809
O	-1.29626	1.30296	1.53474
C	0.68672	0.00209	0.24508
C	0.06317	-0.80365	-0.91549
C	2.17214	-0.14182	0.34021
C	-0.13008	-2.2609	-0.54223
C	-3.97055	-2.76067	0.04188
C	-2.65359	-2.71487	-0.763
C	-1.42147	-2.60018	0.17613
C	0.80434	-3.17972	-0.81023
C	-2.54936	-3.95035	-1.66833
O	-6.86055	-0.12619	3.02591
O	-3.32585	0.96738	-2.36717
C	2.92675	-0.3695	1.44681
O	4.23912	-0.42352	1.06474
C	4.21837	-0.21469	-0.29124
N	3.00965	-0.05166	-0.76136
C	5.43445	-0.21127	-1.07884
C	6.72723	-0.25097	-0.6919
C	7.19855	-0.31694	0.75837
C	7.81559	-0.27287	-1.736
C	7.49406	-1.75572	1.19182
O	8.40537	0.42397	0.94208
C	8.20612	1.82413	0.99208
H	-6.69361	1.69762	1.11125
H	-5.3663	-2.158	2.4631
H	-6.44101	-2.21345	1.06009
H	-3.6672	-0.90828	1.16306
H	-6.15185	2.36545	-1.14618
H	-5.93234	1.01758	-2.27207
H	-4.21319	2.76497	-2.55841
H	-3.23613	1.87895	0.20085
H	-3.98252	3.42884	-0.13393
H	-2.10088	4.21507	-1.39654
H	-1.52036	2.58431	-1.72098
H	-1.53095	3.79895	1.08616
H	0.93614	4.23839	0.72173
H	-0.07748	5.19843	-0.37038

H	0.76242	3.75397	-0.97133
H	0.22978	-0.27894	1.19584
H	0.70625	-0.69756	-1.79353
H	-0.89955	-0.33902	-1.15779
H	-4.77529	-3.15165	-0.59443
H	-3.85886	-3.47362	0.87075
H	-2.67831	-1.83024	-1.41364
H	-1.61873	-1.82843	0.93386
H	-1.3124	-3.55035	0.71607
H	0.68755	-4.21808	-0.5086
H	1.71992	-2.92414	-1.33749
H	-1.60733	-3.95477	-2.22444
H	-3.3726	-3.97775	-2.39136
H	-2.59401	-4.87383	-1.07557
H	-3.4847	0.1724	-1.83026
H	2.71956	-0.51489	2.49528
H	5.20944	-0.17157	-2.14147
H	6.42586	0.09538	1.41791
H	7.39776	-0.28555	-2.7466
H	8.47823	0.59421	-1.64124
H	8.45505	-1.15693	-1.61703
H	7.85671	-1.75818	2.22441
H	6.58783	-2.36563	1.13115
H	8.26513	-2.20555	0.55807
H	9.18545	2.27828	1.16482
H	7.78929	2.22226	0.05435
H	7.5308	2.10549	1.8153

Compound 1a: Isomer 2S, 5R, 11R, 13R, 17S, 23R conformer 31

C	-3.08183	2.18749	-0.30501
C	-4.13593	2.97274	0.04557
C	-5.4724	2.75888	-0.50705
C	-5.55898	1.61076	-1.51389
C	-4.53721	0.51995	-1.20373
O	-3.20484	1.09104	-1.08883
C	-1.68775	2.34198	0.2267
C	-1.54022	1.52617	1.53882
C	-0.09238	1.46015	2.02154
C	0.07733	0.7263	3.3647
C	-0.50403	-0.71198	3.46536

C	0.21782	-1.65915	2.51939
C	-0.41033	-1.2342	4.90449
O	-0.25656	-1.55712	1.24909
O	1.12426	-2.40373	2.83032
C	0.44821	-2.32685	0.23101
C	-0.47077	-2.39315	-0.99437
C	1.76023	-1.67338	-0.09054
C	-1.81768	-3.06278	-0.74715
C	-4.49485	-0.58969	-2.25367
C	-3.28748	-1.55137	-2.21857
C	-3.04976	-2.18339	-0.8235
C	-1.89884	-4.37141	-0.48523
C	-3.46483	-2.62245	-3.30531
O	-6.43959	3.45615	-0.2336
O	-2.31694	2.0996	2.58735
C	3.01405	-2.07076	0.24659
O	3.90033	-1.16603	-0.27084
C	3.12278	-0.22839	-0.89986
N	1.84446	-0.49571	-0.82202
C	3.70648	0.9057	-1.58682
C	4.9842	1.34074	-1.62246
C	6.15078	0.67286	-0.89917
C	5.33746	2.54767	-2.45522
C	6.92029	-0.27381	-1.82511
O	7.088	1.64284	-0.43116
C	6.66314	2.32156	0.73591
H	-3.97815	3.83409	0.68602
H	-6.57553	1.20563	-1.51173
H	-5.37051	2.02093	-2.51688
H	-4.76124	0.0973	-0.21297
H	-1.46809	3.39363	0.43607
H	-0.97475	1.96712	-0.51584
H	-1.88383	0.50335	1.32849
H	0.28351	2.48497	2.14523
H	0.50736	0.98939	1.23538
H	-0.41462	1.31708	4.14427
H	1.14501	0.68849	3.61895
H	-1.55622	-0.67508	3.16277
H	-0.83907	-2.2378	4.99314
H	-0.95372	-0.5673	5.58193
H	0.63262	-1.29061	5.23048

H	0.63144	-3.32321	0.64008
H	0.08131	-2.93533	-1.77209
H	-0.59815	-1.3723	-1.36497
H	-4.52988	-0.12856	-3.25002
H	-5.4277	-1.16205	-2.14775
H	-2.40273	-0.9543	-2.47012
H	-2.93818	-1.37738	-0.08664
H	-3.93371	-2.76845	-0.53467
H	-2.85239	-4.86026	-0.30235
H	-1.01624	-5.0063	-0.4364
H	-2.59498	-3.28389	-3.36024
H	-3.60617	-2.16449	-4.29143
H	-4.34255	-3.24904	-3.09884
H	-3.24175	2.12561	2.28966
H	3.42304	-2.88611	0.82064
H	2.95325	1.45423	-2.14657
H	5.77731	0.09862	-0.04326
H	4.4671	2.92718	-2.99786
H	5.74418	3.35442	-1.83579
H	6.11952	2.30337	-3.18531
H	7.76614	-0.7069	-1.28232
H	6.26859	-1.08135	-2.17175
H	7.31128	0.26204	-2.69604
H	7.46669	3.00867	1.01386
H	5.74082	2.89992	0.57252
H	6.48736	1.62128	1.56739

Compound 1a: Isomer 2S, 5R, 11R, 13R, 17S, 23R conformer 34

C	-2.95152	-1.90326	-1.06289
C	-4.10899	-1.95372	-1.77485
C	-5.4062	-1.70198	-1.15624
C	-5.33485	-1.4191	0.34518
C	-4.02939	-0.71285	0.69851
O	-2.89064	-1.47446	0.21572
C	-1.61813	-2.332	-1.60931
C	-0.53281	-1.23449	-1.72373
C	-1.08964	0.11019	-2.22403
C	-0.0347	1.04297	-2.85373
C	-0.33235	2.56003	-2.66954
C	0.34802	3.0717	-1.40305

C	0.09781	3.40404	-3.87412
O	0.10046	2.25227	-0.34586
O	1.05931	4.04963	-1.33131
C	0.94566	2.42497	0.82418
C	0.27421	1.67026	1.98118
C	2.31751	1.90143	0.51784
C	-1.05067	2.27582	2.42559
C	-3.82878	-0.49918	2.19438
C	-2.49504	0.14779	2.62396
C	-2.32181	1.56371	2.01541
C	-1.07439	3.3993	3.15108
C	-2.41581	0.17068	4.15756
O	-6.47192	-1.76348	-1.75605
O	0.08848	-1.12367	-0.44747
C	3.49775	2.56967	0.50959
O	4.48642	1.68475	0.19378
C	3.8542	0.48307	0.01163
N	2.55703	0.57073	0.18018
C	4.74715	-0.61419	-0.31425
C	4.53244	-1.94565	-0.25567
C	3.22797	-2.5937	0.19145
C	5.64541	-2.89349	-0.62495
C	2.54252	-3.36041	-0.94002
O	3.48317	-3.54654	1.22923
C	3.73358	-2.95915	2.49302
H	-4.09454	-2.27209	-2.81123
H	-6.19831	-0.81503	0.64004
H	-5.40089	-2.37683	0.88196
H	-3.9994	0.24669	0.16178
H	-1.78838	-2.76486	-2.60012
H	-1.20844	-3.1218	-0.96759
H	0.20026	-1.61171	-2.45818
H	-1.55925	0.59424	-1.36269
H	-1.88597	-0.06918	-2.95809
H	0.03213	0.83389	-3.92847
H	0.96154	0.82455	-2.44754
H	-1.41577	2.67951	-2.52361
H	-0.08917	4.46712	-3.70092
H	-0.4541	3.09161	-4.76698
H	1.16854	3.28627	-4.07257
H	1.01102	3.49269	1.04723

H	0.98519	1.67916	2.81586
H	0.1511	0.62749	1.67439
H	-3.92152	-1.47462	2.6908
H	-4.66763	0.11408	2.55439
H	-1.68961	-0.49048	2.24118
H	-2.32921	1.47986	0.92082
H	-3.18575	2.18125	2.29788
H	-2.00814	3.85849	3.46561
H	-0.1618	3.90797	3.45641
H	-1.46202	0.57809	4.50635
H	-2.52435	-0.8393	4.57011
H	-3.2134	0.79326	4.58477
H	0.83868	-0.49841	-0.48113
H	3.78699	3.596	0.66782
H	5.73394	-0.26435	-0.60968
H	2.53889	-1.82804	0.56323
H	6.58815	-2.36599	-0.79576
H	5.78883	-3.63979	0.16326
H	5.39913	-3.44996	-1.53894
H	1.58463	-3.7462	-0.58271
H	2.35698	-2.70299	-1.79521
H	3.15793	-4.20349	-1.26944
H	3.8689	-3.78077	3.20149
H	4.64132	-2.33678	2.49248
H	2.88703	-2.33741	2.82221

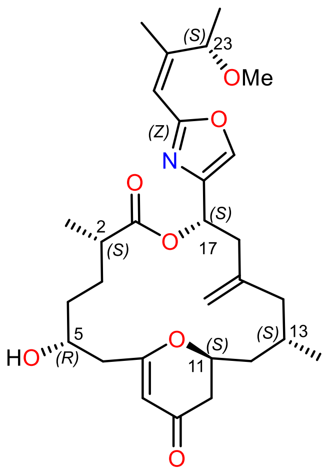

Table 4. Calculated DFT energies of the **1b**-(2S5R11S13S17S23S) diasteroisomer.

Conformers 1b	OPLS2008 Force Field	B3LYP/6-31+G(d,p)	Δ (DFT Energy)	% Population
**1b4**	1.495960803	-1710.456029	0	19.95
**1b3**	1.322944551	-1710.455954	0.0470632	18.43
**1b1**	0	-1710.455935	0.05898588	18.06
**1b18**	2.732791587	-1710.455096	0.58546626	7.42
**1b8**	1.785062141	-1710.454916	0.69841795	6.13
**1b2**	0.431716061	-1710.454814	0.76242391	5.50
**1b22**	3.070650096	-1710.454601	0.89608341	4.39
**1b17**	2.713217017	-1710.454415	1.01280016	3.60
**1b21**	2.994502868	-1710.454414	1.01342767	3.60
**1b27**	3.242160612	-1710.453776	1.41377866	1.83
**1b12**	2.148183556	-1710.453697	1.4633519	1.68
**1b10**	2.04292543	-1710.453516	1.5769311	1.39
**1b11**	2.075262906	-1710.453341	1.68674524	1.15
**1b6**	1.530640535	-1710.453309	1.70682554	1.12
**1b19**	2.852868069	-1710.453155	1.80346199	0.95
**1b56**	4.674689293	-1710.452849	1.99547986	0.68
**1b42**	4.111089866	-1710.45258	2.16427989	0.51
**1b32**	3.536567878	-1710.452455	2.24271856	0.45
**1b37**	3.785994264	-1710.452396	2.27974162	0.42
**1b55**	4.660994264	-1710.45214	2.44038402	0.32
**1b47**	4.423064054	-1710.452031	2.50878255	0.29
**1b23**	3.084799235	-1710.451875	2.60667401	0.24
**1b46**	4.35250956	-1710.451748	2.6863677	0.21
**1b52**	4.594144359	-1710.451529	2.82379226	0.17
**1b15**	2.470960803	-1710.451487	2.85014765	0.16
**1b28**	3.356046845	-1710.451399	2.90536848	0.15
**1b5**	1.508819312	-1710.451364	2.92733131	0.14
**1b63**	4.930760038	-1710.451278	2.98129712	0.13
**1b16**	2.509703633	-1710.451185	3.03965549	0.12
**1b9**	1.942853728	-1710.451176	3.04530307	0.12
**1b57**	4.736304971	-1710.451132	3.07291349	0.11
**1b13**	2.355066922	-1710.451021	3.14256703	0.10

32 conformers counting for the 99.54% of the DFT conformational population

DFT COORDINATES FOR CONFORMATIONAL SEARCH OF 1b
(2S*, 5R*, 11S*, 13S* , 17S*, 23S*)

Compound 1b: Isomer 2S, 5R, 11S, 13S, 17S, 23S conformer 1

C	-2.7622	1.47339	0.93925
C	-3.73055	2.32989	1.35375
C	-5.02562	2.40471	0.67885
C	-5.20834	1.39109	-0.45396
C	-3.88435	1.08016	-1.14898
O	-2.87568	0.70807	-0.17165
C	-1.47093	1.19493	1.65369
C	-1.42775	-0.25262	2.19889
C	-0.02543	-0.66238	2.64639
C	0.07092	-2.12399	3.1162
C	-0.28689	-3.21873	2.07283
C	0.71631	-3.24607	0.92742
C	-0.35465	-4.59943	2.73863
O	0.45508	-2.28699	0.00019
O	1.64775	-4.01942	0.83824
C	1.37386	-2.22347	-1.13629
C	0.66047	-1.48268	-2.27808
C	2.6547	-1.55016	-0.73799
C	-0.66458	-2.10069	-2.69273
C	-3.99053	-0.04051	-2.18409
C	-2.68874	-0.5075	-2.88788
C	-1.9234	-1.56028	-2.03703
C	-0.71036	-3.07892	-3.6041
C	-1.81353	0.67013	-3.34197
O	-5.92419	3.16194	1.01863
O	-2.29582	-0.40011	3.32177
C	3.83859	-2.12124	-0.39136
O	4.72965	-1.12495	-0.12173
C	4.03033	0.04058	-0.31204
N	2.7934	-0.16766	-0.684
C	4.78523	1.25905	-0.09841
C	4.32864	2.52954	-0.09821
C	2.87008	2.90304	-0.33419

C	5.28751	3.67516	0.09831
C	2.60639	3.23188	-1.80586
O	2.50338	4.05748	0.42331
C	2.27764	3.78636	1.79282
H	-3.57203	2.94733	2.23092
H	-5.62901	0.47081	-0.02217
H	-5.94053	1.78093	-1.1673
H	-3.51118	1.99887	-1.62362
H	-0.6464	1.33217	0.94258
H	-1.33922	1.89646	2.48349
H	-1.7494	-0.917	1.3857
H	0.27583	-0.01042	3.47734
H	0.66883	-0.47768	1.81985
H	-0.60504	-2.25895	3.96748
H	1.08723	-2.31616	3.48477
H	-1.2658	-2.98057	1.6421
H	-0.6248	-5.3772	2.01707
H	-1.10465	-4.59454	3.53642
H	0.61327	-4.87154	3.17
H	1.58677	-3.25403	-1.4293
H	1.35966	-1.48277	-3.12304
H	0.53018	-0.43819	-1.97998
H	-4.47836	-0.90545	-1.71446
H	-4.68877	0.32861	-2.94782
H	-3.01372	-1.03274	-3.79755
H	-1.67102	-1.1263	-1.06818
H	-2.61539	-2.39015	-1.84213
H	-1.64753	-3.55177	-3.88975
H	0.18507	-3.44936	-4.09883
H	-2.39704	1.39116	-3.92795
H	-1.37557	1.20439	-2.49098
H	-0.99285	0.32048	-3.97617
H	-3.18635	-0.12551	3.0493
H	4.17923	-3.13678	-0.27384
H	5.8451	1.08188	0.06767
H	2.23012	2.0586	-0.04904
H	6.3189	3.32212	0.18781
H	5.23444	4.37651	-0.74425
H	5.03794	4.25825	0.99144

H	3.23996	4.05773	-2.14608
H	1.56069	3.53068	-1.93
H	2.8009	2.35343	-2.42758
H	1.47308	3.04655	1.93001
H	3.17823	3.40964	2.30185
H	1.977	4.72863	2.25886

Compound 1b: Isomer 2S, 5R, 11S, 13S, 17S, 23S conformer 2

C	1.93844	-2.17962	0.71187
C	3.01649	-2.88212	1.15156
C	4.0314	-3.37844	0.22732
C	3.69727	-3.15818	-1.25017
C	2.90355	-1.87012	-1.48395
O	1.7672	-1.79169	-0.5719
C	0.83847	-1.65717	1.59021
C	1.16278	-0.22159	2.07575
C	-0.05366	0.47944	2.67874
C	0.23884	1.89811	3.19935
C	0.88708	2.89804	2.20256
C	-0.01915	3.16984	1.00869
C	1.22647	4.2173	2.90902
O	0.11553	2.21778	0.0484
O	-0.78149	4.10906	0.90949
C	-0.73956	2.34823	-1.13015
C	-0.09874	1.49724	-2.23186
C	-2.14399	1.92085	-0.81833
C	1.22869	2.00968	-2.78459
C	3.76066	-0.60021	-1.36758
C	2.99572	0.73718	-1.4408
C	2.36105	1.00313	-2.83138
C	1.37122	3.25583	-3.24778
C	3.91258	1.89861	-1.03268
O	5.03661	-3.98151	0.57819
O	2.19245	-0.23842	3.06221
C	-3.21945	2.70775	-0.55029
O	-4.30396	1.90152	-0.37193
C	-3.82913	0.62391	-0.5339
N	-2.54916	0.59037	-0.80458

C	-4.82276	-0.425	-0.41885
C	-4.62004	-1.75849	-0.3672
C	-3.24039	-2.40427	-0.40205
C	-5.79631	-2.69881	-0.30899
C	-2.8463	-2.82147	-1.82068
O	-3.20284	-3.58297	0.40453
C	-3.14766	-3.31877	1.79309
H	3.09731	-3.15007	2.19972
H	4.62337	-3.1553	-1.83243
H	3.10591	-4.02341	-1.58097
H	2.42481	-1.90489	-2.46587
H	-0.09102	-1.64661	1.01029
H	0.70517	-2.30333	2.4637
H	1.48895	0.35537	1.20242
H	-0.43129	-0.1225	3.51644
H	-0.84105	0.50157	1.91683
H	0.92192	1.81944	4.05156
H	-0.69374	2.33788	3.57696
H	1.80954	2.44727	1.82091
H	1.72388	4.91703	2.2294
H	1.89418	4.02859	3.75617
H	0.31925	4.70386	3.27934
H	-0.7509	3.4058	-1.40211
H	-0.82554	1.43029	-3.052
H	0.00965	0.47847	-1.84711
H	4.31656	-0.6353	-0.42118
H	4.5175	-0.64557	-2.16415
H	2.17935	0.68737	-0.7139
H	3.14165	1.3449	-3.52365
H	1.96638	0.0645	-3.24487
H	2.31004	3.60053	-3.67347
H	0.56072	3.98124	-3.22906
H	4.28199	1.77259	-0.00783
H	4.78612	1.96482	-1.69481
H	3.37847	2.85266	-1.08513
H	2.97507	-0.65243	2.66194
H	-3.36685	3.76946	-0.43873
H	-5.84185	-0.04709	-0.38599
H	-2.49647	-1.68515	-0.03604

H	-6.74467	-2.15578	-0.35456
H	-5.76586	-3.41062	-1.1439
H	-5.78236	-3.30408	0.60377
H	-3.57115	-3.52673	-2.24062
H	-1.8671	-3.30986	-1.79592
H	-2.78716	-1.94126	-2.46687
H	-2.2583	-2.72509	2.05611
H	-4.03684	-2.7802	2.15524
H	-3.09102	-4.2864	2.29892

Compound 1b: Isomer 2S, 5R, 11S, 13S, 17S, 23S conformer 3

C	1.68675	2.21305	-0.99075
C	2.69087	3.02325	-1.41999
C	3.49482	3.80213	-0.48325
C	3.01475	3.72926	0.96837
C	2.40341	2.36925	1.31492
O	1.41745	1.97669	0.3126
C	0.79836	1.3983	-1.88652
C	1.40954	-0.00518	-2.13165
C	0.40117	-0.98122	-2.73546
C	0.96338	-2.3944	-2.96993
C	1.57245	-3.12015	-1.73822
C	0.53189	-3.31819	-0.64364
C	2.16486	-4.47497	-2.14825
O	0.43643	-2.23102	0.1623
O	-0.14914	-4.31391	-0.50197
C	-0.56378	-2.28385	1.23112
C	-0.15383	-1.2261	2.25864
C	-1.94103	-2.04669	0.67727
C	1.14825	-1.49312	3.00782
C	3.45008	1.25743	1.48613
C	2.8898	-0.16528	1.68871
C	2.13724	-0.34465	3.03499
C	1.38657	-2.64719	3.6393
C	4.0101	-1.2059	1.55308
O	4.43306	4.51294	-0.81746
O	2.52582	0.07018	-3.0151
C	-2.818	-2.96007	0.1793

O	-3.95076	-2.30151	-0.19611
C	-3.71103	-0.98159	0.0943
N	-2.52751	-0.7869	0.61702
C	-4.80485	-0.07747	-0.19925
C	-4.83825	1.26526	-0.06279
C	-3.67076	2.093	0.4616
C	-6.10156	2.02236	-0.38205
C	-3.77056	2.29868	1.97574
O	-3.64932	3.39189	-0.13249
C	-3.12758	3.40524	-1.44652
H	2.86021	3.15916	-2.48295
H	3.8496	3.96148	1.63605
H	2.26164	4.51867	1.10032
H	1.80338	2.45507	2.22429
H	-0.17721	1.28968	-1.39952
H	0.66173	1.89656	-2.85161
H	1.72701	-0.39822	-1.15844
H	0.05532	-0.5817	-3.69821
H	-0.46951	-1.02083	-2.07154
H	1.75374	-2.33498	-3.72572
H	0.1691	-3.02747	-3.38682
H	2.36831	-2.48901	-1.32867
H	2.63811	-4.97692	-1.29783
H	2.92165	-4.33417	-2.92706
H	1.38526	-5.13885	-2.53335
H	-0.52089	-3.28984	1.65336
H	-0.97556	-1.13859	2.98123
H	-0.10978	-0.26132	1.74407
H	4.10851	1.25375	0.60729
H	4.08545	1.53813	2.33871
H	2.169	-0.34959	0.88634
H	2.86857	-0.4929	3.84019
H	1.5865	0.57442	3.27963
H	2.30394	-2.80917	4.19941
H	0.67933	-3.47374	3.63127
H	4.47655	-1.16102	0.56151
H	4.79877	-1.03931	2.29878
H	3.61963	-2.21848	1.69752
H	3.18556	0.65419	-2.60564

H	-2.78338	-4.02449	0.01697
H	-5.69538	-0.58989	-0.55508
H	-2.7287	1.5736	0.24578
H	-6.91304	1.34445	-0.66213
H	-6.42848	2.61647	0.48075
H	-5.94574	2.7365	-1.19811
H	-4.70863	2.79539	2.24512
H	-2.94098	2.92872	2.31147
H	-3.71652	1.33552	2.49085
H	-2.08948	3.03815	-1.47123
H	-3.72381	2.79522	-2.14275
H	-3.14395	4.44456	-1.78575

Compound 1b: Isomer 2S, 5R, 11S, 13S, 17S, 23S conformer 4

C	2.13043	-2.14392	0.39905
C	2.49396	-3.38098	-0.02555
C	3.56162	-3.56701	-1.00781
C	4.25772	-2.27395	-1.44624
C	3.30316	-1.08367	-1.40771
O	2.64889	-1.00477	-0.11344
C	1.15591	-1.82941	1.49586
C	1.85895	-1.19417	2.72274
C	0.89864	-0.47453	3.67543
C	0.1197	0.70511	3.06633
C	0.99338	1.87865	2.53278
C	0.17412	2.665	1.51865
C	1.50794	2.78846	3.6519
O	0.13331	2.00309	0.33274
O	-0.39963	3.71328	1.72473
C	-0.7763	2.52016	-0.68717
C	-0.31672	1.93777	-2.0311
C	-2.18251	2.12886	-0.3411
C	0.94582	2.54992	-2.63273
C	3.97068	0.27132	-1.62959
C	3.02386	1.48888	-1.52922
C	2.15438	1.64948	-2.80497
C	0.95544	3.82249	-3.04581
C	3.8274	2.76648	-1.25014

O	3.92473	-4.66248	-1.41339
O	2.51529	-2.19576	3.49684
C	-3.14422	2.87619	0.2624
O	-4.26664	2.11017	0.38018
C	-3.92791	0.89565	-0.16287
N	-2.69873	0.86638	-0.60925
C	-4.97845	-0.10242	-0.15894
C	-4.88151	-1.40718	-0.49209
C	-3.58885	-2.06863	-0.95545
C	-6.10428	-2.28716	-0.45751
C	-3.48646	-2.08885	-2.4828
O	-3.5187	-3.42574	-0.51444
C	-3.16394	-3.55946	0.84759
H	2.01899	-4.263	0.38948
H	5.10454	-2.09604	-0.76699
H	4.66836	-2.41316	-2.45078
H	2.49923	-1.24995	-2.14015
H	0.41974	-1.13005	1.08376
H	0.63532	-2.73564	1.82139
H	2.60058	-0.47279	2.35101
H	1.49055	-0.14323	4.53728
H	0.18164	-1.2101	4.06352
H	-0.56299	1.10501	3.82696
H	-0.51798	0.3371	2.25507
H	1.83911	1.45766	1.97794
H	2.1724	3.565	3.25904
H	2.06615	2.20927	4.39469
H	0.67391	3.28827	4.15376
H	-0.69288	3.6091	-0.68096
H	-1.14588	2.077	-2.73507
H	-0.2088	0.85613	-1.90481
H	4.76662	0.37164	-0.87958
H	4.46681	0.25535	-2.6103
H	2.35274	1.31857	-0.68124
H	2.79079	2.03823	-3.61139
H	1.80504	0.66121	-3.13585
H	1.83223	4.26709	-3.50937
H	0.08679	4.47007	-2.94306
H	4.37257	2.68974	-0.30195

H	4.56404	2.95229	-2.04345
H	3.17085	3.64035	-1.19177
H	3.12594	-2.66924	2.90851
H	-3.18468	3.87891	0.65552
H	-5.94337	0.2909	0.15172
H	-2.7319	-1.5093	-0.56007
H	-7.00016	-1.71704	-0.1953
H	-6.26947	-2.76305	-1.43246
H	-5.98673	-3.10557	0.26116
H	-2.5684	-2.60632	-2.77819
H	-3.45903	-1.06661	-2.87059
H	-4.33531	-2.61755	-2.92899
H	-3.11497	-4.63073	1.06061
H	-2.18021	-3.10841	1.05392
H	-3.90073	-3.09642	1.52231

Compound 1b: Isomer 2S, 5R, 11S, 13S, 17S, 23S conformer 5

C	-1.44485	2.62473	1.37115
C	-0.24764	3.25658	1.2719
C	0.21273	3.83461	0.01283
C	-0.76265	3.66387	-1.15051
C	-1.57884	2.38495	-0.99221
O	-2.24186	2.37106	0.30378
C	-2.03846	2.12195	2.65366
C	-2.20031	0.58309	2.73535
C	-0.86568	-0.16445	2.64572
C	-0.95484	-1.6617	2.98796
C	-1.88789	-2.54108	2.10765
C	-1.21836	-2.96768	0.80667
C	-2.35931	-3.78096	2.87776
O	-0.99618	-1.91169	-0.02037
O	-0.92004	-4.10757	0.51797
C	-0.40088	-2.21938	-1.31986
C	-0.80058	-1.09686	-2.29347
C	1.0822	-2.3825	-1.18874
C	-2.29745	-1.00814	-2.54557
C	-2.62526	2.19402	-2.08741
C	-3.7046	1.11879	-1.85283

C	-3.17392	-0.30491	-1.51601
C	-2.81792	-1.60392	-3.62657
C	-4.68836	1.11949	-3.03196
O	1.26472	4.45031	-0.10748
O	-2.79652	0.23986	3.98759
C	1.79326	-3.53997	-1.21405
O	3.11283	-3.23092	-1.0784
C	3.14879	-1.8618	-0.96061
N	1.96149	-1.31686	-1.031
C	4.47794	-1.30644	-0.79388
C	4.82984	-0.05471	-0.43259
C	3.8373	1.0581	-0.12041
C	6.28852	0.30864	-0.32095
C	3.72275	2.06412	-1.26835
O	4.25643	1.79043	1.03416
C	4.01646	1.11235	2.25072
H	0.36267	3.40688	2.15512
H	-1.43344	4.53536	-1.17141
H	-0.20409	3.65702	-2.09157
H	-0.88785	1.53281	-0.9685
H	-1.42915	2.45651	3.49877
H	-3.03582	2.5728	2.76723
H	-2.85369	0.26455	1.90982
H	-0.16333	0.2988	3.35244
H	-0.44865	-0.02194	1.64363
H	-1.31387	-1.749	4.01779
H	0.05643	-2.08901	2.97395
H	-2.76607	-1.94417	1.83026
H	-2.9969	-4.41859	2.2584
H	-2.92536	-3.47296	3.76288
H	-1.50723	-4.38638	3.20329
H	-0.82943	-3.16758	-1.65256
H	-0.2816	-1.30853	-3.23536
H	-0.38493	-0.15761	-1.91575
H	-3.13587	3.15304	-2.24962
H	-2.07849	1.97798	-3.01597
H	-4.26379	1.42515	-0.95904
H	-2.6353	-0.25335	-0.56722
H	-4.0593	-0.92733	-1.33005

H	-3.88779	-1.62408	-3.81557
H	-2.19146	-2.10728	-4.35995
H	-5.12491	2.11516	-3.17572
H	-4.19445	0.83303	-3.96703
H	-5.51255	0.41698	-2.85922
H	-3.67761	0.64387	4.02095
H	1.53368	-4.58308	-1.29404
H	5.26362	-2.0346	-0.98227
H	2.84739	0.62196	0.06248
H	6.93466	-0.53876	-0.56796
H	6.53746	1.1383	-0.99488
H	6.52932	0.65649	0.68918
H	4.69437	2.51446	-1.49703
H	3.03535	2.8654	-0.98236
H	3.34596	1.5662	-2.16778
H	4.57759	0.16746	2.32413
H	4.34065	1.77856	3.055
H	2.94619	0.88664	2.38222

Compound 1b: Isomer 2S, 5R, 11S, 13S, 17S, 23S conformer 6

C	-1.64397	-2.01349	-2.067
C	-0.34154	-2.31301	-2.30832
C	0.47562	-3.02258	-1.33311
C	-0.27507	-3.44566	-0.07274
C	-1.37785	-2.44311	0.25801
O	-2.26198	-2.26774	-0.88972
C	-2.55004	-1.32528	-3.04381
C	-2.91169	0.12935	-2.64335
C	-1.68739	1.0485	-2.57827
C	-2.02093	2.53222	-2.34845
C	-2.66656	2.90034	-0.98519
C	-1.64021	2.85741	0.14012
C	-3.29734	4.2991	-1.04531
O	-1.53962	1.61888	0.68964
O	-0.9846	3.80642	0.51701
C	-0.64224	1.48827	1.83583
C	-1.09489	0.27706	2.68116
C	0.77127	1.33508	1.35961

C	-2.59467	0.13832	2.87509
C	-2.21574	-2.84624	1.46981
C	-3.51971	-2.06268	1.73836
C	-3.41105	-0.50966	1.76728
C	-3.17322	0.59672	3.99182
C	-4.19561	-2.61077	3.00366
O	1.65923	-3.29666	-1.50938
O	-3.80114	0.67997	-3.61579
C	1.77784	2.24594	1.34455
O	2.87995	1.65052	0.79393
C	2.48224	0.37368	0.48694
N	1.23263	0.14827	0.8061
C	3.37157	-0.57311	-0.1527
C	4.69586	-0.48866	-0.40382
C	5.58122	0.69577	-0.02949
C	5.37553	-1.6169	-1.13996
C	5.7539	1.66639	-1.20123
O	6.89115	0.26426	0.343
C	6.95141	-0.30228	1.63756
H	0.1069	-2.05093	-3.25985
H	-0.70519	-4.4443	-0.2402
H	0.43468	-3.52412	0.75606
H	-0.90666	-1.4658	0.40823
H	-2.08984	-1.31235	-4.03659
H	-3.48408	-1.90107	-3.11576
H	-3.4023	0.09921	-1.65942
H	-1.1513	0.96562	-3.53344
H	-1.00872	0.67738	-1.80269
H	-2.71301	2.85197	-3.13388
H	-1.10728	3.12836	-2.47007
H	-3.44381	2.16406	-0.75231
H	-3.75781	4.57246	-0.09013
H	-4.06971	4.32779	-1.82087
H	-2.54049	5.05455	-1.27642
H	-0.73697	2.41036	2.41338
H	-0.60213	0.389	3.65439
H	-0.67722	-0.62515	2.23111
H	-2.48458	-3.90665	1.36651
H	-1.55373	-2.78948	2.34509

H	-4.18374	-2.27419	0.88963
H	-3.02854	-0.17409	0.80043
H	-4.43995	-0.13418	1.84099
H	-4.24741	0.5333	4.14805
H	-2.59627	1.05597	4.79175
H	-4.34872	-3.6943	2.93266
H	-3.59137	-2.41242	3.89627
H	-5.17618	-2.14515	3.15903
H	-4.62312	0.16571	-3.59558
H	1.86939	3.28114	1.63031
H	2.84176	-1.46663	-0.47212
H	5.13609	1.24006	0.8125
H	5.89022	-1.24471	-2.03508
H	6.1481	-2.08812	-0.52192
H	4.65351	-2.3785	-1.44563
H	6.19425	1.16094	-2.06668
H	6.42116	2.48198	-0.90523
H	4.78648	2.08564	-1.49298
H	6.33263	-1.20793	1.72873
H	7.99616	-0.56823	1.81995
H	6.62584	0.41556	2.40725

Compound 1b: Isomer 2S, 5R, 11S, 13S, 17S, 23S conformer 8

C	-1.30813	2.36179	0.73763
C	-1.38994	3.71158	0.63625
C	-2.54415	4.36444	0.02249
C	-3.66677	3.41135	-0.40285
C	-3.12208	2.04597	-0.81379
O	-2.23345	1.52576	0.20817
C	-0.19897	1.58623	1.39737
C	-0.70077	0.4182	2.27339
C	0.42593	-0.54195	2.65526
C	-0.05773	-1.85685	3.29185
C	-1.01169	-2.74126	2.43948
C	-0.37451	-3.13418	1.11212
C	-1.42013	-3.99911	3.21739
O	-0.54101	-2.16506	0.17402
O	0.22111	-4.16995	0.90005

C	0.09744	-2.38243	-1.12294
C	-0.56875	-1.4026	-2.09624
C	1.58252	-2.18257	-1.03341
C	-1.95866	-1.77521	-2.60368
C	-4.19858	0.98195	-1.03269
C	-3.6746	-0.45573	-1.24131
C	-2.97581	-0.65051	-2.6162
C	-2.25221	-2.9969	-3.0612
C	-4.81321	-1.47017	-1.06553
O	-2.65428	5.57712	-0.0975
O	-1.28958	0.88547	3.48994
C	2.55047	-3.13575	-1.00272
O	3.76256	-2.51327	-0.98938
C	3.47543	-1.1713	-1.00082
N	2.18892	-0.92969	-1.03124
C	4.63133	-0.29605	-1.02749
C	4.67101	1.03814	-0.82822
C	3.44426	1.87437	-0.48994
C	5.97355	1.7835	-0.96388
C	2.83389	2.52478	-1.7335
O	3.77198	2.93111	0.41366
C	3.97592	2.49852	1.74541
H	-0.6089	4.33707	1.05383
H	-4.35429	3.29847	0.44846
H	-4.23257	3.86805	-1.22042
H	-2.50267	2.16853	-1.71454
H	0.42048	1.16343	0.59372
H	0.42816	2.26068	1.98907
H	-1.44416	-0.12894	1.68526
H	1.08595	-0.03159	3.36963
H	1.02251	-0.75228	1.76188
H	-0.58555	-1.62482	4.22393
H	0.81739	-2.46133	3.56441
H	-1.90808	-2.15589	2.20677
H	-2.13821	-4.60242	2.65216
H	-1.88218	-3.71977	4.17023
H	-0.54813	-4.6271	3.42223
H	-0.09211	-3.42001	-1.40523
H	0.09793	-1.28773	-2.9609

H	-0.59085	-0.42247	-1.60952
H	-4.85935	0.99061	-0.15514
H	-4.81491	1.29117	-1.88878
H	-2.93338	-0.65004	-0.45854
H	-3.74095	-0.83927	-3.38038
H	-2.46627	0.27705	-2.91126
H	-3.23208	-3.22906	-3.47016
H	-1.53285	-3.81261	-3.05706
H	-5.24267	-1.41338	-0.05828
H	-5.62253	-1.28717	-1.78494
H	-4.45141	-2.49213	-1.21911
H	-2.10074	1.3659	3.26208
H	2.54594	-4.21296	-0.96951
H	5.55761	-0.82095	-1.24912
H	2.68741	1.22525	-0.03227
H	6.78843	1.11459	-1.25535
H	5.89036	2.57482	-1.71999
H	6.24799	2.28574	-0.03015
H	3.55838	3.17527	-2.23443
H	1.97399	3.13489	-1.43901
H	2.50039	1.75422	-2.43424
H	3.08531	1.98972	2.14592
H	4.832	1.81292	1.83941
H	4.17312	3.39268	2.34279

Compound 1b: Isomer 2S, 5R, 11S, 13S, 17S, 23S conformer 9

C	-1.81954	-2.6245	-1.46403
C	-0.6657	-2.84629	-2.14348
C	0.56398	-3.23287	-1.46234
C	0.42352	-3.38807	0.05053
C	-0.64731	-2.44919	0.59946
O	-1.9032	-2.6446	-0.11162
C	-3.1313	-2.29022	-2.10806
C	-3.61248	-0.8417	-1.83481
C	-2.62985	0.21618	-2.34875
C	-3.15905	1.65726	-2.27388
C	-3.44007	2.22578	-0.85477
C	-2.16365	2.6985	-0.16776

C	-4.44265	3.38589	-0.9239
O	-1.4245	1.67346	0.32536
O	-1.83691	3.86335	-0.05476
C	-0.2848	2.04492	1.17488
C	-0.02695	0.88526	2.14963
C	0.92583	2.39003	0.34945
C	-1.22614	0.51245	3.00542
C	-0.89341	-2.62477	2.09647
C	-2.14075	-1.94353	2.69725
C	-2.28325	-0.41722	2.42721
C	-1.35325	1.03447	4.23198
C	-2.23548	-2.27741	4.193
O	1.61676	-3.46132	-2.04707
O	-4.85704	-0.62348	-2.49945
C	1.15023	3.50856	-0.39387
O	2.39911	3.41621	-0.93138
C	2.9012	2.22338	-0.47515
N	2.05414	1.57971	0.28753
C	4.26554	1.94053	-0.87121
C	5.01317	0.85329	-0.585
C	4.51515	-0.33017	0.23901
C	6.45236	0.79722	-1.02793
C	4.74632	-0.09167	1.73501
O	5.20977	-1.5266	-0.09897
C	4.75218	-2.14337	-1.29381
H	-0.65328	-2.78613	-3.22574
H	0.1535	-4.43222	0.26671
H	1.3897	-3.1944	0.52664
H	-0.35278	-1.41635	0.37477
H	-3.06242	-2.43834	-3.19003
H	-3.89477	-2.98253	-1.72421
H	-3.74133	-0.7258	-0.74856
H	-2.40278	-0.00911	-3.39953
H	-1.69044	0.12366	-1.7941
H	-4.09887	1.70227	-2.83245
H	-2.45634	2.32473	-2.78943
H	-3.86277	1.42645	-0.23466
H	-4.63994	3.80557	0.06742
H	-5.38885	3.03263	-1.3468

H	-4.05918	4.19473	-1.55325
H	-0.6035	2.92921	1.7319
H	0.80181	1.20541	2.7905
H	0.34977	0.0314	1.58148
H	-0.96613	-3.70017	2.30888
H	0.01247	-2.27557	2.61151
H	-3.00998	-2.39628	2.20223
H	-2.34448	-0.26195	1.34704
H	-3.25881	-0.1242	2.83653
H	-2.21828	0.8282	4.8575
H	-0.59933	1.69425	4.65619
H	-2.22408	-3.36191	4.35454
H	-1.40019	-1.84417	4.75442
H	-3.16433	-1.88665	4.62527
H	-5.51176	-1.22901	-2.1183
H	0.56858	4.38446	-0.62672
H	4.71582	2.74893	-1.44213
H	3.43814	-0.46187	0.07027
H	6.75537	1.72807	-1.51628
H	7.1145	0.62164	-0.17073
H	6.62916	-0.03338	-1.71977
H	5.80918	0.06978	1.94428
H	4.41815	-0.97148	2.29773
H	4.17826	0.77975	2.07146
H	4.85247	-1.48198	-2.16815
H	5.37945	-3.02539	-1.4495
H	3.70424	-2.46275	-1.21978

Compound 1b: Isomer 2S, 5R, 11S, 13S, 17S, 23S conformer 10

C	2.27138	-2.31289	0.0209
C	2.95704	-3.29105	-0.62467
C	4.23248	-3.03237	-1.2849
C	4.74015	-1.59881	-1.12869
C	3.58722	-0.60312	-1.03067
O	2.64244	-1.01089	0.00123
C	1.01828	-2.5559	0.81621
C	1.11952	-2.34468	2.35208
C	1.30984	-0.8957	2.81802

C	0.1762	0.05385	2.40512
C	0.47099	1.54449	2.71923
C	-0.35293	2.44904	1.80556
C	0.25836	1.91692	4.18882
O	-0.09837	2.15146	0.504
O	-1.12137	3.31758	2.15487
C	-0.89029	2.80311	-0.52164
C	-0.16953	2.53662	-1.85389
C	-2.28472	2.25103	-0.52062
C	1.19618	3.19692	-2.01642
C	4.04021	0.81386	-0.68923
C	2.92625	1.87891	-0.59446
C	2.4328	2.31591	-1.99877
C	1.28495	4.5147	-2.23169
C	3.42429	3.08523	0.2137
O	4.88298	-3.8899	-1.86742
O	-0.0832	-2.81372	2.9699
C	-3.454	2.88688	-0.25062
O	-4.47133	1.99019	-0.40117
C	-3.85789	0.81338	-0.75064
N	-2.55838	0.92731	-0.84402
C	-4.74328	-0.31229	-0.97261
C	-4.40844	-1.61562	-1.07844
C	-2.9801	-2.13488	-0.95763
C	-5.47159	-2.64637	-1.35912
C	-2.31734	-2.28323	-2.32937
O	-2.95439	-3.42305	-0.33975
C	-3.14999	-3.38341	1.0642
H	2.58968	-4.31105	-0.61205
H	5.35342	-1.55149	-0.21667
H	5.39074	-1.35401	-1.97361
H	3.02091	-0.62109	-1.97368
H	0.21381	-1.92474	0.42235
H	0.71807	-3.59618	0.64586
H	1.97667	-2.93392	2.71585
H	2.26082	-0.53135	2.4143
H	1.40799	-0.92128	3.91013
H	-0.75601	-0.24447	2.89868
H	0.00937	-0.03657	1.32988

H	1.51951	1.73448	2.4452
H	0.50183	2.96776	4.3711
H	0.88899	1.30013	4.83737
H	-0.78638	1.76501	4.47897
H	-0.92473	3.87383	-0.30142
H	-0.83714	2.89587	-2.64625
H	-0.09932	1.45092	-1.97709
H	4.57098	0.76191	0.27102
H	4.78658	1.11704	-1.4373
H	2.08298	1.43532	-0.0585
H	3.25459	2.84461	-2.50106
H	2.22679	1.41992	-2.60191
H	2.24247	5.00914	-2.37382
H	0.40397	5.15209	-2.27774
H	3.71295	2.78626	1.22865
H	4.3044	3.54314	-0.2583
H	2.65185	3.8558	0.29409
H	-0.17759	-3.75831	2.77007
H	-3.72056	3.88569	0.05524
H	-5.78965	-0.02876	-1.05817
H	-2.38781	-1.42792	-0.36269
H	-6.45104	-2.18076	-1.50229
H	-5.22745	-3.22177	-2.26123
H	-5.54577	-3.37669	-0.5462
H	-1.30992	-2.69368	-2.20579
H	-2.24557	-1.30732	-2.81734
H	-2.88598	-2.96432	-2.97125
H	-3.17512	-4.42246	1.40666
H	-2.33075	-2.85618	1.57469
H	-4.10039	-2.90224	1.3406

Compound 1b: Isomer 2S, 5R, 11S, 13S, 17S, 23S conformer 11

C	2.39057	-2.28419	0.05881
C	3.09646	-3.24806	-0.58547
C	4.34218	-2.95363	-1.28646
C	4.79557	-1.49769	-1.17614
C	3.60612	-0.54703	-1.06688
O	2.70927	-0.96777	0.00123

C	1.16367	-2.55915	0.88282
C	1.2844	-2.29918	2.40018
C	1.44383	-0.82936	2.82658
C	0.28513	0.08071	2.39801
C	0.52417	1.58259	2.69863
C	-0.34719	2.4534	1.79436
C	0.32095	1.95804	4.16904
O	-0.14216	2.1272	0.49296
O	-1.10791	3.32404	2.15612
C	-0.9725	2.75844	-0.51798
C	-0.29153	2.47857	-1.86804
C	-2.36115	2.19626	-0.46021
C	1.05928	3.15373	-2.08529
C	4.01281	0.89428	-0.77216
C	2.86554	1.92313	-0.67529
C	2.31237	2.29524	-2.0762
C	1.12019	4.46612	-2.34021
C	3.34817	3.17199	0.07564
O	5.01161	-3.79697	-1.86818
O	0.0902	-2.87378	2.94573
C	-3.51858	2.818	-0.11624
O	-4.53572	1.91564	-0.22723
C	-3.93368	0.74969	-0.63121
N	-2.64189	0.87628	-0.79205
C	-4.82255	-0.37853	-0.8217
C	-4.48501	-1.67403	-0.99551
C	-3.04713	-2.18149	-1.01295
C	-5.55756	-2.70815	-1.22147
C	-2.49804	-2.25624	-2.44036
O	-2.95998	-3.49587	-0.46419
C	-3.01913	-3.52861	0.95255
H	2.77013	-4.28082	-0.53937
H	5.43115	-1.40478	-0.28306
H	5.41245	-1.24703	-2.04436
H	3.01485	-0.61036	-1.99243
H	0.32709	-1.97342	0.48448
H	0.89975	-3.61382	0.76728
H	2.16182	-2.85782	2.7656
H	2.38433	-0.44993	2.41091

H	1.56104	-0.81208	3.92101
H	-0.64485	-0.24383	2.88126
H	0.13231	-0.02877	1.3229
H	1.56034	1.81236	2.40774
H	0.51686	3.02082	4.33696
H	0.99264	1.37975	4.81216
H	-0.71042	1.76084	4.48015
H	-1.00671	3.83165	-0.31084
H	-0.98892	2.81745	-2.64344
H	-0.21253	1.39206	-1.97761
H	4.56856	0.88642	0.17531
H	4.72898	1.20523	-1.54611
H	2.05571	1.47017	-0.0974
H	3.10548	2.81887	-2.62762
H	2.10317	1.37262	-2.63618
H	2.06541	4.97146	-2.52108
H	0.22766	5.08756	-2.38124
H	3.67962	2.91781	1.08984
H	4.19668	3.64189	-0.44031
H	2.55314	3.91925	0.15511
H	0.09879	-2.7187	3.90351
H	-3.77482	3.80919	0.22148
H	-5.87484	-0.10436	-0.81724
H	-2.41398	-1.49577	-0.43537
H	-6.54996	-2.25066	-1.27139
H	-5.38034	-3.25416	-2.15685
H	-5.55762	-3.46236	-0.42724
H	-1.48227	-2.66352	-2.41745
H	-2.47164	-1.25805	-2.88603
H	-3.11359	-2.91169	-3.06547
H	-2.96903	-4.58126	1.24423
H	-2.17351	-2.99581	1.41037
H	-3.95646	-3.09923	1.33942

Compound 1b: Isomer 2S, 5R, 11S, 13S, 17S, 23S conformer 12

C	-1.6503	-2.03011	-2.04984
C	-0.3477	-2.32941	-2.29286
C	0.47589	-3.02409	-1.31299

C	-0.26951	-3.43649	-0.04633
C	-1.37393	-2.43383	0.27928
O	-2.26183	-2.26747	-0.86706
C	-2.56348	-1.36189	-3.03439
C	-2.93221	0.0975	-2.66101
C	-1.70887	1.0265	-2.57975
C	-2.05746	2.5082	-2.35965
C	-2.68892	2.8772	-0.9907
C	-1.64696	2.85011	0.12132
C	-3.33386	4.26953	-1.04974
O	-1.53702	1.61842	0.68241
O	-0.99036	3.80559	0.47942
C	-0.63561	1.502	1.82702
C	-1.08432	0.29814	2.68491
C	0.77692	1.34762	1.34853
C	-2.58362	0.16	2.88476
C	-2.20756	-2.83147	1.49587
C	-3.51175	-2.04862	1.76504
C	-3.4048	-0.49546	1.78474
C	-3.15762	0.62552	4.00092
C	-4.18135	-2.59051	3.03632
O	1.66017	-3.29514	-1.49055
O	-3.91427	0.58399	-3.57662
C	1.78384	2.25802	1.32965
O	2.88552	1.66017	0.78068
C	2.48712	0.38227	0.47869
N	1.23753	0.15867	0.79911
C	3.37577	-0.56771	-0.15717
C	4.7002	-0.48536	-0.40828
C	5.58638	0.70003	-0.03888
C	5.37929	-1.61749	-1.13896
C	5.7593	1.66589	-1.21455
O	6.89614	0.26936	0.33482
C	6.95672	-0.29175	1.63182
H	0.09579	-2.08071	-3.25039
H	-0.69761	-4.43774	-0.20296
H	0.44281	-3.5052	0.7811
H	-0.90471	-1.45443	0.42169
H	-2.09299	-1.37468	-4.02536

H	-3.50119	-1.92626	-3.10132
H	-3.44833	0.07777	-1.69649
H	-1.14963	0.94266	-3.52511
H	-1.03236	0.66565	-1.79688
H	-2.77024	2.81297	-3.1339
H	-1.15633	3.11845	-2.50175
H	-3.45606	2.13495	-0.74496
H	-3.78342	4.54246	-0.0894
H	-4.11842	4.28622	-1.81325
H	-2.58779	5.03141	-1.29474
H	-0.73127	2.42971	2.39542
H	-0.58862	0.4195	3.65551
H	-0.66714	-0.60774	2.24175
H	-2.47497	-3.89279	1.39873
H	-1.54248	-2.76934	2.36851
H	-4.17898	-2.2654	0.92038
H	-3.0279	-0.16566	0.81383
H	-4.43361	-0.12071	1.86091
H	-4.23112	0.56264	4.16184
H	-2.57756	1.08988	4.79568
H	-4.333	-3.67467	2.97211
H	-3.57384	-2.38604	3.92538
H	-5.16212	-2.12578	3.19264
H	-3.49118	0.65486	-4.44854
H	1.87627	3.29405	1.6122
H	2.84536	-1.46243	-0.47233
H	5.14172	1.24796	0.80101
H	5.89506	-1.24985	-2.03535
H	6.15093	-2.08668	-0.51826
H	4.65676	-2.37985	-1.44152
H	6.19892	1.15664	-2.07815
H	6.42733	2.48214	-0.92211
H	4.79205	2.08475	-1.50748
H	6.33729	-1.19651	1.72714
H	8.00136	-0.55772	1.81468
H	6.63215	0.42963	2.39858

Compound 1b: Isomer 2S, 5R, 11S, 13S, 17S, 23S conformer 13

C	2.52474	-2.22618	-0.33131
C	3.43618	-3.0325	-0.93359
C	4.79523	-2.5845	-1.21765
C	5.10478	-1.17023	-0.72714
C	3.86362	-0.28157	-0.75782
O	2.75704	-0.91879	-0.05768
C	1.14936	-2.67946	0.07133
C	0.85807	-2.82493	1.58935
C	0.90988	-1.51725	2.40537
C	-0.1123	-0.45688	1.97752
C	0.07085	0.92034	2.66788
C	-0.67144	2.00072	1.88368
C	-0.35196	0.93443	4.13923
O	-0.22604	2.033	0.59894
O	-1.53275	2.73741	2.30902
C	-0.91342	2.86915	-0.35991
C	-0.0538	2.84336	-1.63887
C	-2.29471	2.33212	-0.60363
C	1.31728	3.50545	-1.53703
C	4.09296	1.08059	-0.10708
C	2.88886	2.04485	-0.05745
C	2.55742	2.62987	-1.45567
C	1.41515	4.83946	-1.58484
C	3.16811	3.16057	0.9595
O	5.645	-3.2841	-1.75304
O	-0.37874	-3.50967	1.76793
C	-3.49433	2.96963	-0.58593
O	-4.46667	2.05502	-0.87645
C	-3.79935	0.87018	-1.04556
N	-2.50488	0.99691	-0.91269
C	-4.63238	-0.29026	-1.3088
C	-4.28184	-1.5931	-1.25069
C	-2.87936	-2.05744	-0.88565
C	-5.29203	-2.66756	-1.56548
C	-2.18177	-2.73302	-2.06693
O	-2.89452	-3.0223	0.18677
C	-3.51595	-2.56072	1.38406
H	3.17994	-4.05943	-1.16841
H	5.48641	-1.2414	0.30201

H	5.90184	-0.74304	-1.34312
H	3.53109	-0.17232	-1.80088
H	0.42038	-1.98631	-0.36821
H	0.9756	-3.66545	-0.37035
H	1.6202	-3.49703	2.00427
H	1.922	-1.10089	2.32801
H	0.75323	-1.79412	3.45486
H	-1.1259	-0.82236	2.18249
H	-0.04062	-0.29923	0.89869
H	1.13711	1.18058	2.59403
H	-0.19399	1.91996	4.58706
H	0.22256	0.20006	4.71231
H	-1.41572	0.69463	4.23944
H	-0.98133	3.88546	0.04031
H	-0.63819	3.35325	-2.41393
H	0.03666	1.79745	-1.95213
H	4.43601	0.89435	0.91967
H	4.93394	1.55925	-0.62942
H	2.02082	1.47846	0.28735
H	3.42243	3.2167	-1.79425
H	2.4439	1.80309	-2.17126
H	2.37531	5.34653	-1.53411
H	0.53925	5.47882	-1.6779
H	3.3459	2.74528	1.95883
H	4.05899	3.73942	0.67969
H	2.32774	3.85725	1.02632
H	-1.02399	-3.24275	1.08621
H	-3.81821	3.97833	-0.38577
H	-5.65979	-0.03452	-1.55782
H	-2.29217	-1.19209	-0.56587
H	-6.30477	-2.2606	-1.63736
H	-5.05897	-3.1571	-2.52056
H	-5.27884	-3.45476	-0.80399
H	-2.73727	-3.61296	-2.40497
H	-1.17918	-3.05936	-1.77637
H	-2.08966	-2.02997	-2.90084
H	-3.15621	-1.56241	1.66663
H	-4.6085	-2.52133	1.28587
H	-3.24762	-3.27334	2.16684

Compound 1b: Isomer 2S, 5R, 11S, 13S, 17S, 23S conformer 15

C	-1.68707	-2.08744	-1.95739
C	-0.40005	-2.4944	-2.10642
C	0.30509	-3.21052	-1.05071
C	-0.54473	-3.50607	0.18294
C	-1.57736	-2.40328	0.39773
O	-2.38448	-2.22907	-0.80538
C	-2.48808	-1.3973	-3.02089
C	-2.77438	0.09791	-2.72225
C	-1.49812	0.94227	-2.64567
C	-1.74663	2.45491	-2.51756
C	-2.43411	2.94472	-1.21426
C	-1.46971	2.9081	-0.03547
C	-2.97064	4.37283	-1.38901
O	-1.47798	1.70158	0.58858
O	-0.77272	3.83574	0.31979
C	-0.63801	1.57799	1.77778
C	-1.19982	0.44371	2.66385
C	0.77927	1.31227	1.36774
C	-2.712	0.40637	2.80006
C	-2.50563	-2.66909	1.58194
C	-3.75694	-1.77628	1.73297
C	-3.52334	-0.23811	1.68609
C	-3.30397	0.94907	3.87113
C	-4.54074	-2.2006	2.98309
O	1.47081	-3.58316	-1.14391
O	-3.57782	0.64311	-3.77004
C	1.8448	2.15286	1.35826
O	2.92307	1.46441	0.8711
C	2.44977	0.20596	0.594
N	1.17745	0.07681	0.87512
C	3.29602	-0.82247	0.02852
C	4.62471	-0.82048	-0.21261
C	5.54499	0.35633	0.06851
C	5.26182	-2.03414	-0.8387
C	5.61352	1.30408	-1.14203
O	6.83286	-0.17973	0.38408
C	7.6701	0.70073	1.10212
H	0.11857	-2.32092	-3.04254

H	-1.0469	-4.4742	0.03855
H	0.10988	-3.59761	1.05467
H	-1.03798	-1.45764	0.51954
H	-1.97343	-1.47075	-3.98383
H	-3.45328	-1.91533	-3.11756
H	-3.31359	0.15784	-1.76569
H	-0.92444	0.77089	-3.56667
H	-0.8814	0.57593	-1.81773
H	-2.37689	2.76852	-3.35578
H	-0.79136	2.98511	-2.62449
H	-3.26731	2.27303	-0.97984
H	-3.463	4.7299	-0.47834
H	-3.69743	4.40161	-2.20745
H	-2.15618	5.06623	-1.61867
H	-0.69642	2.53368	2.30322
H	-0.73997	0.57824	3.65025
H	-0.82149	-0.50504	2.27909
H	-2.84924	-3.71129	1.52385
H	-1.88727	-2.6081	2.48841
H	-4.38804	-1.98393	0.85855
H	-3.06526	0.00775	0.72544
H	-4.51969	0.22279	1.68148
H	-4.3854	0.95989	3.98394
H	-2.73104	1.40646	4.675
H	-4.77513	-3.27153	2.95745
H	-3.97098	-2.00126	3.8978
H	-5.4881	-1.65361	3.05784
H	-4.43094	0.18198	-3.7642
H	1.99654	3.18972	1.6103
H	2.72818	-1.7116	-0.23091
H	5.17327	0.91754	0.93514
H	4.51128	-2.79055	-1.0824
H	5.80139	-1.76549	-1.75629
H	6.00765	-2.46647	-0.16318
H	5.96115	0.76677	-2.03046
H	6.30336	2.13337	-0.9516
H	4.62666	1.72771	-1.35016
H	7.95944	1.587	0.51741
H	7.1946	1.04247	2.03573

H	8.57549	0.14066	1.35195

Compound 1b: Isomer 2S, 5R, 11S, 13S, 17S, 23S conformer 16

C	2.73325	-2.14587	-0.09569
C	3.5902	-2.98828	-0.72683
C	4.82884	-2.51434	-1.33655
C	5.0885	-1.01995	-1.14811
C	3.78475	-0.22796	-1.08282
O	2.88565	-0.79929	-0.0892
C	1.50656	-2.60121	0.64352
C	1.54022	-2.45872	2.18064
C	1.5757	-1.02023	2.726
C	0.3745	-0.15742	2.31642
C	0.50769	1.32986	2.72598
C	-0.42021	2.21649	1.89436
C	0.29217	1.58225	4.2214
O	-0.27419	1.94791	0.57208
O	-1.16742	3.06636	2.32675
C	-1.12733	2.6482	-0.37243
C	-0.49204	2.44485	-1.75978
C	-2.52559	2.11278	-0.30267
C	0.81512	3.19058	-2.0108
C	3.99582	1.23827	-0.71518
C	2.73411	2.12111	-0.61051
C	2.12383	2.41834	-2.00543
C	0.78813	4.49827	-2.29565
C	3.07976	3.41958	0.1328
O	5.63106	-3.24467	-1.90276
O	0.36801	-3.15744	2.61664
C	-3.65899	2.76228	0.06819
O	-4.70554	1.89639	-0.05551
C	-4.14504	0.72223	-0.49402
N	-2.85085	0.81077	-0.6648
C	-5.0737	-0.37083	-0.69535
C	-4.78758	-1.67756	-0.87352
C	-3.37231	-2.22671	-0.90231
C	-5.89859	-2.67287	-1.07767
C	-2.8031	-2.21668	-2.3312

O	-3.42839	-3.55275	-0.37058
C	-2.20237	-4.02268	0.15511
H	3.39407	-4.05451	-0.73636
H	5.65311	-0.88598	-0.21357
H	5.71957	-0.65931	-1.96597
H	3.26242	-0.3247	-2.04575
H	0.63458	-2.06309	0.25522
H	1.35193	-3.66335	0.43399
H	2.43941	-2.98304	2.5439
H	2.5037	-0.54673	2.38455
H	1.64833	-1.0829	3.82232
H	-0.54808	-0.57221	2.74158
H	0.26298	-0.20021	1.23181
H	1.52499	1.65187	2.45355
H	0.41097	2.6417	4.46411
H	1.01067	1.0101	4.81758
H	-0.71869	1.28755	4.52324
H	-1.13746	3.708	-0.10259
H	-1.23505	2.78181	-2.49181
H	-0.37227	1.3672	-1.91415
H	4.52234	1.2581	0.24874
H	4.68926	1.66794	-1.45251
H	1.9893	1.57791	-0.02338
H	2.86579	2.98145	-2.58824
H	1.96633	1.46948	-2.53747
H	1.69576	5.06034	-2.50065
H	-0.14372	5.05888	-2.33776
H	3.45694	3.20776	1.14074
H	3.85794	3.98447	-0.39838
H	2.20551	4.07001	0.22699
H	0.32396	-3.08884	3.58341
H	-3.8799	3.75231	0.4333
H	-6.11561	-0.06103	-0.67835
H	-2.72926	-1.60145	-0.26879
H	-6.87638	-2.18278	-1.09412
H	-5.76715	-3.21963	-2.02052
H	-5.88552	-3.42654	-0.28396
H	-1.8033	-2.6639	-2.34998
H	-2.7214	-1.18858	-2.69492

H	-3.44517	-2.78942	-3.00884
H	-1.42138	-4.11497	-0.61526
H	-1.82215	-3.37652	0.95972
H	-2.39869	-5.01724	0.56532

Compound 1b: Isomer 2S, 5R, 11S, 13S, 17S, 23S conformer 17

C	2.25333	-2.15792	0.52584
C	2.56711	-3.4029	0.09215
C	3.55226	-3.61461	-0.96573
C	4.22171	-2.33337	-1.47338
C	3.26868	-1.14449	-1.39709
O	2.7152	-1.02961	-0.05962
C	1.35207	-1.81898	1.67828
C	2.05392	-0.95666	2.75615
C	1.07079	-0.21217	3.67588
C	0.20625	0.86681	2.99715
C	0.9989	2.07588	2.42122
C	0.1315	2.7727	1.38068
C	1.45239	3.05912	3.50402
O	0.08607	2.04155	0.23826
O	-0.46852	3.81484	1.53871
C	-0.85308	2.477	-0.79356
C	-0.40101	1.82625	-2.10727
C	-2.24444	2.07747	-0.39913
C	0.84949	2.41948	-2.75145
C	3.90926	0.20567	-1.7075
C	2.94635	1.41235	-1.62702
C	2.06176	1.5199	-2.89789
C	0.84537	3.67237	-3.22098
C	3.73191	2.71015	-1.39435
O	3.87324	-4.71878	-1.38455
O	2.97484	-1.73738	3.51387
C	-3.20307	2.83261	0.19994
O	-4.3093	2.05438	0.37584
C	-3.96445	0.82529	-0.12946
N	-2.74662	0.79799	-0.60576
C	-4.99815	-0.18789	-0.05915
C	-4.89244	-1.50115	-0.35392

C	-3.6065	-2.15887	-0.84075
C	-6.10015	-2.39638	-0.25044
C	-3.56226	-2.23984	-2.36876
O	-3.49526	-3.49565	-0.34812
C	-3.08243	-3.56609	1.00172
H	2.1388	-4.27437	0.57425
H	5.10741	-2.14207	-0.85011
H	4.56711	-2.49224	-2.49933
H	2.41207	-1.33401	-2.06189
H	0.49664	-1.26587	1.27012
H	0.96545	-2.73776	2.1355
H	2.68755	-0.22787	2.2451
H	1.66448	0.22873	4.48557
H	0.39976	-0.94588	4.14942
H	-0.52049	1.24881	3.72575
H	-0.38149	0.41032	2.19335
H	1.87166	1.69154	1.88071
H	2.04966	3.87072	3.07635
H	2.06277	2.55198	4.25832
H	0.58767	3.51058	3.99986
H	-0.79321	3.56582	-0.85289
H	-1.23834	1.91678	-2.80965
H	-0.27854	0.75454	-1.92298
H	4.73214	0.34823	-0.99475
H	4.36631	0.15165	-2.70561
H	2.2879	1.25597	-0.76604
H	2.68832	1.87994	-3.7251
H	1.71631	0.5167	-3.18566
H	1.71413	4.10157	-3.71334
H	-0.02641	4.31856	-3.13756
H	4.28977	2.66855	-0.45139
H	4.45632	2.88424	-2.2015
H	3.06261	3.57537	-1.35363
H	2.46293	-2.38444	4.02625
H	-3.25	3.84908	0.5551
H	-5.95857	0.20142	0.27004
H	-2.74527	-1.5701	-0.50184
H	-6.99224	-1.83321	0.0384
H	-6.3001	-2.89307	-1.20852

H	-5.9407	-3.19953	0.47728
H	-2.64639	-2.75252	-2.67875
H	-3.56936	-1.234	-2.79834
H	-4.41728	-2.80103	-2.76002
H	-3.00759	-4.62656	1.25742
H	-2.09806	-3.09279	1.14729
H	-3.79802	-3.08487	1.68667

Compound 1b: Isomer 2S, 5R, 11S, 13S, 17S, 23S conformer 18

C	3.96007	0.4291	1.58591
C	3.86152	-0.06145	2.84865
C	3.88168	-1.49602	3.11858
C	4.07381	-2.35945	1.87108
C	3.41123	-1.71435	0.6588
O	3.8941	-0.34677	0.479
C	4.21155	1.87665	1.26538
C	3.17432	2.58402	0.36151
C	1.76464	2.5663	0.95629
C	0.73272	3.45785	0.23844
C	0.67352	3.36044	-1.30462
C	0.60327	1.92257	-1.79058
C	-0.49524	4.19392	-1.86046
O	-0.53201	1.30168	-1.39449
O	1.47125	1.37444	-2.44518
C	-0.62095	-0.12393	-1.70434
C	0.13658	-0.89399	-0.60337
C	-2.0703	-0.47331	-1.78668
C	0.49099	-2.32572	-0.95066
C	3.66916	-2.45243	-0.65256
C	2.90941	-1.93193	-1.89606
C	1.49785	-2.55773	-2.06157
C	-0.06929	-3.34684	-0.29132
C	3.72791	-2.21531	-3.16666
O	3.82342	-1.97929	4.24159
O	3.5517	3.95719	0.20982
C	-2.79234	-0.83599	-2.87916
O	-4.07367	-1.08323	-2.48148
C	-4.075	-0.84771	-1.12834

N	-2.90093	-0.49511	-0.67465
C	-5.34789	-1.03924	-0.46313
C	-5.6703	-0.7186	0.80826
C	-4.69729	-0.08154	1.79392
C	-7.04876	-1.02215	1.33527
C	-4.02635	-1.13438	2.68008
O	-5.37169	0.82798	2.66527
C	-5.65678	2.07697	2.06518
H	3.87728	0.61596	3.69495
H	5.15296	-2.48147	1.69557
H	3.65421	-3.35314	2.05365
H	2.33606	-1.63414	0.85681
H	4.29524	2.43964	2.20015
H	5.18926	1.93701	0.76308
H	3.16023	2.09026	-0.61809
H	1.8169	2.88607	2.00556
H	1.41742	1.52606	0.97245
H	0.94624	4.50548	0.476
H	-0.25936	3.23116	0.64784
H	1.61292	3.74851	-1.70871
H	-0.50954	4.1816	-2.95607
H	-0.39665	5.23603	-1.53704
H	-1.45571	3.8113	-1.50138
H	-0.14747	-0.27474	-2.6765
H	-0.46793	-0.86056	0.30786
H	1.0572	-0.33405	-0.41257
H	4.75009	-2.40297	-0.83887
H	3.43198	-3.51299	-0.49086
H	2.80382	-0.84422	-1.81538
H	1.08543	-2.16621	-3.00358
H	1.61127	-3.64019	-2.21065
H	0.18152	-4.3806	-0.51791
H	-0.80554	-3.18559	0.4921
H	4.70715	-1.72553	-3.12049
H	3.89823	-3.29226	-3.29861
H	3.21179	-1.84637	-4.0603
H	4.37686	3.98396	-0.29919
H	-2.57203	-0.9691	-3.92659
H	-6.10174	-1.49736	-1.09889

H	-3.91519	0.45147	1.23974
H	-7.65829	-1.53915	0.5885
H	-6.99263	-1.65176	2.23236
H	-7.56796	-0.10735	1.64097
H	-4.76856	-1.70775	3.2455
H	-3.36247	-0.63724	3.394
H	-3.43696	-1.8222	2.06704
H	-6.13703	2.6947	2.82895
H	-4.73833	2.57897	1.72346
H	-6.33731	1.98401	1.20448

Compound 1b: Isomer 2S, 5R, 11S, 13S, 17S, 23S conformer 19

C	1.56184	-2.51281	1.76967
C	0.25202	-2.88483	1.78961
C	-0.43976	-3.32442	0.58516
C	0.45091	-3.41465	-0.6504
C	1.54476	-2.34907	-0.61257
O	2.29514	-2.42937	0.63782
C	2.3137	-2.06254	2.99034
C	1.99117	-0.58589	3.37294
C	2.35095	0.40993	2.26318
C	2.1337	1.87337	2.66761
C	2.58182	2.92699	1.6177
C	1.63103	3.02216	0.42614
C	2.74277	4.31161	2.25788
O	1.65383	1.89425	-0.3299
O	0.94789	3.98712	0.15497
C	0.83654	1.89767	-1.53987
C	1.38766	0.82463	-2.50661
C	-0.5967	1.6341	-1.1843
C	2.90098	0.72848	-2.58883
C	2.52116	-2.46037	-1.78321
C	3.80873	-1.60781	-1.74515
C	3.62884	-0.07721	-1.52299
C	3.56337	1.3559	-3.56831
C	4.65462	-1.90685	-2.99177
O	-1.62713	-3.63438	0.55699
O	0.63647	-0.44828	3.78683

C	-1.63784	2.50118	-1.10068
O	-2.74644	1.79681	-0.71691
C	-2.31817	0.5014	-0.57688
N	-1.04306	0.36433	-0.8422
C	-3.20498	-0.56113	-0.15177
C	-4.53892	-0.55355	0.06075
C	-5.44435	0.66107	-0.1207
C	-5.20802	-1.81212	0.55483
C	-5.68887	1.38716	1.20518
O	-6.7262	0.28007	-0.62243
C	-6.72146	-0.03118	-2.00228
H	-0.28145	-2.92091	2.73312
H	0.8995	-4.41862	-0.6829
H	-0.16641	-3.29813	-1.54594
H	1.05852	-1.36681	-0.59124
H	2.04166	-2.68966	3.84478
H	3.38866	-2.16823	2.80869
H	2.57628	-0.35849	4.27243
H	1.75369	0.18157	1.37464
H	3.40093	0.2575	1.97701
H	2.70672	2.07395	3.58335
H	1.08163	2.0338	2.93116
H	3.55072	2.60236	1.21223
H	3.0508	5.05861	1.52076
H	3.49788	4.2729	3.05018
H	1.79888	4.65139	2.69555
H	0.93098	2.89264	-1.98052
H	0.97331	1.06624	-3.49271
H	0.95706	-0.13701	-2.22195
H	2.82608	-3.51218	-1.87259
H	1.94818	-2.23651	-2.69383
H	4.37775	-1.94785	-0.86958
H	3.13422	0.07322	-0.56159
H	4.64035	0.33573	-1.41594
H	4.64797	1.32471	-3.63804
H	3.04708	1.92772	-4.33647
H	4.85462	-2.98088	-3.08599
H	4.14909	-1.57559	-3.90602
H	5.62102	-1.39106	-2.94317

H	0.07936	-0.6266	3.00913
H	-1.75297	3.56436	-1.23457
H	-2.66362	-1.48803	0.01726
H	-4.98448	1.36443	-0.82532
H	-5.96641	-2.16512	-0.1528
H	-4.47782	-2.60896	0.71844
H	-5.73869	-1.62637	1.49781
H	-6.15456	0.7192	1.93677
H	-6.36145	2.23453	1.03912
H	-4.74528	1.75735	1.617
H	-6.07424	-0.89008	-2.23627
H	-7.7506	-0.27889	-2.27596
H	-6.38702	0.82648	-2.60751

Compound 1b: Isomer 2S, 5R, 11S, 13S, 17S, 23S conformer 21

C	-4.67497	0.3207	-1.17096
C	-5.77745	-0.12428	-1.8259
C	-6.42225	-1.38791	-1.48305
C	-5.77763	-2.11453	-0.30299
C	-4.27519	-1.85294	-0.2523
O	-4.00047	-0.42144	-0.25801
C	-4.08365	1.68715	-1.37135
C	-4.14789	2.59368	-0.11591
C	-2.86976	2.59166	0.74333
C	-1.65096	3.23178	0.06177
C	-0.41607	3.36848	0.97933
C	0.02939	2.00763	1.49587
C	0.73639	4.09559	0.26377
O	0.43631	1.20691	0.48282
O	0.00271	1.66239	2.66026
C	0.7831	-0.17117	0.8378
C	0.45431	-1.02458	-0.39981
C	2.21696	-0.23813	1.2711
C	0.1896	-2.48668	-0.07008
C	-3.6028	-2.45474	0.97834
C	-2.08636	-2.21822	1.12963
C	-1.26523	-2.90678	0.01051
C	1.19056	-3.35582	0.10624

C	-1.6288	-2.69009	2.51817
O	-7.42648	-1.81377	-2.03757
O	-4.44269	3.9052	-0.60946
C	2.71639	-0.13045	2.53131
O	4.07525	-0.22182	2.46018
C	4.35217	-0.37923	1.12499
N	3.2739	-0.40139	0.38484
C	5.75673	-0.5031	0.79043
C	6.32035	-0.4975	-0.43645
C	5.5289	-0.35962	-1.73192
C	7.81085	-0.66603	-0.58121
C	5.23761	-1.72382	-2.36243
O	6.25787	0.39744	-2.70029
C	6.22669	1.79209	-2.46507
H	-6.25027	0.50144	-2.57423
H	-6.25253	-1.75876	0.62312
H	-5.9805	-3.18656	-0.38562
H	-3.82339	-2.24333	-1.17558
H	-3.04771	1.58732	-1.71694
H	-4.64706	2.19188	-2.15965
H	-4.98283	2.2469	0.51315
H	-2.65639	1.55806	1.04245
H	-3.08501	3.13729	1.67503
H	-1.92711	4.23063	-0.29474
H	-1.3602	2.65587	-0.82464
H	-0.69661	3.93284	1.87605
H	1.60345	4.21373	0.92296
H	0.41224	5.09377	-0.05049
H	1.05651	3.54191	-0.62404
H	0.15192	-0.44291	1.68447
H	1.27718	-0.93204	-1.11418
H	-0.43518	-0.58408	-0.86308
H	-4.1111	-2.05636	1.86679
H	-3.80628	-3.53521	0.96432
H	-1.92574	-1.13517	1.06128
H	-1.32859	-3.99551	0.14121
H	-1.72718	-2.68184	-0.96201
H	1.00251	-4.40006	0.34487
H	2.23029	-3.05459	0.01123

H	-2.13599	-2.13319	3.31432
H	-1.85201	-3.75582	2.65941
H	-0.54942	-2.56282	2.64955
H	-4.5279	4.49425	0.15737
H	2.27858	0.02038	3.50455
H	6.39722	-0.60998	1.66267
H	4.5731	0.13594	-1.52244
H	8.2921	-0.82121	0.38874
H	8.04628	-1.52341	-1.22466
H	8.26489	0.2065	-1.06341
H	4.6254	-2.32939	-1.68798
H	6.1648	-2.2633	-2.58243
H	4.69461	-1.58099	-3.30183
H	6.78337	2.2645	-3.27904
H	5.19535	2.17778	-2.46506
H	6.69595	2.0673	-1.5077

Compound 1b: Isomer 2S, 5R, 11S, 13S, 17S, 23S conformer 22

C	0.43668	2.33816	-1.28348
C	-0.1082	3.56324	-1.48873
C	0.38289	4.7485	-0.78982
C	1.60612	4.5047	0.10022
C	1.61686	3.09249	0.68017
O	1.40845	2.10916	-0.36823
C	0.04342	1.06597	-1.98907
C	1.24681	0.17385	-2.35913
C	0.81632	-1.22332	-2.80493
C	1.97729	-2.22538	-2.9319
C	2.83136	-2.46872	-1.65467
C	1.95545	-2.91559	-0.49121
C	3.92273	-3.51279	-1.9247
O	1.47942	-1.85775	0.21057
O	1.68563	-4.06981	-0.22477
C	0.5424	-2.14314	1.30132
C	0.50595	-0.88306	2.1676
C	-0.81585	-2.49103	0.75252
C	1.73587	-0.58146	3.01485
C	2.92778	2.71591	1.37354

C	3.05348	1.23658	1.79903
C	2.16258	0.87395	3.02179
C	2.36725	-1.511	3.73899
C	4.52025	0.88251	2.08174
O	-0.08932	5.86752	-0.93722
O	2.01649	0.74076	-3.42319
C	-1.26703	-3.68933	0.29104
O	-2.566	-3.53431	-0.09439
C	-2.8621	-2.21878	0.1572
N	-1.84765	-1.5623	0.65949
C	-4.221	-1.82132	-0.15275
C	-4.81339	-0.62778	0.06609
C	-4.12385	0.55124	0.74494
C	-6.26131	-0.42887	-0.29934
C	-4.33539	0.50932	2.26197
O	-4.65163	1.79537	0.2876
C	-4.11854	2.2157	-0.95389
H	-0.89548	3.69217	-2.22306
H	2.50503	4.66285	-0.51398
H	1.62215	5.25292	0.89836
H	0.76339	2.98203	1.36524
H	-0.60248	0.49829	-1.30324
H	-0.54108	1.3022	-2.8842
H	1.86454	0.07841	-1.46201
H	0.31759	-1.13927	-3.77949
H	0.06956	-1.60178	-2.09833
H	2.66168	-1.87882	-3.71438
H	1.575	-3.1914	-3.26365
H	3.3009	-1.52222	-1.3665
H	4.57298	-3.6448	-1.05337
H	4.54431	-3.19951	-2.77026
H	3.47782	-4.48434	-2.15874
H	0.94004	-3.00317	1.84377
H	-0.36753	-0.95834	2.82805
H	0.29312	-0.0398	1.50324
H	3.74758	2.95357	0.6819
H	3.05462	3.37625	2.24304
H	2.72364	0.62567	0.95205
H	2.70856	1.10855	3.94445

H	1.26091	1.50207	3.02871
H	3.20754	-1.25693	4.37995
H	2.07248	-2.55775	3.73312
H	5.1429	1.0262	1.19073
H	4.93234	1.5102	2.88299
H	4.61443	-0.16361	2.39164
H	2.45852	1.53215	-3.07865
H	-0.8221	-4.66187	0.1637
H	-4.80807	-2.62467	-0.59109
H	-3.04654	0.51063	0.54455
H	-6.70777	-1.35174	-0.68075
H	-6.84087	-0.0942	0.57013
H	-6.37675	0.35432	-1.05688
H	-5.40174	0.52974	2.51056
H	-3.8617	1.38387	2.7187
H	-3.89052	-0.39729	2.68219
H	-3.02885	2.36124	-0.89449
H	-4.33026	1.50106	-1.76479
H	-4.58973	3.17227	-1.19496

Compound 1b: Isomer 2S, 5R, 11S, 13S, 17S, 23S conformer 23

C	-1.68796	-2.1051	-1.93937
C	-0.40109	-2.51587	-2.08292
C	0.30374	-3.21837	-1.01842
C	-0.54775	-3.50016	0.21695
C	-1.57946	-2.39413	0.42001
O	-2.38421	-2.22713	-0.78584
C	-2.48977	-1.4333	-3.01447
C	-2.77909	0.06653	-2.74655
C	-1.50164	0.91728	-2.64358
C	-1.76057	2.42952	-2.53207
C	-2.44332	2.92523	-1.2292
C	-1.47061	2.903	-0.05639
C	-2.98968	4.34856	-1.41156
O	-1.47523	1.70381	0.58
O	-0.77282	3.83548	0.28406
C	-0.63578	1.59254	1.77082
C	-1.1993	0.4666	2.66639

C	0.78162	1.3237	1.36367
C	-2.71195	0.43238	2.80099
C	-2.51047	-2.65056	1.60428
C	-3.76077	-1.75528	1.74745
C	-3.52419	-0.21807	1.69098
C	-3.30333	0.9837	3.868
C	-4.54689	-2.17028	2.99922
O	1.4701	-3.59089	-1.10619
O	-3.67083	0.55946	-3.74745
C	1.8486	2.16243	1.35341
O	2.92646	1.47076	0.86977
C	2.45134	0.2123	0.59556
N	1.17839	0.0862	0.87513
C	3.29646	-0.81945	0.03447
C	4.62489	-0.81944	-0.20818
C	5.54605	0.35858	0.06514
C	5.26066	-2.03696	-0.82808
C	5.61102	1.3012	-1.14966
O	6.83497	-0.17581	0.37884
C	7.67364	0.7072	1.09216
H	0.11754	-2.35832	-3.02198
H	-1.05091	-4.46906	0.08163
H	0.1054	-3.58361	1.09057
H	-1.03926	-1.44821	0.53586
H	-1.9619	-1.5343	-3.97082
H	-3.45876	-1.93731	-3.11193
H	-3.35014	0.13984	-1.81631
H	-0.89681	0.73971	-3.54704
H	-0.89652	0.56142	-1.80212
H	-2.40539	2.7299	-3.36537
H	-0.8131	2.96989	-2.65472
H	-3.27096	2.25033	-0.98585
H	-3.47672	4.709	-0.49953
H	-3.72457	4.36594	-2.22299
H	-2.18159	5.04554	-1.653
H	-0.69516	2.55334	2.28683
H	-0.74045	0.60953	3.65208
H	-0.82138	-0.48589	2.29045
H	-2.85521	-3.69267	1.55215

H	-1.89344	-2.58462	2.51136
H	-4.39086	-1.96706	0.87352
H	-3.06558	0.01975	0.72869
H	-4.51947	0.2447	1.68239
H	-4.38478	0.99741	3.98007
H	-2.72991	1.44533	4.66913
H	-4.78318	-3.24092	2.97993
H	-3.97818	-1.96618	3.91361
H	-5.49343	-1.62131	3.06866
H	-3.19339	0.55026	-4.59373
H	2.00204	3.19959	1.60327
H	2.72784	-1.70977	-0.21921
H	5.1769	0.92353	0.93043
H	5.79924	-1.77396	-1.7479
H	6.00724	-2.46561	-0.15105
H	4.50949	-2.79454	-1.06614
H	5.95539	0.75989	-2.03695
H	6.30211	2.13074	-0.96496
H	4.62375	1.7247	-1.35618
H	7.20059	1.05099	2.02623
H	8.58017	0.14847	1.34078
H	7.96059	1.59215	0.50426

Compound 1b: Isomer 2S, 5R, 11S, 13S, 17S, 23S conformer 27

C	0.38974	2.33013	-1.2863
C	-0.14154	3.56238	-1.48836
C	0.39069	4.74661	-0.81843
C	1.64257	4.49282	0.0277
C	1.64831	3.08841	0.62628
O	1.38596	2.0953	-0.39979
C	-0.04651	1.05674	-1.96289
C	1.13207	0.15689	-2.39197
C	0.67656	-1.25196	-2.77183
C	1.82849	-2.25737	-2.9424
C	2.74542	-2.48408	-1.70608
C	1.92973	-2.92635	-0.49784
C	3.82982	-3.52306	-2.01998
O	1.46549	-1.86458	0.20735

O	1.69248	-4.08009	-0.20117
C	0.56824	-2.15004	1.33113
C	0.53895	-0.88054	2.18446
C	-0.79832	-2.52757	0.82837
C	1.78452	-0.56292	3.00345
C	2.97453	2.69644	1.28049
C	3.08715	1.22037	1.72019
C	2.22107	0.88889	2.96886
C	2.42105	-1.47868	3.74066
C	4.55463	0.84641	1.97146
O	-0.07329	5.87056	-0.95585
O	1.8247	0.69733	-3.52075
C	-1.25735	-3.75476	0.46271
O	-2.55927	-3.62465	0.07913
C	-2.84817	-2.29187	0.23078
N	-1.82725	-1.60288	0.67442
C	-4.20443	-1.91408	-0.10923
C	-4.78389	-0.69794	-0.02116
C	-4.07522	0.52829	0.52759
C	-6.22246	-0.518	-0.42595
C	-4.19292	0.57457	2.06206
O	-4.67166	1.66996	-0.08849
C	-3.91737	2.86009	0.02483
H	-0.9513	3.69519	-2.19714
H	2.5205	4.62563	-0.62178
H	1.70242	5.25103	0.81428
H	0.81698	3.00337	1.34174
H	-0.65655	0.49615	-1.23965
H	-0.67768	1.28971	-2.82642
H	1.81104	0.08653	-1.53733
H	0.11918	-1.19317	-3.7159
H	-0.02472	-1.60997	-2.01062
H	2.47274	-1.92351	-3.76365
H	1.40956	-3.22763	-3.23942
H	3.22182	-1.53205	-1.44862
H	4.52127	-3.64473	-1.17941
H	4.40852	-3.21204	-2.89624
H	3.38106	-4.49922	-2.22577
H	0.99563	-2.99667	1.87182

H	-0.31956	-0.9577	2.86428
H	0.3053	-0.04608	1.51614
H	3.77546	2.91081	0.55979
H	3.14162	3.36418	2.1375
H	2.72649	0.60456	0.8894
H	2.78849	1.13941	3.87432
H	1.32355	1.52288	2.98174
H	3.27425	-1.21534	4.36045
H	2.11807	-2.52282	3.76641
H	5.15732	0.97023	1.06392
H	4.99585	1.47661	2.75508
H	4.64003	-0.19745	2.29122
H	2.27176	1.50865	-3.23378
H	-0.81597	-4.73547	0.40225
H	-4.80055	-2.74918	-0.46786
H	-3.0104	0.48404	0.26355
H	-6.67067	-1.46565	-0.73814
H	-6.81498	-0.10501	0.40046
H	-6.30103	0.20414	-1.24526
H	-5.24427	0.58871	2.36872
H	-3.70652	1.46951	2.46419
H	-3.70486	-0.29952	2.50239
H	-3.87888	3.24147	1.05573
H	-2.88484	2.72409	-0.33294
H	-4.41262	3.60703	-0.60128

Compound 1b: Isomer 2S, 5R, 11S, 13S, 17S, 23S conformer 28

C	2.92299	-2.16422	-0.65628
C	3.92555	-2.81814	-1.2965
C	5.27679	-2.2721	-1.37443
C	5.46152	-0.94772	-0.63318
C	4.17821	-0.12104	-0.63971
O	3.056	-0.91139	-0.15556
C	1.55044	-2.73739	-0.44529
C	1.19181	-3.12413	1.00622
C	1.06116	-1.96031	2.00439
C	-0.02102	-0.93103	1.64909
C	-0.02467	0.31043	2.57641

C	-0.7326	1.48983	1.91029
C	-0.61951	0.04258	3.96219
O	-0.20127	1.7257	0.68223
O	-1.62186	2.1546	2.39203
C	-0.7916	2.74977	-0.14467
C	0.14614	2.90688	-1.36105
C	-2.18992	2.37202	-0.55214
C	1.48058	3.59579	-1.09017
C	4.26522	1.12695	0.2348
C	3.00546	2.01543	0.31224
C	2.74922	2.76341	-1.02206
C	1.52514	4.92919	-0.98009
C	3.14699	2.99775	1.48392
O	6.20872	-2.838	-1.93031
O	-0.03426	-3.84986	0.8739
C	-3.25442	3.19059	-0.75937
O	-4.30296	2.42459	-1.18245
C	-3.82075	1.14207	-1.19586
N	-2.56116	1.06878	-0.84817
C	-4.77802	0.12348	-1.58587
C	-4.72503	-1.20189	-1.33742
C	-3.60763	-1.8722	-0.54813
C	-5.82322	-2.10575	-1.83652
C	-2.6603	-2.67661	-1.43897
O	-4.14928	-2.78725	0.41158
C	-4.74986	-2.14982	1.52281
H	3.74945	-3.80247	-1.71537
H	5.75483	-1.17332	0.40272
H	6.28369	-0.39065	-1.09235
H	3.93137	0.13969	-1.67941
H	0.80269	-2.03079	-0.82248
H	1.46171	-3.65084	-1.03984
H	1.98212	-3.79873	1.37546
H	2.03439	-1.46064	2.07769
H	0.86492	-2.39546	2.99621
H	-1.01082	-1.40361	1.67104
H	0.13552	-0.58958	0.62454
H	1.02155	0.63326	2.69176
H	-0.58998	0.94015	4.58614

H	-0.06539	-0.75087	4.47433
H	-1.66743	-0.2659	3.88048
H	-0.82876	3.68431	0.42478
H	-0.4065	3.49382	-2.10321
H	0.29303	1.91096	-1.79449
H	4.5259	0.79601	1.24942
H	5.11778	1.72274	-0.12213
H	2.14711	1.36828	0.51058
H	3.60905	3.41961	-1.21553
H	2.72585	2.03218	-1.84228
H	2.45755	5.45986	-0.80543
H	0.63118	5.54444	-1.0629
H	3.2767	2.46153	2.43181
H	4.0212	3.64964	1.35123
H	2.26641	3.63993	1.5754
H	-0.36192	-4.05729	1.76306
H	-3.43497	4.24884	-0.65738
H	-5.63763	0.52334	-2.11943
H	-3.02503	-1.10326	-0.02683
H	-6.60666	-1.5418	-2.35099
H	-5.42603	-2.85323	-2.53578
H	-6.27217	-2.66855	-1.01134
H	-1.88949	-3.14222	-0.81767
H	-2.18278	-2.01614	-2.16999
H	-3.19723	-3.46633	-1.97503
H	-5.07574	-2.94029	2.20474
H	-4.03656	-1.49436	2.04742
H	-5.62358	-1.54362	1.23893

Compound 1b: Isomer 2S, 5R, 11S, 13S, 17S, 23S conformer 32

C	-0.44416	2.35696	1.25478
C	0.08458	3.59268	1.43546
C	-0.43793	4.75886	0.73062
C	-1.67674	4.47974	-0.12614
C	-1.66457	3.06499	-0.69972
O	-1.40566	2.0895	0.34286
C	-0.01282	1.10579	1.97479
C	-1.18551	0.22024	2.41671

C	-0.73517	-1.20252	2.78148
C	-1.8874	-2.20978	2.94673
C	-2.7792	-2.45685	1.69565
C	-1.92838	-2.91601	0.51702
C	-3.86797	-3.49443	1.99822
O	-1.46902	-1.8658	-0.20227
O	-1.66569	-4.07406	0.26101
C	-0.54284	-2.15956	-1.30258
C	-0.52125	-0.90851	-2.18185
C	0.82122	-2.49663	-0.76324
C	-1.76336	-0.62313	-3.01719
C	-2.98356	2.65611	-1.35934
C	-3.08724	1.17315	-1.77606
C	-2.21421	0.82453	-3.01535
C	-2.38474	-1.56166	-3.73851
C	-4.55149	0.78691	-2.02808
O	0.01824	5.88847	0.84933
O	-1.80458	0.88408	3.52526
C	1.2872	-3.6929	-0.31172
O	2.58508	-3.52574	0.07201
C	2.86526	-2.20458	-0.17046
N	1.84213	-1.5565	-0.66575
C	4.21908	-1.79277	0.14273
C	4.79959	-0.59343	-0.07673
C	4.10212	0.576	-0.76448
C	6.24312	-0.37787	0.29608
C	4.31442	0.52226	-2.28122
O	4.62269	1.82652	-0.31781
C	4.07734	2.26179	0.91364
H	0.87147	3.74568	2.16541
H	-2.56207	4.61512	0.51223
H	-1.73345	5.22253	-0.92754
H	-0.8267	2.97534	-1.40726
H	0.61305	0.52436	1.28362
H	0.59998	1.37011	2.84273
H	-1.88667	0.16856	1.57791
H	-0.17113	-1.15294	3.72181
H	-0.04342	-1.56706	2.01424
H	-2.54988	-1.88373	3.76129

H	-1.4729	-3.17462	3.26538
H	-3.24998	-1.51141	1.40423
H	-4.53804	-3.62927	1.14271
H	-4.47044	-3.17572	2.85587
H	-3.42072	-4.46591	2.22756
H	-0.94444	-3.02644	-1.83082
H	0.34471	-0.98871	-2.85154
H	-0.30441	-0.05772	-1.52871
H	-3.79047	2.87797	-0.64792
H	-3.1468	3.30924	-2.22834
H	-2.72706	0.57382	-0.93316
H	-2.78102	1.04975	-3.9278
H	-1.3232	1.46717	-3.0385
H	-3.23582	-1.32079	-4.37024
H	-2.07028	-2.60281	-3.73983
H	-5.15916	0.92238	-1.12548
H	-4.9925	1.40254	-2.8233
H	-4.63041	-0.26227	-2.33237
H	-2.68123	0.49334	3.6573
H	0.85418	-4.67166	-0.19049
H	4.81302	-2.58899	0.58476
H	3.0251	0.53021	-0.56388
H	6.69818	-1.29521	0.68069
H	6.82321	-0.03655	-0.57038
H	6.34482	0.40719	1.05365
H	5.3808	0.54827	-2.52924
H	3.83475	1.3896	-2.7453
H	3.8764	-0.39101	-2.69413
H	2.98873	2.40807	0.84154
H	4.28084	1.55645	1.73481
H	4.54714	3.22065	1.14811

Compound 1b: Isomer 2S, 5R, 11S, 13S, 17S, 23S conformer 37

C	1.70988	-2.55687	1.65043
C	0.41912	-2.99071	1.65189
C	-0.25594	-3.39867	0.42664
C	0.63346	-3.38097	-0.81296
C	1.67855	-2.27102	-0.71992

O	2.4356	-2.38176	0.52397
C	2.44252	-2.13239	2.89194
C	2.04778	-0.69488	3.34869
C	2.35555	0.37229	2.29062
C	2.06138	1.80086	2.76475
C	2.45484	2.92441	1.76676
C	1.50212	3.02795	0.57717
C	2.54478	4.28393	2.47137
O	1.58329	1.94077	-0.23253
O	0.77249	3.9699	0.34945
C	0.7772	1.96776	-1.44978
C	1.38891	0.9751	-2.46461
C	-0.64496	1.61594	-1.1284
C	2.90564	0.96142	-2.53878
C	2.65433	-2.28005	-1.89608
C	3.90625	-1.37864	-1.81875
C	3.6689	0.13102	-1.51817
C	3.53983	1.68216	-3.47161
C	4.75604	-1.57929	-3.08234
O	-1.42695	-3.76353	0.38452
O	0.68911	-0.64579	3.76928
C	-1.73104	2.42349	-1.02641
O	-2.80965	1.64457	-0.70408
C	-2.31676	0.36674	-0.61808
N	-1.03088	0.30929	-0.85947
C	-3.16024	-0.75868	-0.27854
C	-4.49817	-0.82469	-0.10675
C	-5.43661	0.3653	-0.22336
C	-5.12827	-2.14271	0.26284
C	-5.587	1.08528	1.1285
O	-6.6939	-0.13124	-0.69064
C	-7.51825	0.84618	-1.28835
H	-0.10927	-3.10103	2.59251
H	1.12646	-4.36062	-0.89931
H	0.0088	-3.24459	-1.70056
H	1.14882	-1.31425	-0.64801
H	2.20502	-2.81466	3.71382
H	3.5208	-2.17482	2.70431
H	2.62315	-0.48392	4.25845

H	1.77258	0.15847	1.38917
H	3.41246	0.28924	2.00147
H	2.62247	1.98776	3.69074
H	1.00216	1.89296	3.03234
H	3.43933	2.66908	1.3493
H	2.81768	5.07866	1.77123
H	3.29843	4.2457	3.26507
H	1.58377	4.55605	2.91896
H	0.82656	2.98794	-1.83686
H	0.9687	1.24555	-3.4407
H	1.00599	-0.02022	-2.23302
H	3.00316	-3.31215	-2.0394
H	2.06846	-2.03575	-2.7931
H	4.4932	-1.73987	-0.96371
H	3.17447	0.21476	-0.54818
H	4.66468	0.57619	-1.39475
H	4.62497	1.71231	-3.53353
H	2.99841	2.27342	-4.20709
H	4.99307	-2.63885	-3.23575
H	4.23465	-1.21675	-3.97563
H	5.70411	-1.03324	-3.00902
H	0.13925	-0.81668	2.98474
H	-1.89889	3.48466	-1.11163
H	-2.58341	-1.67113	-0.15677
H	-5.04185	1.0764	-0.9601
H	-5.83613	-2.4581	-0.51105
H	-4.3693	-2.91764	0.39796
H	-5.71004	-2.05374	1.18966
H	-5.95691	0.39327	1.89205
H	-6.2935	1.91865	1.05025
H	-4.62419	1.48898	1.45518
H	-7.01039	1.34701	-2.12846
H	-8.40009	0.32404	-1.6697
H	-7.85033	1.61696	-0.57664

Compound 1b: Isomer 2S, 5R, 11S, 13S, 17S, 23S conformer 42

C	1.55529	-2.22485	0.98573
C	1.40653	-3.56401	0.82897

C	2.11474	-4.29077	-0.22214
C	3.07546	-3.43073	-1.04976
C	2.56121	-2.00072	-1.18289
O	2.25426	-1.45253	0.12638
C	0.96037	-1.38926	2.08364
C	2.01439	-0.54969	2.84178
C	1.4012	0.58565	3.68029
C	0.67858	1.68859	2.88542
C	1.60473	2.5447	1.9703
C	0.75951	3.11402	0.83955
C	2.32237	3.65648	2.74078
O	0.53552	2.16611	-0.10467
O	0.30055	4.23604	0.79232
C	-0.44079	2.48542	-1.14624
C	-0.17143	1.52655	-2.30922
C	-1.82417	2.33094	-0.58514
C	1.11968	1.75627	-3.08901
C	3.54116	-1.02386	-1.8278
C	3.00455	0.42016	-1.95822
C	2.06988	0.57912	-3.18919
C	1.37094	2.9213	-3.69603
C	4.16148	1.42652	-2.0227
O	2.00669	-5.49598	-0.40619
O	2.85035	-1.38192	3.64287
C	-2.62832	3.27799	-0.03325
O	-3.7844	2.67318	0.36644
C	-3.62794	1.34962	0.03665
N	-2.47721	1.10472	-0.53545
C	-4.74216	0.48781	0.37191
C	-4.83714	-0.84888	0.20598
C	-3.74782	-1.68912	-0.43761
C	-6.08839	-1.57411	0.62341
C	-3.91891	-1.7106	-1.96736
O	-3.83519	-2.99773	0.12877
C	-2.69097	-3.80187	-0.07783
H	0.80782	-4.13306	1.53139
H	4.05267	-3.43011	-0.54519
H	3.21108	-3.89029	-2.03335
H	1.60708	-2.01607	-1.73202

H	0.22932	-0.71704	1.61681
H	0.41644	-2.02931	2.78877
H	2.69806	-0.12224	2.10438
H	2.21579	1.01789	4.2745
H	0.68949	0.15211	4.40015
H	0.17525	2.36406	3.58859
H	-0.11576	1.23836	2.27988
H	2.34143	1.88313	1.50139
H	3.02948	4.19254	2.09939
H	2.88078	3.24142	3.58646
H	1.60013	4.3847	3.12179
H	-0.27961	3.52524	-1.43757
H	-1.02353	1.60793	-2.99602
H	-0.20846	0.50816	-1.91093
H	4.45278	-1.02354	-1.21592
H	3.82645	-1.41556	-2.81419
H	2.42	0.63749	-1.05791
H	2.69108	0.67448	-4.08963
H	1.47658	-0.33722	-3.31905
H	2.26967	3.07402	-4.28789
H	0.6896	3.76743	-3.63371
H	4.76973	1.38796	-1.11127
H	4.82213	1.21442	-2.87401
H	3.78674	2.4492	-2.13509
H	2.29994	-1.76143	4.34741
H	-2.53003	4.33457	0.15495
H	-5.58211	1.02111	0.80944
H	-2.7668	-1.25419	-0.20563
H	-6.84116	-0.88141	1.01063
H	-6.52233	-2.12712	-0.21955
H	-5.85834	-2.32152	1.38982
H	-3.15217	-2.33553	-2.43722
H	-3.81925	-0.69842	-2.36909
H	-4.90143	-2.10971	-2.24126
H	-2.83627	-4.71252	0.50949
H	-2.55652	-4.0864	-1.13138
H	-1.77169	-3.30009	0.26342

Compound 1b: Isomer 2S, 5R, 11S, 13S, 17S, 23S conformer 46

C	-1.23075	2.74529	1.43531
C	-0.00734	3.33445	1.47046
C	0.53849	4.02608	0.30654
C	-0.4065	4.05998	-0.8981
C	-1.20128	2.76264	-0.95634
O	-1.92917	2.56575	0.29274
C	-2.00013	2.31044	2.65116
C	-2.69815	0.93517	2.58197
C	-1.71833	-0.24052	2.56787
C	-2.41385	-1.61239	2.59758
C	-3.13205	-2.04159	1.28388
C	-2.11966	-2.66868	0.3383
C	-4.26612	-3.03416	1.56859
O	-1.41788	-1.7373	-0.34972
O	-1.92328	-3.86373	0.2331
C	-0.35798	-2.24563	-1.22784
C	-0.11491	-1.16634	-2.27838
C	0.87424	-2.55678	-0.42346
C	-1.26357	-0.88348	-3.23934
C	-2.24157	2.63878	-2.06839
C	-2.57663	1.16245	-2.38756
C	-1.58422	0.58308	-3.44261
C	-1.91317	-1.85071	-3.89489
C	-4.02707	0.9854	-2.85275
O	1.62839	4.58547	0.29544
O	-3.51776	0.77467	3.74408
C	1.13418	-3.65597	0.33615
O	2.37157	-3.50569	0.88916
C	2.82727	-2.29593	0.42718
N	1.96625	-1.6992	-0.35642
C	4.15916	-1.92993	0.86268
C	4.8667	-0.82443	0.5448
C	4.36681	0.26803	-0.39657
C	6.26873	-0.65647	1.0699
C	4.6763	-0.08791	-1.85515
O	5.00966	1.51148	-0.1308
C	4.42383	2.2515	0.92758
H	0.53418	3.40479	2.40704

H	-1.08165	4.92248	-0.7994
H	0.17978	4.19798	-1.81137
H	-0.48379	1.93022	-1.01605
H	-1.34594	2.34382	3.52821
H	-2.78369	3.06703	2.81643
H	-3.32094	0.90803	1.67651
H	-1.07784	-0.15425	3.45572
H	-1.06684	-0.1558	1.6909
H	-3.15859	-1.59674	3.39929
H	-1.68425	-2.38694	2.86589
H	-3.54135	-1.15087	0.79284
H	-4.75954	-3.35507	0.6453
H	-5.01538	-2.56935	2.21795
H	-3.88061	-3.92943	2.06543
H	-0.73756	-3.1659	-1.67526
H	0.773	-1.45942	-2.85358
H	0.17628	-0.25234	-1.75163
H	-3.13683	3.18411	-1.74526
H	-1.87427	3.14618	-2.96992
H	-2.45716	0.5917	-1.45776
H	-2.00374	0.74814	-4.4433
H	-0.64371	1.15031	-3.40892
H	-2.6933	-1.6223	-4.61658
H	-1.69259	-2.90555	-3.74847
H	-4.73351	1.30107	-2.07595
H	-4.2298	1.58072	-3.75275
H	-4.2347	-0.06388	-3.09035
H	-4.23367	1.42706	3.69881
H	0.5845	-4.55034	0.57775
H	4.61722	-2.67829	1.50473
H	3.28006	0.37594	-0.2887
H	6.59092	-1.53569	1.6356
H	6.97469	-0.49066	0.24669
H	6.35021	0.22429	1.71668
H	5.75268	-0.22855	-2.00151
H	4.34719	0.72926	-2.50467
H	4.15399	-1.0047	-2.14169
H	3.40263	2.57467	0.68335
H	4.40104	1.68181	1.87008

H 5.04247 3.14153 1.06949

Compound 1b: Isomer 2S, 5R, 11S, 13S, 17S, 23S conformer 47

C	3.07992	-1.86512	-0.18172
C	4.17267	-2.63845	-0.4096
C	5.50231	-2.06141	-0.57467
C	5.53906	-0.53873	-0.44995
C	4.23656	0.08769	-0.94403
O	3.09738	-0.51127	-0.25563
C	1.73767	-2.42015	0.21158
C	1.56278	-2.62349	1.73262
C	1.53049	-1.32856	2.56388
C	0.30782	-0.44422	2.28583
C	0.38372	0.95708	2.93601
C	-0.61142	1.91933	2.28502
C	0.20509	0.94799	4.45738
O	-0.53442	1.83249	0.93591
O	-1.34766	2.68442	2.86958
C	-1.41306	2.66695	0.13312
C	-0.81007	2.70223	-1.28278
C	-2.79356	2.08325	0.14391
C	0.62999	3.18512	-1.37018
C	4.19446	1.60251	-0.73683
C	3.13935	2.44206	-1.49992
C	1.70085	2.1572	-1.02951
C	0.88237	4.45082	-1.72324
C	3.28729	2.31589	-3.02466
O	6.51841	-2.72371	-0.73862
O	0.3499	-3.37222	1.86489
C	-3.9238	2.54983	0.73505
O	-4.92865	1.65761	0.49524
C	-4.34493	0.65436	-0.23869
N	-3.0796	0.8759	-0.48043
C	-5.21257	-0.44703	-0.60257
C	-4.85401	-1.62905	-1.14865
C	-3.41795	-1.99896	-1.50548
C	-5.90577	-2.65672	-1.47647
C	-3.10377	-1.65756	-2.96516

O	-3.19311	-3.39949	-1.34674
C	-2.95821	-3.78838	-0.00391
H	4.08422	-3.71914	-0.38439
H	5.70295	-0.28011	0.60684
H	6.39212	-0.15505	-1.01726
H	4.10899	-0.16756	-2.00552
H	0.94281	-1.77542	-0.17429
H	1.61586	-3.40535	-0.24927
H	2.41253	-3.23304	2.07956
H	2.45154	-0.76665	2.35951
H	1.57159	-1.60415	3.62788
H	-0.60394	-0.95216	2.62389
H	0.20272	-0.31257	1.20791
H	1.37549	1.37359	2.69693
H	0.25338	1.9611	4.86505
H	0.98406	0.34563	4.93604
H	-0.77012	0.5297	4.73031
H	-1.42868	3.66607	0.57735
H	-1.45714	3.35629	-1.87871
H	-0.90169	1.69473	-1.70485
H	4.10194	1.7885	0.3419
H	5.18525	1.97711	-1.02767
H	3.37989	3.48014	-1.23098
H	1.38066	1.18224	-1.42034
H	1.70096	2.02358	0.05845
H	1.88812	4.852	-1.80459
H	0.07585	5.14476	-1.94839
H	4.30879	2.54971	-3.34879
H	3.04993	1.30221	-3.3699
H	2.60586	3.00053	-3.53838
H	0.19668	-3.52245	2.81111
H	-4.16517	3.41404	1.33248
H	-6.26215	-0.2617	-0.38728
H	-2.7274	-1.43757	-0.86445
H	-6.91189	-2.27917	-1.27178
H	-5.85202	-2.9469	-2.53342
H	-5.75276	-3.57763	-0.90325
H	-2.08089	-1.9699	-3.19796
H	-3.19268	-0.57976	-3.12787

H	-3.78348	-2.17938	-3.64731
H	-2.82862	-4.87425	-0.00913
H	-2.04675	-3.32565	0.40262
H	-3.80398	-3.53719	0.65565

Compound 1b: Isomer 2S, 5R, 11S, 13S, 17S, 23S conformer 52

C	0.07107	-2.71796	1.17567
C	-1.03572	-3.18034	0.53624
C	-0.93489	-3.87721	-0.74472
C	0.50062	-4.05154	-1.2496
C	1.30767	-2.82171	-0.86123
O	1.28708	-2.65777	0.58704
C	0.11951	-2.32864	2.62795
C	0.94334	-1.07635	3.00075
C	0.28525	0.23631	2.57034
C	1.07809	1.48564	2.99555
C	2.36977	1.78793	2.18091
C	1.99633	2.50037	0.89035
C	3.34854	2.64855	2.98934
O	1.5996	1.62687	-0.06996
O	2.00568	3.70383	0.73296
C	1.08521	2.21435	-1.30425
C	1.22879	1.1456	-2.38716
C	-0.33566	2.64949	-1.10687
C	2.65595	0.75253	-2.75598
C	2.7783	-2.78603	-1.27268
C	3.30204	-1.33652	-1.39173
C	2.93704	-0.73509	-2.7838
C	3.58718	1.65689	-3.07602
C	4.81081	-1.24138	-1.13748
O	-1.89612	-4.33415	-1.35128
O	1.07198	-1.01768	4.42586
C	-0.81434	3.91312	-0.96581
O	-2.16845	3.84185	-0.8282
C	-2.46272	2.50026	-0.88001
N	-1.40076	1.75675	-1.0523
C	-3.87309	2.18354	-0.75736
C	-4.45686	0.98175	-0.56496

C	-3.69403	-0.32946	-0.42118
C	-5.95993	0.88594	-0.49177
C	-3.77642	-1.18036	-1.69054
O	-4.24287	-1.1177	0.6428
C	-3.9809	-0.59303	1.92975
H	-2.00665	-3.1363	1.01678
H	0.93634	-4.95757	-0.80412
H	0.48588	-4.18508	-2.33528
H	0.78567	-1.94155	-1.26607
H	-0.90021	-2.22819	3.01354
H	0.56841	-3.18108	3.16264
H	1.93644	-1.16336	2.53765
H	-0.70546	0.27748	3.04258
H	0.12514	0.22629	1.48664
H	1.3625	1.36442	4.04555
H	0.42555	2.36684	2.94996
H	2.85155	0.83986	1.91331
H	4.24976	2.87978	2.41199
H	3.64766	2.12037	3.90079
H	2.88532	3.59847	3.27255
H	1.69515	3.09201	-1.52667
H	0.71472	1.51741	-3.28364
H	0.65723	0.27022	-2.06298
H	3.34967	-3.35117	-0.52615
H	2.90497	-3.31084	-2.22874
H	2.79711	-0.74228	-0.61911
H	3.75368	-0.95545	-3.48328
H	2.0494	-1.24664	-3.18152
H	4.58676	1.35894	-3.38201
H	3.39423	2.72707	-3.05322
H	5.0629	-1.57148	-0.12281
H	5.37164	-1.86813	-1.8433
H	5.16063	-0.20956	-1.25303
H	1.62096	-1.76526	4.70862
H	-0.36329	4.89144	-0.92762
H	-4.50806	3.06332	-0.83366
H	-2.63937	-0.11778	-0.20727
H	-6.43121	1.86848	-0.5866
H	-6.34852	0.24228	-1.29134

H	-6.28123	0.42762	0.44945
H	-4.81696	-1.38629	-1.96203
H	-3.27403	-2.13839	-1.52927
H	-3.29363	-0.6573	-2.52224
H	-2.89958	-0.49318	2.11243
H	-4.44423	0.39327	2.0858
H	-4.40242	-1.298	2.65188

Compound 1b: Isomer 2S, 5R, 11S, 13S, 17S, 23S conformer 55

C	-2.51548	2.15526	0.63899
C	-3.06862	3.30755	0.1809
C	-4.05809	3.31611	-0.89132
C	-4.466	1.9259	-1.37748
C	-3.31315	0.93083	-1.27082
O	-2.73679	0.9464	0.06832
C	-1.58168	2.09422	1.81658
C	-2.06543	1.29907	3.06178
C	-2.19143	-0.22462	2.87936
C	-0.88777	-0.92836	2.47408
C	-1.06312	-2.44019	2.17612
C	0.04757	-2.94273	1.25453
C	-1.15675	-3.30646	3.43505
O	0.11101	-2.18414	0.13221
O	0.77099	-3.89292	1.46431
C	1.15693	-2.48344	-0.83908
C	0.73429	-1.81511	-2.15468
C	2.47599	-1.97127	-0.33933
C	-0.51301	-2.38498	-2.82279
C	-3.73688	-0.50714	-1.55928
C	-2.61472	-1.56754	-1.53379
C	-1.77324	-1.5378	-2.83711
C	-0.46778	-3.57502	-3.43313
C	-3.21687	-2.96065	-1.30224
O	-4.57205	4.33406	-1.33562
O	-1.22587	1.62641	4.16974
C	3.45799	-2.64889	0.31158
O	4.4707	-1.77558	0.58044
C	4.04762	-0.5715	0.07448

N	2.86799	-0.64568	-0.48616
C	4.96743	0.53374	0.24457
C	4.76734	1.83327	-0.0618
C	3.49109	2.35049	-0.70173
C	5.85333	2.84496	0.18938
C	3.5713	2.24796	-2.23473
O	3.32071	3.70292	-0.26978
C	2.00437	4.19556	-0.40308
H	-2.8221	4.25192	0.65279
H	-5.31214	1.58392	-0.76337
H	-4.81906	1.99566	-2.4107
H	-2.504	1.24892	-1.94487
H	-0.61942	1.68546	1.47781
H	-1.39713	3.11621	2.15848
H	-3.04868	1.69068	3.34564
H	-2.95288	-0.42057	2.11647
H	-2.56606	-0.62885	3.82764
H	-0.12969	-0.81871	3.26365
H	-0.48837	-0.45175	1.57594
H	-1.98994	-2.54848	1.59357
H	-1.31667	-4.3579	3.18017
H	-1.98522	-2.97591	4.06966
H	-0.23103	-3.24612	4.01676
H	1.20429	-3.5698	-0.94702
H	1.58484	-1.91348	-2.83979
H	0.62209	-0.74353	-1.96312
H	-4.50153	-0.77151	-0.81653
H	-4.24069	-0.5196	-2.53636
H	-1.95283	-1.33751	-0.69446
H	-2.4158	-1.86604	-3.66557
H	-1.48951	-0.49867	-3.05845
H	-1.33422	-3.9928	-3.93946
H	0.43806	-4.17796	-3.45482
H	-3.75263	-3.00476	-0.34621
H	-3.93397	-3.21749	-2.09377
H	-2.44134	-3.73237	-1.29369
H	-0.33522	1.29351	3.97501
H	3.57485	-3.66416	0.65352
H	5.9192	0.23945	0.67915

H	2.64149	1.74588	-0.35787
H	6.75546	2.37157	0.58748
H	6.11663	3.37716	-0.73383
H	5.50812	3.60972	0.89279
H	2.66804	2.65674	-2.70009
H	3.65939	1.20018	-2.53491
H	4.43427	2.80435	-2.61564
H	1.99475	5.1941	0.04222
H	1.6842	4.27868	-1.45212
H	1.27761	3.56103	0.13002

Compound 1b: Isomer 2S, 5R, 11S, 13S, 17S, 23S conformer 56

C	2.21206	2.32299	-1.56363
C	1.9058	3.53712	-1.03635
C	2.65446	4.10055	0.08456
C	3.80011	3.21326	0.58143
C	3.42619	1.7465	0.41676
O	3.08838	1.47215	-0.97442
C	1.68391	1.81321	-2.87765
C	0.72596	0.5882	-2.85985
C	1.38297	-0.69841	-2.34535
C	0.53153	-1.9622	-2.53688
C	1.05507	-3.20449	-1.75128
C	0.53395	-3.17756	-0.31842
C	0.66161	-4.52248	-2.42647
O	1.0121	-2.09679	0.36128
O	-0.23802	-3.97189	0.16976
C	0.3434	-1.72433	1.59515
C	1.09807	-0.50298	2.1515
C	-1.07858	-1.34667	1.29177
C	2.47376	-0.70821	2.7639
C	4.50553	0.7444	0.81831
C	4.08823	-0.74311	0.67816
C	3.58453	-1.39588	1.99358
C	2.69733	-0.26017	4.00681
C	5.25554	-1.58042	0.12966
O	2.44201	5.20965	0.55737
O	-0.47719	0.89963	-2.17094

C	-2.23676	-1.76931	1.85533
O	-3.27095	-1.10352	1.25039
C	-2.68402	-0.29119	0.32205
N	-1.37704	-0.40399	0.31724
C	-3.45891	0.54798	-0.56837
C	-4.77146	0.86365	-0.5268
C	-5.75134	0.37382	0.536
C	-5.3571	1.73562	-1.60827
C	-6.53147	-0.85602	0.06269
O	-6.7154	1.37814	0.85013
C	-6.21017	2.40737	1.68047
H	1.16399	4.16325	-1.51848
H	4.7041	3.4452	-0.0006
H	4.00902	3.44869	1.62931
H	2.50972	1.56914	0.99239
H	1.15211	2.6361	-3.36405
H	2.54788	1.56309	-3.51026
H	0.43631	0.43882	-3.91075
H	1.60547	-0.56664	-1.28589
H	2.3513	-0.83224	-2.84667
H	0.50905	-2.22641	-3.60234
H	-0.50675	-1.75632	-2.25175
H	2.15111	-3.13551	-1.70144
H	1.01163	-5.38588	-1.85283
H	1.0952	-4.57326	-3.43085
H	-0.42631	-4.60548	-2.51435
H	0.36649	-2.57142	2.28715
H	0.4512	-0.05128	2.9112
H	1.13805	0.21741	1.32625
H	5.38523	0.94162	0.19212
H	4.80148	0.96591	1.85205
H	3.27985	-0.79648	-0.05498
H	3.26277	-2.41479	1.73816
H	4.44192	-1.5054	2.67121
H	3.66898	-0.36125	4.48404
H	1.92057	0.22307	4.59493
H	5.57451	-1.21284	-0.85231
H	6.12385	-1.53817	0.80039
H	4.97366	-2.63452	0.01953

H	-0.45543	0.54221	-1.26011
H	-2.49879	-2.49242	2.61055
H	-2.84425	0.94762	-1.37145
H	-5.20473	0.11043	1.44978
H	-5.77147	2.66044	-1.19289
H	-4.6071	1.99299	-2.36077
H	-6.1913	1.22885	-2.11011
H	-7.09695	-0.63503	-0.84822
H	-7.2405	-1.15808	0.8397
H	-5.84864	-1.68659	-0.13874
H	-5.38901	2.96512	1.20535
H	-7.03843	3.09393	1.87426
H	-5.84504	2.00749	2.63959

Compound 1b: Isomer 2S, 5R, 11S, 13S, 17S, 23S conformer 57

C	-2.23629	2.4214	0.49289
C	-2.91479	3.53541	0.11615
C	-4.20037	3.461	-0.5686
C	-4.75034	2.04284	-0.72811
C	-3.63488	1.00948	-0.87028
O	-2.64389	1.16945	0.18375
C	-0.93325	2.46128	1.25362
C	-0.73692	1.45688	2.41946
C	-0.47752	0.03636	1.92725
C	-0.20423	-0.99657	3.03116
C	-0.49519	-2.46883	2.62425
C	0.35248	-2.95034	1.44798
C	-0.32287	-3.41924	3.81434
O	0.01419	-2.30953	0.29975
O	1.21003	-3.80613	1.50338
C	0.78751	-2.61195	-0.89789
C	0.02091	-1.99246	-2.07657
C	2.18163	-2.06493	-0.7967
C	-1.3235	-2.62801	-2.41824
C	-4.14449	-0.42907	-0.78844
C	-3.08913	-1.55319	-0.87506
C	-2.5574	-1.75038	-2.31823
C	-1.39601	-3.89319	-2.84664

C	-3.67876	-2.86073	-0.32674
O	-4.8333	4.44192	-0.93677
O	0.40708	1.84805	3.18686
C	3.34132	-2.74328	-0.59551
O	4.37044	-1.85028	-0.63477
C	3.77659	-0.6319	-0.85126
N	2.47549	-0.71592	-0.96153
C	4.68551	0.4923	-0.95796
C	4.38097	1.80684	-0.93347
C	2.96613	2.34177	-0.74868
C	5.46489	2.83691	-1.12143
C	2.30492	2.66394	-2.0911
O	2.96807	3.54819	0.01542
C	3.19949	3.34336	1.40077
H	-2.52545	4.51758	0.35981
H	-5.35651	1.81467	0.16098
H	-5.41774	2.01263	-1.59456
H	-3.09671	1.18833	-1.81265
H	-0.10696	2.30681	0.54508
H	-0.81689	3.4789	1.64401
H	-1.63209	1.46645	3.06353
H	0.35752	0.06387	1.21895
H	-1.35402	-0.26707	1.35554
H	-0.84634	-0.78496	3.89718
H	0.82704	-0.9092	3.39189
H	-1.53826	-2.50531	2.27884
H	-0.57039	-4.44946	3.54149
H	-0.97527	-3.11289	4.63916
H	0.71189	-3.41348	4.17049
H	0.83925	-3.70004	-0.99024
H	0.68005	-2.06277	-2.95075
H	-0.09225	-0.92372	-1.87074
H	-4.68372	-0.52465	0.16362
H	-4.89518	-0.56195	-1.58085
H	-2.24214	-1.27662	-0.24077
H	-3.36195	-2.17814	-2.93161
H	-2.32071	-0.76926	-2.75488
H	-2.34253	-4.35019	-3.12373
H	-0.51351	-4.52297	-2.94228

H	-3.97728	-2.74882	0.72263
H	-4.56989	-3.16125	-0.89436
H	-2.95234	-3.67646	-0.38804
H	0.22395	2.71268	3.58708
H	3.58866	-3.77404	-0.40086
H	5.72486	0.1949	-1.07627
H	2.35994	1.58324	-0.23726
H	6.43649	2.36707	-1.29982
H	5.23583	3.48944	-1.97377
H	5.5468	3.49532	-0.25025
H	2.8884	3.4014	-2.65222
H	1.30819	3.08223	-1.91669
H	2.20838	1.7537	-2.68947
H	2.41844	2.71829	1.85639
H	4.17747	2.87717	1.59533
H	3.18686	4.33258	1.86803

Compound 1b: Isomer 2S, 5R, 11S, 13S, 17S, 23S conformer 63

C	-0.40192	2.3504	1.26978
C	0.12411	3.58867	1.44432
C	-0.42466	4.75602	0.76161
C	-1.68872	4.47536	-0.05689
C	-1.68085	3.06612	-0.64356
O	-1.38653	2.08406	0.38268
C	0.05745	1.09647	1.9669
C	-1.09706	0.19943	2.43315
C	-0.63022	-1.22707	2.76062
C	-1.77331	-2.23994	2.95186
C	-2.71005	-2.47221	1.73108
C	-1.90611	-2.92676	0.51804
C	-3.7935	-3.50593	2.06467
O	-1.45432	-1.87255	-0.20136
O	-1.67155	-4.08445	0.23566
C	-0.56443	-2.16524	-1.33134
C	-0.54768	-0.90283	-2.19502
C	0.80628	-2.53389	-0.83499
C	-1.80349	-0.59879	-3.00383
C	-3.01188	2.65018	-1.27315

C	-3.10856	1.17053	-1.70314
C	-2.25797	0.84702	-2.96426
C	-2.43374	-1.52261	-3.73649
C	-4.57355	0.7711	-1.92861
O	0.03268	5.88655	0.86844
O	-1.68256	0.84504	3.57028
C	1.27587	-3.75863	-0.4748
O	2.57781	-3.61964	-0.09474
C	2.85574	-2.28369	-0.24285
N	1.82813	-1.60158	-0.68134
C	4.20932	-1.89589	0.0961
C	4.77819	-0.67445	0.01081
C	4.05922	0.54748	-0.53439
C	6.21525	-0.48351	0.41558
C	4.17424	0.59668	-2.06911
O	4.64802	1.69194	0.08277
C	3.88768	2.87878	-0.03233
H	0.93098	3.74078	2.15236
H	-2.55438	4.59622	0.61065
H	-1.77745	5.22501	-0.84895
H	-0.86079	2.99026	-1.37346
H	0.66661	0.52486	1.25297
H	0.69391	1.35682	2.81866
H	-1.82481	0.15735	1.61656
H	-0.03298	-1.18997	3.68079
H	0.03446	-1.57673	1.96344
H	-2.40708	-1.92761	3.7943
H	-1.34584	-3.20804	3.24226
H	-3.18559	-1.52177	1.46442
H	-4.49377	-3.63166	1.23229
H	-4.36364	-3.18954	2.94498
H	-3.34413	-4.48139	2.27192
H	-0.9944	-3.01776	-1.86047
H	0.30444	-0.98358	-2.88237
H	-0.31088	-0.0622	-1.53569
H	-3.80278	2.85548	-0.53917
H	-3.20448	3.31001	-2.13104
H	-2.72384	0.56587	-0.87496
H	-2.84214	1.08855	-3.86151

H	-1.36884	1.49216	-2.99128
H	-3.29586	-1.27038	-4.34849
H	-2.1162	-2.56242	-3.76659
H	-5.16328	0.89092	-1.01202
H	-5.03776	1.39001	-2.70793
H	-4.64777	-0.27571	-2.24196
H	-2.54591	0.43722	3.73476
H	0.84279	-4.74323	-0.41676
H	4.81285	-2.72692	0.45175
H	2.9951	0.49463	-0.26899
H	6.67162	-1.42801	0.72557
H	6.80389	-0.06343	-0.40998
H	6.28758	0.23759	1.23635
H	5.22504	0.62102	-2.37713
H	3.679	1.48769	-2.46899
H	3.6937	-0.2812	-2.51019
H	3.854	3.26216	-1.06257
H	2.85385	2.73697	0.31869
H	4.37543	3.62657	0.59855

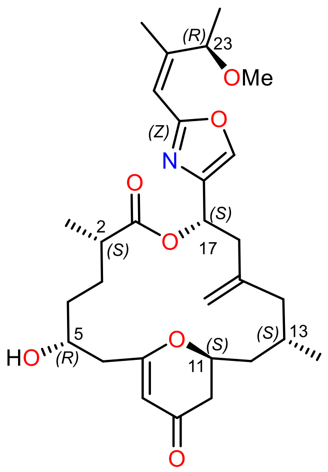

Table 5. Calculated DFT energies of the **1c**-(2S5R11S13S17S23R*)* diasteroisomer.

**Conformers 1c**	OPLS2008 Force Field	B3LYP/6-31+G(d,p)	Δ (DFT Energy)	% Population
**1c27**	3.033771511	-1710.457316	0	31.66
**1c3**	0.665033461	-1710.456605	0.44615918	14.90
**1c10**	1.587858509	-1710.456251	0.6682975	10.24
**1c1**	0	-1710.456055	0.79128934	8.31
**1c49**	4.037237094	-1710.455728	0.99648491	5.88
**1c38**	3.522538241	-1710.455608	1.07178604	5.18
**1c23**	2.803417782	-1710.455529	1.12135928	4.76
**1c7**	1.343140535	-1710.455497	1.14143958	4.60
**1c30**	3.134942639	-1710.454966	1.47464707	2.62
**1c16**	2.067710325	-1710.454909	1.5104151	2.47
**1c22**	2.673087954	-1710.454587	1.71247313	1.75
**1c51**	4.068618547	-1710.454511	1.76016384	1.62
**1c52**	4.156716061	-1710.454278	1.90637353	1.26
**1c13**	1.850860421	-1710.454269	1.91202111	1.25
**1c42**	3.694694073	-1710.453716	2.25903381	0.70
**1c46**	3.740248566	-1710.453422	2.44352157	0.51
**1c57**	4.662165392	-1710.453352	2.48744723	0.47
**1c11**	1.756835564	-1710.453154	2.61169409	0.38
**1c55**	4.470984704	-1710.452868	2.79116177	0.28
**1c29**	3.091037285	-1710.452806	2.83006735	0.27
**1c31**	3.143283939	-1710.452093	3.27748155	0.12
**1c58**	4.684369025	-1710.452083	3.28375664	0.12
**1c19**	2.379732314	-1710.452035	3.31387709	0.12
**1c12**	1.842997132	-1710.451995	3.33897747	0.11

24 conformers counting for the 99.58% of the DFT conformational population

DFT COORDINATES FOR CONFORMATIONAL SEARCH OF 1c
(2S*, 5R*, 11S*, 13S* , 17S*, 23R*)

Compound 1c: Isomer 2S, 5R, 11S, 13S, 17S, 23R conformer 1

C	-2.78941	1.3837	1.26069
C	-3.719	2.19976	1.82011
C	-4.92354	2.59881	1.09399
C	-5.07342	1.95431	-0.28648
C	-3.71604	1.6918	-0.93558
O	-2.85075	0.96248	-0.02468
C	-1.60595	0.78632	1.96638
C	-1.7275	-0.75212	2.07559
C	-0.41494	-1.40686	2.503
C	-0.47684	-2.94303	2.5511
C	-0.80364	-3.67487	1.21944
C	0.3051	-3.48493	0.19306
C	-1.03276	-5.17153	1.46749
O	0.20625	-2.28942	-0.44625
O	1.18577	-4.28867	-0.03541
C	1.24332	-1.99936	-1.43554
C	0.70312	-0.91046	-2.37544
C	2.51377	-1.58387	-0.75301
C	-0.61251	-1.26401	-3.04904
C	-3.80923	0.90525	-2.24382
C	-2.48369	0.51452	-2.95062
C	-1.89013	-0.80057	-2.37073
C	-0.63374	-1.95145	-4.19649
C	-1.48023	1.67678	-2.9921
O	-5.78341	3.33394	1.55945
O	-2.72085	-1.1189	3.03247
C	3.60545	-2.34131	-0.46571
O	4.51967	-1.54968	0.16385
C	3.92989	-0.31256	0.23805
N	2.73181	-0.29189	-0.28702
C	4.72791	0.7156	0.87598
C	4.48196	2.04149	0.93867
C	3.25451	2.70446	0.32549
C	5.43147	2.94946	1.67695
C	2.14842	2.9059	1.36457

O	3.57224	3.99599	-0.19621
C	4.2466	3.95191	-1.43914
H	-3.60167	2.5365	2.84411
H	-5.61885	1.00699	-0.16222
H	-5.6877	2.6032	-0.91782
H	-3.21623	2.65516	-1.11092
H	-0.70291	1.03177	1.3933
H	-1.50935	1.21272	2.96972
H	-2.00549	-1.12933	1.08231
H	-0.15639	-1.03971	3.50526
H	0.37481	-1.07389	1.82128
H	-1.2466	-3.23458	3.2738
H	0.47815	-3.32862	2.932
H	-1.7158	-3.23782	0.79792
H	-1.28555	-5.69551	0.54002
H	-1.85449	-5.3125	2.17734
H	-0.13291	-5.64047	1.87661
H	1.41985	-2.92373	-1.99025
H	1.48302	-0.74406	-3.12844
H	0.61291	0.01919	-1.80599
H	-4.40698	0.00072	-2.06722
H	-4.39753	1.53422	-2.92602
H	-2.75521	0.2899	-3.99214
H	-1.70844	-0.67467	-1.30209
H	-2.65713	-1.5796	-2.47254
H	-1.56777	-2.23711	-4.67547
H	0.27912	-2.25944	-4.70213
H	-1.94536	2.58386	-3.39798
H	-1.0933	1.91536	-1.99486
H	-0.62747	1.43125	-3.63297
H	-3.55328	-0.688	2.77954
H	3.85666	-3.38072	-0.59853
H	5.6266	0.32623	1.34847
H	2.86319	2.06965	-0.47882
H	6.24956	2.38565	2.13429
H	5.8566	3.70742	1.01004
H	4.909	3.50211	2.46834
H	1.29705	3.4103	0.89652
H	1.81988	1.93897	1.75691

H	2.49631	3.52669	2.19678
H	5.22419	3.45023	-1.36918
H	3.64833	3.43243	-2.20389
H	4.40339	4.98754	-1.75273

Compound 1c: Isomer 2S, 5R, 11S, 13S, 17S, 23R conformer 3

C	1.8576	2.12526	-1.24422
C	2.90328	2.88424	-1.66826
C	3.59038	3.80602	-0.76921
C	2.94392	3.93462	0.61219
C	2.30164	2.62653	1.08077
O	1.44006	2.07371	0.0408
C	1.07889	1.17618	-2.10896
C	1.71141	-0.23828	-2.0751
C	0.78088	-1.30434	-2.65111
C	1.37052	-2.7258	-2.62988
C	1.85565	-3.26772	-1.25696
C	0.71017	-3.34601	-0.25653
C	2.50182	-4.64988	-1.42146
O	0.51724	-2.16302	0.37962
O	0.03017	-4.33096	-0.05011
C	-0.59404	-2.09556	1.33008
C	-0.31945	-0.88742	2.22919
C	-1.9043	-1.98204	0.60236
C	0.89036	-1.00056	3.15208
C	3.3273	1.57438	1.53138
C	2.75788	0.18017	1.86454
C	1.85037	0.17233	3.12402
C	1.07066	-2.04414	3.968
C	3.89525	-0.84009	2.01191
O	4.55932	4.47958	-1.093
O	2.92792	-0.27351	-2.81805
C	-2.72234	-2.9795	0.1704
O	-3.80416	-2.41092	-0.43409
C	-3.59591	-1.05758	-0.34109
N	-2.47117	-0.75999	0.25674
C	-4.6389	-0.22746	-0.9114
C	-4.80421	1.10593	-0.77925

C	-3.86897	1.99766	0.02869
C	-5.94957	1.7965	-1.4736
C	-2.85043	2.70649	-0.8671
O	-4.59663	3.01869	0.71462
C	-5.27523	2.55173	1.86493
H	3.19423	2.86922	-2.71333
H	3.69388	4.27869	1.33048
H	2.17727	4.71863	0.53915
H	1.60003	2.83055	1.89375
H	0.05291	1.12496	-1.7284
H	1.05679	1.5289	-3.14496
H	1.90627	-0.48574	-1.0247
H	0.54662	-1.04408	-3.6921
H	-0.16072	-1.26783	-2.09166
H	2.23465	-2.75321	-3.30215
H	0.62728	-3.4247	-3.03567
H	2.59806	-2.56882	-0.85702
H	2.89292	-5.02317	-0.4692
H	3.33116	-4.59427	-2.13431
H	1.77228	-5.37772	-1.78868
H	-0.58387	-3.02863	1.89717
H	-1.22019	-0.72164	2.83464
H	-0.2306	-0.00735	1.58495
H	4.08852	1.46377	0.74747
H	3.85194	1.98694	2.40541
H	2.14129	-0.13064	1.01572
H	2.48153	0.16678	4.02228
H	1.26	1.09857	3.16293
H	1.91748	-2.09496	4.64757
H	0.3825	-2.88607	3.99845
H	4.47851	-0.92242	1.08683
H	4.58546	-0.55	2.81515
H	3.49879	-1.83209	2.25123
H	3.53174	0.37717	-2.42299
H	-2.66874	-4.05541	0.18512
H	-5.35656	-0.78925	-1.50472
H	-3.32317	1.38449	0.75588
H	-6.55022	1.09091	-2.05482
H	-6.60056	2.3059	-0.75497

H	-5.58181	2.57584	-2.15373
H	-2.19247	3.32869	-0.25271
H	-2.24294	1.97058	-1.40187
H	-3.34971	3.35219	-1.59695
H	-6.04267	1.80024	1.62331
H	-4.57765	2.10802	2.59245
H	-5.76139	3.41841	2.321

Compound 1c: Isomer 2S, 5R, 11S, 13S, 17S, 23R conformer 7

C	-1.83637	-2.14743	-1.2671
C	-2.8184	-3.00451	-1.64015
C	-3.4011	-3.95222	-0.69881
C	-2.72637	-3.97239	0.67555
C	-2.18226	-2.60029	1.08028
O	-1.3803	-2.0298	0.00359
C	-1.12827	-1.18676	-2.1795
C	-1.70131	0.24758	-2.08485
C	-0.74207	1.29408	-2.6691
C	-1.30526	2.7263	-2.67519
C	-1.80679	3.29328	-1.3196
C	-0.67879	3.37784	-0.29984
C	-2.43699	4.67859	-1.51612
O	-0.50394	2.20049	0.35
O	-0.00185	4.36353	-0.08751
C	0.57021	2.14575	1.34249
C	0.25555	0.95326	2.2485
C	1.90729	2.01986	0.6693
C	-0.98734	1.08697	3.1238
C	-3.27653	-1.60828	1.50189
C	-2.78638	-0.18157	1.82257
C	-1.92489	-0.10373	3.11284
C	-1.21542	2.16174	3.88546
C	-3.97266	0.78884	1.90513
O	-4.30693	-4.72647	-0.98039
O	-3.00669	0.32258	-2.65742
C	2.76746	3.00911	0.30679
O	3.86582	2.43219	-0.25904
C	3.62214	1.08194	-0.21663

N	2.46267	0.79494	0.31648
C	4.67506	0.24366	-0.75541
C	4.77866	-1.10126	-0.69606
C	3.74083	-2.00135	-0.03608
C	5.95305	-1.79653	-1.33484
C	2.76555	-2.57856	-1.06501
O	4.36072	-3.11169	0.6154
C	4.95096	-2.77804	1.85714
H	-3.16479	-3.01961	-2.66709
H	-3.43691	-4.34462	1.41947
H	-1.90123	-4.69641	0.6216
H	-1.45556	-2.71623	1.88907
H	-0.06959	-1.16709	-1.8952
H	-1.19057	-1.53822	-3.21667
H	-1.85538	0.47928	-1.03035
H	-0.49006	1.01981	-3.7057
H	0.19619	1.24856	-2.10357
H	-2.16029	2.76375	-3.35982
H	-0.54514	3.40691	-3.08049
H	-2.56225	2.60668	-0.92377
H	-2.8343	5.07151	-0.57461
H	-3.26019	4.61871	-2.23581
H	-1.69674	5.39347	-1.88756
H	0.54192	3.08814	1.89307
H	1.13191	0.78974	2.88976
H	0.1842	0.065	1.61309
H	-4.02662	-1.5511	0.70276
H	-3.78957	-2.03663	2.37529
H	-2.15511	0.13784	0.98803
H	-2.5878	-0.07213	3.98725
H	-1.32078	-1.01646	3.21364
H	-2.087	2.2244	4.53188
H	-0.54569	3.01872	3.90296
H	-4.51838	0.82773	0.95512
H	-4.68108	0.48077	2.68561
H	-3.63157	1.80205	2.14251
H	-2.92343	0.15075	-3.61004
H	2.73744	4.0853	0.34951
H	5.45811	0.80889	-1.25534

H 3.16714 -1.41903 0.69481
H 6.61681 -1.08516 -1.83471
H 6.53254 -2.35754 -0.59359
H 5.61669 -2.53335 -2.07564
H 2.02164 -3.20028 -0.55758
H 2.25037 -1.76789 -1.58863
H 3.28977 -3.19865 -1.79975
H 5.76412 -2.04324 1.75148
H 4.20812 -2.36886 2.55955
H 5.36231 -3.70208 2.27238

Compound 1c: Isomer 2S, 5R, 11S, 13S, 17S, 23R conformer 10

C 2.21585 -2.08652 0.78186
C 2.52339 -3.39761 0.61015
C 3.4612 -3.83187 -0.42452
C 4.10263 -2.692 -1.22304
C 3.16289 -1.49566 -1.34518
O 2.66583 -1.10853 -0.03671
C 1.38259 -1.50445 1.886
C 2.22258 -0.60651 2.83021
C 1.37638 0.34183 3.68592
C 0.53943 1.371 2.90639
C 1.35381 2.38183 2.04595
C 0.43209 2.94441 0.97293
C 1.97854 3.50251 2.88161
O 0.25559 2.03288 -0.01952
O -0.11202 4.0278 0.99607
C -0.78026 2.3188 -1.00831
C -0.49288 1.43109 -2.22719
C -2.12546 2.04512 -0.40349
C 0.69432 1.84184 -3.09451
C 3.80405 -0.24527 -1.94224
C 2.87932 0.99113 -2.01479
C 1.86712 0.88401 -3.18621
C 0.6651 2.98337 -3.7918
C 3.71438 2.27398 -2.12844
O 3.76831 -5.00102 -0.61279
O 2.97383 -1.40476 3.7427

C	-3.01688	2.93017	0.11549
O	-4.09337	2.22956	0.57482
C	-3.80091	0.91413	0.31244
N	-2.63526	0.75984	-0.26068
C	-4.81104	-0.04075	0.72233
C	-4.85943	-1.363	0.45371
C	-3.80376	-2.10074	-0.36162
C	-5.98508	-2.20203	1.00122
C	-2.75966	-2.76391	0.54061
O	-4.39302	-3.1383	-1.14732
C	-5.04623	-2.66394	-2.30896
H	2.10411	-4.14826	1.27087
H	5.02579	-2.39127	-0.70572
H	4.38882	-3.06585	-2.21071
H	2.27784	-1.79807	-1.92464
H	0.59038	-0.91181	1.41427
H	0.91886	-2.29874	2.47901
H	2.90982	-0.01295	2.21048
H	2.0615	0.84624	4.37789
H	0.7041	-0.26243	4.30931
H	-0.06789	1.94181	3.62045
H	-0.1719	0.84511	2.26043
H	2.13878	1.83234	1.51446
H	2.59791	4.16104	2.26389
H	2.61062	3.08782	3.67369
H	1.19915	4.11671	3.3427
H	-0.70914	3.3777	-1.26658
H	-1.39979	1.44165	-2.84397
H	-0.38331	0.40237	-1.87036
H	4.68237	-0.00261	-1.32909
H	4.18278	-0.49563	-2.94336
H	2.31229	1.03754	-1.07961
H	2.40731	1.05342	-4.12747
H	1.4738	-0.14119	-3.23546
H	1.4846	3.27825	-4.44199
H	-0.18023	3.66735	-3.74591
H	4.36454	2.39947	-1.2545
H	4.35593	2.25139	-3.01978
H	3.0722	3.15738	-2.20079

H	3.51516	-2.0195	3.22099
H	-3.02608	3.99917	0.25291
H	-5.60435	0.40386	1.31843
H	-3.29002	-1.38885	-1.01913
H	-6.65804	-1.60939	1.62756
H	-6.56806	-2.66069	0.19523
H	-5.59536	-3.03308	1.60287
H	-2.04325	-3.31471	-0.0768
H	-2.22311	-2.00379	1.11598
H	-3.22784	-3.47072	1.23358
H	-5.89056	-1.99756	-2.07348
H	-4.35216	-2.11871	-2.96749
H	-5.42641	-3.54045	-2.84057

Compound 1c: Isomer 2S, 5R, 11S, 13S, 17S, 23R conformer 11

C	-1.39546	-1.94654	-2.15565
C	-0.1021	-2.36063	-2.16974
C	0.46368	-3.14762	-1.08162
C	-0.53462	-3.51026	0.01551
C	-1.58398	-2.41301	0.16913
O	-2.23279	-2.15262	-1.11167
C	-2.05137	-1.1779	-3.2639
C	-2.36502	0.29946	-2.90722
C	-1.10702	1.11432	-2.59011
C	-1.36179	2.61793	-2.38788
C	-2.21031	3.0256	-1.15251
C	-1.40139	2.90347	0.13293
C	-2.72009	4.46671	-1.29772
O	-1.48761	1.65798	0.66853
O	-0.75366	3.79877	0.63374
C	-0.79875	1.44449	1.93777
C	-1.46628	0.25518	2.66429
C	0.65636	1.19349	1.68127
C	-2.98451	0.22995	2.61718
C	-2.65338	-2.74599	1.20822
C	-3.90863	-1.84866	1.26775
C	-3.66397	-0.31494	1.36939
C	-3.69462	0.70179	3.64932

C	-4.8465	-2.35301	2.37408
O	1.63105	-3.52586	-1.05107
O	-3.0049	0.92707	-4.01941
C	1.72471	2.00075	1.90272
O	2.84843	1.34026	1.48505
C	2.39838	0.13874	0.99951
N	1.09979	0.00996	1.1076
C	3.29422	-0.8539	0.44429
C	4.62097	-0.79446	0.1978
C	5.51134	0.40846	0.49625
C	5.31297	-2.00561	-0.37674
C	6.23312	0.25819	1.83872
O	6.525	0.56415	-0.49808
C	6.04394	1.10819	-1.71222
H	0.53012	-2.13417	-3.02081
H	-1.01616	-4.46288	-0.25067
H	0.00552	-3.66232	0.95467
H	-1.06287	-1.48255	0.41835
H	-1.41858	-1.19617	-4.15654
H	-2.99911	-1.67506	-3.51662
H	-3.03628	0.30473	-2.03591
H	-0.40951	0.99958	-3.43108
H	-0.61683	0.67869	-1.71286
H	-1.87561	2.99823	-3.27645
H	-0.3982	3.14079	-2.33218
H	-3.06652	2.34665	-1.07055
H	-3.32391	4.76693	-0.43488
H	-3.33689	4.55653	-2.19794
H	-1.88253	5.1663	-1.37514
H	-0.91824	2.36044	2.52075
H	-1.12692	0.30983	3.70556
H	-1.04964	-0.66768	2.25664
H	-2.99306	-3.77669	1.03499
H	-2.15155	-2.7567	2.18587
H	-4.42598	-1.98074	0.30807
H	-3.09771	-0.00047	0.48974
H	-4.65124	0.15799	1.28731
H	-4.78167	0.72305	3.63557
H	-3.21664	1.08874	4.54678

H	-5.07605	-3.4172	2.24269
H	-4.39892	-2.22393	3.36614
H	-5.79552	-1.8038	2.36572
H	-3.85405	0.48249	-4.16715
H	1.85731	2.99647	2.29354
H	2.765	-1.76933	0.19426
H	4.90355	1.32049	0.52584
H	4.60151	-2.81303	-0.56766
H	5.83008	-1.76055	-1.31044
H	6.08679	-2.37591	0.3091
H	6.87996	1.1253	2.00529
H	5.50833	0.19269	2.65584
H	6.85721	-0.64114	1.84912
H	5.29648	0.46023	-2.19473
H	5.59091	2.10024	-1.55735
H	6.90536	1.21101	-2.37769

Compound 1c: Isomer 2S, 5R, 11S, 13S, 17S, 23R conformer 12

C	0.62154	-2.32543	1.88626
C	-0.47976	-2.8835	1.31209
C	-0.41786	-3.55285	0.02082
C	0.98805	-3.64719	-0.56272
C	1.81042	-2.41861	-0.1776
O	1.82543	-2.25308	1.27538
C	0.59078	-1.65976	3.23346
C	0.05788	-0.19476	3.17579
C	0.97864	0.74647	2.38963
C	0.56658	2.22108	2.49073
C	1.48258	3.21884	1.72589
C	1.22374	3.17246	0.22345
C	1.30119	4.6518	2.24012
O	1.6658	2.00963	-0.31806
O	0.68379	4.04545	-0.42269
C	1.36485	1.77379	-1.72712
C	2.29264	0.6463	-2.24278
C	-0.08635	1.40613	-1.8383
C	3.69884	0.63605	-1.66732
C	3.24553	-2.49002	-0.69994

C	4.28061	-1.47346	-0.17192
C	3.90111	0.03161	-0.28372
C	4.71357	1.16663	-2.36038
C	5.64802	-1.77062	-0.80525
O	-1.396	-4.02901	-0.54903
O	-1.28018	-0.1508	2.68789
C	-1.15256	2.16104	-2.20738
O	-2.28494	1.41075	-2.03423
C	-1.84698	0.21106	-1.53854
N	-0.54486	0.16305	-1.42005
C	-2.75976	-0.8651	-1.19874
C	-4.06577	-0.81607	-0.86021
C	-4.88975	0.4637	-0.76405
C	-4.78326	-2.09958	-0.52204
C	-5.92042	0.57669	-1.88915
O	-5.61658	0.5049	0.46564
C	-4.80922	0.7952	1.59437
H	-1.42103	-2.88428	1.85016
H	1.46802	-4.55923	-0.17823
H	0.92085	-3.74158	-1.65066
H	1.29149	-1.53009	-0.55435
H	-0.07079	-2.22437	3.89738
H	1.5983	-1.6638	3.6634
H	-0.0171	0.15066	4.2142
H	0.98435	0.43432	1.341
H	2.00809	0.62776	2.75559
H	0.57966	2.51946	3.5481
H	-0.47106	2.3384	2.15583
H	2.52424	2.90498	1.87979
H	1.93933	5.35431	1.69572
H	1.55576	4.7053	3.30401
H	0.26577	4.98271	2.11357
H	1.56021	2.70837	-2.25748
H	2.33408	0.75739	-3.33265
H	1.79882	-0.30814	-2.04907
H	3.63601	-3.49498	-0.48823
H	3.17968	-2.41854	-1.79505
H	4.36951	-1.65426	0.90774
H	3.00294	0.1983	0.31212

H	4.70491	0.58714	0.21666
H	5.72511	1.19832	-1.96319
H	4.5709	1.59549	-3.34995
H	5.94585	-2.81171	-0.63222
H	5.6321	-1.60025	-1.88783
H	6.4266	-1.12662	-0.37933
H	-1.2588	-0.44277	1.75953
H	-1.28262	3.17129	-2.55923
H	-2.26341	-1.83157	-1.18753
H	-4.22428	1.33422	-0.81003
H	-5.53877	-2.33706	-1.28352
H	-4.08588	-2.93938	-0.45923
H	-5.32147	-2.00018	0.42619
H	-6.48382	1.50804	-1.77598
H	-5.42319	0.57872	-2.86439
H	-6.62919	-0.25656	-1.85636
H	-4.04642	0.02589	1.78181
H	-4.29853	1.76466	1.48377
H	-5.47989	0.84431	2.45621

Compound 1c: Isomer 2S, 5R, 11S, 13S, 17S, 23R conformer 13

C	-1.93528	-2.03967	-1.29909
C	-3.01994	-2.73925	-1.72667
C	-3.75251	-3.63129	-0.83333
C	-3.11258	-3.80219	0.54673
C	-2.40439	-2.53134	1.02256
O	-1.51317	-2.02051	-0.01402
C	-1.10746	-1.12737	-2.1573
C	-1.64797	0.3237	-2.09418
C	-0.64953	1.34057	-2.64509
C	-1.16091	2.79205	-2.6246
C	-1.65149	3.349	-1.25975
C	-0.52628	3.38164	-0.23375
C	-2.24056	4.75536	-1.43305
O	-0.37685	2.18489	0.38849
O	0.17194	4.34645	0.00432
C	0.70524	2.08101	1.36972
C	0.37759	0.86876	2.24574

C	2.03547	1.94912	0.68276
C	-0.83863	1.01045	3.15643
C	-3.37575	-1.42955	1.47479
C	-2.73662	-0.07022	1.82556
C	-1.84574	-0.12103	3.0953
C	-0.98581	2.04346	3.99232
C	-3.82105	1.00694	1.96951
O	-4.75442	-4.25269	-1.16076
O	-2.8579	0.44992	-2.83852
C	2.88892	2.93469	0.29499
O	3.98248	2.35127	-0.27386
C	3.7444	1.00161	-0.20562
N	2.59161	0.71963	0.34569
C	4.79234	0.15539	-0.73841
C	4.89853	-1.18733	-0.64733
C	3.85693	-2.05644	0.03371
C	6.07164	-1.89888	-1.26628
C	2.76215	-2.47791	-0.96092
O	4.5385	-3.19104	0.57622
C	3.8408	-3.84169	1.61678
H	-3.31193	-2.70045	-2.7708
H	-3.87862	-4.11254	1.26337
H	-2.38689	-4.62378	0.46767
H	-1.71627	-2.77396	1.83638
H	-0.07626	-1.14996	-1.78876
H	-1.11857	-1.46291	-3.19908
H	-1.83035	0.56204	-1.03953
H	-0.40862	1.07535	-3.68334
H	0.2767	1.24887	-2.06658
H	-2.00515	2.87168	-3.31751
H	-0.3712	3.45252	-3.00638
H	-2.42904	2.67868	-0.87792
H	-2.63026	5.14394	-0.48641
H	-3.06099	4.73184	-2.15786
H	-1.4782	5.45445	-1.78932
H	0.69738	3.00678	1.94853
H	1.26406	0.66255	2.85947
H	0.26653	0.00235	1.58638
H	-4.12335	-1.27257	0.68578

H	-3.92906	-1.82066	2.34101
H	-2.09456	0.21508	0.98679
H	-2.48789	-0.10406	3.98566
H	-1.29506	-1.07181	3.12587
H	-1.83793	2.11511	4.66332
H	-0.26397	2.8553	4.04816
H	-4.38726	1.12826	1.0382
H	-4.53559	0.74521	2.76109
H	-3.37669	1.97446	2.2241
H	-3.50266	-0.16942	-2.45816
H	2.85645	4.01119	0.3216
H	5.57378	0.70836	-1.25331
H	3.38678	-1.48707	0.84575
H	6.73467	-1.19998	-1.78433
H	6.64151	-2.43418	-0.49983
H	5.73734	-2.65894	-1.98442
H	2.00866	-3.10448	-0.47162
H	2.25749	-1.59066	-1.35335
H	3.19304	-3.04321	-1.79415
H	3.60956	-3.15116	2.44355
H	2.90055	-4.30049	1.27599
H	4.49695	-4.63292	1.98989

Compound 1c: Isomer 2S, 5R, 11S, 13S, 17S, 23R conformer 16

C	0.75335	2.29315	-1.3455
C	0.33555	3.56035	-1.58824
C	0.92527	4.70726	-0.90198
C	2.09411	4.36488	0.02837
C	1.94436	2.97297	0.63619
O	1.67627	1.99232	-0.39992
C	0.25515	1.04805	-2.03283
C	1.37268	0.03355	-2.35286
C	0.81502	-1.32648	-2.77373
C	1.86729	-2.44731	-2.82932
C	2.64174	-2.74614	-1.51403
C	1.68554	-3.06872	-0.37281
C	3.6266	-3.90472	-1.71978
O	1.29364	-1.94567	0.28084

O	1.29584	-4.181	-0.08011
C	0.30165	-2.10579	1.34497
C	0.35844	-0.82141	2.17477
C	-1.06459	-2.34835	0.76679
C	1.5833	-0.61975	3.05953
C	3.18386	2.48207	1.38604
C	3.13526	1.01017	1.85039
C	2.16327	0.7813	3.04359
C	2.08734	-1.58684	3.83278
C	4.54381	0.5106	2.20157
O	0.57163	5.86421	-1.08577
O	2.2172	0.49183	-3.4125
C	-1.66449	-3.53714	0.49122
O	-2.91057	-3.2817	0.0019
C	-3.02293	-1.9137	-0.0002
N	-1.94076	-1.31945	0.43374
C	-4.30266	-1.40812	-0.46016
C	-4.79408	-0.15347	-0.37634
C	-4.03746	1.01883	0.23647
C	-6.16818	0.1517	-0.91521
C	-3.51169	1.97889	-0.8335
O	-4.89192	1.78764	1.08727
C	-5.16016	1.16749	2.33065
H	-0.42236	3.74849	-2.34037
H	3.02126	4.41721	-0.56145
H	2.16282	5.12664	0.81081
H	1.06045	2.96346	1.29107
H	-0.45682	0.56252	-1.34957
H	-0.28355	1.31789	-2.94711
H	1.95979	-0.09599	-1.43934
H	0.36225	-1.22166	-3.76851
H	0.00806	-1.60183	-2.0861
H	2.61444	-2.19508	-3.59038
H	1.37897	-3.37443	-3.15677
H	3.1977	-1.84665	-1.22866
H	4.23012	-4.08027	-0.82292
H	4.30475	-3.67956	-2.54965
H	3.09059	-4.8308	-1.94731
H	0.59526	-2.98145	1.92732

H	-0.53962	-0.79401	2.80619
H	0.25208	0.01802	1.48079
H	4.04815	2.61321	0.72078
H	3.34894	3.14796	2.24484
H	2.77467	0.41399	1.0054
H	2.69544	0.98444	3.98167
H	1.33538	1.50213	2.99493
H	2.92791	-1.40139	4.49637
H	1.68395	-2.59663	3.84601
H	5.21172	0.56226	1.33348
H	4.99002	1.11363	3.00352
H	4.51404	-0.53052	2.54005
H	2.71676	1.25453	-3.082
H	-1.35501	-4.56731	0.5523
H	-4.9228	-2.18122	-0.90814
H	-3.18965	0.63704	0.81761
H	-6.65404	-0.74465	-1.311
H	-6.80425	0.58745	-0.13744
H	-6.11742	0.89629	-1.72029
H	-2.93978	2.78256	-0.36009
H	-2.86154	1.44962	-1.53659
H	-4.33769	2.43189	-1.39108
H	-5.70114	0.21563	2.21618
H	-4.23316	0.97085	2.89162
H	-5.78204	1.86097	2.90309

Compound 1c: Isomer 2S, 5R, 11S, 13S, 17S, 23R conformer 19

C	1.39845	-1.97502	2.12917
C	0.10633	-2.39364	2.14433
C	-0.46458	-3.16399	1.04748
C	0.53041	-3.51432	-0.0565
C	1.57536	-2.41178	-0.2034
O	2.22871	-2.1614	1.07607
C	2.06172	-1.23261	3.25085
C	2.38342	0.23958	2.91909
C	1.12621	1.06611	2.59622
C	1.37681	2.57369	2.41703
C	2.22113	3.00631	1.18606

C	1.40106	2.90591	-0.09639
C	2.72967	4.44597	1.35043
O	1.48994	1.67427	-0.65494
O	0.74749	3.8112	-0.57049
C	0.79501	1.47731	-1.92519
C	1.46042	0.29546	-2.66561
C	-0.65901	1.22415	-1.66652
C	2.97869	0.26682	-2.61804
C	2.64132	-2.73126	-1.25075
C	3.89803	-1.83537	-1.30104
C	3.65632	-0.2998	-1.37898
C	3.68992	0.75431	-3.6421
C	4.83206	-2.32484	-2.41722
O	-1.63372	-3.53719	1.0132
O	3.06041	0.72765	4.08155
C	-1.73017	2.02806	-1.88673
O	-2.85146	1.36233	-1.47124
C	-2.39722	0.16109	-0.98823
N	-1.0981	0.0378	-1.09589
C	-3.28898	-0.83722	-0.43685
C	-4.61673	-0.78575	-0.1938
C	-5.51312	0.41265	-0.4922
C	-5.30302	-2.00249	0.37566
C	-6.22991	0.26097	-1.83722
O	-6.53066	0.5604	0.49922
C	-6.05654	1.10339	1.7165
H	-0.52104	-2.18227	3.00283
H	1.01676	-4.46709	0.20015
H	-0.01239	-3.66046	-0.99509
H	1.04949	-1.48094	-0.44193
H	1.42622	-1.26259	4.14158
H	3.00861	-1.72579	3.50154
H	3.06716	0.25036	2.05752
H	0.419	0.93858	3.4267
H	0.64412	0.65217	1.7035
H	1.877	2.95667	3.31706
H	0.41374	3.09766	2.37368
H	3.07614	2.32797	1.07974
H	3.33025	4.75869	0.49013

H	3.35035	4.52952	2.24941
H	1.89098	5.14242	1.44001
H	0.9146	2.40037	-2.49692
H	1.12096	0.36316	-3.70605
H	1.04203	-0.631	-2.26812
H	2.98061	-3.76445	-1.09263
H	2.1366	-2.72819	-2.22702
H	4.41746	-1.98202	-0.34481
H	3.09053	0.00058	-0.49415
H	4.64459	0.16964	-1.28911
H	4.77701	0.77279	-3.62772
H	3.21311	1.15608	-4.53368
H	5.0608	-3.39094	-2.30123
H	4.38202	-2.18161	-3.40629
H	5.78184	-1.77703	-2.40366
H	3.46993	1.5761	3.85664
H	-1.86692	3.02364	-2.2766
H	-2.7555	-1.75058	-0.1882
H	-4.91054	1.32831	-0.51799
H	-4.58721	-2.80602	0.56665
H	-5.82466	-1.76291	1.30826
H	-6.07237	-2.3756	-0.31366
H	-6.88133	1.1246	-2.00392
H	-5.50234	0.20136	-2.65232
H	-6.84864	-0.64198	-1.85132
H	-5.30776	0.4577	2.19999
H	-5.60773	2.09806	1.56587
H	-6.9208	1.20031	2.37914

Compound 1c: Isomer 2S, 5R, 11S, 13S, 17S, 23R conformer 22

C	2.906	-2.13185	0.39122
C	3.70354	-3.21367	0.58751
C	5.09609	-3.22984	0.14391
C	5.58127	-1.91092	-0.46379
C	4.4521	-1.1744	-1.18128
O	3.29665	-1.04215	-0.31048
C	1.53117	-1.94194	0.96442
C	1.52499	-0.82965	2.04062

C	0.10883	-0.43361	2.45449
C	0.05713	0.77097	3.41013
C	0.63019	2.11099	2.87041
C	-0.17027	2.59899	1.67091
C	0.63297	3.18177	3.96932
O	0.27358	2.04637	0.5103
O	-1.10489	3.37124	1.72428
C	-0.47298	2.39183	-0.69563
C	0.41474	2.07982	-1.91228
C	-1.7599	1.62149	-0.73611
C	1.79638	2.71075	-1.86505
C	4.85021	0.21957	-1.66936
C	3.75608	1.0889	-2.34435
C	2.91757	1.86592	-1.28776
C	2.00535	3.95077	-2.32098
C	2.91188	0.28659	-3.34568
O	5.84618	-4.18421	0.2969
O	2.2097	-1.25082	3.21978
C	-3.02446	2.06055	-0.50838
O	-3.87596	0.99974	-0.65566
C	-3.06678	-0.06508	-0.9569
N	-1.80176	0.26243	-1.02051
C	-3.60904	-1.38646	-1.19906
C	-4.86396	-1.85751	-1.03687
C	-6.04404	-1.0311	-0.53152
C	-5.17628	-3.28676	-1.40397
C	-6.87902	-0.47362	-1.68782
O	-6.92288	-1.82469	0.26588
C	-6.43902	-2.0627	1.57443
H	3.32626	-4.07608	1.12569
H	5.97913	-1.2863	0.34978
H	6.40794	-2.11677	-1.1502
H	4.11477	-1.7884	-2.02838
H	0.84787	-1.65453	0.15552
H	1.17689	-2.87689	1.40984
H	2.02802	0.04446	1.60573
H	-0.35353	-1.29491	2.95474
H	-0.47418	-0.23444	1.54946
H	0.61826	0.52176	4.31757

H	-0.98352	0.94172	3.71585
H	1.65752	1.93903	2.53177
H	1.07149	4.11944	3.61199
H	1.21625	2.83616	4.82927
H	-0.38653	3.39818	4.30203
H	-0.69034	3.46138	-0.64577
H	-0.13844	2.43641	-2.78986
H	0.48856	0.99275	-2.00658
H	5.28694	0.77407	-0.82769
H	5.66555	0.05963	-2.38819
H	4.29374	1.85277	-2.92454
H	2.49938	1.15915	-0.56927
H	3.61031	2.51176	-0.73222
H	2.98423	4.42236	-2.26831
H	1.20838	4.54563	-2.76247
H	3.55306	-0.244	-4.06074
H	2.28	-0.45561	-2.84529
H	2.25909	0.95038	-3.921
H	3.10252	-1.53317	2.96294
H	-3.46071	3.0061	-0.2306
H	-2.84319	-2.06203	-1.57136
H	-5.67715	-0.19261	0.0721
H	-4.29945	-3.79369	-1.81652
H	-5.53345	-3.85248	-0.53672
H	-5.98215	-3.33235	-2.1476
H	-7.73201	0.08216	-1.28636
H	-6.27506	0.19799	-2.30507
H	-7.26375	-1.28163	-2.3183
H	-5.49517	-2.62922	1.576
H	-6.2757	-1.12064	2.12082
H	-7.20291	-2.64713	2.09402

Compound 1c: Isomer 2S, 5R, 11S, 13S, 17S, 23R conformer 23

C	0.18187	2.81215	-1.65089
C	-0.79801	3.68324	-1.29475
C	-0.62963	4.62333	-0.19073
C	0.75084	4.56806	0.46681
C	1.29009	3.14331	0.45073

O	1.29816	2.61691	-0.90905
C	0.18645	2.05011	-2.94869
C	0.27867	0.50192	-2.92198
C	1.59808	-0.04667	-2.36666
C	1.81121	-1.53472	-2.68117
C	2.94715	-2.20259	-1.85091
C	2.38442	-2.718	-0.53214
C	3.63377	-3.34144	-2.61172
O	1.86119	-1.70912	0.21289
O	2.35625	-3.88009	-0.18654
C	1.07413	-2.09924	1.3778
C	0.98201	-0.86756	2.27893
C	-0.27132	-2.58001	0.92264
C	2.29462	-0.36546	2.87
C	2.71313	2.98455	0.98238
C	3.09028	1.52617	1.32176
C	2.54521	1.12017	2.72127
C	3.14525	-1.17127	3.5134
C	4.60587	1.3036	1.24514
O	-1.47871	5.43674	0.14914
O	-0.85689	-0.09065	-2.2958
C	-0.71676	-3.85806	0.82127
O	-1.99358	-3.8265	0.34533
C	-2.28157	-2.4969	0.16942
N	-1.27572	-1.71864	0.48419
C	-3.62272	-2.23037	-0.31696
C	-4.3445	-1.09404	-0.22624
C	-3.86042	0.17773	0.45861
C	-5.73546	-1.04486	-0.80472
C	-3.61369	1.3142	-0.5335
O	-4.84451	0.64736	1.38656
C	-4.92347	-0.11867	2.57311
H	-1.69199	3.76825	-1.90143
H	1.42874	5.23653	-0.08402
H	0.67741	4.94271	1.49213
H	0.59332	2.50512	1.01604
H	-0.724	2.31953	-3.49196
H	1.03544	2.4204	-3.54284
H	0.19686	0.20117	-3.97604

H	1.61422	0.11231	-1.28629
H	2.43511	0.53681	-2.77293
H	2.05804	-1.64541	-3.74517
H	0.87138	-2.07947	-2.52909
H	3.69048	-1.43008	-1.60801
H	4.40561	-3.81994	-2.00201
H	4.10019	-2.95488	-3.52418
H	2.91116	-4.11404	-2.8932
H	1.59781	-2.91976	1.8722
H	0.28796	-1.11266	3.09433
H	0.49315	-0.07341	1.70548
H	3.39361	3.39028	0.2231
H	2.82893	3.6149	1.87461
H	2.62408	0.87837	0.57009
H	3.25217	1.46545	3.48677
H	1.60096	1.65016	2.9135
H	4.05694	-0.7893	3.96543
H	2.96935	-2.23913	3.62231
H	4.9807	1.4872	0.23141
H	5.13903	1.97975	1.92661
H	4.86448	0.27568	1.52117
H	-0.68884	-0.22593	-1.34586
H	-0.2784	-4.82582	1.00298
H	-4.07991	-3.10065	-0.78269
H	-2.9283	-0.03455	0.99709
H	-6.05976	-2.02569	-1.16408
H	-6.4502	-0.67929	-0.06035
H	-5.78034	-0.34388	-1.64854
H	-3.25955	2.19773	0.00591
H	-2.86223	1.0223	-1.27309
H	-4.53937	1.58499	-1.05166
H	-5.24305	-1.15502	2.38428
H	-3.95653	-0.14728	3.1
H	-5.66287	0.3677	3.21512

Compound 1c: Isomer 2S, 5R, 11S, 13S, 17S, 23R conformer 27

C	-2.14792	2.65372	1.24769
C	-1.92057	3.80776	0.56744

C	-2.77686	4.23197	-0.53494
C	-3.95014	3.28699	-0.80905
C	-3.5434	1.8469	-0.52554
O	-3.03917	1.71999	0.83543
C	-1.51176	2.31975	2.56819
C	-0.57136	1.08789	2.64677
C	-1.26856	-0.26037	2.42323
C	-0.39935	-1.45608	2.84234
C	-0.91716	-2.83795	2.34185
C	-0.37696	-3.10992	0.94437
C	-0.52713	-3.9788	3.28723
O	-0.87804	-2.22473	0.0405
O	0.42759	-3.96692	0.65139
C	-0.26217	-2.20813	-1.27054
C	-1.19426	-1.39282	-2.17313
C	1.09908	-1.57979	-1.17939
C	-2.59393	-1.95377	-2.39337
C	-4.65513	0.80876	-0.66785
C	-4.12518	-0.63555	-0.8052
C	-3.71155	-0.94004	-2.27528
C	-2.81812	-3.23254	-2.71068
C	-5.14389	-1.6675	-0.30534
O	-2.63176	5.27977	-1.15146
O	0.55758	1.25762	1.80138
C	2.27754	-1.99934	-1.70299
O	3.23437	-1.06673	-1.39849
C	2.58663	-0.10946	-0.67107
N	1.30851	-0.37146	-0.52802
C	3.27005	1.05152	-0.14064
C	4.57657	1.39029	-0.19797
C	5.66358	0.57471	-0.89359
C	5.02944	2.68935	0.41964
C	5.9033	1.06564	-2.32477
O	6.91007	0.68449	-0.20728
C	6.98055	-0.10336	0.96655
H	-1.16008	4.49722	0.91538
H	-4.79428	3.57847	-0.16699
H	-4.26997	3.40198	-1.84909
H	-2.69776	1.59582	-1.18467

H	-0.93506	3.19303	2.88645
H	-2.31994	2.17914	3.30093
H	-0.17924	1.10272	3.67533
H	-1.53873	-0.3437	1.36778
H	-2.21244	-0.28077	2.98433
H	-0.34816	-1.49328	3.93845
H	0.62943	-1.30095	2.49564
H	-2.01205	-2.78525	2.26562
H	-0.87177	-4.94623	2.90972
H	-0.96883	-3.81434	4.27589
H	0.56011	-4.03781	3.39884
H	-0.16025	-3.23814	-1.62175
H	-0.69327	-1.27932	-3.1443
H	-1.25422	-0.3839	-1.74998
H	-5.30429	0.89949	0.21205
H	-5.27219	1.06335	-1.54012
H	-3.23432	-0.72172	-0.17129
H	-4.59685	-1.28266	-2.82623
H	-3.38716	-0.00972	-2.76378
H	-3.82199	-3.59926	-2.90837
H	-2.01832	-3.96501	-2.79221
H	-5.35941	-1.52527	0.76004
H	-6.09202	-1.58477	-0.853
H	-4.76537	-2.68673	-0.44014
H	0.45767	0.73064	0.98268
H	2.60069	-2.86317	-2.26113
H	2.58177	1.71247	0.379
H	5.37148	-0.48114	-0.92945
H	5.54174	3.31617	-0.32126
H	4.18496	3.25275	0.82517
H	5.75418	2.51779	1.22297
H	6.70708	0.47967	-2.78118
H	4.99535	0.95311	-2.9248
H	6.20127	2.11897	-2.3323
H	6.23303	0.19487	1.71734
H	6.83672	-1.17213	0.74333
H	7.97983	0.04086	1.38578

Compound 1c: Isomer 2S, 5R, 11S, 13S, 17S, 23R conformer 29

C	5.24545	0.35129	0.23434
C	5.78352	-0.03132	-0.95027
C	5.78144	-1.4265	-1.37508
C	5.2492	-2.39888	-0.32007
C	4.1092	-1.77767	0.48604
O	4.53896	-0.4889	1.03767
C	5.39301	1.72221	0.83765
C	4.07522	2.41007	1.27479
C	3.06735	2.55951	0.12299
C	1.76651	3.24255	0.56174
C	0.68604	3.30471	-0.53945
C	0.29455	1.90977	-1.00852
C	-0.54807	4.09799	-0.07445
O	-0.33513	1.20485	-0.03663
O	0.5195	1.46517	-2.11703
C	-0.79703	-0.13128	-0.40469
C	-0.9347	-0.93826	0.89588
C	-2.09338	-0.03187	-1.15612
C	0.35629	-1.23523	1.6429
C	2.79758	-1.60209	-0.31287
C	1.71345	-2.67692	-0.05454
C	1.06448	-2.54498	1.3483
C	0.80943	-0.39958	2.58495
C	2.20963	-4.11343	-0.28497
O	6.22863	-1.81634	-2.44542
O	3.51002	1.76566	2.41084
C	-2.28054	0.07206	-2.49943
O	-3.62102	0.15686	-2.73579
C	-4.20633	0.09931	-1.49526
N	-3.33205	-0.00451	-0.52816
C	-5.65317	0.17728	-1.48518
C	-6.48426	0.00437	-0.4354
C	-6.0077	-0.31044	0.97772
C	-7.97165	0.16288	-0.6178
C	-5.86146	0.96177	1.81644
O	-6.93744	-1.15571	1.65829
C	-6.84988	-2.51378	1.27337
H	6.29274	0.69441	-1.57411

H	4.92358	-3.31952	-0.80987
H	6.08391	-2.65529	0.34745
H	3.92367	-2.36396	1.38911
H	6.02175	1.63489	1.73418
H	5.92806	2.35618	0.12298
H	4.34561	3.41238	1.6309
H	2.84826	1.56878	-0.29621
H	3.5379	3.13052	-0.69026
H	1.98447	4.27147	0.88027
H	1.36634	2.72216	1.43721
H	1.10901	3.78614	-1.42867
H	-1.31054	4.14788	-0.85962
H	-0.25882	5.12345	0.18059
H	-0.99998	3.63793	0.80945
H	-0.04223	-0.55676	-1.06756
H	-1.43474	-1.87904	0.63274
H	-1.62902	-0.39419	1.54302
H	2.37424	-0.62856	-0.04866
H	3.01937	-1.56925	-1.3861
H	0.92789	-2.49455	-0.80048
H	0.33954	-3.36629	1.45335
H	1.82772	-2.71842	2.11842
H	1.70693	-0.61689	3.1599
H	0.29372	0.52407	2.83116
H	2.65026	-4.22818	-1.28199
H	2.9648	-4.41117	0.4533
H	1.38089	-4.82606	-0.20361
H	3.43149	0.8234	2.18484
H	-1.62073	0.12742	-3.34966
H	-6.07716	0.41104	-2.45882
H	-5.0296	-0.80463	0.92963
H	-8.21906	0.46555	-1.63943
H	-8.50316	-0.76739	-0.38928
H	-8.37134	0.91649	0.07269
H	-5.55654	0.69298	2.83265
H	-5.10078	1.61426	1.37866
H	-6.80907	1.50739	1.87689
H	-7.08292	-2.66475	0.20779
H	-5.84595	-2.92341	1.4661

H	-7.57947	-3.06309	1.87469

Compound 1c: Isomer 2S, 5R, 11S, 13S, 17S, 23R conformer 30

C	-1.52704	2.67842	1.32865
C	-1.21378	3.78064	0.59823
C	-2.1221	4.31723	-0.41072
C	-3.44498	3.55471	-0.52332
C	-3.22052	2.07219	-0.25617
O	-2.58568	1.88276	1.0424
C	-0.79504	2.26094	2.57434
C	0.00909	0.93343	2.54424
C	-0.85179	-0.32573	2.37773
C	-0.07211	-1.61719	2.66615
C	-0.78173	-2.92164	2.19512
C	-0.45584	-3.17114	0.72891
C	-0.37953	-4.13519	3.03923
O	-0.99972	-2.21277	-0.06624
O	0.23594	-4.07107	0.3028
C	-0.55796	-2.1823	-1.4519
C	-1.56347	-1.31424	-2.20977
C	0.82217	-1.59631	-1.51193
C	-3.01666	-1.76864	-2.19636
C	-4.47773	1.20317	-0.24799
C	-4.17741	-0.30104	-0.43619
C	-4.01613	-0.65936	-1.9451
C	-3.38692	-3.03325	-2.41847
C	-5.24876	-1.18415	0.21566
O	-1.89618	5.32542	-1.06726
O	1.06788	0.98754	1.59654
C	1.9962	-2.18352	-1.85283
O	2.98683	-1.24257	-1.75703
C	2.35953	-0.1044	-1.33427
N	1.06926	-0.26703	-1.183
C	3.08273	1.13625	-1.12202
C	4.38382	1.33317	-0.82689
C	5.39119	0.21759	-0.56655
C	4.90164	2.73769	-0.64632
C	6.56756	0.22953	-1.54255

O	5.94055	0.37186	0.7455
C	5.05175	-0.01858	1.78239
H	-0.32851	4.35621	0.8433
H	-4.15475	3.97103	0.20649
H	-3.8661	3.7099	-1.52114
H	-2.50056	1.6976	-1.
H	-0.09623	3.06158	2.83289
H	-1.5333	2.19543	3.38642
H	0.51164	0.88355	3.52035
H	-1.247	-0.34749	1.35922
H	-1.72292	-0.26535	3.04409
H	0.09871	-1.69919	3.74781
H	0.92103	-1.55214	2.20503
H	-1.86694	-2.76738	2.26999
H	-0.86074	-5.04955	2.67912
H	-0.66955	-3.97874	4.08376
H	0.70244	-4.29586	3.00018
H	-0.53541	-3.20847	-1.82541
H	-1.21471	-1.24067	-3.24947
H	-1.48472	-0.30145	-1.80198
H	-4.99216	1.37855	0.70515
H	-5.15711	1.5496	-1.03841
H	-3.22812	-0.51264	0.07086
H	-4.99931	-0.92976	-2.35087
H	-3.69003	0.23209	-2.50003
H	-4.43496	-3.32034	-2.44438
H	-2.66778	-3.83214	-2.58426
H	-5.29487	-1.01614	1.29798
H	-6.24282	-0.97194	-0.19957
H	-5.03392	-2.24542	0.04787
H	0.73059	0.73769	0.71435
H	2.29115	-3.17614	-2.15206
H	2.4242	1.9997	-1.17585
H	4.89181	-0.75525	-0.63028
H	5.61061	3.00009	-1.443
H	4.09172	3.47205	-0.66283
H	5.4448	2.82408	0.30033
H	7.24163	-0.60074	-1.31109
H	6.21413	0.12108	-2.5734

H	7.13646	1.1607	-1.46116
H	4.11096	0.5494	1.7691
H	4.80978	-1.09107	1.71469
H	5.57191	0.16767	2.72568

Compound 1c: Isomer 2S, 5R, 11S, 13S, 17S, 23R conformer 31

C	1.4311	-2.46552	1.81841
C	0.18605	-2.9663	1.58771
C	-0.20795	-3.47024	0.27871
C	0.91396	-3.46545	-0.75558
C	1.85995	-2.28935	-0.5225
O	2.35954	-2.3019	0.84963
C	1.88348	-1.95637	3.15764
C	1.36205	-0.51728	3.45597
C	1.85358	0.5194	2.43808
C	1.45041	1.9559	2.79501
C	2.00907	3.05844	1.85356
C	1.29655	3.09825	0.50326
C	1.93522	4.44058	2.51439
O	1.55557	1.98788	-0.23392
O	0.5959	4.00671	0.10983
C	0.94786	1.9181	-1.55853
C	1.73768	0.89363	-2.40631
C	-0.49386	1.52772	-1.4238
C	3.24659	0.92179	-2.23483
C	3.048	-2.28684	-1.48481
C	4.21521	-1.31412	-1.20707
C	3.84651	0.18927	-1.04335
C	4.01129	1.5921	-3.10528
C	5.31102	-1.52212	-2.26299
O	-1.32896	-3.89908	0.02173
O	-0.05504	-0.50126	3.58394
C	-1.61202	2.28306	-1.56655
O	-2.69688	1.48614	-1.31609
C	-2.1744	0.2555	-1.00793
N	-0.86623	0.23787	-1.06822
C	-3.01594	-0.87115	-0.66717
C	-4.34659	-0.92892	-0.44306

C	-5.28607	0.26079	-0.55827
C	-4.97568	-2.25127	-0.08802
C	-5.82335	0.40276	-1.99344
O	-6.34956	0.05623	0.37512
C	-7.02325	1.2373	0.75524
H	-0.5163	-3.05736	2.40893
H	1.46399	-4.41496	-0.67777
H	0.47786	-3.41597	-1.75767
H	1.27707	-1.36462	-0.60472
H	1.50481	-2.61192	3.9477
H	2.97823	-1.9641	3.19362
H	1.72972	-0.25153	4.45474
H	1.45856	0.26038	1.45067
H	2.94779	0.45088	2.36192
H	1.81956	2.18373	3.80446
H	0.35799	2.03168	2.85056
H	3.05999	2.81529	1.64171
H	2.32665	5.21983	1.85409
H	2.51835	4.44496	3.44147
H	0.90024	4.70298	2.75495
H	1.02351	2.91597	-1.99655
H	1.47447	1.09906	-3.45073
H	1.34624	-0.10039	-2.18161
H	3.46591	-3.30272	-1.51144
H	2.63917	-2.10459	-2.48865
H	4.63348	-1.6031	-0.23371
H	3.17205	0.28459	-0.1908
H	4.77431	0.70253	-0.75908
H	5.09114	1.65127	-2.99266
H	3.58739	2.11166	-3.96206
H	5.62795	-2.57112	-2.30207
H	4.96167	-1.23747	-3.26204
H	6.1956	-0.91518	-2.03624
H	-0.42586	-0.71275	2.70939
H	-1.80577	3.31786	-1.79733
H	-2.44196	-1.78856	-0.57397
H	-4.75084	1.18029	-0.28939
H	-5.75003	-2.52731	-0.81627
H	-4.22662	-3.04652	-0.05178

H	-5.47966	-2.18374	0.8816
H	-6.5274	1.23885	-2.06552
H	-5.002	0.59147	-2.69099
H	-6.34364	-0.51058	-2.29981
H	-7.75345	0.95177	1.51739
H	-6.33089	1.97857	1.18575
H	-7.55933	1.70994	-0.08151

Compound 1cc: Isomer 2S, 5R, 11S, 13S, 17S, 23R conformer 38

C	-1.96066	2.36044	0.76331
C	-2.17945	3.69382	0.64622
C	-3.41179	4.2222	0.06868
C	-4.45168	3.15865	-0.30192
C	-3.79313	1.84507	-0.71406
O	-2.82138	1.43051	0.28114
C	-0.75275	1.71278	1.38918
C	-1.09395	0.46261	2.2261
C	0.15165	-0.33713	2.60884
C	-0.15054	-1.72084	3.21069
C	-0.97197	-2.70363	2.32865
C	-0.30373	-2.91367	0.97633
C	-1.15176	-4.0499	3.0429
O	-0.73847	-2.00593	0.06401
O	0.52846	-3.76165	0.72834
C	-0.09028	-2.01316	-1.24473
C	-0.99237	-1.19449	-2.17202
C	1.28735	-1.42339	-1.14672
C	-2.33164	-1.80876	-2.56185
C	-4.77034	0.6795	-0.87648
C	-4.11901	-0.70084	-1.11187
C	-3.50697	-0.85083	-2.53459
C	-2.46212	-3.08356	-2.94271
C	-5.12927	-1.82474	-0.84133
O	-3.64383	5.41675	-0.06347
O	-1.76839	0.80402	3.44153
C	2.48486	-2.06313	-1.11675
O	3.4706	-1.11766	-1.03938
C	2.81067	0.08294	-1.01005

N	1.51076	-0.0536	-1.07702
C	3.52449	1.34182	-0.94566
C	4.8354	1.59072	-0.73877
C	5.89884	0.51874	-0.51506
C	5.33444	3.01375	-0.75845
C	6.62466	0.16344	-1.81605
O	6.89533	0.96712	0.40315
C	6.47797	0.92819	1.7551
H	-1.44941	4.39939	1.02687
H	-5.09464	2.9952	0.57562
H	-5.08752	3.54506	-1.10422
H	-3.221	2.00942	-1.6393
H	-0.08742	1.40111	0.57046
H	-0.21591	2.44391	2.00259
H	-1.73814	-0.17017	1.60924
H	0.72541	0.24473	3.34246
H	0.78478	-0.43995	1.72119
H	-0.70766	-1.58648	4.14464
H	0.79846	-2.20766	3.47107
H	-1.95566	-2.26048	2.14096
H	-1.78991	-4.72677	2.46472
H	-1.61536	-3.89812	4.02343
H	-0.18564	-4.543	3.18554
H	-0.00841	-3.05404	-1.56515
H	-0.42179	-0.98459	-3.08709
H	-1.14742	-0.22237	-1.69444
H	-5.38159	0.62967	0.03493
H	-5.45815	0.92272	-1.6987
H	-3.3064	-0.80485	-0.38521
H	-4.28954	-1.18676	-3.22691
H	-3.16876	0.12809	-2.90148
H	-3.41936	-3.48339	-3.26711
H	-1.6265	-3.7796	-2.95459
H	-5.48322	-1.79933	0.19612
H	-6.00599	-1.73584	-1.49654
H	-4.67585	-2.80607	-1.01687
H	-2.64811	1.13722	3.20585
H	2.79247	-3.09596	-1.11867
H	2.85258	2.18248	-1.09869

H	5.43137	-0.38933	-0.11607
H	4.52866	3.71614	-0.98913
H	5.78014	3.29379	0.20208
H	6.12657	3.13973	-1.50747
H	7.39682	-0.58407	-1.60962
H	5.92	-0.24481	-2.5465
H	7.10963	1.04489	-2.24777
H	5.62126	1.59165	1.94892
H	6.19933	-0.09229	2.06103
H	7.32633	1.26102	2.35884

Compound 1c: Isomer 2S, 5R, 11S, 13S, 17S, 23R conformer 42

C	-0.5658	2.26511	1.42417
C	-0.0333	3.48346	1.69276
C	-0.52023	4.69481	1.03726
C	-1.7213	4.48055	0.10969
C	-1.69369	3.09712	-0.5341
O	-1.51561	2.0713	0.47754
C	-0.17432	0.96469	2.07633
C	-1.36863	0.03475	2.37345
C	-0.91686	-1.37624	2.75199
C	-2.05169	-2.41489	2.77151
C	-2.83829	-2.61457	1.44494
C	-1.90395	-2.97383	0.2962
C	-3.91001	-3.69997	1.61157
O	-1.4144	-1.86442	-0.3127
O	-1.60885	-4.10359	-0.03767
C	-0.44567	-2.06755	-1.39207
C	-0.40587	-0.75473	-2.17723
C	0.90165	-2.44479	-0.84152
C	-1.60883	-0.43518	-3.05738
C	-2.96667	2.73392	-1.29957
C	-3.03444	1.27734	-1.80823
C	-2.07753	1.0061	-3.00479
C	-2.18502	-1.3389	-3.85647
C	-4.47639	0.90266	-2.17821
O	-0.05884	5.80981	1.24087
O	-2.17009	0.52704	3.45112

C	1.38675	-3.68985	-0.5891
O	2.66483	-3.5643	-0.13338
C	2.91469	-2.2146	-0.12861
N	1.88546	-1.51068	-0.5278
C	4.25425	-1.84509	0.28422
C	4.85663	-0.63972	0.20761
C	4.16193	0.6111	-0.29689
C	6.28298	-0.47746	0.66147
C	3.58094	1.42103	0.87501
O	5.13538	1.36543	-1.02492
C	4.5857	2.31844	-1.91087
H	0.7418	3.58421	2.44425
H	-2.63706	4.59594	0.70819
H	-1.72888	5.26561	-0.65243
H	-0.81101	3.02745	-1.1872
H	0.49308	0.44096	1.37675
H	0.38812	1.1629	2.99449
H	-1.96779	-0.02184	1.46023
H	-0.46051	-1.33662	3.74987
H	-0.13142	-1.69192	2.05677
H	-2.78178	-2.12853	3.53704
H	-1.63702	-3.38556	3.07321
H	-3.32266	-1.66732	1.18487
H	-4.5179	-3.80374	0.70656
H	-4.57567	-3.44707	2.44361
H	-3.44806	-4.67033	1.81563
H	-0.81734	-2.89453	-2.00024
H	0.49471	-0.76975	-2.80517
H	-0.24304	0.05105	-1.45454
H	-3.81982	2.91504	-0.63169
H	-3.0734	3.43688	-2.1378
H	-2.72693	0.62947	-0.98043
H	-2.58725	1.27661	-3.93825
H	-1.19559	1.65788	-2.93468
H	-3.00613	-1.07089	-4.51631
H	-1.8624	-2.37657	-3.89613
H	-5.14303	0.98386	-1.31142
H	-4.86814	1.56122	-2.96479
H	-4.52747	-0.12774	-2.54549

H	-2.61615	1.33131	3.14352
H	0.97458	-4.68334	-0.6499
H	4.82339	-2.68555	0.67351
H	3.34212	0.32481	-0.96751
H	6.68763	-1.41468	1.05421
H	6.90811	-0.13692	-0.17047
H	6.36538	0.28998	1.44194
H	3.04782	2.30792	0.517
H	2.87287	0.80908	1.44113
H	4.37915	1.7496	1.54889
H	3.89547	1.85084	-2.63128
H	4.04907	3.12404	-1.38835
H	5.4233	2.75853	-2.4589

Compound 1c: Isomer 2S, 5R, 11S, 13S, 17S, 23R conformer 46

C	-0.84672	2.31585	1.31059
C	-0.48887	3.607	1.51916
C	-1.14527	4.70518	0.81748
C	-2.31406	4.27837	-0.07679
C	-2.09292	2.88927	-0.66876
O	-1.74252	1.93983	0.36982
C	-0.2719	1.11962	2.02427
C	-1.32141	0.06429	2.39557
C	-0.68532	-1.28615	2.75932
C	-1.68572	-2.45156	2.86291
C	-2.49513	-2.79568	1.57941
C	-1.55984	-3.1078	0.41667
C	-3.43661	-3.98059	1.83179
O	-1.21597	-1.98439	-0.25817
O	-1.15039	-4.21472	0.13157
C	-0.23882	-2.13018	-1.34041
C	-0.34846	-0.85936	-2.18564
C	1.14267	-2.32902	-0.78413
C	-1.59018	-0.71596	-3.05881
C	-3.31922	2.31623	-1.38181
C	-3.19603	0.84298	-1.82622
C	-2.23878	0.65397	-3.03751
C	-2.05177	-1.7072	-3.82818

C	-4.58019	0.25695	-2.13825
O	-0.84734	5.88362	0.96288
O	-2.08248	0.60492	3.48265
C	1.78864	-3.49888	-0.53331
O	3.0318	-3.20533	-0.05886
C	3.09495	-1.83385	-0.03959
N	1.98606	-1.27345	-0.45037
C	4.36262	-1.29	0.40974
C	4.80547	-0.01618	0.34652
C	3.99435	1.14146	-0.22322
C	6.1773	0.3274	0.86764
C	3.45169	2.05226	0.88097
O	4.80439	1.96346	-1.06725
C	5.07118	1.38763	-2.33204
H	0.25841	3.85	2.26612
H	-3.22558	4.27981	0.5386
H	-2.45032	5.0215	-0.86827
H	-1.2265	2.92514	-1.3463
H	0.45699	0.65045	1.34902
H	0.26223	1.44626	2.92251
H	-1.9666	-0.07003	1.52166
H	-0.17356	-1.17522	3.7241
H	0.08335	-1.53018	2.01812
H	-2.41409	-2.24159	3.65925
H	-1.14867	-3.3548	3.17941
H	-3.08634	-1.91965	1.28959
H	-4.05835	-4.18927	0.95494
H	-4.09956	-3.76569	2.67694
H	-2.86496	-4.8854	2.05762
H	-0.5197	-3.02072	-1.90606
H	0.54095	-0.8112	-2.82779
H	-0.26392	-0.00716	-1.50441
H	-4.17272	2.40711	-0.69659
H	-3.5434	2.95759	-2.24602
H	-2.78245	0.28238	-0.98112
H	-2.79912	0.82431	-3.96574
H	-1.44736	1.41552	-3.00906
H	-2.90726	-1.56425	-4.48311
H	-1.59707	-2.69499	-3.84732

H	-5.2317	0.28709	-1.25671
H	-5.07752	0.81875	-2.94011
H	-4.49866	-0.78691	-2.45987
H	-2.89722	0.08712	3.56424
H	1.51584	-4.53882	-0.60426
H	5.01885	-2.04886	0.82979
H	3.15145	0.74274	-0.80004
H	6.7018	-0.55851	1.23676
H	6.78333	0.80063	0.08776
H	6.11465	1.05408	1.68803
H	2.83326	2.84004	0.44045
H	2.8428	1.47815	1.58574
H	4.27001	2.52856	1.43042
H	5.65817	2.118	-2.89532
H	5.64676	0.45248	-2.25378
H	4.14147	1.17417	-2.88223

Compound 1c: Isomer 2S, 5R, 11S, 13S, 17S, 23R conformer 49

C	-2.32167	2.65158	1.16352
C	-2.13369	3.80825	0.47586
C	-2.97846	4.17678	-0.65484
C	-4.09475	3.17132	-0.95046
C	-3.62705	1.7563	-0.63593
O	-3.15541	1.67097	0.73972
C	-1.70425	2.36331	2.50361
C	-0.72296	1.16741	2.62348
C	-1.36948	-0.20818	2.41526
C	-0.47439	-1.36621	2.88262
C	-0.94499	-2.7747	2.40973
C	-0.35836	-3.07494	1.037
C	-0.557	-3.87781	3.39968
O	-0.84573	-2.22282	0.09437
O	0.46861	-3.92552	0.79193
C	-0.18223	-2.22309	-1.19318
C	-1.09463	-1.44579	-2.14824
C	1.16383	-1.56942	-1.06304
C	-2.47001	-2.04634	-2.41341
C	-4.6852	0.66572	-0.79361

C	-4.08854	-0.75563	-0.89101
C	-3.61953	-1.06437	-2.34302
C	-2.64674	-3.33388	-2.72558
C	-5.07577	-1.82302	-0.40274
O	-2.86854	5.22376	-1.28014
O	0.41112	1.36517	1.79128
C	2.36583	-1.97019	-1.54569
O	3.29692	-1.02176	-1.20926
C	2.60872	-0.07449	-0.50517
N	1.33058	-0.35718	-0.40688
C	3.25907	1.08878	0.05812
C	4.56202	1.44479	0.04112
C	5.65858	0.65737	-0.65946
C	4.99013	2.7223	0.7144
C	5.77589	1.0744	-2.13655
O	6.8764	0.90369	0.04681
C	7.87027	-0.08067	-0.14785
H	-1.4171	4.538	0.83458
H	-4.97128	3.42817	-0.33762
H	-4.38857	3.25789	-2.0009
H	-2.7525	1.53769	-1.26843
H	-1.16643	3.26228	2.81847
H	-2.52278	2.20669	3.22155
H	-0.35127	1.21526	3.65893
H	-1.61233	-0.32352	1.35626
H	-2.32521	-0.24653	2.95494
H	-0.44369	-1.37294	3.98007
H	0.55641	-1.19166	2.55164
H	-2.03815	-2.75041	2.30082
H	-0.86715	-4.86387	3.041
H	-1.03214	-3.69546	4.36953
H	0.52745	-3.9069	3.54516
H	-0.0503	-3.25826	-1.51822
H	-0.55673	-1.33838	-3.10011
H	-1.19666	-0.43114	-1.74746
H	-5.36412	0.74045	0.06506
H	-5.28646	0.87914	-1.68772
H	-3.21351	-0.79293	-0.23089
H	-4.47658	-1.43897	-2.91739

H	-3.30679	-0.13024	-2.83185
H	-3.63249	-3.73123	-2.95303
H	-1.8239	-4.04379	-2.7713
H	-5.33062	-1.67135	0.65262
H	-6.0091	-1.79264	-0.98047
H	-4.6485	-2.82645	-0.50617
H	0.37123	0.78442	1.00457
H	2.72207	-2.8295	-2.09046
H	2.5511	1.72797	0.57805
H	5.43231	-0.41483	-0.61086
H	5.50613	3.38584	0.00846
H	4.1334	3.25629	1.13365
H	5.70862	2.51023	1.51287
H	6.59079	0.53413	-2.63018
H	4.84889	0.8509	-2.67284
H	5.97843	2.14742	-2.21689
H	7.50732	-1.08317	0.12979
H	8.23389	-0.11875	-1.18556
H	8.70749	0.18501	0.50315

Compound 1c: Isomer 2S, 5R, 11S, 13S, 17S, 23R conformer 51

C	0.88478	2.30319	-1.28625
C	0.57817	3.6043	-1.51549
C	1.27801	4.68533	-0.82898
C	2.43293	4.22458	0.06584
C	2.16282	2.85374	0.6806
O	1.76067	1.90406	-0.3394
C	0.27753	1.11825	-1.99015
C	1.31326	0.04176	-2.37373
C	0.65626	-1.2903	-2.76197
C	1.64792	-2.4606	-2.87636
C	2.4466	-2.82041	-1.59126
C	1.5077	-3.12195	-0.43003
C	3.37149	-4.01746	-1.84759
O	1.17686	-1.99333	0.24718
O	1.0811	-4.22205	-0.14325
C	0.19917	-2.12907	1.32838
C	0.32551	-0.8637	2.18021

C	-1.18544	-2.30662	0.77173
C	1.56538	-0.74479	3.05999
C	3.37588	2.24255	1.38541
C	3.21274	0.77382	1.83196
C	2.2454	0.60987	3.03862
C	1.99916	-1.74403	3.83518
C	4.58088	0.15462	2.15048
O	1.02487	5.87213	-0.99036
O	2.21963	0.50308	-3.37675
C	-1.84806	-3.46653	0.51825
O	-3.08737	-3.15446	0.04489
C	-3.13114	-1.78249	0.02899
N	-2.01421	-1.23852	0.44028
C	-4.39158	-1.21944	-0.41727
C	-4.8175	0.05978	-0.34742
C	-3.99031	1.2035	0.22718
C	-6.18553	0.42403	-0.86469
C	-3.43267	2.11	-0.87325
O	-4.78883	2.03491	1.07266
C	-5.06317	1.46029	2.33658
H	-0.16142	3.86668	-2.26379
H	3.33973	4.18055	-0.55482
H	2.6039	4.97301	0.84544
H	1.30735	2.93335	1.36809
H	-0.44888	0.65992	-1.30403
H	-0.27909	1.45853	-2.87241
H	1.94182	-0.11848	-1.49825
H	0.14437	-1.16845	-3.72938
H	-0.12652	-1.52821	-2.0335
H	2.3849	-2.22596	-3.6535
H	1.10728	-3.35677	-3.20734
H	3.051	-1.95284	-1.30637
H	3.99115	-4.23674	-0.97173
H	4.03623	-3.80589	-2.69176
H	2.78862	-4.91457	-2.07622
H	0.46668	-3.02563	1.89104
H	-0.56591	-0.80419	2.81861
H	0.25862	-0.00683	1.50295
H	4.22637	2.30566	0.69365

H	3.62586	2.87788	2.24708
H	2.79024	0.2224	0.98546
H	2.80469	0.7697	3.96936
H	1.47156	1.3891	3.00438
H	2.85432	-1.61929	4.49421
H	1.52154	-2.72098	3.85415
H	5.23595	0.16782	1.27141
H	5.08824	0.70455	2.95438
H	4.47228	-0.88673	2.47215
H	1.70491	0.66947	-4.18361
H	-1.58942	-4.51028	0.58612
H	-5.05857	-1.9675	-0.83981
H	-3.15376	0.79057	0.8033
H	-6.72112	-0.45281	-1.23953
H	-6.78519	0.89871	-0.08077
H	-6.11547	1.1559	-1.6799
H	-2.80239	2.88645	-0.42935
H	-2.83222	1.52854	-1.57931
H	-4.24301	2.60101	-1.42167
H	-5.65018	0.53251	2.2564
H	-4.13633	1.23451	2.88657
H	-5.64128	2.19695	2.90081

Compound 1c: Isomer 2S, 5R, 11S, 13S, 17S, 23R conformer 52

C	2.93583	-2.10803	0.57774
C	3.69035	-3.22144	0.74947
C	5.06582	-3.29301	0.26447
C	5.57263	-1.9995	-0.37943
C	4.43939	-1.26006	-1.08581
O	3.32954	-1.05341	-0.17247
C	1.5835	-1.86292	1.18476
C	1.57044	-0.61116	2.08951
C	0.15135	-0.22927	2.52907
C	0.09521	1.05057	3.38059
C	0.63947	2.34773	2.72199
C	-0.17821	2.7212	1.49333
C	0.6346	3.50775	3.72627
O	0.26178	2.07553	0.38112

O	-1.11928	3.48751	1.48936
C	-0.48538	2.32474	-0.84833
C	0.40527	1.92287	-2.03581
C	-1.77146	1.55204	-0.83046
C	1.78631	2.55738	-2.03072
C	4.85028	0.09636	-1.65925
C	3.76249	0.92733	-2.39081
C	2.90727	1.7579	-1.3908
C	1.99441	3.76154	-2.57471
C	2.93003	0.07081	-3.35676
O	5.78841	-4.27067	0.40738
O	2.45694	-0.75725	3.1993
C	-3.03523	2.00561	-0.62729
O	-3.88787	0.93835	-0.70454
C	-3.08007	-0.14391	-0.94099
N	-1.81495	0.17756	-1.02748
C	-3.62519	-1.47589	-1.10675
C	-4.88178	-1.93303	-0.91989
C	-6.05795	-1.07593	-0.45865
C	-5.20048	-3.37766	-1.21387
C	-6.8842	-0.56548	-1.64258
O	-6.94593	-1.82538	0.37091
C	-6.47031	-2.00455	1.69148
H	3.30806	-4.05523	1.32761
H	5.99728	-1.36593	0.41281
H	6.37818	-2.23947	-1.07978
H	4.04981	-1.90496	-1.88657
H	0.85409	-1.7117	0.37748
H	1.26975	-2.74599	1.75523
H	1.99656	0.21224	1.51336
H	-0.27481	-1.05648	3.11857
H	-0.47772	-0.13127	1.63735
H	0.68248	0.88897	4.2917
H	-0.94118	1.22957	3.69552
H	1.66681	2.16192	2.39169
H	1.05468	4.41753	3.28496
H	1.23253	3.24663	4.60579
H	-0.38497	3.73702	4.05004
H	-0.70453	3.3946	-0.88002

H	-0.14575	2.21208	-2.93919
H	0.48025	0.83184	-2.04807
H	5.29145	0.69836	-0.8535
H	5.66287	-0.11431	-2.36793
H	4.30536	1.65958	-3.00594
H	2.48771	1.08922	-0.63787
H	3.58783	2.44007	-0.86481
H	2.97238	4.23745	-2.55342
H	1.19792	4.32139	-3.06068
H	3.579	-0.50225	-4.03076
H	2.28764	-0.6405	-2.82513
H	2.28723	0.70174	-3.97866
H	2.13483	-1.49721	3.74027
H	-3.46973	2.96731	-0.40847
H	-2.86166	-2.1728	-1.44265
H	-5.68705	-0.21328	0.10753
H	-4.32518	-3.90983	-1.59688
H	-5.56444	-3.8968	-0.32073
H	-6.00378	-3.45651	-1.95753
H	-7.73303	0.01647	-1.27046
H	-6.27186	0.07085	-2.28819
H	-7.27483	-1.39834	-2.23603
H	-5.5297	-2.5757	1.72462
H	-6.30433	-1.03891	2.19422
H	-7.24017	-2.55989	2.23372

Compound 1c: Isomer 2S, 5R, 11S, 13S, 17S, 23R conformer 55

C	-2.38803	2.32716	1.43987
C	-2.26244	3.51798	0.79827
C	-3.14674	3.89738	-0.30066
C	-4.21397	2.85061	-0.63616
C	-3.66445	1.45262	-0.3863
O	-3.2	1.3383	0.9905
C	-1.71087	1.99194	2.74123
C	-0.62427	0.87896	2.7374
C	-1.17024	-0.51239	2.39507
C	-0.16866	-1.65577	2.61764
C	-0.60152	-3.01073	1.97652

C	-0.1885	-3.05378	0.50902
C	-0.01398	-4.2145	2.72053
O	-0.83897	-2.10109	-0.21746
O	0.63713	-3.79672	0.0277
C	-0.31127	-1.77686	-1.53041
C	-1.24005	-0.70002	-2.12147
C	1.07819	-1.22346	-1.38644
C	-2.62418	-1.10895	-2.59712
C	-4.64986	0.31007	-0.62048
C	-4.06296	-1.10713	-0.3868
C	-3.59169	-1.82912	-1.67755
C	-2.98513	-0.81458	-3.8535
C	-5.08676	-2.00167	0.3318
O	-3.09733	4.97824	-0.87348
O	0.47366	1.2514	1.91668
C	2.22023	-1.54377	-2.04267
O	3.21665	-0.71917	-1.58777
C	2.62858	0.07152	-0.64275
N	1.35006	-0.18881	-0.50213
C	3.3625	1.07658	0.09686
C	4.67628	1.38989	0.06642
C	5.71727	0.7144	-0.82254
C	5.18576	2.51667	0.92935
C	5.94916	1.5027	-2.11526
O	6.97952	0.62686	-0.16205
C	7.03934	-0.40258	0.80765
H	-1.56568	4.26056	1.16955
H	-5.09675	3.03223	-0.00568
H	-4.5197	2.97038	-1.67985
H	-2.77652	1.32519	-1.01711
H	-1.2412	2.90585	3.11628
H	-2.49105	1.70603	3.46148
H	-0.23674	0.8588	3.76743
H	-1.48956	-0.50638	1.35227
H	-2.07415	-0.70024	2.99043
H	-0.04187	-1.82183	3.6955
H	0.8175	-1.36669	2.23589
H	-1.699	-3.06456	2.00903
H	-0.30159	-5.15749	2.24614

H	-0.36828	-4.22428	3.75677
H	1.07971	-4.17117	2.72904
H	-0.29078	-2.68251	-2.14399
H	-0.70732	-0.25281	-2.96748
H	-1.30216	0.08404	-1.35802
H	-5.50385	0.47111	0.04995
H	-5.03446	0.40372	-1.64439
H	-3.20079	-1.00758	0.27672
H	-3.14085	-2.77984	-1.3612
H	-4.48025	-2.09162	-2.26739
H	-3.97078	-1.06541	-4.23783
H	-2.31193	-0.3144	-4.54582
H	-5.36516	-1.57589	1.30253
H	-6.0046	-2.11074	-0.26082
H	-4.68502	-3.00689	0.50764
H	0.44324	0.77652	1.0615
H	2.4951	-2.27475	-2.78566
H	2.70765	1.6279	0.76677
H	5.3837	-0.29653	-1.08368
H	4.3715	2.99634	1.47876
H	5.93355	2.16228	1.64714
H	5.68874	3.27815	0.31983
H	6.72022	1.00511	-2.71154
H	5.02656	1.55802	-2.70091
H	6.28875	2.52036	-1.89799
H	6.32026	-0.24945	1.62671
H	6.84531	-1.3895	0.35923
H	8.05194	-0.392	1.21945

Compound 1c: Isomer 2S, 5R, 11S, 13S, 17S, 23R conformer 57

C	-0.22278	2.36953	1.80271
C	0.83157	2.92592	1.14991
C	0.65048	3.72055	-0.05757
C	-0.81563	3.99409	-0.40238
C	-1.64736	2.75494	-0.08516
O	-1.48462	2.38179	1.31924
C	-0.09865	1.64495	3.11282
C	0.10372	0.10993	2.97263

C	-1.12306	-0.62063	2.41289
C	-1.00242	-2.14629	2.52351
C	-2.10076	-2.94211	1.7614
C	-1.74193	-3.01653	0.28387
C	-2.27582	-4.35413	2.33079
O	-1.93684	-1.82557	-0.33722
O	-1.29973	-3.99577	-0.28031
C	-1.46176	-1.72882	-1.71156
C	-2.15834	-0.52668	-2.35714
C	0.02655	-1.51911	-1.69564
C	-3.66085	-0.37525	-2.18393
C	-3.15436	2.87731	-0.31771
C	-3.87533	1.50529	-0.42999
C	-4.09432	1.05223	-1.91623
C	-4.52643	-1.38773	-2.28261
C	-5.21294	1.50204	0.32399
O	1.57805	4.16236	-0.72835
O	1.27961	-0.18606	2.22351
C	1.04003	-2.37489	-1.98234
O	2.22418	-1.7071	-1.80653
C	1.86839	-0.45048	-1.39647
N	0.57093	-0.29643	-1.31979
C	2.84123	0.59032	-1.11919
C	4.15226	0.50021	-0.81216
C	4.92279	-0.80808	-0.66772
C	4.93166	1.76555	-0.55332
C	5.96643	-0.9948	-1.77076
O	5.62604	-0.83516	0.57567
C	4.78892	-1.09041	1.69368
H	1.82919	2.82971	1.5626
H	-1.16838	4.8566	0.18166
H	-0.89404	4.25071	-1.46287
H	-1.22998	1.91579	-0.65795
H	0.7643	2.04685	3.65164
H	-0.99649	1.83689	3.71235
H	0.31853	-0.26698	3.98053
H	-1.25835	-0.33038	1.36692
H	-2.02266	-0.28329	2.94587
H	-1.05764	-2.43342	3.58242

H	-0.01219	-2.46119	2.17221
H	-3.04617	-2.38981	1.84439
H	-3.03423	-4.91693	1.77764
H	-2.58259	-4.30104	3.38088
H	-1.33884	-4.91587	2.26941
H	-1.70169	-2.6688	-2.21254
H	-1.91566	-0.55361	-3.42959
H	-1.66502	0.36967	-1.97584
H	-3.56379	3.46215	0.5154
H	-3.32536	3.4715	-1.22417
H	-3.23469	0.76081	0.05787
H	-5.15177	1.17927	-2.17797
H	-3.53206	1.7149	-2.58911
H	-5.59805	-1.22867	-2.19123
H	-4.20683	-2.41254	-2.45686
H	-5.06697	1.71224	1.38992
H	-5.89688	2.2606	-0.07869
H	-5.70589	0.52677	0.23653
H	1.11941	0.08434	1.30191
H	1.10047	-3.40733	-2.28593
H	2.38637	1.57742	-1.14664
H	4.22469	-1.65277	-0.69945
H	4.27744	2.64195	-0.5402
H	5.46836	1.69591	0.39861
H	5.69378	1.92147	-1.32882
H	6.4825	-1.94902	-1.62683
H	5.4885	-0.99518	-2.75585
H	6.71382	-0.19605	-1.74195
H	4.00479	-0.33156	1.82123
H	4.30034	-2.07371	1.61006
H	5.43587	-1.08968	2.57502

Compound 1c: Isomer 2S, 5R, 11S, 13S, 17S, 23R conformer 58

C	2.79859	-2.23324	0.91023
C	3.14633	-3.26152	0.09367
C	4.13078	-3.09789	-0.97071
C	4.74003	-1.69588	-1.04883
C	3.72344	-0.64426	-0.62027

O	3.19997	-0.95959	0.70265
C	1.98109	-2.39599	2.16255
C	0.63595	-1.62515	2.20157
C	0.77679	-0.10744	2.36105
C	-0.53417	0.58535	2.75638
C	-0.44084	2.14219	2.77002
C	-0.99015	2.74242	1.47893
C	-1.15183	2.76415	3.97726
O	-0.39399	2.20013	0.38692
O	-1.85564	3.58837	1.40988
C	-0.93599	2.56808	-0.90953
C	0.11223	2.13119	-1.94308
C	-2.26209	1.90058	-1.13474
C	1.38381	2.97309	-1.999
C	4.26439	0.78089	-0.53723
C	3.17052	1.87248	-0.4916
C	2.6997	2.22589	-1.92866
C	1.33908	4.29954	-2.16546
C	3.66423	3.12081	0.25018
O	4.49696	-4.00486	-1.70729
O	-0.10997	-1.84153	1.01001
C	-3.43911	2.44927	-1.53584
O	-4.35297	1.44297	-1.67363
C	-3.67714	0.3022	-1.32285
N	-2.42991	0.52998	-1.00941
C	-4.44725	-0.93105	-1.38875
C	-4.18004	-2.11315	-0.79436
C	-3.01097	-2.31232	0.16052
C	-5.08498	-3.29768	-1.0174
C	-3.4886	-2.58487	1.58864
O	-2.19658	-3.4425	-0.20941
C	-1.52088	-3.26842	-1.45377
H	2.76655	-4.25895	0.28395
H	5.62153	-1.66072	-0.39177
H	5.08327	-1.5104	-2.07111
H	2.86111	-0.69412	-1.30246
H	1.7803	-3.46558	2.2896
H	2.59085	-2.08445	3.02264
H	0.08431	-2.01513	3.07425

H	1.13476	0.29583	1.41199
H	1.54691	0.10846	3.11421
H	-0.82731	0.25368	3.76184
H	-1.32866	0.26327	2.07523
H	0.62354	2.41781	2.80872
H	-1.08585	3.8555	3.96063
H	-0.70037	2.40007	4.90656
H	-2.21433	2.49907	3.98344
H	-1.07946	3.65144	-0.9261
H	-0.37038	2.1549	-2.92875
H	0.34558	1.07983	-1.74421
H	4.89802	0.83666	0.35693
H	4.92441	0.95814	-1.39743
H	2.31277	1.47349	0.0627
H	3.4894	2.81738	-2.41122
H	2.60639	1.29927	-2.51375
H	2.24552	4.8932	-2.25022
H	0.40191	4.84822	-2.23023
H	3.91618	2.88379	1.29049
H	4.56451	3.5333	-0.22516
H	2.89909	3.90355	0.25492
H	-0.52251	-2.72292	0.99718
H	-3.7819	3.45096	-1.73984
H	-5.353	-0.83787	-1.98457
H	-2.38143	-1.4217	0.15372
H	-5.61803	-3.57122	-0.09738
H	-5.83031	-3.09781	-1.79262
H	-4.4995	-4.17754	-1.30835
H	-2.62645	-2.62667	2.26174
H	-4.15178	-1.78394	1.93146
H	-4.02173	-3.53828	1.65606
H	-2.22845	-3.18632	-2.29017
H	-0.88394	-2.37578	-1.43558
H	-0.90173	-4.15777	-1.59793

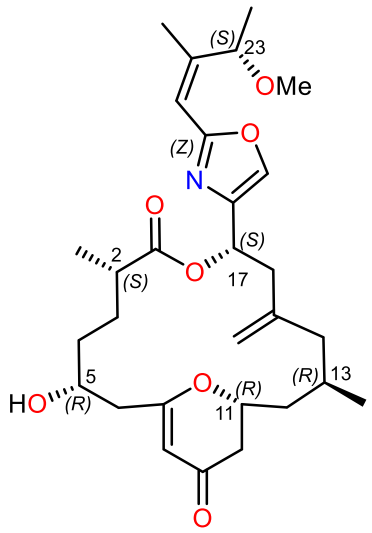

Table 6. Calculated DFT energies of the **1d**-(2S5R11R13R17S23S*)* diasteroisomer.

Conformers 1d	OPLS2008 Force Field	B3LYP/6-31+G(d,p)	Δ (DFT Energy)	% Population
**1d4**	0.675979924	-1710.462343	0	60.29
**1d25**	2.917853728	-1710.461345	0.62625437	20.92
**1d15**	2.413838432	-1710.460657	1.05798083	10.09
**1d1**	0	-1710.459641	1.69553037	3.44
**1d22**	2.878847992	-1710.458274	2.55333571	0.81
**1d28**	3.050454111	-1710.458268	2.55710077	0.80
**1d45**	3.916849904	-1710.457978	2.73907849	0.59
**1d20**	2.781477055	-1710.457663	2.93674395	0.42
**1d6**	1.353608987	-1710.457538	3.01518262	0.37
**1d2**	0.363957935	-1710.457466	3.0603633	0.34
**1d17**	2.638432122	-1710.457395	3.10491647	0.32
**1d3**	0.483843212	-1710.457216	3.21724065	0.26
**1d33**	3.420458891	-1710.457205	3.22414325	0.26
**1d39**	3.690774379	-1710.456651	3.57178345	0.14
**1d55**	4.390869981	-1710.456577	3.61821915	0.13
**1d57**	4.408819312	-1710.456514	3.65775224	0.12
**1d11**	1.783699809	-1710.456114	3.908756	0.08
1d49	4.078967495	-1710.456082	3.9288363	0.08
1d16	2.592638623	-1710.455898	4.04429803	0.06

19 conformers counting for the 99.53% of the DFT conformational population

DFT COORDINATES FOR CONFORMATIONAL SEARCH OF 1d

(2S*, 5R*, 11R*, 13R* , 17S*, 23S*)

Compound 1c: Isomer 2S, 5R, 11R, 13R, 17S, 23S conformer 1

C	-2.71147	1.51437	1.11078
C	-3.87926	1.81271	1.74062
C	-5.11957	2.03067	0.99687
C	-4.96889	1.91817	-0.52103
C	-3.86493	0.93351	-0.89822
O	-2.62631	1.27878	-0.21907
C	-1.40309	1.27155	1.80327
C	-1.27705	-0.23433	2.15522
C	0.1226	-0.60754	2.64153
C	0.2402	-2.06575	3.12184
C	-0.22323	-3.17713	2.13989
C	0.66763	-3.23929	0.90815
C	-0.24422	-4.54213	2.83963
O	0.36161	-2.26218	0.01409
O	1.55844	-4.04449	0.7305
C	1.2047	-2.1897	-1.17644
C	0.43197	-1.37922	-2.22272
C	2.53001	-1.56851	-0.84402
C	-0.89199	-1.99883	-2.65611
C	-3.58582	0.87317	-2.39831
C	-2.29788	0.14968	-2.84951
C	-2.16402	-1.27695	-2.25857
C	-0.91962	-3.13107	-3.36697
C	-2.23933	0.14124	-4.38448
O	-6.18391	2.32261	1.52393
O	-2.20007	-0.59314	3.17997
C	3.72069	-2.18455	-0.62017
O	4.65744	-1.22484	-0.37365
C	3.97807	-0.03484	-0.45063
N	2.7106	-0.19406	-0.73369
C	4.781	1.15239	-0.23629
C	4.36052	2.43009	-0.12158
C	2.89781	2.84939	-0.20403
C	5.36366	3.54039	0.05661
C	2.51458	3.27479	-1.62377

O	2.63045	3.96259	0.6508
C	2.52119	3.61313	2.01688
H	-3.88785	1.98083	2.81235
H	-5.92679	1.6174	-0.9563
H	-4.72657	2.91591	-0.91546
H	-4.13753	-0.06391	-0.52254
H	-1.33681	1.85845	2.72478
H	-0.58568	1.56277	1.13405
H	-1.48768	-0.80339	1.23832
H	0.39337	0.04806	3.48056
H	0.82919	-0.4038	1.82977
H	-0.36657	-2.17667	4.0262
H	1.2811	-2.26401	3.4103
H	-1.23671	-2.93111	1.80468
H	-0.58531	-5.33146	2.16213
H	-0.91996	-4.51134	3.70063
H	0.75553	-4.81731	3.18904
H	1.36612	-3.21413	-1.51906
H	1.09572	-1.27224	-3.08934
H	0.2882	-0.37142	-1.82417
H	-3.5523	1.89927	-2.78874
H	-4.45973	0.3954	-2.86416
H	-1.45474	0.7456	-2.47957
H	-2.18847	-1.20523	-1.16318
H	-3.03142	-1.87831	-2.56384
H	-1.85515	-3.58845	-3.67894
H	-0.00982	-3.65062	-3.66143
H	-2.31714	1.15812	-4.787
H	-3.06388	-0.44883	-4.8059
H	-1.30469	-0.29632	-4.74784
H	-3.09162	-0.36524	2.867
H	4.0412	-3.21258	-0.57941
H	5.84617	0.94275	-0.1749
H	2.26173	2.00352	0.0856
H	6.38879	3.15936	0.03774
H	5.25977	4.29099	-0.7374
H	5.20521	4.07509	0.99935
H	1.47122	3.60546	-1.63426
H	2.6273	2.43179	-2.31128

H	3.13959	4.10465	-1.96996
H	1.71333	2.88301	2.18315
H	3.45372	3.18785	2.41881
H	2.28786	4.53121	2.56291

Compound 1c: Isomer 2S, 5R, 11R, 13R, 17S, 23S conformer 2

C	-2.77561	1.42462	1.15819
C	-3.93767	1.77674	1.75988
C	-5.15212	2.03561	0.99411
C	-4.98024	1.9087	-0.52047
C	-3.89594	0.89611	-0.88254
O	-2.66285	1.19852	-0.17467
C	-1.48065	1.16043	1.87004
C	-1.29463	-0.35072	2.15224
C	0.12504	-0.6861	2.63014
C	0.2963	-2.14715	3.08535
C	-0.12728	-3.25797	2.08589
C	0.76109	-3.26425	0.85079
C	-0.09337	-4.63454	2.76168
O	0.42594	-2.27146	-0.01337
O	1.67013	-4.04265	0.64803
C	1.25371	-2.15029	-1.21129
C	0.44584	-1.33826	-2.22819
C	2.5656	-1.50226	-0.87742
C	-0.86799	-1.98371	-2.65639
C	-3.59481	0.85425	-2.38077
C	-2.29713	0.14872	-2.8302
C	-2.15167	-1.28337	-2.25745
C	-0.87667	-3.11682	-3.36628
C	-2.22509	0.16114	-4.36468
O	-6.2153	2.37482	1.49714
O	-2.29369	-0.83059	3.05155
C	3.77356	-2.0923	-0.6772
O	4.68762	-1.1146	-0.4165
C	3.97741	0.05902	-0.46127
N	2.712	-0.12646	-0.7373
C	4.752	1.26111	-0.22632
C	4.30209	2.52652	-0.08896

C	2.83028	2.91388	-0.16546
C	5.27946	3.65632	0.10875
C	2.43887	3.35796	-1.5772
O	2.5374	4.00432	0.71056
C	2.43074	3.62509	2.06826
H	-3.97416	1.90869	2.83507
H	-5.93911	1.62943	-0.96808
H	-4.70956	2.89864	-0.91655
H	-4.20386	-0.09748	-0.52587
H	-1.44936	1.7155	2.81586
H	-0.65251	1.5154	1.24448
H	-1.48429	-0.89103	1.22104
H	0.38549	-0.03405	3.47908
H	0.82748	-0.44231	1.8255
H	-0.30588	-2.30222	3.98757
H	1.3427	-2.31205	3.37441
H	-1.15036	-3.04448	1.75844
H	-0.40166	-5.42388	2.06902
H	-0.7717	-4.64785	3.62118
H	0.91629	-4.87611	3.10774
H	1.43852	-3.16245	-1.57754
H	1.09374	-1.19305	-3.10135
H	0.28071	-0.34421	-1.8044
H	-3.56291	1.88519	-2.75862
H	-4.46003	0.37785	-2.86402
H	-1.4638	0.74886	-2.44508
H	-2.18712	-1.2243	-1.16178
H	-3.0091	-1.89151	-2.5767
H	-1.80474	-3.59039	-3.67625
H	0.04126	-3.62106	-3.66231
H	-2.31123	1.18227	-4.75459
H	-3.03934	-0.43255	-4.80073
H	-1.28258	-0.26112	-4.7262
H	-2.15261	-0.39971	3.91082
H	4.12072	-3.11238	-0.66297
H	5.82194	1.07562	-0.17021
H	2.21291	2.04876	0.10715
H	6.31314	3.29965	0.08297
H	5.15776	4.41824	-0.67181

H	5.1094	4.17069	1.06079
H	1.3879	3.66354	-1.58311
H	2.57265	2.53172	-2.28109
H	3.04436	4.20914	-1.90586
H	1.63609	2.87641	2.21647
H	3.36986	3.20897	2.46472
H	2.1783	4.52705	2.63244

Compound 1c: Isomer 2S, 5R, 11R, 13R, 17S, 23S conformer 3

C	2.92859	-2.11991	-0.24281
C	4.04635	-2.58196	0.37437
C	5.34307	-1.93861	0.20345
C	5.33109	-0.76452	-0.77584
C	3.99001	-0.03404	-0.75608
O	2.89042	-0.96575	-0.95233
C	1.58976	-2.80096	-0.17745
C	0.82328	-2.53187	1.14632
C	0.51674	-1.04217	1.36047
C	-0.10525	-0.74847	2.73487
C	0.03128	0.7141	3.24003
C	-0.82771	1.70078	2.45636
C	-0.2989	0.81673	4.73418
O	-0.39512	1.83609	1.17499
O	-1.77478	2.31304	2.9008
C	-1.14398	2.74279	0.3216
C	-0.29182	2.96405	-0.93959
C	-2.48697	2.16774	-0.01785
C	1.08471	3.55568	-0.67548
C	3.88316	1.03983	-1.8347
C	2.56543	1.83871	-1.9238
C	2.27064	2.61351	-0.61178
C	1.23051	4.87445	-0.5055
C	2.63276	2.7739	-3.14081
O	6.37458	-2.3246	0.73803
O	-0.32533	-3.36228	1.23202
C	-3.72762	2.69135	0.16237
O	-4.63993	1.8093	-0.34295
C	-3.89533	0.75543	-0.80368

N	-2.61031	0.93899	-0.64807
C	-4.64475	-0.3604	-1.3529
C	-4.19955	-1.61464	-1.57946
C	-2.77754	-2.05361	-1.26273
C	-5.11686	-2.65583	-2.16834
C	-1.98064	-2.37685	-2.52668
O	-2.75618	-3.24254	-0.44911
C	-3.408	-3.09594	0.81145
H	4.00199	-3.49719	0.955
H	6.14708	-0.07982	-0.52585
H	5.52803	-1.15797	-1.78399
H	3.84066	0.39942	0.24372
H	1.71882	-3.88434	-0.26357
H	0.98394	-2.46035	-1.02594
H	1.46457	-2.87067	1.97015
H	-0.12697	-0.67229	0.55366
H	1.45881	-0.48657	1.27116
H	0.39317	-1.37819	3.48416
H	-1.16017	-1.04788	2.75138
H	1.07547	1.01859	3.07934
H	-0.19504	1.84383	5.09582
H	0.37356	0.17463	5.31278
H	-1.32945	0.50313	4.9279
H	-1.27855	3.68329	0.86351
H	-0.86652	3.63703	-1.58679
H	-0.22059	2.00662	-1.46547
H	4.06521	0.55639	-2.80406
H	4.71712	1.7392	-1.67765
H	1.75546	1.1166	-2.08878
H	2.09558	1.88749	0.19255
H	3.16306	3.19224	-0.33596
H	2.19962	5.32249	-0.30069
H	0.38597	5.55872	-0.56048
H	2.84636	2.21015	-4.05652
H	3.42453	3.52454	-3.01749
H	1.6929	3.31425	-3.28861
H	-0.90353	-3.23165	0.45576
H	-4.11505	3.59224	0.61013
H	-5.68309	-0.12164	-1.5718

H	-2.27273	-1.25066	-0.71836
H	-6.15052	-2.30147	-2.21688
H	-4.80456	-2.92164	-3.18715
H	-5.08629	-3.58247	-1.585
H	-0.96503	-2.68708	-2.26123
H	-1.91593	-1.49207	-3.16746
H	-2.44373	-3.19189	-3.09123
H	-3.18325	-3.99922	1.38172
H	-3.02813	-2.22444	1.36106
H	-4.49478	-2.99573	0.69614

Compound 1c: Isomer 2S, 5R, 11R, 13R, 17S, 23S conformer 4

C	5.12942	-0.56206	-0.50222
C	5.87754	-0.69138	0.62025
C	6.13656	0.43786	1.50997
C	5.53812	1.76063	1.0314
C	4.23314	1.53783	0.27391
O	4.43104	0.56947	-0.80368
C	4.9915	-1.61406	-1.56735
C	3.54152	-2.07127	-1.86214
C	2.8074	-2.5997	-0.61929
C	1.47092	-3.26557	-0.97667
C	0.55606	-3.54307	0.23621
C	0.22322	-2.24143	0.95275
C	-0.72624	-4.28512	-0.18164
O	-0.3953	-1.37297	0.11833
O	0.49917	-1.98592	2.10778
C	-0.62079	-0.02016	0.62178
C	-0.48965	0.90697	-0.59754
C	-1.94661	0.05158	1.31635
C	-0.22253	2.3494	-0.2103
C	3.67206	2.8076	-0.36221
C	2.2361	2.71692	-0.92315
C	1.18915	2.6957	0.22278
C	-1.19526	3.26663	-0.23941
C	1.9727	3.87633	-1.89395
O	6.82445	0.36454	2.51828
O	2.79948	-1.04344	-2.51515

C	-2.20468	-0.11253	2.64108
O	-3.55043	0.00137	2.8297
C	-4.0668	0.22932	1.5781
N	-3.14523	0.27367	0.65134
C	-5.50712	0.38284	1.52141
C	-6.29254	0.46697	0.42663
C	-5.76285	0.42789	-1.0021
C	-7.78148	0.64328	0.579
C	-5.60969	1.83642	-1.58192
O	-6.65923	-0.27818	-1.8628
C	-6.58331	-1.68406	-1.72215
H	6.37796	-1.62969	0.831
H	5.37306	2.41208	1.89463
H	6.27169	2.25646	0.37891
H	3.50385	1.07799	0.9562
H	5.60176	-2.47677	-1.28219
H	5.40349	-1.21441	-2.50392
H	3.61113	-2.88257	-2.59833
H	2.64474	-1.75946	0.0699
H	3.45174	-3.31087	-0.08344
H	1.66625	-4.21961	-1.48547
H	0.93546	-2.6281	-1.68738
H	1.09659	-4.14563	0.97509
H	-1.36823	-4.48913	0.68232
H	-0.47082	-5.24397	-0.64617
H	-1.30028	-3.69562	-0.90322
H	0.16458	0.17551	1.35549
H	-1.40115	0.83078	-1.19584
H	0.33424	0.51133	-1.20371
H	4.36347	3.10377	-1.16182
H	3.71127	3.60586	0.39203
H	2.15091	1.78083	-1.49159
H	1.51484	1.97636	0.98837
H	1.19168	3.68047	0.70863
H	-1.0165	4.2988	0.053
H	-2.20619	3.01302	-0.54676
H	2.65989	3.84013	-2.74698
H	2.11151	4.8436	-1.39291
H	0.94907	3.845	-2.27906

H	2.99819	-0.21867	-2.0405
H	-1.59716	-0.3177	3.50758
H	-5.97027	0.42929	2.50419
H	-4.78137	-0.06091	-1.01304
H	-8.07079	0.71751	1.63129
H	-8.12302	1.54962	0.06259
H	-8.32822	-0.18864	0.12187
H	-5.24865	1.76871	-2.61284
H	-4.89188	2.41269	-0.99051
H	-6.56814	2.36597	-1.58924
H	-7.28153	-2.11423	-2.44538
H	-5.56971	-2.05651	-1.93694
H	-6.86562	-2.02039	-0.7124

Compound 1c: Isomer 2S, 5R, 11R, 13R, 17S, 23S conformer 6

C	-2.76752	1.24087	1.345
C	-3.97585	1.41785	1.94325
C	-5.16213	1.80475	1.18064
C	-4.90834	2.01275	-0.31314
C	-3.77723	1.11919	-0.81665
O	-2.58951	1.29493	0.00415
C	-1.51294	0.82945	2.05682
C	-1.42198	-0.71952	2.08329
C	-0.06243	-1.21878	2.5706
C	0.00962	-2.7489	2.7273
C	-0.40095	-3.60775	1.49928
C	0.57198	-3.42217	0.34465
C	-0.48439	-5.09121	1.8805
O	0.34443	-2.26448	-0.32892
O	1.46396	-4.19348	0.0547
C	1.28153	-1.95259	-1.40856
C	0.59956	-0.91784	-2.30906
C	2.58018	-1.45169	-0.84419
C	-0.68725	-1.40049	-2.96859
C	-3.3959	1.38289	-2.27196
C	-2.07917	0.75838	-2.78368
C	-1.9859	-0.76486	-2.51328
C	-0.66091	-2.34266	-3.91702

C	-1.91595	1.08617	-4.27537
O	-6.26035	1.99344	1.68472
O	-2.4171	-1.26965	2.94203
C	3.70628	-2.15824	-0.55935
O	4.63883	-1.293	-0.06813
C	4.02436	-0.06595	-0.06911
N	2.79919	-0.11525	-0.5267
C	4.83543	1.0333	0.41134
C	4.47281	2.3217	0.58623
C	3.08788	2.84814	0.25423
C	5.47286	3.32614	1.09311
C	3.00182	3.2659	-1.22386
O	2.83421	3.95257	1.12756
C	1.46752	4.26513	1.28684
H	-4.05958	1.35271	3.02293
H	-5.83333	1.82046	-0.86533
H	-4.64471	3.06885	-0.47097
H	-4.07177	0.06744	-0.68445
H	-1.50834	1.20311	3.08546
H	-0.64709	1.23824	1.52371
H	-1.57148	-1.07308	1.0532
H	0.1528	-0.76638	3.54802
H	0.70113	-0.85917	1.87228
H	-0.65553	-3.04048	3.54635
H	1.02747	-3.03025	3.02839
H	-1.38669	-3.26811	1.16304
H	-0.79148	-5.70495	1.02761
H	-1.2134	-5.23033	2.68561
H	0.48748	-5.46162	2.22025
H	1.46109	-2.88237	-1.95237
H	1.33183	-0.6391	-3.07649
H	0.428	-0.01695	-1.71415
H	-3.33873	2.46903	-2.42531
H	-4.2353	1.02979	-2.88818
H	-1.26369	1.24604	-2.23551
H	-2.0899	-0.93398	-1.43345
H	-2.82804	-1.27228	-3.00372
H	-1.57002	-2.70188	-4.39262
H	0.26895	-2.79809	-4.25217

H	-1.96726	2.16757	-4.44906
H	-2.70964	0.61476	-4.86986
H	-0.95825	0.72674	-4.66358
H	-3.28338	-0.96515	2.62353
H	3.97707	-3.19929	-0.61961
H	5.85503	0.74146	0.64931
H	2.34712	2.05845	0.43629
H	6.45546	2.86958	1.24315
H	5.58018	4.16311	0.39098
H	5.13143	3.76327	2.03708
H	2.01436	3.68036	-1.45422
H	3.16043	2.39696	-1.8684
H	3.75497	4.0267	-1.45485
H	0.99786	4.61123	0.35416
H	0.89544	3.40085	1.6619
H	1.41151	5.07128	2.02353

Compound 1c: Isomer 2S, 5R, 11R, 13R, 17S, 23S conformer 11

C	-1.8569	1.27538	1.61962
C	-2.51787	2.27858	2.25274
C	-3.49415	3.11173	1.54808
C	-3.66383	2.76289	0.0652
C	-3.47954	1.26309	-0.13941
O	-2.17879	0.85834	0.37185
C	-0.72142	0.4702	2.18295
C	-1.09655	-1.02045	2.34568
C	0.12752	-1.89685	2.60697
C	-0.15958	-3.4077	2.58152
C	-0.77456	-3.98145	1.27453
C	0.09934	-3.67711	0.06358
C	-0.99416	-5.49454	1.39952
O	-0.15351	-2.43997	-0.42899
O	0.92556	-4.43015	-0.41245
C	0.64185	-1.99372	-1.57453
C	-0.11509	-0.82436	-2.20079
C	2.02424	-1.5986	-1.13245
C	-1.42627	-1.12219	-2.90012
C	-3.58249	0.69903	-1.55714

C	-2.54405	1.21352	-2.58306
C	-2.09407	0.07931	-3.54546
C	-1.97084	-2.34057	-2.99359
C	-3.07656	2.39673	-3.40763
O	-4.08866	4.04561	2.0677
O	-1.99627	-1.21243	3.43881
C	3.12322	-2.38716	-0.98545
O	4.15891	-1.60103	-0.57559
C	3.63643	-0.33529	-0.48763
N	2.36902	-0.28995	-0.80995
C	4.57658	0.69331	-0.08965
C	4.35052	2.01416	0.0753
C	2.99981	2.67934	-0.16385
C	5.48535	2.9298	0.4548
C	2.87905	3.18099	-1.6061
O	2.82227	3.81313	0.68531
C	2.44559	3.47896	2.00697
H	-2.25491	2.54747	3.2698
H	-4.65282	3.08965	-0.26973
H	-2.91272	3.32663	-0.50473
H	-4.22149	0.74533	0.48898
H	-0.40692	0.8824	3.14702
H	0.12162	0.54385	1.48291
H	-1.56659	-1.33454	1.40645
H	0.53389	-1.63618	3.59321
H	0.89606	-1.64593	1.86823
H	-0.85422	-3.64973	3.39365
H	0.77362	-3.94766	2.7897
H	-1.73905	-3.48972	1.10644
H	-1.48665	-5.90156	0.51017
H	-1.6231	-5.71294	2.26903
H	-0.03985	-6.01609	1.51813
H	0.71715	-2.83957	-2.26218
H	0.56164	-0.34233	-2.92047
H	-0.27532	-0.07501	-1.41767
H	-4.60154	0.88889	-1.92077
H	-3.48553	-0.38834	-1.45864
H	-1.65996	1.55638	-2.0311
H	-2.96764	-0.26175	-4.1166

H	-1.40035	0.52266	-4.27635
H	-2.90185	-2.49792	-3.53268
H	-1.52276	-3.22398	-2.5503
H	-3.40767	3.22425	-2.77164
H	-3.93369	2.08943	-4.02093
H	-2.30665	2.78552	-4.08455
H	-2.7836	-0.66849	3.27591
H	3.31341	-3.44098	-1.10227
H	5.58279	0.31126	0.06451
H	2.19829	1.95268	0.01755
H	6.43419	2.38839	0.50919
H	5.59095	3.74339	-0.27395
H	5.30328	3.41327	1.42096
H	1.91723	3.68718	-1.7351
H	2.93604	2.34095	-2.30413
H	3.67519	3.89489	-1.84217
H	3.19695	2.85268	2.51259
H	2.34209	4.41872	2.55597
H	1.48203	2.94618	2.02853

Compound 1c: Isomer 2S, 5R, 11R, 13R, 17S, 23S conformer 15

C	-5.36749	0.3516	0.38565
C	-5.74278	0.34868	1.68783
C	-5.66504	-0.85561	2.51097
C	-5.18099	-2.09533	1.75893
C	-4.1776	-1.72628	0.67124
O	-4.739	-0.70219	-0.20893
C	-5.61972	1.47902	-0.57653
C	-4.35939	2.06105	-1.2622
C	-3.30251	2.56299	-0.26496
C	-2.17144	3.33365	-0.96073
C	-0.94562	3.60887	-0.06285
C	-0.34852	2.29757	0.42915
C	0.11216	4.45386	-0.79607
O	0.0269	1.51593	-0.60974
O	-0.24567	1.96616	1.59361
C	0.46021	0.15745	-0.29236
C	0.03704	-0.70587	-1.49125

C	1.93302	0.14244	-0.01048
C	-0.02926	-2.18376	-1.15362
C	-3.77186	-2.90319	-0.21279
C	-2.57069	-2.66974	-1.15538
C	-1.23533	-2.65646	-0.36451
C	0.94512	-3.02187	-1.52336
C	-2.55021	-3.73401	-2.26122
O	-6.01546	-0.90262	3.68156
O	-3.80702	1.13521	-2.19523
C	2.55449	0.31324	1.1865
O	3.90454	0.26971	0.97314
C	4.04282	0.06599	-0.3762
N	2.89489	-0.00903	-0.99791
C	5.34667	-0.02163	-1.00162
C	6.579	-0.05776	-0.45126
C	6.86322	-0.01014	1.04774
C	7.79363	-0.10579	-1.34443
C	7.17458	1.41346	1.51863
O	7.99703	-0.81151	1.38297
C	7.72009	-2.19871	1.4098
H	-6.20083	1.2311	2.11999
H	-4.73396	-2.7949	2.47139
H	-6.05479	-2.59164	1.31181
H	-3.29991	-1.26327	1.14447
H	-6.15376	2.27315	-0.0454
H	-6.28218	1.11177	-1.37207
H	-4.6962	2.91032	-1.87074
H	-2.89787	1.69728	0.27766
H	-3.78191	3.19889	0.49264
H	-2.55719	4.2975	-1.32057
H	-1.84845	2.76958	-1.84138
H	-1.26342	4.13625	0.84381
H	0.97669	4.65554	-0.1541
H	-0.3178	5.41614	-1.09529
H	0.46587	3.94048	-1.69544
H	-0.07147	-0.1364	0.61567
H	0.73508	-0.5299	-2.31353
H	-0.94594	-0.33817	-1.80919
H	-4.65421	-3.18754	-0.80073

H	-3.54978	-3.75572	0.44401
H	-2.70129	-1.69129	-1.63747
H	-1.35411	-2.01664	0.52237
H	-1.04977	-3.67053	0.0139
H	0.91448	-4.07963	-1.27166
H	1.812	-2.67414	-2.07848
H	-3.45858	-3.68518	-2.8725
H	-2.48997	-4.74262	-1.83102
H	-1.68737	-3.60177	-2.9209
H	-3.8032	0.26957	-1.75286
H	2.21856	0.49228	2.19493
H	5.25845	-0.05024	-2.08464
H	5.99259	-0.3791	1.60221
H	7.51362	-0.07165	-2.40104
H	8.46372	0.73865	-1.1383
H	8.38449	-1.0108	-1.1665
H	7.40204	1.40216	2.5891
H	6.3159	2.06846	1.34412
H	8.04213	1.82046	0.98921
H	6.92945	-2.4376	2.13835
H	7.40904	-2.5839	0.42655
H	8.64339	-2.70131	1.71019

Compound 1c: Isomer 2S, 5R, 11R, 13R, 17S, 23S conformer 16

C	2.73464	-2.24733	-0.11873
C	3.79251	-2.81657	0.51274
C	5.15641	-2.34145	0.30537
C	5.2784	-1.21865	-0.72656
C	4.03354	-0.33406	-0.74741
O	2.83161	-1.13879	-0.88913
C	1.31313	-2.72693	-0.01715
C	0.61497	-2.33093	1.31055
C	0.73453	-0.83725	1.60733
C	0.00205	-0.39833	2.88172
C	0.20851	1.09118	3.2721
C	-0.5937	2.03984	2.38517
C	-0.13943	1.33971	4.74448
O	-0.21384	1.94535	1.08475

O	-1.45901	2.79718	2.76828
C	-0.93773	2.75506	0.11916
C	-0.0851	2.77038	-1.16056
C	-2.31291	2.20766	-0.12281
C	1.30395	3.36443	-0.97076
C	4.04355	0.68261	-1.88598
C	2.78799	1.56128	-2.07606
C	2.47462	2.41333	-0.81831
C	1.47128	4.69112	-0.93517
C	2.97839	2.43232	-3.32711
O	6.13779	-2.82532	0.85376
O	-0.77675	-2.63031	1.2386
C	-3.50812	2.81963	0.08252
O	-4.49595	1.97847	-0.33645
C	-3.84142	0.85382	-0.7743
N	-2.54102	0.9538	-0.67824
C	-4.69801	-0.20218	-1.28225
C	-4.39782	-1.50719	-1.4479
C	-3.05416	-2.11447	-1.06758
C	-5.41441	-2.44225	-2.05294
C	-2.17205	-2.33644	-2.29702
O	-3.22397	-3.39614	-0.44354
C	-3.83145	-3.32344	0.84067
H	3.64424	-3.6885	1.14094
H	6.17096	-0.62501	-0.50726
H	5.42513	-1.68018	-1.7142
H	3.94158	0.17043	0.22547
H	1.28374	-3.81949	-0.11771
H	0.74281	-2.2908	-0.84334
H	1.07856	-2.90655	2.12968
H	0.35157	-0.28523	0.74628
H	1.8	-0.59143	1.70835
H	0.36244	-1.00359	3.72499
H	-1.06879	-0.61042	2.78756
H	1.26828	1.33613	3.10712
H	-0.00604	2.3912	5.01379
H	0.50153	0.73002	5.39016
H	-1.18301	1.07864	4.94764
H	-1.02494	3.76538	0.52859

H	-0.64313	3.35614	-1.9004
H	-0.03573	1.74635	-1.54412
H	4.22663	0.13575	-2.82085
H	4.91855	1.33063	-1.73161
H	1.94077	0.88684	-2.25362
H	2.26568	1.73815	0.02111
H	3.36967	2.99293	-0.55286
H	2.44839	5.14202	-0.78065
H	0.637	5.37988	-1.05413
H	3.18505	1.81332	-4.20815
H	3.8214	3.12455	-3.20036
H	2.09024	3.03517	-3.53795
H	-0.92288	-3.46413	0.76427
H	-3.81242	3.76676	0.49815
H	-5.69507	0.14396	-1.54594
H	-2.52844	-1.44794	-0.37652
H	-6.35511	-1.92708	-2.26801
H	-5.03875	-2.86864	-2.9925
H	-5.61903	-3.29233	-1.39405
H	-1.23264	-2.80907	-1.99412
H	-1.94507	-1.37746	-2.77174
H	-2.6599	-2.99082	-3.02694
H	-3.89176	-4.34942	1.21537
H	-3.22896	-2.71971	1.5308
H	-4.84752	-2.90596	0.79485

Compound 1c: Isomer 2S, 5R, 11R, 13R, 17S, 23S conformer 17

C	-2.7476	-2.10758	-0.59778
C	-3.92832	-2.40476	-1.19791
C	-5.20854	-2.07586	-0.58134
C	-5.09137	-1.45198	0.81021
C	-3.8167	-0.6221	0.95184
O	-2.65123	-1.3915	0.5485
C	-1.39534	-2.47389	-1.14365
C	-0.92156	-1.54857	-2.29936
C	-1.02808	-0.06414	-1.94554
C	-0.5563	0.8772	-3.06117
C	-0.74525	2.38995	-2.76254

C	0.26544	2.90137	-1.74011
C	-0.66674	3.23106	-4.04147
O	0.1214	2.27581	-0.54395
O	1.10066	3.75642	-1.94514
C	1.06914	2.62541	0.5068
C	0.45164	2.15933	1.83331
C	2.40591	1.99466	0.24692
C	-0.91608	2.75936	2.12787
C	-3.57987	-0.13693	2.38013
C	-2.269	0.62401	2.67403
C	-2.14315	1.91938	1.82975
C	-1.01839	3.99434	2.63143
C	-2.189	0.91401	4.18053
O	-6.29444	-2.32194	-1.08975
O	0.40797	-1.90697	-2.68148
C	3.53558	2.58587	-0.22287
O	4.52483	1.64933	-0.25692
C	3.93928	0.49388	0.19766
N	2.67741	0.65481	0.50772
C	4.82923	-0.6455	0.29792
C	4.52388	-1.92197	0.61402
C	3.10992	-2.40804	0.91197
C	5.62209	-2.94533	0.74861
C	2.78871	-2.30382	2.40555
O	2.9428	-3.77649	0.54816
C	2.80537	-3.99395	-0.84495
H	-3.93698	-2.95748	-2.13137
H	-5.97583	-0.83739	1.00327
H	-5.09008	-2.26682	1.54909
H	-3.86859	0.22777	0.25512
H	-1.39836	-3.50337	-1.51483
H	-0.66761	-2.4129	-0.32626
H	-1.53111	-1.76188	-3.18561
H	-0.45819	0.11868	-1.02892
H	-2.07507	0.1641	-1.7079
H	-1.12568	0.65514	-3.97386
H	0.49373	0.6785	-3.30823
H	-1.73863	2.51764	-2.30811
H	-0.78884	4.29602	-3.82499

H	-1.45198	2.92512	-4.74076
H	0.30291	3.10488	-4.53371
H	1.17881	3.71262	0.49609
H	1.16024	2.43956	2.62162
H	0.41503	1.06596	1.82495
H	-3.62633	-1.01247	3.04177
H	-4.43563	0.49885	2.64987
H	-1.43902	-0.04278	2.40861
H	-2.12731	1.64632	0.76683
H	-3.03949	2.53373	1.99213
H	-1.98289	4.453	2.83464
H	-0.1415	4.59844	2.85628
H	-2.27105	-0.01103	4.76314
H	-3.00193	1.5812	4.49632
H	-1.24575	1.39951	4.44811
H	1.00935	-1.53687	-2.01362
H	3.7793	3.57696	-0.56923
H	5.86893	-0.38789	0.11132
H	2.38952	-1.78669	0.36126
H	6.60761	-2.4928	0.60617
H	5.59546	-3.41254	1.74126
H	5.50511	-3.76161	0.02784
H	1.78463	-2.69885	2.58952
H	2.82555	-1.25932	2.72593
H	3.50008	-2.88711	2.99959
H	2.69308	-5.07222	-0.98609
H	1.91621	-3.49137	-1.25322
H	3.68506	-3.65387	-1.41248

Compound 1c: Isomer 2S, 5R, 11R, 13R, 17S, 23S conformer 20

C	2.93644	-2.04926	0.42014
C	4.1562	-2.29594	0.9632
C	5.38856	-1.84386	0.32739
C	5.17544	-1.14713	-1.01637
C	3.84681	-0.39475	-1.05259
O	2.74744	-1.26759	-0.66845
C	1.642	-2.56679	0.9862
C	1.15026	-1.75974	2.217

C	0.88463	-0.29334	1.89133
C	0.46322	0.53566	3.11379
C	0.58349	2.0739	2.94772
C	-0.43965	2.64955	1.97299
C	0.47556	2.79124	4.29852
O	-0.26463	2.16291	0.71895
O	-1.30012	3.45564	2.25863
C	-1.19425	2.63252	-0.30279
C	-0.54889	2.33102	-1.66261
C	-2.52975	1.96694	-0.15302
C	0.81418	2.97583	-1.86579
C	3.52749	0.17726	-2.4307
C	2.18228	0.91227	-2.6113
C	2.04604	2.11948	-1.64673
C	0.91096	4.25987	-2.22746
C	2.04102	1.33311	-4.08207
O	6.50611	-2.04772	0.7831
O	-0.07019	-2.31429	2.71163
C	-3.68096	2.48159	0.35314
O	-4.65137	1.52829	0.25914
C	-4.02937	0.44177	-0.30448
N	-2.76873	0.66369	-0.57286
C	-4.87258	-0.71632	-0.52264
C	-4.48669	-1.96683	-0.85312
C	-3.03178	-2.38034	-1.04234
C	-5.5204	-3.03851	-1.08537
C	-2.61778	-2.3221	-2.51491
O	-2.818	-3.72195	-0.60196
C	-2.76502	-3.85046	0.80939
H	4.23806	-2.90683	1.85603
H	6.01033	-0.4655	-1.20455
H	5.19193	-1.91383	-1.80487
H	3.87227	0.40259	-0.29545
H	1.77454	-3.61312	1.28869
H	0.86815	-2.53516	0.21266
H	1.9262	-1.81883	2.99796
H	0.12569	-0.2381	1.1057
H	1.804	0.12997	1.46897
H	1.10767	0.26473	3.96155

H	-0.55733	0.2712	3.4136
H	1.57176	2.28029	2.51085
H	0.56783	3.87469	4.18182
H	1.26645	2.44518	4.97259
H	-0.49226	2.59355	4.77032
H	-1.31124	3.71006	-0.16341
H	-1.2497	2.69174	-2.42478
H	-0.49859	1.24437	-1.77753
H	3.57336	-0.64811	-3.15409
H	4.34842	0.86123	-2.69085
H	1.38443	0.19498	-2.3812
H	2.02847	1.74369	-0.61561
H	2.93842	2.75344	-1.74452
H	1.87377	4.74593	-2.36423
H	0.03129	4.87789	-2.39681
H	2.14908	0.46963	-4.74903
H	2.81024	2.06685	-4.35682
H	1.06756	1.79204	-4.2789
H	0.11214	-3.21338	3.02718
H	-3.95321	3.4222	0.80333
H	-5.9325	-0.50754	-0.39779
H	-2.38581	-1.69939	-0.47416
H	-6.53559	-2.6401	-1.00028
H	-5.40524	-3.48037	-2.08336
H	-5.40647	-3.86405	-0.37455
H	-1.58178	-2.66149	-2.61465
H	-2.69266	-1.29599	-2.88562
H	-3.25013	-2.97169	-3.12944
H	-2.57921	-4.90793	1.02032
H	-1.95629	-3.24445	1.24293
H	-3.71136	-3.55839	1.29065

Compound 1c: Isomer 2S, 5R, 11R, 13R, 17S, 23S conformer 22

C	5.03239	0.77026	0.43854
C	5.83036	0.73198	-0.65909
C	6.1425	-0.51701	-1.34134
C	5.56524	-1.75743	-0.66086
C	4.23669	-1.45979	0.02982

O	4.3571	-0.30152	0.91436
C	4.80046	2.01991	1.24568
C	3.58777	2.86735	0.7779
C	2.23857	2.25668	1.17636
C	1.02256	2.97855	0.5719
C	0.79002	2.72775	-0.94243
C	0.62154	1.23365	-1.16611
C	-0.38979	3.55851	-1.46749
O	-0.63157	0.81565	-0.87455
O	1.52143	0.48484	-1.49974
C	-0.84355	-0.62941	-0.86223
C	-0.57155	-1.15206	0.56747
C	-2.23904	-0.8839	-1.33097
C	-0.15912	-2.60999	0.57864
C	3.74725	-2.62257	0.8926
C	2.29082	-2.53555	1.39623
C	1.29507	-2.89527	0.25967
C	-1.04291	-3.581	0.83388
C	2.09331	-3.44555	2.61647
O	6.85988	-0.5959	-2.33029
O	3.70905	4.2073	1.25829
C	-2.64915	-1.43835	-2.50177
O	-4.01241	-1.48715	-2.4972
C	-4.38145	-0.94137	-1.29247
N	-3.35676	-0.58158	-0.56471
C	-5.80797	-0.87818	-1.04657
C	-6.44393	-0.24551	-0.03748
C	-5.72708	0.5362	1.0577
C	-7.94514	-0.31846	0.06982
C	-5.51126	-0.31928	2.30873
O	-6.49054	1.67542	1.45867
C	-6.40774	2.75666	0.54993
H	6.3179	1.63747	-1.00368
H	5.43729	-2.54757	-1.40687
H	6.30061	-2.11623	0.07474
H	3.49285	-1.17263	-0.72507
H	5.684	2.65961	1.16555
H	4.6739	1.74475	2.30148
H	3.65084	2.96462	-0.31087

H	2.16665	2.29432	2.27478
H	2.23561	1.19454	0.91179
H	1.14194	4.05817	0.72116
H	0.11423	2.67839	1.109
H	1.69716	2.9994	-1.49242
H	-0.55763	3.38481	-2.53591
H	-0.18435	4.62536	-1.32708
H	-1.31334	3.31139	-0.93596
H	-0.13521	-1.06496	-1.56934
H	-1.46662	-0.98536	1.17317
H	0.23071	-0.53881	0.99152
H	4.43078	-2.69749	1.74845
H	3.87336	-3.54958	0.3156
H	2.11052	-1.49923	1.71238
H	1.5774	-2.33385	-0.64165
H	1.41741	-3.96015	0.02024
H	-0.76006	-4.63092	0.81597
H	-2.08183	-3.36075	1.06706
H	2.74199	-3.13884	3.44516
H	2.33752	-4.48771	2.37024
H	1.05693	-3.42208	2.96754
H	3.6045	4.18076	2.22392
H	-2.14905	-1.83135	-3.37284
H	-6.39689	-1.4169	-1.785
H	-4.74671	0.86226	0.68945
H	-8.37244	-0.95086	-0.71369
H	-8.24709	-0.72401	1.04395
H	-8.39809	0.67672	0.00402
H	-5.02678	0.28514	3.08187
H	-4.87132	-1.17487	2.07449
H	-6.46342	-0.68485	2.7074
H	-6.99734	3.57421	0.97364
H	-5.36797	3.09452	0.41917
H	-6.81336	2.50342	-0.44189

Compound 1c: Isomer 2S, 5R, 11R, 13R, 17S, 23S conformer 25

C	5.09547	-0.59792	-0.53721
C	5.8613	-0.7083	0.57523

C	6.14349	0.43821	1.43538
C	5.54652	1.75478	0.93817
C	4.22798	1.52576	0.20643
O	4.40106	0.53242	-0.85247
C	4.92915	-1.67269	-1.57522
C	3.46995	-2.12827	-1.82451
C	2.76386	-2.62197	-0.55157
C	1.41353	-3.28549	-0.85807
C	0.52475	-3.51833	0.38382
C	0.22169	-2.1911	1.06605
C	-0.77229	-4.26182	0.01812
O	-0.42542	-1.35058	0.22402
O	0.54338	-1.89481	2.19897
C	-0.62895	0.01998	0.68477
C	-0.50748	0.90434	-0.5679
C	-1.94349	0.1303	1.3936
C	-0.22719	2.35736	-0.23415
C	3.66698	2.78562	-0.44916
C	2.22282	2.69329	-0.98823
C	1.19141	2.70773	0.17211
C	-1.19314	3.28077	-0.28556
C	1.9551	3.83166	-1.98246
O	6.84796	0.3818	2.43325
O	2.71834	-1.11083	-2.48331
C	-2.17827	0.0888	2.73155
O	-3.52285	0.20193	2.93175
C	-4.06201	0.30164	1.67314
N	-3.15587	0.27058	0.73027
C	-5.50402	0.42771	1.62158
C	-6.30179	0.37205	0.53419
C	-5.77245	0.18676	-0.87633
C	-7.79217	0.52664	0.68039
C	-5.46539	1.54374	-1.532
O	-6.76703	-0.53439	-1.61022
C	-6.2702	-1.20975	-2.74655
H	6.35762	-1.64583	0.79868
H	5.39985	2.4255	1.78989
H	6.27311	2.23131	0.26387
H	3.50606	1.08702	0.91013

H	5.54178	-2.53188	-1.28465
H	5.32087	-1.29626	-2.52988
H	3.51658	-2.95701	-2.54279
H	2.62509	-1.76511	0.12195
H	3.41646	-3.32557	-0.01574
H	1.58794	-4.25603	-1.3428
H	0.86657	-2.6642	-1.57441
H	1.07838	-4.1021	1.12805
H	-1.39696	-4.43225	0.90175
H	-0.53496	-5.23722	-0.4209
H	-1.35723	-3.6904	-0.70913
H	0.16842	0.23305	1.4005
H	-1.42748	0.81216	-1.15096
H	0.3063	0.48323	-1.17065
H	4.34917	3.05933	-1.26452
H	3.72231	3.59973	0.2869
H	2.12228	1.74497	-1.53346
H	1.52336	2.00675	0.95173
H	1.20556	3.70489	0.63176
H	-1.004	4.32079	-0.02987
H	-2.20884	3.02624	-0.57626
H	2.62944	3.76896	-2.84417
H	2.10964	4.8093	-1.50682
H	0.92573	3.80018	-2.35192
H	2.93361	-0.27676	-2.03267
H	-1.55524	-0.02329	3.60421
H	-5.95991	0.57109	2.59793
H	-4.84522	-0.3992	-0.84006
H	-8.07435	0.7085	1.72144
H	-8.16382	1.35913	0.06868
H	-8.30631	-0.37011	0.31964
H	-5.10897	1.40686	-2.55864
H	-4.6838	2.06287	-0.96994
H	-6.36195	2.17209	-1.56209
H	-7.10731	-1.77155	-3.17027
H	-5.88684	-0.52255	-3.5156
H	-5.46619	-1.914	-2.4803

Compound 1c: Isomer 2S, 5R, 11R, 13R, 17S, 23S conformer 28

C	5.02726	0.75077	0.43457
C	5.82059	0.7151	-0.66708
C	6.12464	-0.53012	-1.35889
C	5.54617	-1.77219	-0.68289
C	4.22252	-1.47436	0.01739
O	4.35011	-0.31993	0.90617
C	4.80323	1.99653	1.24977
C	3.59959	2.85805	0.78066
C	2.25202	2.27606	1.2065
C	1.03476	2.99648	0.60282
C	0.79138	2.74746	-0.91035
C	0.62715	1.25382	-1.13842
C	-0.39552	3.57509	-1.42461
O	-0.62518	0.83144	-0.84948
O	1.52907	0.50866	-1.47505
C	-0.83533	-0.61364	-0.84644
C	-0.57132	-1.14352	0.58171
C	-2.22735	-0.86812	-1.32569
C	-0.17067	-2.60472	0.58849
C	3.73639	-2.63936	0.87902
C	2.28389	-2.54934	1.39293
C	1.27948	-2.90131	0.26176
C	-1.06062	-3.56916	0.84706
C	2.09101	-3.46312	2.61101
O	6.83699	-0.60485	-2.35202
O	3.67309	4.16078	1.3637
C	-2.62818	-1.4165	-2.50255
O	-3.99134	-1.46937	-2.50733
C	-4.36978	-0.93221	-1.30159
N	-3.35096	-0.57383	-0.56499
C	-5.79806	-0.87507	-1.06451
C	-6.44255	-0.25028	-0.05593
C	-5.73558	0.52775	1.04825
C	-7.94418	-0.32874	0.04114
C	-5.5254	-0.33357	2.29621
O	-6.50569	1.66232	1.4498
C	-6.41898	2.74907	0.54808
H	6.31248	1.61952	-1.00868

H	5.4109	-2.55738	-1.43283
H	6.28452	-2.13826	0.0461
H	3.47437	-1.18207	-0.73111
H	5.70555	2.61504	1.18054
H	4.66555	1.72991	2.30382
H	3.64751	2.93229	-0.3165
H	2.20208	2.35216	2.30052
H	2.237	1.20967	0.96184
H	1.15015	4.07548	0.756
H	0.13052	2.69285	1.1451
H	1.69303	3.02309	-1.46775
H	-0.57087	3.40482	-2.49247
H	-0.19425	4.6424	-1.28096
H	-1.31402	3.32234	-0.88714
H	-0.12199	-1.04421	-1.55162
H	-1.46664	-0.97214	1.1857
H	0.23454	-0.53788	1.00997
H	4.42566	-2.71925	1.72979
H	3.85594	-3.56449	0.29756
H	2.10947	-1.51367	1.71406
H	1.56143	-2.34206	-0.64106
H	1.39231	-3.967	0.0212
H	-0.78593	-4.62122	0.82674
H	-2.0966	-3.34109	1.08565
H	2.74508	-3.16094	3.43708
H	2.33076	-4.50514	2.35974
H	1.05672	-3.43764	2.96803
H	4.43409	4.62123	0.97727
H	-2.1212	-1.80281	-3.37264
H	-6.38045	-1.41149	-1.80977
H	-4.75382	0.85891	0.68843
H	-8.36435	-0.95765	-0.74903
H	-8.25113	-0.74127	1.01076
H	-8.39981	0.66543	-0.02138
H	-5.048	0.26834	3.07565
H	-4.88107	-1.18582	2.06178
H	-6.47899	-0.70435	2.68657
H	-7.01405	3.5625	0.97207
H	-5.37921	3.09055	0.42727

H	-6.8164	2.50049	-0.44829

Compound 1c: Isomer 2S, 5R, 11R, 13R, 17S, 23S conformer 33

C	5.01932	-0.38132	0.08736
C	5.61308	-0.15452	1.2876
C	5.69724	1.17996	1.86801
C	5.13355	2.28012	0.97013
C	3.95922	1.77254	0.13864
O	4.32182	0.56105	-0.59075
C	5.08952	-1.70098	-0.63142
C	3.83168	-2.6067	-0.52303
C	2.70722	-2.18659	-1.4704
C	1.48046	-3.11465	-1.46548
C	0.77061	-3.28413	-0.1018
C	0.48299	-1.92794	0.52103
C	-0.50876	-4.12725	-0.24221
O	-0.37512	-1.20775	-0.23969
O	0.98893	-1.51115	1.54523
C	-0.6063	0.17753	0.16921
C	-0.72098	0.99647	-1.12553
C	-1.81252	0.25152	1.0532
C	-0.44586	2.47477	-0.92754
C	3.4715	2.7854	-0.8968
C	2.08417	2.50514	-1.5123
C	0.95744	2.8796	-0.51452
C	-1.41022	3.38299	-1.1104
C	1.91978	3.26103	-2.83808
O	6.23254	1.42753	2.94048
O	4.17994	-3.94622	-0.88171
C	-1.85558	0.23344	2.41215
O	-3.16241	0.29066	2.79725
C	-3.87627	0.3367	1.62514
N	-3.11037	0.32249	0.56543
C	-5.31598	0.40248	1.77669
C	-6.25512	0.32247	0.81015
C	-5.93314	0.15695	-0.67071
C	-7.71485	0.43826	1.16533
C	-5.90596	1.50779	-1.39084

O	-6.91857	-0.64383	-1.32609
C	-6.76702	-2.02997	-1.08734
H	6.12653	-0.962	1.79769
H	4.82416	3.12702	1.58986
H	5.93935	2.6321	0.30913
H	3.14887	1.47515	0.81999
H	5.93562	-2.25753	-0.21332
H	5.30973	-1.52638	-1.69186
H	3.4708	-2.57309	0.51558
H	3.115	-2.15953	-2.48955
H	2.41709	-1.15816	-1.22654
H	1.78559	-4.1092	-1.80709
H	0.75283	-2.73114	-2.19091
H	1.44899	-3.78345	0.59722
H	-0.99313	-4.28358	0.72805
H	-0.26376	-5.11025	-0.65919
H	-1.22747	-3.64006	-0.90826
H	0.2672	0.47391	0.75153
H	-1.71591	0.84837	-1.55319
H	0.00388	0.57299	-1.83121
H	4.22975	2.82536	-1.68933
H	3.46278	3.77848	-0.42633
H	2.03104	1.42928	-1.72621
H	1.18822	2.43095	0.46279
H	0.98227	3.96622	-0.35778
H	-1.22618	4.44703	-0.98132
H	-2.42002	3.0935	-1.38959
H	2.67054	2.94192	-3.57004
H	2.03719	4.34259	-2.68843
H	0.92811	3.09536	-3.27137
H	4.77422	-4.29526	-0.19956
H	-1.11154	0.17376	3.19021
H	-5.63299	0.53367	2.80849
H	-4.94783	-0.31247	-0.77819
H	-7.8536	0.62043	2.23497
H	-8.18798	1.25883	0.61089
H	-8.2644	-0.46818	0.88908
H	-5.70355	1.34878	-2.45464
H	-5.12105	2.14291	-0.96968

H	-6.86765	2.02361	-1.29914
H	-7.54918	-2.53853	-1.65764
H	-5.78373	-2.39214	-1.42552
H	-6.87962	-2.28769	-0.0227

Compound 1c: Isomer 2S, 5R, 11R, 13R, 17S, 23S conformer 39

C	4.86318	-0.97567	-0.3385
C	5.13358	-1.31825	-1.62421
C	4.48034	-2.44552	-2.27564
C	3.54865	-3.23724	-1.35916
C	2.88877	-2.34613	-0.30945
O	3.88755	-1.54852	0.40138
C	5.61671	0.09412	0.40643
C	5.00807	1.51587	0.27086
C	3.77154	1.71777	1.14652
C	3.03418	3.0457	0.90573
C	2.28082	3.16404	-0.44782
C	1.3041	2.00687	-0.56353
C	1.62495	4.54371	-0.59888
O	0.13488	2.25562	0.07742
O	1.56168	0.94791	-1.10132
C	-0.78605	1.13912	0.20482
C	-0.5312	0.4719	1.58298
C	-2.16842	1.6866	0.03007
C	-0.9925	-0.96794	1.66901
C	2.13234	-3.14322	0.75265
C	1.20448	-2.32772	1.67839
C	-0.13131	-1.9931	0.95826
C	-2.0877	-1.30828	2.357
C	0.95452	-3.08575	2.98948
O	4.69968	-2.78914	-3.43011
O	5.95683	2.499	0.69029
C	-2.6215	2.96209	0.15642
O	-3.96532	2.9583	-0.0983
C	-4.27242	1.65087	-0.36828
N	-3.22885	0.86273	-0.31274
C	-5.66156	1.38078	-0.67822
C	-6.25043	0.17691	-0.84312

C	-5.51001	-1.15105	-0.73028
C	-7.71241	0.09521	-1.1991
C	-5.05572	-1.65646	-2.10216
O	-6.3527	-2.16176	-0.17088
C	-6.49474	-2.06103	1.23244
H	5.91191	-0.79651	-2.17067
H	2.78715	-3.73501	-1.96699
H	4.14195	-4.02309	-0.86809
H	2.23832	-1.61697	-0.81015
H	6.63819	0.12826	0.01041
H	5.68274	-0.17015	1.46798
H	4.74812	1.66901	-0.78735
H	4.10935	1.68777	2.19067
H	3.09945	0.8658	1.00616
H	3.75639	3.86787	0.96279
H	2.3077	3.2036	1.71323
H	2.99455	3.01427	-1.2647
H	1.08287	4.62977	-1.54679
H	2.39309	5.32439	-0.57678
H	0.91916	4.73757	0.21448
H	-0.57446	0.43104	-0.5982
H	-1.00231	1.08135	2.36102
H	0.55195	0.51397	1.75132
H	2.88414	-3.67189	1.35296
H	1.54344	-3.91858	0.2428
H	1.72188	-1.3905	1.92352
H	0.09667	-1.63174	-0.05371
H	-0.69821	-2.92531	0.83469
H	-2.42422	-2.34038	2.41935
H	-2.69066	-0.5676	2.87625
H	1.89149	-3.24661	3.53512
H	0.50957	-4.07052	2.79316
H	0.26987	-2.53574	3.64267
H	6.68488	2.50791	0.0497
H	-2.16709	3.91368	0.37596
H	-6.262	2.28069	-0.78773
H	-4.62245	-1.02282	-0.09937
H	-8.14866	1.08947	-1.33241
H	-7.85583	-0.47293	-2.12706

H	-8.28095	-0.43742	-0.42895
H	-4.56775	-2.62924	-1.98656
H	-4.34396	-0.95376	-2.54453
H	-5.90634	-1.77861	-2.78104
H	-7.12302	-2.89775	1.55029
H	-5.52083	-2.12958	1.74194
H	-6.97632	-1.11945	1.53975

Compound 1c: Isomer 2S, 5R, 11R, 13R, 17S, 23S conformer 45

C	2.87806	-2.43016	-0.25443
C	3.87862	-3.1479	0.31786
C	5.28209	-2.85959	0.05119
C	5.51282	-1.75429	-0.98195
C	4.39889	-0.70876	-0.95803
O	3.09465	-1.34066	-1.03237
C	1.40878	-2.68623	-0.0752
C	0.81812	-2.20551	1.29063
C	1.42823	-0.87839	1.76561
C	0.7617	-0.306	3.02616
C	1.0468	1.20454	3.27578
C	0.16931	2.0552	2.36503
C	0.81991	1.59517	4.73949
O	0.52089	1.89306	1.05917
O	-0.74672	2.76453	2.7165
C	-0.33853	2.46919	0.05119
C	0.42961	2.36337	-1.28036
C	-1.65499	1.74531	0.00087
C	1.79655	3.03609	-1.25768
C	4.49509	0.29007	-2.11161
C	3.30007	1.24278	-2.33468
C	3.01558	2.14643	-1.10695
C	1.8986	4.36552	-1.36294
C	3.55053	2.07356	-3.60256
O	6.21431	-3.47482	0.55318
O	-0.59552	-2.12139	1.17154
C	-2.9055	2.26715	-0.0607
O	-3.79828	1.23121	-0.1444
C	-3.03702	0.09859	-0.12473

N	-1.75319	0.35929	-0.04116
C	-3.62794	-1.22355	-0.15392
C	-4.90975	-1.59011	-0.37144
C	-6.05696	-0.62355	-0.6524
C	-5.28466	-3.04878	-0.30037
C	-6.8296	-0.28113	0.62492
O	-6.99717	-1.19133	-1.5637
C	-6.56273	-1.17232	-2.91022
H	3.64087	-3.99344	0.9546
H	6.48549	-1.28815	-0.79724
H	5.55872	-2.22604	-1.9746
H	4.42179	-0.18724	0.0102
H	1.18646	-3.75191	-0.18992
H	0.87763	-2.14951	-0.86793
H	1.00292	-2.98318	2.04214
H	1.34669	-0.15909	0.94754
H	2.49954	-1.02399	1.95314
H	1.11011	-0.86113	3.90686
H	-0.32116	-0.4655	2.97488
H	2.09174	1.40572	3.00109
H	0.9977	2.6628	4.90026
H	1.49661	1.02912	5.38828
H	-0.20991	1.38345	5.04394
H	-0.51591	3.51867	0.30467
H	-0.2053	2.81999	-2.04869
H	0.51844	1.3019	-1.53451
H	4.64926	-0.2795	-3.03804
H	5.41135	0.87785	-1.95632
H	2.41774	0.61407	-2.50767
H	2.87284	1.5135	-0.22242
H	3.89783	2.77255	-0.91562
H	2.86023	4.87115	-1.32655
H	1.02482	5.00307	-1.48437
H	3.70311	1.42579	-4.47381
H	4.44621	2.6993	-3.49181
H	2.70975	2.73974	-3.81765
H	-0.80447	-1.28402	0.70392
H	-3.31788	3.26286	-0.0349
H	-2.89157	-1.99662	0.05087

H	-5.66291	0.30504	-1.08262
H	-4.4282	-3.66674	-0.01799
H	-6.08409	-3.20956	0.43394
H	-5.67799	-3.40695	-1.258
H	-7.65891	0.3896	0.37984
H	-6.17333	0.21277	1.34739
H	-7.24476	-1.18343	1.08514
H	-7.37077	-1.59846	-3.51061
H	-6.36455	-0.14509	-3.25464
H	-5.65111	-1.76936	-3.06574

Compound 1c: Isomer 2S, 5R, 11R, 13R, 17S, 23S conformer 49

C	-5.00604	0.36123	0.07376
C	-5.60298	0.09985	1.26533
C	-5.67844	-1.24926	1.81202
C	-5.10129	-2.32207	0.88988
C	-3.92768	-1.78408	0.07663
O	-4.29788	-0.55831	-0.62446
C	-5.08413	1.69769	-0.61245
C	-3.83304	2.60912	-0.47804
C	-2.70193	2.21673	-1.42906
C	-1.4783	3.14829	-1.39448
C	-0.77768	3.28921	-0.02226
C	-0.49796	1.91868	0.57205
C	0.50216	4.13514	-0.13556
O	0.38178	1.2221	-0.18599
O	-1.0291	1.47198	1.57056
C	0.60909	-0.17234	0.1923
C	0.76103	-0.9558	-1.1206
C	1.79104	-0.26499	1.10609
C	0.49019	-2.44054	-0.97051
C	-3.42629	-2.76642	-0.98145
C	-2.03552	-2.46197	-1.57803
C	-0.91515	-2.86438	-0.58393
C	1.45979	-3.33914	-1.17237
C	-1.85809	-3.17484	-2.92571
O	-6.21658	-1.52808	2.87537
O	-4.18858	3.95392	-0.80918

C	1.79396	-0.31288	2.46463
O	3.09013	-0.36149	2.88583
C	3.8388	-0.33363	1.7351
N	3.10396	-0.28393	0.65416
C	5.27397	-0.36609	1.92652
C	6.23744	-0.21323	0.99363
C	5.94118	-0.01151	-0.48155
C	7.68907	-0.27374	1.38717
C	5.83645	-1.36377	-1.20757
O	6.99634	0.79129	-1.02002
C	6.65474	1.47206	-2.20895
H	-6.12497	0.89035	1.793
H	-4.78749	-3.18145	1.48991
H	-5.90103	-2.66432	0.21651
H	-3.12306	-1.49623	0.76876
H	-5.9355	2.23775	-0.18359
H	-5.29975	1.54764	-1.67761
H	-3.47647	2.55511	0.56106
H	-3.10401	2.21367	-2.45086
H	-2.40978	1.18355	-1.2093
H	-1.78431	4.14947	-1.71536
H	-0.74454	2.78364	-2.12353
H	-1.46181	3.7736	0.68156
H	0.97929	4.27143	0.84124
H	0.2598	5.12652	-0.53385
H	1.22636	3.66218	-0.80579
H	-0.27773	-0.48834	0.74319
H	1.76561	-0.79041	-1.51865
H	0.05152	-0.51741	-1.83299
H	-4.17812	-2.78921	-1.78073
H	-3.41553	-3.77175	-0.53787
H	-1.98467	-1.37967	-1.75739
H	-1.15614	-2.44986	0.40579
H	-0.93641	-3.95576	-0.464
H	1.27869	-4.40737	-1.07867
H	2.47123	-3.03792	-1.43266
H	-2.60382	-2.83492	-3.65344
H	-1.97317	-4.26092	-2.81129
H	-0.86335	-2.99237	-3.34506

H	-4.78505	4.28538	-0.12021
H	1.0268	-0.30652	3.22222
H	5.56751	-0.52303	2.96125
H	4.98549	0.51659	-0.5901
H	7.80547	-0.47855	2.45536
H	8.21812	-1.0531	0.8234
H	8.18931	0.66959	1.14504
H	5.659	-1.21683	-2.27849
H	5.0012	-1.94199	-0.80197
H	6.76084	-1.9392	-1.08965
H	7.51158	2.09925	-2.47046
H	6.45457	0.78852	-3.0477
H	5.77149	2.11516	-2.06848

Compound 1c: Isomer 2S, 5R, 11R, 13R, 17S, 23S conformer 55

C	3.50777	-2.30004	-0.4896
C	4.63516	-2.78247	0.09886
C	5.94198	-2.17441	-0.12195
C	5.92179	-0.98946	-1.08842
C	4.5954	-0.2401	-0.99801
O	3.48003	-1.14952	-1.19605
C	2.17531	-2.99318	-0.42621
C	1.03069	-2.21868	0.27359
C	1.50836	-1.4465	1.5187
C	0.40232	-1.14403	2.54866
C	0.65796	0.13796	3.39887
C	-0.02897	1.32552	2.73429
C	0.18584	-0.00152	4.84948
O	0.34672	1.44186	1.43121
O	-0.84841	2.05103	3.25293
C	-0.47987	2.28195	0.58609
C	0.27223	2.44895	-0.74461
C	-1.81111	1.61579	0.38675
C	1.59347	3.19555	-0.62433
C	4.45379	0.8814	-2.02097
C	3.11479	1.64868	-2.02375
C	2.8689	2.38121	-0.6781
C	1.6109	4.52478	-0.4766

C	3.09104	2.6205	-3.21284
O	6.9828	-2.59402	0.36859
O	0.44333	-1.37317	-0.70406
C	-3.06568	2.10014	0.56168
O	-3.95569	1.11621	0.21259
C	-3.18673	0.053	-0.16275
N	-1.9045	0.30759	-0.06931
C	-3.75791	-1.20497	-0.59395
C	-5.04718	-1.55343	-0.7944
C	-6.2422	-0.628	-0.57796
C	-5.37877	-2.96174	-1.21803
C	-6.84432	-0.80867	0.81879
O	-7.28176	-0.90309	-1.51527
C	-7.0366	-0.37333	-2.80498
H	4.5923	-3.693	0.68609
H	6.76148	-0.32525	-0.86211
H	6.06822	-1.37258	-2.10908
H	4.48743	0.14988	0.02474
H	2.32392	-3.95006	0.0841
H	1.83379	-3.20861	-1.44616
H	0.29942	-2.98426	0.59117
H	1.9522	-0.51486	1.15573
H	2.31025	-2.00548	2.01902
H	0.30573	-1.99625	3.23281
H	-0.5699	-1.05147	2.04846
H	1.73832	0.34053	3.39307
H	0.34478	0.92382	5.41027
H	0.73388	-0.80948	5.34581
H	-0.88371	-0.23255	4.89311
H	-0.62111	3.2474	1.07893
H	-0.40123	2.98932	-1.42048
H	0.41896	1.45032	-1.16721
H	4.61144	0.44892	-3.0182
H	5.28134	1.58614	-1.85385
H	2.31976	0.90598	-2.16263
H	2.83519	1.63769	0.12891
H	3.72241	3.04163	-0.47095
H	2.54133	5.07747	-0.37467
H	0.69575	5.11344	-0.44767

H	3.24937	2.08703	-4.15747
H	3.88151	3.37739	-3.12001
H	2.13701	3.15226	-3.28115
H	-0.31505	-0.88076	-0.32689
H	-3.48248	3.02872	0.91649
H	-2.98231	-1.94693	-0.76677
H	-5.92833	0.4163	-0.69185
H	-4.47994	-3.58057	-1.28758
H	-6.06788	-3.43131	-0.50456
H	-5.89089	-2.9762	-2.18605
H	-7.71449	-0.15402	0.92686
H	-6.1093	-0.55352	1.58794
H	-7.17224	-1.8416	0.97323
H	-7.90637	-0.6195	-3.41956
H	-6.91801	0.72101	-2.7743
H	-6.1376	-0.80406	-3.27176

Compound 1c: Isomer 2S, 5R, 11R, 13R, 17S, 23S conformer 57

C	2.95366	-2.39186	-0.41162
C	3.96261	-3.13259	0.11496
C	5.36282	-2.80739	-0.12497
C	5.58181	-1.63042	-1.07832
C	4.45235	-0.60554	-0.98998
O	3.15782	-1.24953	-1.11371
C	1.48755	-2.68049	-0.25686
C	0.88306	-2.29331	1.13291
C	1.46807	-0.98744	1.68961
C	0.79069	-0.50581	2.98233
C	1.04774	0.99099	3.32443
C	0.15774	1.87948	2.46356
C	0.80984	1.28771	4.80843
O	0.5232	1.81113	1.15361
O	-0.77713	2.54343	2.85237
C	-0.33979	2.43449	0.17734
C	0.44026	2.42863	-1.15163
C	-1.64167	1.6913	0.06667
C	1.79712	3.11659	-1.07205
C	4.54175	0.46908	-2.07429

C	3.33503	1.41714	-2.24738
C	3.02676	2.23532	-0.96655
C	1.88228	4.4512	-1.09167
C	3.58655	2.33139	-3.45617
O	6.3018	-3.44301	0.33759
O	-0.53124	-2.22628	1.01225
C	-2.90033	2.19413	0.02141
O	-3.77279	1.15026	-0.14534
C	-2.99067	0.03222	-0.18967
N	-1.71304	0.30957	-0.06966
C	-3.55516	-1.29333	-0.32855
C	-4.82918	-1.66859	-0.57389
C	-5.99136	-0.70199	-0.73668
C	-5.16811	-3.1334	-0.66203
C	-6.65145	-0.40155	0.62088
O	-6.91562	-1.30458	-1.64545
C	-7.80183	-0.39372	-2.2617
H	3.73544	-4.02346	0.69084
H	6.5464	-1.1637	-0.85636
H	5.63968	-2.03206	-2.10073
H	4.46318	-0.14942	0.0109
H	1.28248	-3.74041	-0.43768
H	0.9517	-2.10426	-1.0181
H	1.07859	-3.11191	1.83675
H	1.37377	-0.22133	0.91663
H	2.54172	-1.12438	1.87
H	1.14922	-1.10783	3.82746
H	-0.28906	-0.68181	2.91975
H	2.08964	1.2272	3.06677
H	0.97221	2.34583	5.03534
H	1.49222	0.69244	5.42433
H	-0.21794	1.0438	5.09526
H	-0.53892	3.4621	0.49602
H	-0.19524	2.92679	-1.89316
H	0.54564	1.38776	-1.47524
H	4.71328	-0.03589	-3.03453
H	5.44735	1.05893	-1.87144
H	2.46364	0.78886	-2.46994
H	2.8853	1.54396	-0.12658

H	3.89858	2.85946	-0.727
H	2.83686	4.9653	-1.01331
H	1.00132	5.08401	-1.18148
H	3.76318	1.7431	-4.36429
H	4.4687	2.96473	-3.29269
H	2.7364	2.99457	-3.6417
H	-0.75234	-1.36579	0.59495
H	-3.33127	3.17859	0.10647
H	-2.8019	-2.06648	-0.20275
H	-5.63131	0.23941	-1.17038
H	-4.2925	-3.75679	-0.46254
H	-5.95556	-3.39851	0.05501
H	-5.56502	-3.37408	-1.65364
H	-7.51003	0.26696	0.49641
H	-5.93923	0.0855	1.29324
H	-7.00312	-1.32626	1.08976
H	-8.39041	-0.96698	-2.98296
H	-8.4907	0.07914	-1.54628
H	-7.25812	0.40114	-2.79717
